# Polarization-controlled Floquet engineering in twisted photonic lattices

**DOI:** 10.1038/s41467-026-78660-0

**Published:** 2026-10-15

**Authors:** Jinze Gao, Guibin Li, Zhuochen Du, Kun Liao, Kunyu Yu, C. T. Chan, Ruo-Yang Zhang, Daopeng Yuan, Yan Zhang, Xiaoyong Hu, Qihuang Gong

**Affiliations:** 1 State Key Laboratory for Mesoscopic Physics & Department of Physics, Collaborative Innovation Center of Quantum Matter & Frontiers Science Center for Nano-optoelectronics, Peking University, Beijing, PR China; 2 Department of Physics, Beijing Key Lab for Metamaterials and Devices, Key Laboratory for Terahertz Optoelectronics, Ministry of Education, Beijing Advanced Innovation Center for Imaging Theory and Technology, Capital Normal University, Beijing, PR China; 3 Department of Physics, The Hong Kong University of Science and Technology, Clear Water Bay, Kowloon, PR China; 4 National Laboratory of Solid State Microstructures, School of Physics, Nanjing University, Nanjing, PR China; 5 Taishan College, Shandong University, Jinan, PR China; 6 Key Laboratory for Advanced Optoelectronic Integrated Chips of Jiangsu Province, Peking University Yangtze Delta Institute of Optoelectronics, Nantong, PR China; 7 Collaborative Innovation Center of Extreme Optics, Shanxi University, Taiyuan, PR China; 8 Hefei National Laboratory, Hefei, PR China; 9 Present Address: Department of Chemistry, Massachusetts Institute of Technology, Cambridge, MA USA

**Keywords:** Terahertz optics, Integrated optics

## Abstract

Floquet engineering has emerged as a powerful approach for both classical and quantum systems, enabling versatile exploration of synthetic dimensions, topological phases, and quantum information processing. Further advances, however, require precise and continuous control over both the magnitude and sign of the coupling coefficient. Although static modal-engineering approaches can tune both, existing Floquet photonic platforms typically rely on geometric modulation, auxiliary structures, or phase engineering. Continuous tuning of the coupling coefficient in both magnitude and sign within a Floquet period therefore remains challenging. Here, we present a compact Floquet platform based on twisted photonic waveguide arrays, in which the twist-induced variation of the relative polarization angle between adjacent waveguides directly tunes the coupling coefficient through zero, allowing continuous access to positive and negative values with different magnitudes. To highlight the core capability of the platform, we focus on representative driven phenomena in both one- and two-dimensional lattices: Floquet flat bands and the associated period-nΛ oscillations in one-dimensional lattices, as well as chirality reversal of topological edge states in two-dimensional lattices.

## Abstract (web summary)

The authors demonstrate a polarization-controlled Floquet photonic platform in which twisted waveguides tune coupling in both magnitude and sign, enabling flat-band dynamics and chirality reversal of topological states.

## Introduction

Floquet engineering, particularly along the propagation direction of photonic lattices, has opened up powerful avenues for dynamically tailoring the behavior of both quantum and classical systems^1–8^. Periodically curved or helical waveguides^9–11^ have enabled a range of topological phenomena^9,12–14^, non-Abelian braiding^6,15^, and artificial gauge fields^16–18^. Multi-step driving protocols^19,20^ have further expanded the scope of Floquet photonics^21,22^, leading to non-Hermitian topological phases^23^, Floquet solitons^24,25^, and higher-order corner-state oscillations^26^. More recently, multi-orbital interactions have been incorporated into a Floquet protocol based on the periodic concatenation of two photonic masks, enabling phase-controlled transport of compact photonic states^27,28^. In parallel, coupled micro-ring resonators and fiber-loop lattices have been explored to realize a variety of Floquet-driven photonic phenomena^29–32^. However, coupling control in most existing photonic platforms still relies on structural modulation, auxiliary structures, phase engineering, or the concatenation of discrete photonic masks. Efficient and compact continuous tuning of the coupling coefficient in both magnitude and sign remains experimentally challenging, limiting the exploration of broader physical phenomena.

Polarization is a fundamental internal degree of freedom of light and has long served as a powerful resource for optical control through geometric phases and anisotropic responses^33–35^. More broadly, internal modal degrees of freedom have enabled several nontrivial coupling mechanisms in photonic systems, including tensorial dipole-dipole coupling in dipolar arrays^36^, orbital-parity-induced negative coupling in mixed-s/p lattices^37^, and orientation-controlled magneto-electric coupling in bianisotropic meta-atoms^38^. Related static modal-engineering studies have further demonstrated continuous tuning of the effective coupling coefficient in both magnitude and sign by varying the relative orientation of higher-order waveguide modes^39,40^. These studies highlight the potential of internal degrees of freedom for engineering effective coupling, synthetic flux, and topological states. However, their mechanisms rely on dipolar tensor interactions, engineered orbital parity, or resonant electric magnetic hybridization, rather than on a direct mapping from polarization to inter waveguide coupling. A platform in which the linear polarization of a guided fundamental mode directly controls the modal overlap between adjacent waveguides would provide a compact and geometrically programmable route to continuously control both the magnitude and sign of the nearest-neighbour coupling coefficient, offering a natural driving parameter for Floquet photonic lattices.

**Fig. 1:**
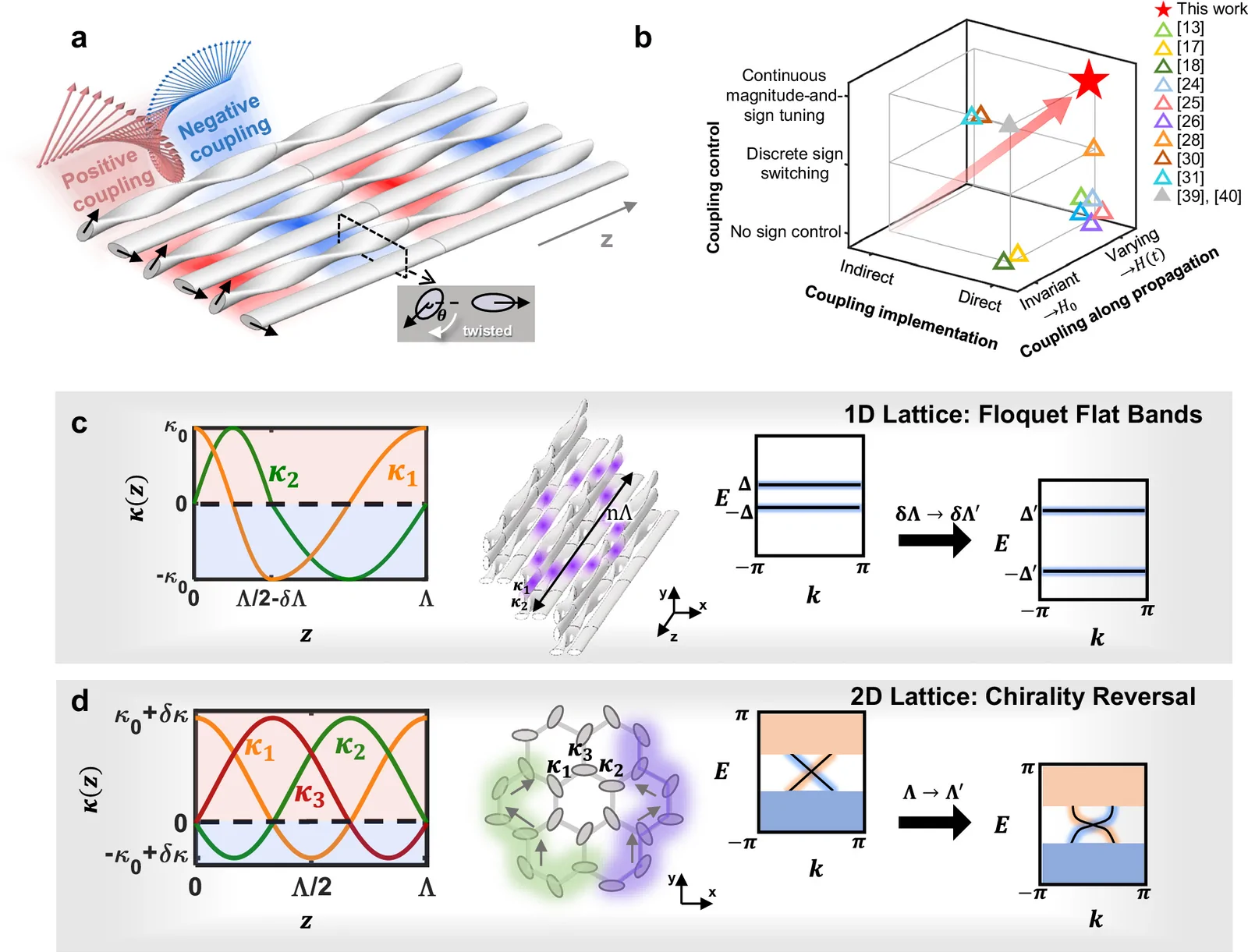
Polarization-controlled Floquet engineering enabled by twisted photonic lattices. **a** Concept of polarization-controlled coupling in twisted waveguide arrays. As the waveguides twist along z, the relative polarization angle θ between adjacent waveguides evolves continuously, allowing the nearest-neighbour coupling coefficient κ(z) to be tuned from positive to negative values. Red and blue denote positive and negative coupling, respectively. **b** Comparison with representative Floquet photonic platforms. The solid gray triangle represents a related non-Floquet modal-engineering platform shown for comparison. The present Floquet photonic platform enables direct nearest-neighbour coupling whose magnitude and sign can be continuously tuned along the propagation direction within each Floquet period through polarization control. **c** 1D lattice for Floquet flat-band engineering. Left: alternating couplings ${\kappa }_{1}$ and ${\kappa }_{2}$. Middle: twisted waveguide array. Right: quasi-energy spectrum exhibits Floquet flat bands at quasi-energies $\pm \Delta$, $\pm \Delta {\prime}$, …, where different flat-band quasi-energies correspond to period-nΛ oscillations with different integer n. **d** 2D lattice for chirality reversal of topological edge states. Left: three couplings, ${\kappa }_{1}$, ${\kappa }_{2}$, and ${\kappa }_{3}$. Middle: twisted waveguide array. Right: the quasi-energy spectrum supports edge states, whose chirality is reversed when the driving period is changed from Λ to ${\Lambda }^{{\prime} }$.

Here, we propose a twisted photonic waveguide platform for Floquet engineering, in which the relative polarization angle between adjacent waveguides is controlled geometrically by the twist, as illustrated in Fig. 1a. We demonstrate that twist-controlled modulation of the relative polarization angle between neighboring waveguides enables continuous tuning of the coupling coefficient in both magnitude and sign within a compact propagation platform. Fabricated through high-precision 3D printing, these twisted waveguide arrays with polarization-controlled coupling provide a versatile platform for exploring diverse physical phenomena. We showcase the broad utility of this platform through experimental demonstrations of representative driven phenomena in one-dimensional lattices and local multidirectional coupling in a two-dimensional configuration, together with a theoretical prediction of topological edge transport in two-dimensional lattices. Specifically, we first realize Floquet flat bands that extend across the entire Brillouin zone, where the quasi-energy levels can be adjusted by varying the twisting rates of positive and negative coupling segments, giving rise to period-nΛ oscillations and self-imaging in one-dimensional (1D) lattices (Fig. 1c). Second, exploiting the ability to realize positive and negative couplings with unequal magnitudes, we theoretically predict the chirality reversal of the topological edge states in two-dimensional (2D) honeycomb lattices, illustrating the potential of the coupling scheme for higher-dimensional Floquet engineering (Fig. 1d). Owing to its geometric nature, the platform is in principle extendable across dimensionalities, materials, and spectral ranges.

To place the present work in the context of existing photonic Floquet platforms, we compare it with representative previous approaches in Table 1. Earlier platforms typically realize either direct but sign-limited coupling, or control coupling through the auxiliaries or additional modulation. By contrast, the present platform uses the relative polarization angle to realize direct and continuous longitudinal modulation of the coupling magnitude and sign within a single compact Floquet platform.

**Table 1:** Comparison of the present work with representative photonic Floquet platforms

Floquet photonic platform	Reference	Coupling control				Physical phenomena
		Direct coupling	Controlled physical degree of freedom	Longitudinal modulation of κ(z)	Continuous magnitude-and-sign control of within one Floquet period	
Twisted waveguide array	Our work	√	Relative polarization angle	Continuous	√	Floquet flat bands and period-nΛ oscillations in 1D lattices; chirality reversal of topological states in 2D lattices
Curved waveguide array	^12^	√	Waveguide spacing	Continuous	×	Anomalous π-edge modes
Curved waveguide array	^17^	√	Waveguide bending trajectory	×	×	Artificial gauge field-enabled dispersionless coupling
Helical waveguide array	^16^	√	Waveguide helical trajectory	×	×	Floquet topological one-way edge states
Waveguide array	^23^	√	Waveguide spacing	Stepwise	×	Parity-time-symmetric topological insulator
	^24^	√	Waveguide spacing	Stepwise	×	Bulk/edge solitons
	^25^	√	Waveguide spacing	Stepwise	×	Topological solitons
Microring resonators	^30^	×	Resonator spacing; Propagation constant detuning	Effective stepwise	×	Full k-space Flat bands
Fiber-loop lattice	^31^	×	Electro-Optic modulation	Effective stepwise	×	Non-Abelian lattice gauge fields
Multi-orbital waveguide array	^28^	√	S/P orbital configuration	Stepwise	×	Phase-controlled chiral transport of Floquet states
Related non-Floquet waveguides	^39,40^	√	Relative orientation of higher-order modes	×	N.A. (static)	Flat bands; Aharonov-Bohm caging; topological states

The table compares this work with representative Floquet photonic platforms in terms of direct nearest-neighbour coupling implementation, controlled physical degree of freedom, the longitudinal modulation of κ(z), continuous control of both coupling magnitude and sign within one Floquet period, and representative physical phenomena. Here, κ(z) denotes the coupling coefficient between adjacent lattice sites. For the related non-Floquet studies, continuous sign tuning denotes tuning the coupling coefficient across different static modal orientations, rather than longitudinal modulation of κ(z) within a single structure.

## Results and discussion

### Theoretical model of polarization-controlled coupling in twisted waveguides

**Fig. 2:**
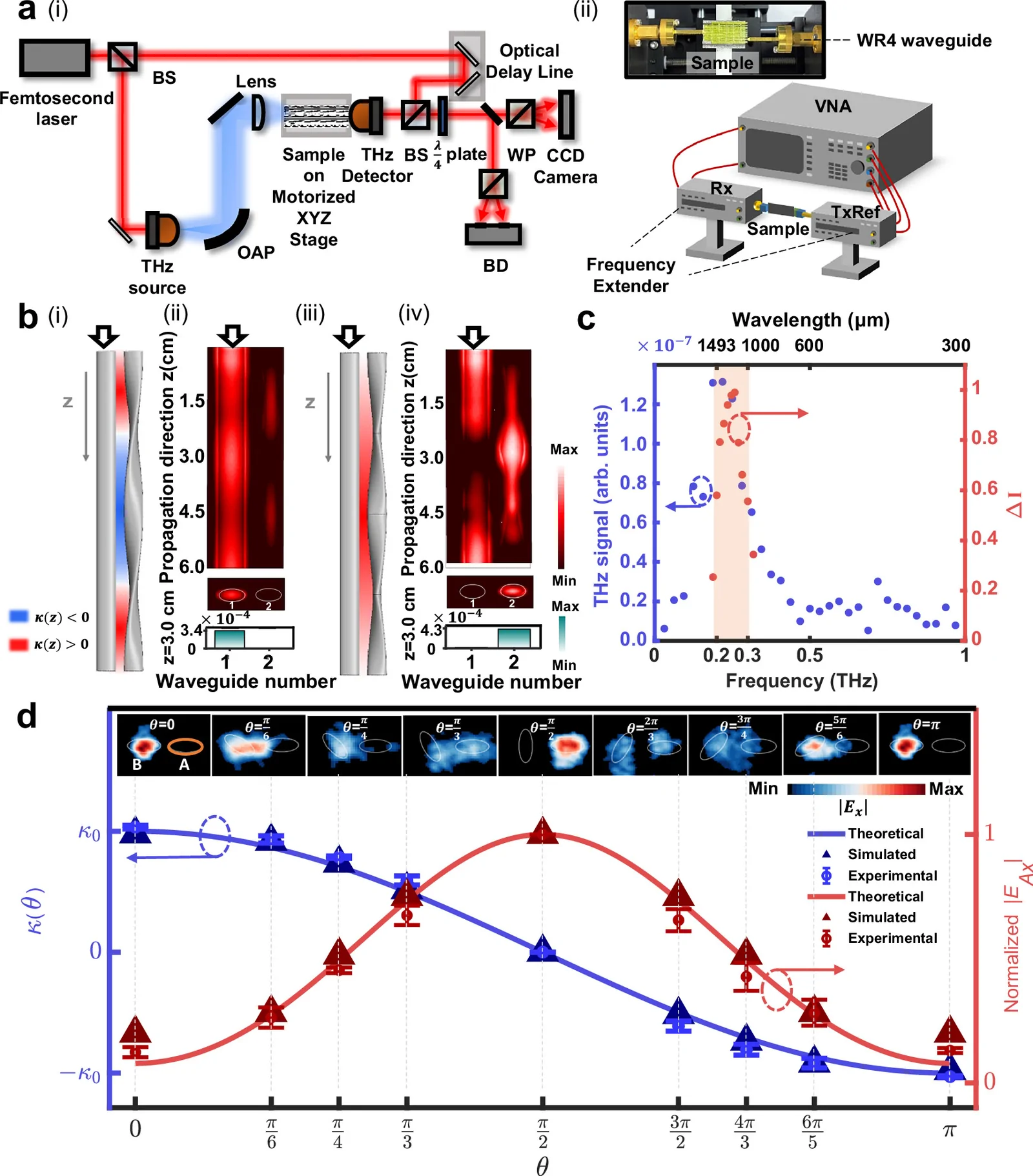
Experimental verification of polarization-controlled coupling in twisted waveguides. **a** Measurement setups. (**i**) THz-TDS setup. OAP, off-axis parabolic mirror; BS, beam splitter; BD, balance detector; WP: Wollaston prism. (**ii**) THz-VNA setup. Inset: photograph of the setup. **b** FDTD simulated and experimental verification of the effect of longitudinal coupling-sign reversal in two coupled waveguides. (**i–ii**), one waveguide is twisted by 2π along the propagation direction, yielding $\kappa \left(z\right)={\kappa }_{0}\cos \theta (z)$. (**iii–iv**), the twisted waveguide undergoes alternating rotations of $\pm \pi /2$, yielding $\kappa \left(z\right)={\kappa }_{0}|\cos \theta \left(z\right)|$. **c** TE mode spectrum of a single waveguide and operational bandwidth determined from the relative output intensity variation $\Delta I\left(\lambda \right)$. Operational bandwidth: 1000 μm to 1493 μm. **d** Experimental, simulated and theoretical results for nine pairs of waveguides with different θ. Top: measured output field distribution (*E*_*Ax*_) after 1 cm of propagation, with normalized intensity plotted as a function of θ. Orange dashed circles highlight the injected waveguides. Bottom: Extracted coupling coefficient $\kappa (\theta )$. Solid lines denote theory; points denote simulations and experiments. Error bars indicate standard deviations. Error bars indicate the standard deviation of 12 repeated measurements.

**Fig. 3:**
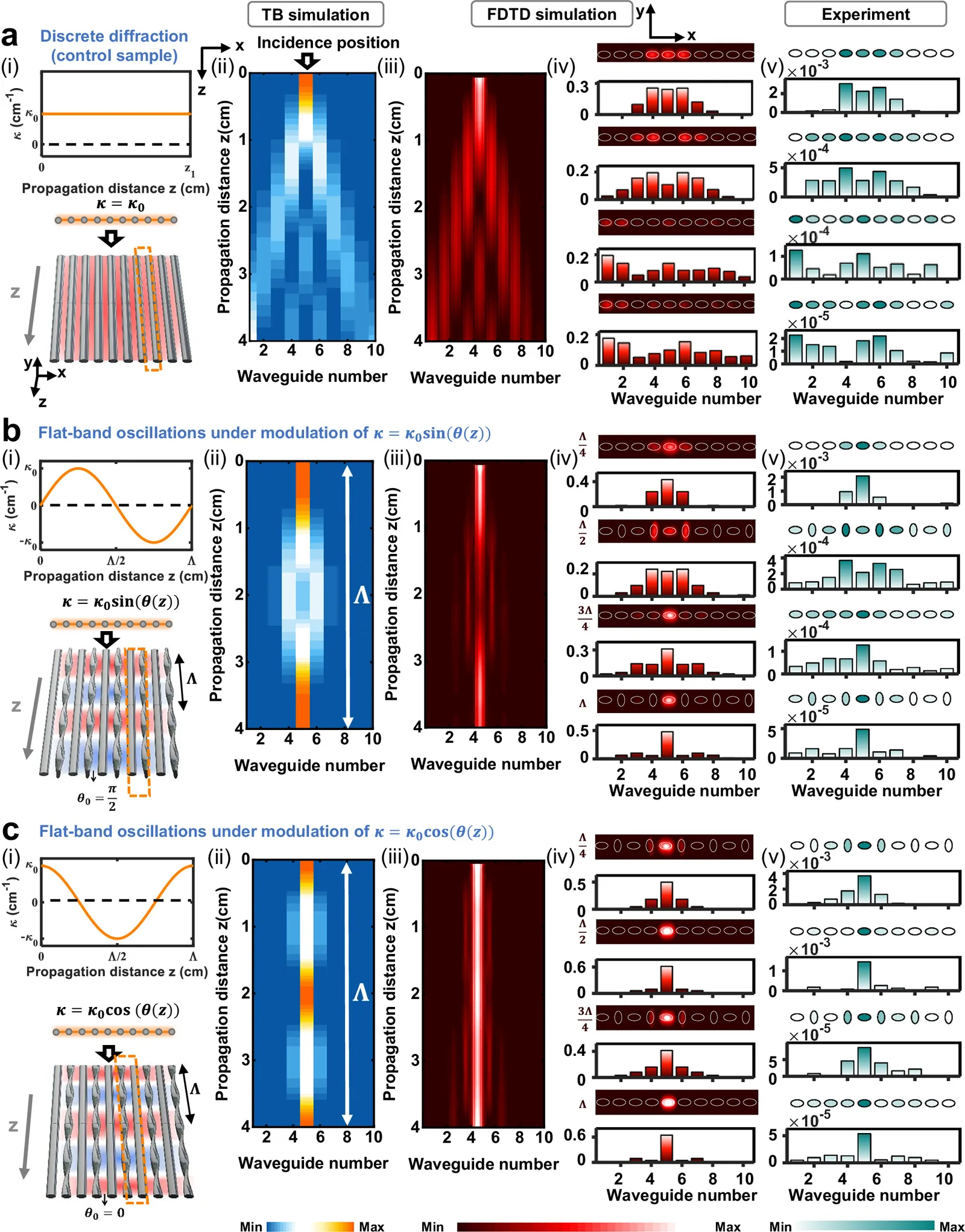
Floquet flat bands enabled by coupling modulation. Ten waveguides spaced by 1100 ${{{\rm{\mu }}}}{{{\rm{m}}}}$ form an array with propagation distance of Λ=4 cm **a** Discrete diffraction in a straight waveguide array with propagation-invariant coupling $\kappa={\kappa }_{0}$ **b** Floquet flat-band dynamics under $\kappa={\kappa }_{0}\sin (\theta \left(z\right))$. **c** Floquet flat-band dynamics under $\kappa={\kappa }_{0}\cos (\theta \left(z\right))$. In the left column, the orange dashed box highlights the coupling corresponding to the function plotted above in panel (**i**). For each case, panel (**i**) shows the coupling modulation and waveguide geometry; (**ii**) and (**iii**) show TB and FDTD simulations; and (**iv**) and (**v**) show FDTD and experimental output intensity distributions at selected propagation distances $\frac{\Lambda }{4}$, $\frac{\Lambda }{2}$, $\frac{3\Lambda }{4}$, and Λ. Light is injected into the central waveguide. The FDTD results use a common normalization scale, whereas the experimental bar plots show directly measured, unnormalized intensities.

**Fig. 4:**
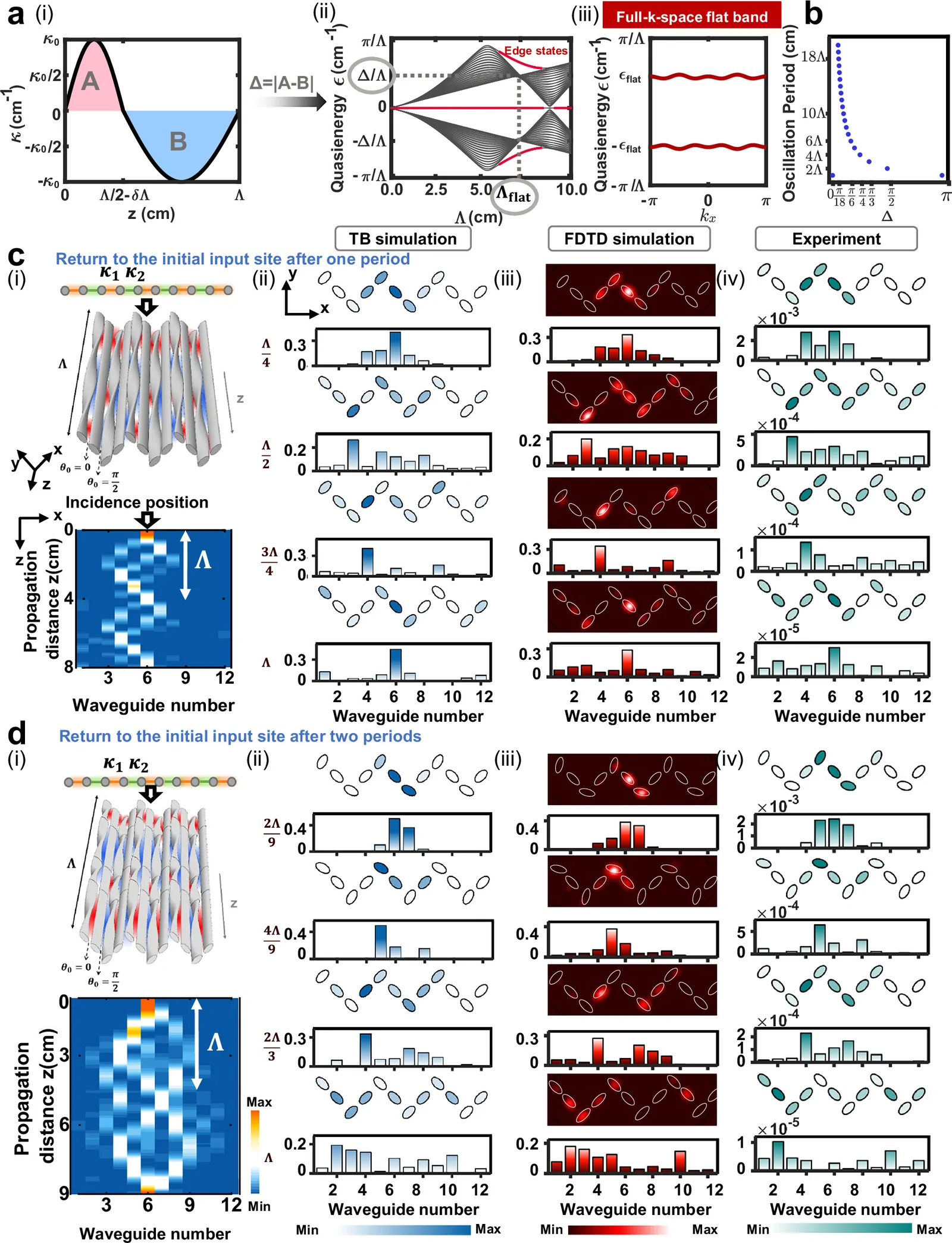
Period- *n*Λ oscillations. **a** Coupling modulation with different twisting rates. (i) Regions A and B correspond to different twisting rates within one driving period. (ii) Quasi-energy spectrum under open boundary conditions, exhibits flat bands at ${\Lambda }_{{flat}}$. (iii) Quasi-energy spectrum under periodic boundary conditions under ${\Lambda }_{{flat}}$ shows flat bands with the value of $\Delta /\Lambda$. **b** Oscillation period as a function of Δ. **c**, **d** Experimental results of period-nΛ oscillations for n=1 and n=2, respectively. (**i**) Schematics of the waveguide arrays and the corresponding tight-binding propagation dynamics, respectively. Transverse intensity distributions are shown for the tight-binding simulations in (**ii**), the FDTD simulations in (**iii**) and the experiments in (**iv**). The FDTD results are normalized to a common scale across different propagation distances, whereas the experimental bar plots show directly measured, unnormalized output intensities.

In our periodically modulated waveguide arrays, the propagation direction is effectively mapped to the time dimension, allowing adiabatic evolution of the eigenpolarization modes during propagation. This feature enables polarization-controlled coupling through periodic modulation. The waveguides, designed with elliptical cross-sections to lift TE-TM degeneracy, are fabricated via high-precision 3D printing with RG resin. Experiments are performed using two complementary terahertz measurement platforms (Fig. 2a). The coupling coefficient as a function of the relative polarization angle is characterized by terahertz time-domain spectroscopy (THz-TDS) combined with two-dimensional terahertz real-time imaging (Fig. 2a(i)). For the THz-TDS system, a femtosecond laser pumps a Terahertz source, and the transmitted signal through the sample is collected by a balanced detector. The sample is mounted on a motorized XYZ stage for spatial movement. The measurements of the propagation dynamics associated with the Floquet flat band and period-nΛ oscillations in Figs. 3 and 4 are carried out on a terahertz vector network analyzer (THz-VNA) platform with frequency extension modules (Fig. 2a(ii)), in which the sample is placed between the transmitting and receiving modules. The TxRef module serves as the transmitting and reference port for terahertz signal generation and coherent detection, whereas the Rx module receives the transmitted terahertz signal. Two WR4 metallic waveguides are connected to the sample to couple the terahertz signal into the structure and collect the transmitted output. Benefiting from swept-frequency coherent detection, the THz-VNA provides a lower noise floor and a higher dynamic range, thereby producing clearer output intensity distributions for the weak transmitted signals involved in these experiments. Throughout this work, the TE mode, aligned with the major axis of the ellipse, serves as the input excitation. According to coupled-mode theory^41^, the coupling coefficient is proportional to the overlap integral of the transverse electric fields of the two modes (Supplementary Note 1), then we derive:

1 $$\kappa={\kappa }_{0}\cos \theta,$$

where ${\kappa }_{0}$ denotes the coupling coefficient when the TE modes in the two waveguides are identically oriented, and θ represents the relative polarization angle between them (Fig. 1a). For two parallel elliptical waveguides, one straight and the other continuously twisted by 2π along the propagation direction (Fig. 2b(i)), the coupling coefficient is given by $\kappa \left(z\right)={\kappa }_{0}\cos \theta (z)$, where $\theta (z)=\frac{2\pi z}{\Lambda }$ denotes the relative polarization angle of their TE modes and Λ is the driving period. At $z=\frac{\Lambda }{4}$ and $\frac{3\Lambda }{4}$, the two polarization directions are orthogonal, such that κ(z) passes through zero and changes sign. To demonstrate the physical effect of this sign reversal, we fabricated another configuration in which the twisted waveguide undergoes successive $\frac{\pi }{2}$ clockwise and counterclockwise twists (Fig. 2b(iii)). This configuration gives $\kappa \left(z\right)={\kappa }_{0}\left|\cos \theta \left(z\right)\right|$, thereby removing the sign reversal while preserving the same coupling magnitude ∣κ(z)∣ at every propagation position. The upper parts of Fig. 2b(ii) and 2b(iv) show the corresponding finite-difference time-domain (FDTD) simulated intensity evolution. The intensity distributions at $z=3.0\,{{{\rm{cm}}}}$ are shown below the propagation maps, with the simulated results placed above the corresponding experimental measurements. The distinctly different light propagation dynamics observed in both the simulations and experiments provide experimental support for the polarization-controlled coupling mechanism. The quantitative comparison yields distribution fidelities F of 0.9994 and 0.9997 for panels (ii) and (iv), respectively, while the corresponding normalized squared Euclidean distances ${D}_{{NSE}}$ are 0.00008 and 0.00006. These values indicate that the relative intensity distribution between the two output waveguides is consistently reproduced in the FDTD simulations and experiments. For the physical phenomenon discussed below, the sign reversal of the coupling coefficient plays the primary role. Further details are presented in Supplementary Note 1 and Supplementary Fig. 1. To experimentally verify the relationship between the coupling coefficient and the relative polarization angle, nine groups of resin waveguides pairs, each 1 cm in length, were fabricated with different relative polarization angles. In Fig. 2c, the blue dots represent the measured THz-TDS output intensity from the waveguide structure, indicating the operational frequency range of our experiment. The red dots show the twist-angle-induced intensity variation ΔI as a function of frequency, demonstrating the bandwidth of the structure. The blue and red dashed circles point to the corresponding left and right vertical axes, respectively. The resin based waveguide pairs were designed with relative polarization angles of 0, $\frac{\pi }{6}$, $\frac{\pi }{4}$, $\frac{\pi }{3}$, $\frac{\pi }{2}$, $\frac{2\pi }{3}$, $\frac{3\pi }{4}$, $\frac{5\pi }{6}$, and π, separately. The TE mode and its frequency response were characterized by THz-TDS. The output intensity variation of the injected waveguide, $\Delta I\left(\lambda \right)=\frac{{I}_{\max }\left(\lambda \right)-{I}_{\min }\left(\lambda \right)}{{I}_{\max }\left(\lambda \right)}$, quantifies the coupling modulation. A wavelength is within the operational bandwidth if ${\rm\Delta }I\left(\lambda \right) >$50% (Supplementary Note 2), spanning a broad range of the supported TE mode. The measured x-polarized output fields after 1 cm of propagation reproduce the overall dependence on the relative polarization angle predicted by theory and simulation (Fig. 2d). In the theoretical model, only the coupling between TE modes polarized along the major axis of the elliptical waveguides is considered. In the practical simulation and experimental systems, however, although the TE and TM modes are lifted from degeneracy, weak residual intermodal coupling may still occur. Together with fabrication imperfections and slight deviations in waveguide geometry, this can lead to a small but non-zero signal near θ=0 and $\theta=\pi$, where the idealized theory predicts vanishing transmission. In the top panel of Fig. 2d, the input light is launched into waveguide A (indicated by the orange circle), and the output intensities are measured for both waveguides A and B. At θ=0 and $\theta=\pi$, the optical field is exactly at the first complete coupling from A to B, resulting in the output field being entirely localized in waveguide B in the first and last panels.

Based on the polarization-controlled coupling mechanism, the coupling coefficient can vary from its maximum to minimum value without increasing the structural spacing, thereby enabling a highly compact platform. We then construct twisted waveguide arrays and analyze light propagation within the tight-binding model, considering only nearest-neighbor interactions. The effective propagation constant of a single waveguide can be treated as a constant. For simplicity, this constant offset term can be reasonably neglected in the theoretical analysis.

### Floquet flat bands and period‑n${{{\boldsymbol{\Lambda }}}}$ oscillations

Owing to the ability to tune the coupling coefficient across both positive and negative regimes, flat bands can be realized through a simple and compact design. We propose a Floquet lattice and demonstrate that when all nearest-neighbor couplings are uniform, with $\kappa (z)={\kappa }_{0}\cos (\theta (z)+{\theta }_{0})$, the system exhibits Floquet flat bands spanning the entire Brillouin zone. In addition, when the coupling coefficients between adjacent waveguides alternate as ${\kappa }_{1}(z)={\kappa }_{0}\cos (\theta (z)+{\theta }_{1})$ and ${\kappa }_{2}(z)={\kappa }_{0}\cos (\theta (z)+{\theta }_{2})$, corresponding to a two-site lattice, the system continues to support Floquet flat bands at specific driving period ${\Lambda }_{{flat}}$, and further enables period‑nΛ oscillations by tuning the twisting rates.

We first consider the one-site lattice. In the control sample, all waveguides are straight (Fig. 3a(i)). Under twisting, the coupling coefficients vary as ${\kappa }_{0}\cos (\theta (z)+{\theta }_{0})$ with ${\theta }_{0}=\frac{\pi }{2}$ (Fig. 3b(i)) and ${\theta }_{0}=0$ (Fig. 3c(i)). The system maps onto a 1D tight-binding lattice with Hamiltonian:

2 $$H (k,z)=2{\kappa}_{0} \cos (\theta (z)+{\theta }_{0}) \cos (k),$$

The Floquet operator $U\left(k,\Lambda \right)=\hat{T}{e}^{-i \int_{0}^{\Lambda }H(k,z)\,{dz}}$, where $\hat{T}$ is the time-ordering operator and Λ is the Floquet driving period, defines the effective Hamiltonian ${H}_{F}=\frac{i}{\Lambda }{{\mathrm{ln}}}U(k,\Lambda )$^42^ and the eigenvalues of ${H}_{F}$ correspond to the system’s quasi-energy spectrum. For this one-site lattice, ${\int_{0}^{\Lambda }H(k,z) \, {dz}=0}$, leading to a zero-quasi-energy flat band across the entire momentum space and vanishing group velocity. Consequently, the optical field reproduces its initial distribution after one driving period.

Tight-binding (TB) simulations, finite-difference time-domain (FDTD) intensity evolutions, and the corresponding output intensity distributions at selected propagation distances are presented in Fig. 3a(ii–iv)–3c(ii–iv), with the experimentally measured output distributions shown in Fig. 3a(v)–3c(v). Here, Λ=4 cm corresponds to one Floquet driving period. Owing to the negligible loss in the simulations, all FDTD results are displayed using a common normalization scale, whereas the experimental bar plots show directly measured, unnormalized intensities; a detailed loss analysis is provided in Supplementary Note 10 and Figs. S12 and S13. Under twisting modulation, the light no longer diffracts into the bulk as in straight arrays (Fig. 3a), but reproduces the incident field distribution after Λ, exhibiting a Floquet self-imaging effect. This behavior depends on where the coupling sign reverses along the propagation. For ${\theta }_{0}=\frac{\pi }{2}$, the sign inversion occurs at $\frac{\Lambda }{2}$, leading to spreading over the first half-period and refocusing over the second half (Fig. 3b). For ${\theta }_{0}=0$, inversions at $\frac{\Lambda }{4}$ and $\frac{3\Lambda }{4}$ result in two successive periods of spreading and refocusing within one driving period (Fig. 3c). These observations reproduce the contrast between diffraction and Floquet refocusing. The quantitative comparison yields mean distribution fidelities $\bar{F}$ above 0.935, with the corresponding mean $\bar{{D}_{{NSE}}}$ values below 0.081, as detailed in Supplementary Table 2. The measured distributions show slight deviations in the relative site intensities, particularly at intermediate and long propagation distances. The simulated intensity distribution is sensitive to the refractive index assumed for the resin, because a small offset in this parameter changes the inter-waveguide coupling strength. The resulting small coupling mismatch can accumulate over the propagation distance and redistribute the site-resolved output intensity. We therefore independently calibrated a single global refractive-index correction using the straight waveguide control array, as detailed in Supplementary Fig. 28. Additional differences may arise from output alignment and the idealized input mode used in the FDTD model. These effects can contribute to the remaining site-resolved differences. The Floquet lattice thus enables self-imaging governed by the relative polarization angle ${\theta }_{0}$, providing an additional degree of control over light propagation, without requiring specific input positions or large arrays as in conventional Bloch oscillations^18,43^.

We next consider the two-site lattice with ${\theta }_{1}=0$ and ${\theta }_{2}=\frac{\pi }{2}$ (Fig. 4). Flat bands persist for a specific driving period ${\Lambda }_{{flat}}$, where the twisting rate in the first half of the driving period is faster than that in the second half. In Fig. 4a(i), the coupling coefficient κ(z) over one driving period Λ is shown. The integrated areas under the curve for the first and second halves of the period are denoted as A and B, respectively, and their difference $\Delta={{{\rm{|A}}}}-{{{\rm{B|}}}}$ (dimensionless) determines the quasi-energy ${\varepsilon }_{{flat}}=\Delta /\Lambda$ of the flat band, as plotted in Fig. 4a(ii) and (iii). Therefore, the corresponding quasi-energy values at ${\Lambda }_{{flat}}$ are no longer zero, which depend on the rate difference. Figure 4a(ii) and 4a(iii) show the quasi-energy bands calculated for an array of eighty waveguides under open and periodic boundary conditions (OBCs and PBCs), respectively. The emergence of flat bands at ${\Lambda }_{{flat}}$ can be understood within the framework of the Magnus expansion^44^. The time evolution operator is expressed as $U\left(z\right)={e}^{-i\Omega (z)}$, where Ω(z) is expanded as $\Omega \left(z\right)={\Omega }_{1}\left(z\right)+{\Omega }_{2}\left(z\right)+\ldots$, with ${\Omega }_{1}\left(z\right)={\int }_{0}^{z}H\left({z}_{1}\right)d{z}_{1}$, ${\Omega }_{2}\left(z\right)=\frac{i}{2}{\int }_{0}^{z}{\int }_{0}^{{z}_{1}}\left[H\left({z}_{1}\right),H\left({z}_{2}\right)\right]d{z}_{2}d{z}_{1}$, and so on. Since $[H\left(z\right),H({z}^{{\prime} })]\ne 0$, the higher-order terms in the Magnus expansion generally contribute nonzero values, resulting in a nonzero quasi-energy spectrum for most driving periods. However, at the specific driving period, denoted as ${\Lambda }_{{flat}}$, all higher-order terms beyond ${\Omega }_{1}\left(z\right)$ sum exactly to zero. Consequently, the quasi-energies are determined entirely by the first-order Magnus term ${\Omega }_{1}\left(z\right)$. For Δ=0, ${\Omega }_{1}\left(z\right)=0$, yielding zero-quasi-energy flat bands across the entire Brillouin zone. For $\Delta \ne 0$, ${\Omega }_{1}\left(z\right)$ becomes nonzero, but its eigenvalues remain momentum independent. The bands therefore remain flat, with their quasi-energy values determined by Δ.

The intra-cell coupling integrates to zero over one driving period, while the inter-cell coupling yields a finite integrated value, whose absolute value is Δ, and ${{{{\rm{\epsilon }}}}}_{{flat}}=\Delta /{\Lambda }_{{flat}}$ gives the flat band quasi-energy across the entire Brillouin zone. By controlling ${{{{\rm{\epsilon }}}}}_{{flat}}$ through the twisting rates, period-nΛ oscillations ($n\in {N}^{+}$) can be realized. Specifically, when the integrated value satisfies $\Delta=\frac{\pi }{n}$, the flat-band quasi-energy becomes ${\varepsilon }_{{flat}}=\pm \frac{\pi }{n{\Lambda }_{{flat}}}$. In this case, the Floquet operator takes the form $U\left({\Lambda }_{{flat}}\right)={P}_{+}{e}^{-i\frac{\pi }{n}}+{P}_{-}{e}^{i\frac{\pi }{n}}$, where ${P}_{\pm }$ are the projectors onto the $\pm \frac{\pi }{n}$ subspaces. After n driving periods, the state evolves as:

3 $$|\psi (n{\Lambda }_{flat})\rangle={({e}^{-i\frac{\pi }{n}})}^{n}|{\psi }_{+}\rangle+{({e}^{i\frac{\pi }{n}})}^{n}|{\psi }_{-} \rangle=-|{\psi }_{+}\rangle -|{\psi }_{-} \rangle=-|{\psi }_{0}\rangle,$$

indicating that the optical field distribution reappears, though with a global phase shift. Thus, the recurrence of field intensities requires ${\Lambda }_{o}=n{\Lambda }_{{flat}}$, while the complete revival of both amplitude and phase requires ${\Lambda }_{o}=2n{\Lambda }_{{flat}}$. The relationship between ${\Lambda }_{o}$ and Δ is shown in Fig. 4b.

When light is injected into the twisted waveguide array with the driving period ${\Lambda }_{{flat}}$, oscillatory dynamics emerges. To minimize coupling mismatch when the two TE modes are either fully parallel or fully orthogonal, a staggered step-like configuration is adopted. In contrast to period-Λ oscillation (detailed in Supplementary Note 3), the array supporting period-2Λ oscillation has a twisting rate that is faster in the first half (1.5 cm, 0 to π) than in the second half ($3{{{\rm{cm}}}}$, π to 2π). Twelve waveguides spaced by 850 ${{{\mu }}}{{{\rm{m}}}}$ form an array over a propagation distance of Λ=4 cm and 4.5 cm, respectively. The corresponding TB simulations are shown in Fig. 4c(ii) and Fig. 4d(ii). The FDTD simulations and experimentally measured output intensity distributions are shown in Fig. 4c (iii, iv) for the period-Λ oscillation at selected propagation distances $\frac{\Lambda }{4}$, $\frac{\Lambda }{2}$, $\frac{3\Lambda }{4}$, and Λ, and in Fig. 4d (iii, iv) for the period-2Λ oscillation at propagation distances $\frac{2\Lambda }{9}$, $\frac{4\Lambda }{9}$, $\frac{2\Lambda }{3}$, and Λ, respectively. In all cases, light is injected into the central waveguide. In the array exhibiting period-Λ, the optical field recovers its original field distribution after one driving period, whereas in the array supporting period-2Λ, full revival occurs only after two periods. For the period-2Λ sample, the measurements extend only to z=Λ because of the intrinsic material loss. The TB, FDTD, and experimental results show the same overall propagation behavior, while the remaining site-resolved differences may partly arise from the sensitivity to the effective excitation position and possible residual TE-TM coupling at the smaller waveguide separations (Supplementary Fig. 35 and Note 21). The period-nΛ oscillations, whose period ${\Lambda }_{o}$ arises from the integral of positive and negative coupling coefficients via polarization-controlled Floquet engineering, enable a self-imaging function with customized device design and adaptable period control, and may find applications in measurement-based quantum information processing^45^.

### Topological phase transitions and chirality reversal of edge states

**Fig. 5:**
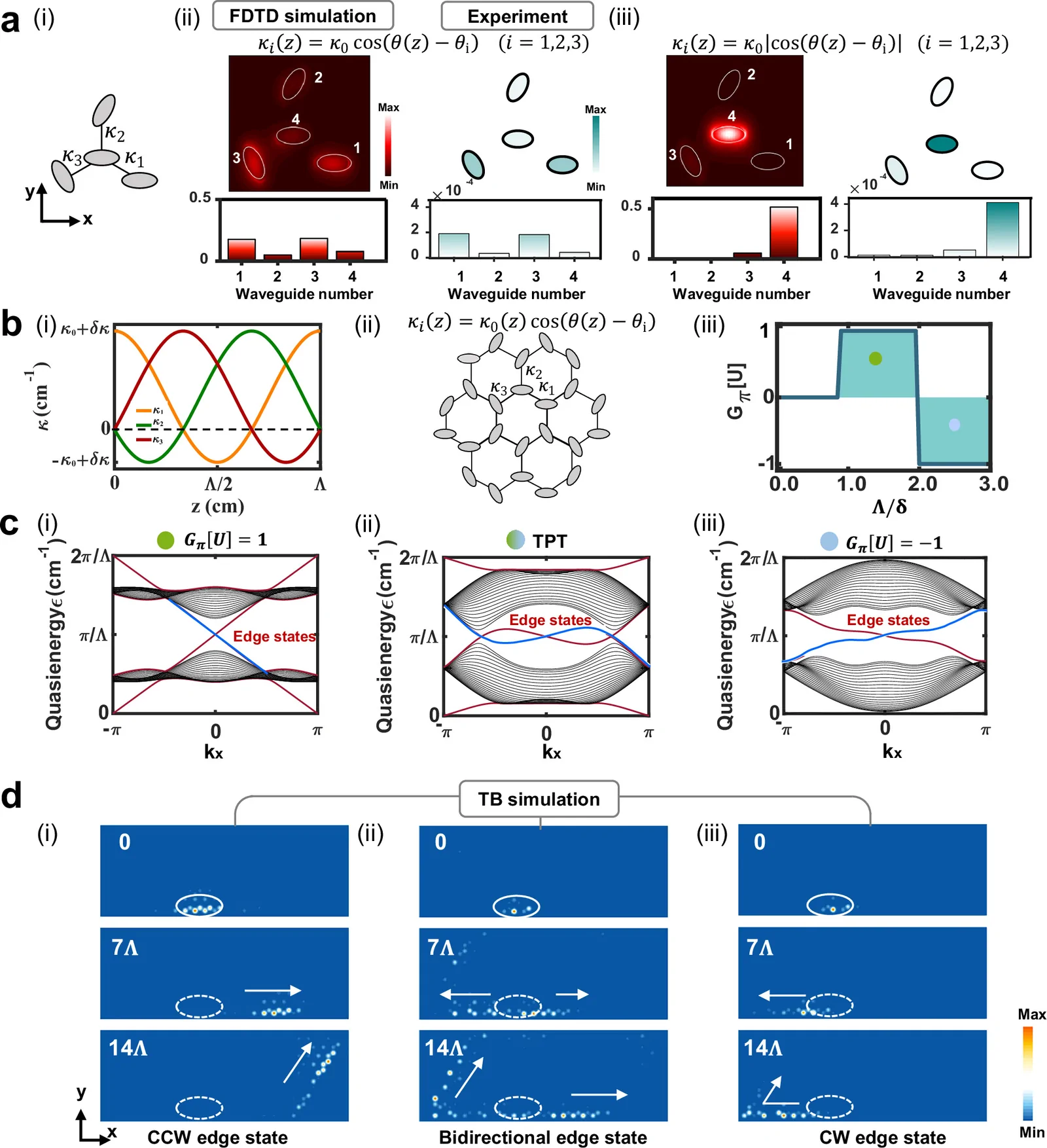
Multidirectional coupling and chirality reversal in two-dimensional Floquet photonic lattices. **a** FDTD-simulated and experimental verification in a four-site, three-arm unit. (i) Cross-sectional schematic of the unit, in which a central waveguide is coupled to three neighboring waveguides. (ii) FDTD-simulated and experimentally measured intensity distributions for the couplings ${\kappa }_{1}(z)={\kappa }_{0}\cos (\theta \left(z\right))$, ${\kappa }_{2}(z)={\kappa }_{0}\cos (\theta \left(z\right)-2\pi /3)$, and ${\kappa }_{3}(z)={\kappa }_{0}\cos (\theta \left(z\right)-4\pi /3)$. (iii) Corresponding results for the couplings ${\kappa }_{1}(z)={\kappa }_{0}\left|\cos (\theta \left(z\right))\right|$, ${\kappa }_{2}(z)={\kappa }_{0}\left|\cos (\theta \left(z\right)-2\pi /3)\right|$, and ${\kappa }_{3}(z)={\kappa }_{0}\left|\cos (\theta \left(z\right)-4\pi /3)\right|$. **b** Topological properties of the 2D lattice. (i) Coupling coefficient ${\kappa }_{1}$, ${\kappa }_{2}$ and ${\kappa }_{3}$ as a function of propagation distance z. (ii) Schematic of the cross section. (iii) Phase diagram of the driven system indicating the emergence of topological phases. **c** Quasi-energy spectra for different types of edge states in 2D lattices under open boundary conditions projected along the zigzag edge. (i) CCW propagating edge state under one topological phase. (ii) Bidirectional edge states at the topological phase transition (TPT) point. (iii) Clockwise (CW) propagating edge state. Red and blue curves indicate topologically protected edge states. **d** TB simulated propagation of 2D Floquet edge states. (i) Excitation at the lower edge shows robust CCW edge transport. (ii) At the TPT point, bidirectional edge states emerge. (iii) Edge transport direction reverses to CW. White dashed circles highlight the injected regions.

Extending the coupling coefficient from positive to the entire real regime enables the exploration of a broad range of Floquet topological phenomena. Before considering the full 2D lattice, we first consider a four-site, three-arm unit in which a central waveguide is coupled to three neighboring waveguides through couplings with relative phase shifts. To demonstrate the effect of longitudinal coupling-sign reversal, we compare the signed couplings, described by ${\kappa }_{1}(z)={\kappa }_{0}\cos (\theta \left(z\right))$, ${\kappa }_{2}(z)={\kappa }_{0}\cos (\theta \left(z\right)-2\pi /3)$, and ${\kappa }_{3}(z)={\kappa }_{0}\cos (\theta \left(z\right)-4\pi /3)$, with the corresponding unsigned couplings, described by their absolute values ${\kappa }_{1}(z)={\kappa }_{0}\left|\cos (\theta \left(z\right))\right|$, ${\kappa }_{2}(z)={\kappa }_{0}\left|\cos (\theta \left(z\right)-2\pi /3)\right|$, and ${\kappa }_{3}(z)={\kappa }_{0}\left|\cos (\theta \left(z\right)-4\pi /3)\right|$. The FDTD simulations predict distinct output intensity distributions for the two cases, and the experiment reproduces the main contrast in the relative intensities across the output sites, as shown in Fig. 5a. The quantitative comparison yields distribution fidelities F of 0.9868 and 0.9928 for panels (ii) and (iii), respectively, while the corresponding normalized squared Euclidean distances ${D}_{{NSE}}$ are 0.00288 and 0.00058. These values indicate close agreement between the FDTD simulations and the experimental measurements. We then theoretically investigate the chirality reversal of Floquet edge states produced by varying the driving period in a 2D honeycomb lattice. These values support the consistency between the FDTD-simulated and experimentally measured output distributions.

We extend the polarization-controlled Floquet engineering (Fig. 5b(i)) to a 2D photonic lattice. In 2D lattices, as illustrated in Fig. 5b(ii), the coupling strength between waveguides can also be controlled by tuning the polarization direction, thereby enabling the construction of Floquet lattice structures that support anomalous topological phases. In conventional static systems, topological edge states typically reside in the zero-energy gap, whereas in Floquet-driven systems, anomalous edge π modes may arise within the π gap of the quasi-energy spectrum^46^. Specifically, the honeycomb lattice can be implemented by laterally displacing the optical axes of one sublattice from their original positions along the propagation direction. The corresponding waveguide trajectories vary adiabatically, so that no effective magnetic field is induced. A more detailed illustration is provided in Supplementary Fig. 29. The coupling coefficient is therefore expressed as

4 $$\kappa \left(z\right)={\kappa }_{0}\left(z\right)\cos \left(\theta \left(z\right)-{\theta }_{0}\right),$$

where θ(z) is the longitudinally varying polarization angle, ${\theta }_{0}$ is the fixed angular offset associated with a given nearest-neighbor bond, and ${\kappa }_{0}\left(z\right)$ is the distance-dependent coupling amplitude determined by the center-to-center separation between two nearest-neighbor waveguides. The polarization orientations are arranged periodically such that the three inequivalent nearest-neighbor couplings correspond to ${\theta }_{0}=0$, $-\frac{2\pi }{3}$, and $-\frac{4\pi }{3}$, producing the phase-shifted coupling modulations. The asymmetric cross-section is further designed so that neighboring mode-field centroids are closer during the positive-coupling intervals and farther apart during the negative-coupling intervals. Because evanescent coupling decreases with increasing separation, this configuration naturally yields $\left|{\kappa }_{{negative}}\right| < \left|{\kappa }_{{positive}}\right|$ while preserving periodic sign reversal and the chiral coupling sequence of the honeycomb lattice. This introduces chiral phase differences in the distribution of coupling strength, effectively breaking time-reversal symmetry. This structure is physically equivalent to a special case of the Floquet graphene model proposed by Takuya Kitagawa^47^, in which only the static and fundamental frequency components of the coupling strength are retained. However, in the present scheme, the coupling strength is extended over the entire real domain, offering greater flexibility for tuning and realizing topological phases.

To verify the topological phase of the system, we calculate its topological invariant, the winding number. For a two-dimensional Class A lattice system without chiral symmetry, the topological classification falls into the integer group ${\mathbb{Z}}$, in contrast to one-dimensional Class A systems, which cannot host nontrivial topological phases^48^. The winding number can be used to classify mappings from the three-dimensional parameter space ${S}^{1}\times {S}^{1}\times {S}^{1}$ to the unitary group U(N) (i.e., the mapping from the Floquet-Brillouin zone to the space of evolution operators), and is formally defined as follows^49^:

5 $${G}_{\pi }\left[U\right]=\frac{1}{8{\pi }^{2}}\int {dtd}{k}_{x}d{k}_{y}\times {{{\rm{Tr}}}}\left({U}^{-1}{\partial }_{t}U\left[{U}^{-1}{\partial }_{{k}_{x}}U,{U}^{-1}{\partial }_{{k}_{y}}U\right]\right),$$

where U(k,z) is the evolution operator of the periodically driven system over one period, and N denotes the number of mode degrees of freedom within the unit cell, which corresponds to the dimension of the unitary group U(N) to which the evolution operator U(k,z) belongs. The above definition requires that U(k,t) be periodic both in time t and in momentum space $\left({k}_{x},{k}_{y}\right)$. However, this condition is not automatically satisfied in general. Therefore, it is necessary to construct a smooth evolution operator ${U}_{\varepsilon }(k,z)$ that is periodic in the three-dimensional parameter space, defined as follows:

6 $${U}_{\varepsilon }\left(k,z\right)=\left\{\begin{array}{cc}U\left(k,2z\right)& {{{\rm{if}}}}\, 0\le z\le \Lambda /2\\ {V}_{\varepsilon }\left(k,2 \Lambda -2z\right)& {{{\rm{if}}}} \, \Lambda /2 \le z \le \Lambda \end{array}\right.,$$

where ${V}_{\varepsilon }\left(k,t\right)={e}^{-i{H}_{F}\left(k\right)z}$. By appropriately choosing the branch cut of the complex logarithm function, the winding number calculation can be focused on a specific quasi-energy band ε:

7 $$\begin{array}{c}\log {e}^{-i\varepsilon \Lambda+i{0}^{-}}=-i\cdot \varepsilon \Lambda,\\ \log {e}^{-i\varepsilon \Lambda+i{0}^{+}}=-i\cdot \varepsilon \Lambda -2\pi i.\end{array}$$

In particular, we focus on the anomalous energy gap located at $\varepsilon=\frac{\pi }{\Lambda }$.The corresponding winding number is shown in Fig. 5b(iii), where δ, the reciprocal of the static component $\frac{{\kappa }_{0}}{2}\cos {\theta }_{0}$, is taken as the energy scale. As the driving period increases, the topological invariant undergoes a sequence of transitions, from 0 to 1, and subsequently from 1 to −1. Correspondingly, the edge-state propagation reverses its direction from counterclockwise (CCW) to clockwise (CW) before and after the second phase transition. In the present driving protocol, the chirality-reversed topological phase appears only when the positive and negative coupling strengths have different magnitudes. It is absent under the equal-magnitude condition within the parameter range considered here, showing that access to positive and negative couplings of unequal magnitude is essential for realizing this transition.

For the 2D honeycomb lattice, the bulk band structure initially touches at the K and K′ points and gradually shifts toward the center of the Brillouin zone, ultimately altering the chirality of the edge states. Figure 5c(i)-(iii) present the quasi-energy spectrum under OBCs projected along the zigzag edge at three representative driving periods situated in the topological phase with ${G}_{\pi }\left[U\right]=1$, at the topological transition point, and in the topological phase with ${G}_{\pi }\left[U\right]=-1$. The two edge-state branches, highlighted in red and blue in the figure, correspond to modes localized at the upper and lower boundaries of the lattice, respectively. The slope of these bands represents the group velocity of the edge states and evolves continuously with the driving period. The opposite slopes at the two boundaries ensure that all edge modes propagate unidirectionally—either CW or CCW—depending on the sign of ${G}_{\pi }\left[U\right]$. TB simulations of light propagation reveal the distribution of the optical field at the output facet after different propagation distances, corresponding to a CCW edge state, bidirectional edge transport near the phase transition, and CW edge state, respectively, as shown in Fig. 5d(i)-(iii). Experimental realization in the present platform is challenging due to the large propagation length required (on the order of tens of centimeters) and the relatively high absorption of the 3D-printed resin at terahertz frequencies. The chirality reversal of edge states in a 2D lattice highlights the potential of the platform for higher-dimensional Floquet engineering.

## Discussion

In summary, we have established a Floquet photonic platform based on polarization-controlled coupling, which enables continuous modulation of the coupling coefficient across both positive and negative values. We report Floquet flat bands spanning the entire Brillouin zone with robust self-imaging, controlled period-nΛ oscillations, and chirality reversal. This work provides a route for exploring a large range of physical phenomena in optical field engineering, topological photonics, and quantum information processing that remain inaccessible in conventional systems. We acknowledge that the relatively high absorption loss of the resin platform used in the present experiments limits the observation of phenomena over substantially longer propagation distances. Although the present experiments are implemented in 3D-printed resin waveguides, the concept may be extended to lower-loss platforms through several complementary approaches, such as porous waveguides with reduced modal overlap with the absorbing material and 3D-printed high-purity alumina ceramic waveguides. Further optimization of material processing and waveguide geometry may improve the performance of these implementations. With continued advances in femtosecond-laser-writing techniques, the concept may also be transferable to waveguide platforms operating in the visible and near-infrared regimes. More broadly, polarization-controlled coupling may also be adapted to other photonic platforms beyond waveguide arrays, such as micro ring resonator lattices, modulated fiber-based systems, and other architectures for optical computing (see Supplementary Note 20). These extensions could further expand the design space of integrated photonics and open directions for programmable photonic circuits and quantum simulators.

## Methods

### Numerical simulation

Finite-difference time-domain (FDTD) simulations in 3D were performed with the structure embedded in an air background. The refractive index of the resin material was set to n=1.7, and the injected wavelength was around $\lambda=1200\,{{{\mu }}}{{{\rm{m}}}}$. The elliptical cross-section of each waveguide was defined by a semi-major axis of $a=350\,{{{\mu }}}{{{\rm{m}}}}$ and a semi-minor axis of $b=150\,{{{\mu }}}{{{\rm{m}}}}$. Depending on the physical phenomenon discussed in the main text, the inter-waveguide spacing was adjusted to match the required driving period. We also perform simulations for metallic hollow waveguide. The metallic cladding was modeled as a perfect electric conductor, while the hollow region was defined as an etched air-filled geometry. In this case, the injected wavelength is around $\lambda=600\,{{{\mu }}}{{{\rm{m}}}}$, with the elliptical cross-section specified by a semi-major axis of $a=450\,{{{\mu }}}{{{\rm{m}}}}$ and a semi-minor axis of $b=250\,{{{\mu }}}{{{\rm{m}}}}$.

### Quantitative comparison of simulated and experimental intensity distributions

To quantify the agreement between the experimentally measured and FDTD-simulated output distributions in Figs. 3 and 4, the site-resolved intensities at each sampled propagation distance z are normalized to unit total intensity:

8 $${p}_{n,m}^{{FDTD}}=\frac{{I}_{n,m}^{{FDTD}}}{{\sum }_{j=1}^{N}{I}_{j,m}^{{FDTD}}},\,{p}_{n,m}^{\exp }=\frac{{I}_{n,m}^{\exp }}{{\sum }_{j=1}^{N}{I}_{j,m}^{\exp }},$$

where N is the number of waveguides. We evaluated the normalized squared Euclidean distance^50^

9 $${D}_{{NSE}}\left({z}_{m}\right)=\frac{1}{2}\frac{{\sum }_{n=1}^{N}{\left[\left({p}_{n,m}^{{FDTD}}-{\bar{p}}_{m}^{{FDTD}}\right)-\left({p}_{n,m}^{\exp }-{\bar{p}}_{m}^{\exp }\right)\right]}^{2}}{{\sum }_{n=1}^{N}{\left({p}_{n,m}^{{FDTD}}-{\bar{p}}_{m}^{{FDTD}}\right)}^{2}+{\sum }_{n=1}^{N}{\left({p}_{n,m}^{\exp }-{\bar{p}}_{m}^{\exp }\right)}^{2}}.$$

The metric satisfies $0\le {D}_{{NSE}}\le 1$, with smaller values indicating closer agreement. As a complementary measure of spatial overlap, we calculated the classical distribution fidelity

10 $${F}_{m}={\left({\sum}_{n=1}^{N}\sqrt{{p}_{n,m}^{{FDTD}}{p}_{n,m}^{\exp }}\right)}^{2},$$

where $0\le {F}_{m}\le 1$, and values closer to unity indicate greater overlap. For M sampled propagation distances, the mean fidelity was calculated as

11 $$\bar{F}=\frac{1}{M}{\sum}_{m=1}^{M}{F}_{m}.$$

These metrics quantify the relative site-resolved intensity distributions after normalization to unit total intensity and do not assess the absolute transmitted power or propagation loss.

### Experimental setup

#### Terahertz time-domain spectroscopy system

Terahertz experiments were conducted using terahertz time-domain spectroscopy combined with 2D terahertz imaging. A Ti:sapphire regenerative amplifier (800 nm central wavelength, 100 fs pulse duration, 1 kHz repetition rate, 900 mW average power) served as the light source. Terahertz pulses were generated via optical rectification in a 1 mm-thick <110 > -oriented ZnTe crystal and detected by free-space electro-optic sampling using a ZnTe sensing crystal. Balanced detection was implemented with a 4 f optical system, a polarizing beam splitter, and a CCD camera. Further details are provided in Supplementary Note 10.

#### Terahertz vector network analyzer system

Additional terahertz measurements are performed using a terahertz vector network analyzer system with frequency-extension modules. In this system, the terahertz signal was generated and detected through swept continuous-wave excitation and coherent detection. The received signal was mixed with a phase-locked reference at the same frequency, allowing the system to selectively extract the signal component coherent with the excitation while suppressing incoherent noise. The narrow intermediate-frequency bandwidth of the VNA receiver further improved the signal-to-noise ratio and dynamic range. Under our measurement conditions, signals down to approximately −100 dB could still be reliably resolved, leading to a lower noise floor and clearer measured output-field distributions. Further details are provided in Supplementary Note 11.

### Sample fabrication

Samples were fabricated using Projection Micro Stereolithography (PμSL), a UV-based 3D printing technique capable of achieving~2 μm resolution. The printing process was based on layer-by-layer photopolymerization of a photosensitive resin using a Digital Micromirror Device (DMD) for spatial light modulation. A commercial resin (RG) was employed, characterized by a hardness of 77 Shore D, elastic modulus of 1765 MPa, and viscosity of 1100 cp at 25 °C, ensuring good mechanical rigidity. The resin also exhibits a dielectric constant of 2.94 and a loss tangent of 0.0197 at 10 GHz. Further details are provided in Supplementary Note 12.

## Supplementary information

Supplementary Information for Polarization-controlled Floquet engineering in twisted photonic lattices

Transparent Peer Review file

## Data Availability

All data are available in the main text or Supplementary Information.
