# Supplementary material for: Polarization-controlled Floquet engineering in twisted photonic lattices
Source: Nat Commun. 2026 Oct 15;17:11655. doi: 10.1038/s41467-026-78660-0 (PMC13760052; doi:10.1038/s41467-026-78660-0)
Supplement: Supplementary file 1 [file 41467_2026_78660_MOESM1_ESM.pdf]

Supplementary Information for

Polarization-controlled Floquet engineering in twisted photonic lattices

Jinze Gao[1]†, Guibin Li[2]†, Zhuochen Du[1]†, Kun Liao[1]†*, Kunyu Yu[1], C. T. Chan[3*], Ruo-Yang Zhang[4*], Daopeng Yuan[5,6], Yan Zhang[2*], Xiaoyong Hu[1,7,8,9*], Qihuang Gong[1,7,8,9]

[1]*State Key Laboratory for Mesoscopic Physics & Department of Physics, Collaborative Innovation Center of Quantum Matter & Frontiers Science Center for Nano-optoelectronics, Peking University, Beijing 100871, P. R. China*

[2]*Department of Physics, Beijing Key Lab for Metamaterials and Devices, Key Laboratory for Terahertz Optoelectronics, Ministry of Education, Beijing Advanced Innovation Center for Imaging Theory and Technology, Capital Normal University, Beijing, 100048, P. R. China*

[3]*Department of Physics, The Hong Kong University of Science and Technology, Clear Water Bay, Kowloon, Hong Kong, P. R. China*

[4]*National Laboratory of Solid State Microstructures, School of Physics, Nanjing University, Nanjing, 210093, P. R. China*

[5]*Taishan College, Shandong University, Jinan, 250100, P. R. China*

[6]*Present address: Department of Chemistry, Massachusetts Institute of Technology, 77 Massachusetts Avenue, Cambridge, MA 02139, USA.*

[7]*Key Laboratory for Advanced Optoelectronic Integrated Chips of Jiangsu Province, Peking University Yangtze Delta Institute of Optoelectronics, Nantong, Jiangsu 226010, P. R. China*

[8]*Collaborative Innovation Center of Extreme Optics, Shanxi University, Taiyuan, Shanxi 030006, P. R. China*

[9]*Hefei National Laboratory, Hefei 230088, P. R. China*

† These authors contributed equally to this work.

*E-mail: kunliao@pku.edu.cn; phchan@ust.hk; yzhang@cnu.edu.cn; zhangruoyang@nju.edu.cn; xiaoyonghu@pku.edu.cn.

Table of Contents

Supplementary Notes:

Supplementary Note 1 - Derivation of the negative coupling coefficient

Supplementary Note 2 - Broadband performance of polarization-controlled coupling

Supplementary Note 3 - Equal twisting rates of the waveguide during the first and second halves of the driving period.

Supplementary Note 4 - Robustness of phenomena under non-sinusoidal coupling (e.g., triangular-wave form).

Supplementary Note 5 - Effective Floquet fermions under extended Floquet Hamiltonian.

Supplementary Note 6 - Chiral symmetry condition of the Floquet system and topological properties under other coupling parameters preserving Chiral symmetry.

Supplementary Note 7 - Calculation of the mean chiral displacement (MCD).

Supplementary Note 8 - Topological phase transition in the 1D lattice.

Supplementary Note 9 - Non-Abelian Transformations Based on Polarization-Controlled Coupling.

Supplementary Note 10 - Terahertz time-domain spectroscopy.

Supplementary Note 11 - Terahertz vector network analyzer.

Supplementary Note 12 - 3D printing technology of sample fabrication.

Supplementary Note 13 - Characterization of resin material parameters.

Supplementary Note 14 - Characterization of metal hollow material parameters.

Supplementary Note 15 - Experimental results using metal hollow waveguides.

Supplementary Note 16 - Comparison between excitation via straight and twisted waveguides.

Supplementary Note 17 - Propagation phenomena in lattices with all waveguides twisted.

Supplementary Note 18 - Theoretical derivation of state evolution in other steps of the X, Y and Z gate.

Supplementary Note 19 - Winding number in two-dimension lattice.

Supplementary Note 20 - Extension to resonator and interferometer platforms.

Supplementary Note 21 - Possible influence of residual TE-TM coupling.

Supplementary Note 22 - Refractive-index correction in the FDTD simulations.

Supplementary Note 23 - Experimental validation of local polarization-controlled coupling.

Supplementary Note 24 - Possible lower-loss implementations.

Supplementary Note 25 - Experimental repeatability and possible sources of the remaining discrepancies.

Supplementary Figures:

Supplementary Figure 1 - Verification of negative coupling and the roles of its sign and functional form.

Supplementary Figure 2 - Supplementary results on flat-band quasi-energy in other structures.

Supplementary Figure 3 - Effective Floquet fermions under extended Floquet Hamiltonian.

Supplementary Figure 4 - Supplementary results for the topological state protected by chiral symmetry.

Supplementary Figure 5 - Robustness of topological states under disorder.

Supplementary Figure 6 - FDTD simulations of Floquet flat-band.

Supplementary Figure 7 - FDTD simulations of period-$n\Lambda$ oscillation.

Supplementary Figure 8 - FDTD simulations of coupling modulation via uniform and relative twisting of waveguides.

Supplementary Figure 9 - Implementation of non-Abelian operations.

Supplementary Figure 10 - Experimental setups of the terahertz time-domain spectroscopy systems.

Supplementary Figure 11 - Experimental setups of the terahertz vector network analyzer.

Supplementary Figure 12 - Terahertz time-domain spectroscopy of air and RG resin films with different thicknesses.

Supplementary Figure 13 - Terahertz characterization of RG resin films and elliptical straight waveguides.

Supplementary Figure 14 - Loss characterization of twisted elliptical waveguides and comparison with straight waveguides.

Supplementary Figure 15 - Measured transmitted intensity as a function of metallic hollow elliptical waveguide length.

Supplementary Figure 16 - Broadband performance of polarization-controlled coupling at different wavelengths.

Supplementary Figure 17 - Resin waveguide samples for loss characterization, coupling coefficient measurement and Floquet flat band experiments.

Supplementary Figure 18 - Resin waveguide samples for the period-n Λ oscillation and topological state experiments.

Supplementary Figure 19 - Metal hollow waveguide samples for the loss characterization, coupling coefficient measurement, Floquet flat band, and topological state experiments.

Supplementary Figure 20 - Experimental verification of Floquet flat-band dynamics using metallic hollow waveguide samples.

Supplementary Figure 21 - Experimental verification of the topological state using metallic hollow waveguide samples.

Supplementary Figure 22 - Extension of polarization-controlled coupling to resonator and interferometer systems.

Supplementary Figure 23 - Topological phenomena in one-dimension lattice.

Supplementary Figure 24 - Realization of logical non-Abelian gates based on polarization-controlled coupling.

Supplementary Figure 25 - Floquet flat band: comparison between TB simulation, FDTD simulation, and THz-TDS experiment.

Supplementary Figure 26 - Period-Λ and Period-2Λ oscillations: comparison between FDTD simulation and experiment.

Supplementary Figure 27 - FDTD simulation with the measured material absorption.

Supplementary Figure 28 - Comparison between FDTD simulations and experimental measurements at different wavelengths for the straight waveguide array after correction of the refractive index used in the simulations.

Supplementary Figure 29 - The schematic of the honeycomb lattice and the phase transition diagram.

Supplementary Figure 30 - Experimental verification of the physical effect of longitudinal coupling-sign reversal in a two-waveguide unit.

Supplementary Figure 31 - Experimental validation of multi-directional polarization-controlled coupling in a four-site unit.

Supplementary Figure 32 - Polarization preservation and reduced propagation loss in a porous resin waveguide.

Supplementary Figure 33 - Optical properties of alumina ceramic and its application to a lower-loss Floquet waveguide array.

Supplementary Figure 34 - Repeatability of representative experimental output distributions at the maximum propagation distance.

Supplementary Figure 35 - Sensitivity of the propagation dynamics to the effective excitation position.

Supplementary Figure 36 - Residual TE-TM mode conversion

Supplementary Tables:

Supplementary Table 1 - Comparison between the present work and representative polarization- or orientation-related experimental photonic platforms.

Supplementary Table 2 - Quantitative comparison between the FDTD-simulated and experimentally measured intensity distributions.

Supplementary Note 1 - Derivation of the negative coupling coefficient and analysis of the roles of its sign and functional form.

According to Coupled-mode theory, the coupling coefficient is proportional to the overlap integral of the transverse electric fields of the two modes

$$\kappa_{pq} = \omega\varepsilon_0 \frac{\iint (n^2 - {n_0}^2)\overrightarrow{E_{p0}^*} \cdot \overrightarrow{E_{q0}} dxdy}{\iint \left(\overrightarrow{E_{p0}} \times \overrightarrow{H_{p0}^*} + \overrightarrow{E_{p0}^*} \times \overrightarrow{H_{p0}}\right) \cdot \vec{z}\, dxdy} \tag{1}$$

$$\kappa_{pq} \propto \overrightarrow{E_{p0}^*}(x_p, y_p) \cdot \overrightarrow{E_{q0}}(x_q, y_q) \tag{2}$$

We set $x_p = x,\ y_p = y,\ x_q = x\cos\theta + y\sin\theta$, and $y_q = -x\sin\theta + y\cos\theta$. For a simplified model, this yields $\kappa_{pq}(\theta) \propto \cos\theta$, reflecting the geometric alignment of the polarization directions. At $\theta = \pi$, the twisted waveguide's polarization reverses to $-x$ and the transverse electric field changes from $\overrightarrow{E_q} = \overrightarrow{E_{q0}}\cos(kz - \omega t + \varphi)\hat{x}$ to $\overrightarrow{E_q} = \overrightarrow{E_{q0}}\cos(kz - \omega t + \varphi)(-\hat{x})$, resulting in $\kappa_{pq}(\pi) = -\kappa_{pq}(0)$.

The emergence of negative coupling coefficients in twisted waveguides arises from the interplay between geometric phase and gauge invariance. During the twisting process, the waveguide's transverse cross-section undergoes adiabatic rotation, introducing an effective "gauge potential" in the parameter space of the propagating mode. This gauge potential, analogous to a Berry connection in quantum systems, modifies the phase accumulation of the electromagnetic field. At a π twist, the twisted waveguide's polarization axis becomes antiparallel to the initial orientation, corresponding to a geometric phase shift of π. Consequently, the overlap integral determining the coupling coefficient $\kappa_{pq}(\theta)$ acquires a negative sign, i.e., $\kappa_{pq}(\pi) = -\kappa_{pq}(0)$.

Although the relative TE polarization angle of 0 and π between two waveguides appears identical in terms of the external geometry, a rotation by π introduces an additional negative sign in the coupling coefficient. To verify the existence of negative coupling, we compared two distinct twisting configurations. In the first configuration (Supplementary Figure 1a(i)), the two waveguides, initially aligned in their TE polarization, are sequentially twisted by $\frac{\pi}{2}$ clockwise, $\frac{\pi}{2}$ counterclockwise, $\frac{\pi}{2}$

clockwise, and $\frac{\pi}{2}$ counterclockwise, yielding an effective coupling described by $\kappa(z) = \kappa_0|\cos(\theta(z))|$. In the second configuration (Supplementary Figure 1b(i)), the waveguides undergo a continuous 2π clockwise twist, leading to a coupling $\kappa(z) = \kappa_0\cos(\theta(z))$. Under otherwise identical parameters (waveguide separation d = 1000 μm, driving period Λ = 12 cm, elliptical cross-section semi-major axis a = 350 μm, semi-minor axis b = 150 μm), light injected from a straight waveguide exhibits dramatically different field evolution within a single driving period, as confirmed by both theoretical analysis and numerical simulations (Supplementary Figure 1a(ii–iii), Supplementary Figure 1b(ii–iii)).

When the polarization angle exceeds π, the induced coupling coefficient becomes negative. This effect can alternatively be interpreted as an effective negative sign introduced to the basis corresponding to one of the waveguides, originating from a geometric phase of π. Under this interpretation, the coupling itself is no longer regarded as negative. To eliminate the negative sign of the coupling coefficient $\kappa(z)$, we introduce a basis transformation from the original Hamiltonian $H(z) = \begin{bmatrix} 0 & \kappa(z) \\ \kappa(z) & 0 \end{bmatrix}$ using the unitary operator $U = \begin{bmatrix} 1 & 0 \\ 0 & -1 \end{bmatrix}$. When $\kappa(z) < 0$, the Hamiltonian can be rewritten as $H(z) = \begin{bmatrix} 0 & -|\kappa(z)| \\ -|\kappa(z)| & 0 \end{bmatrix}$. Applying the basis transformation yields $UH(z)U^\dagger = \begin{bmatrix} 0 & |\kappa(z)| \\ |\kappa(z)| & 0 \end{bmatrix}$, which effectively removes the negative sign by embedding it into the basis. Physically, this operation corresponds to a global inversion of the field amplitude in the second waveguide, indicating that the sign change of the coupling is equivalent to a π-phase flip of one of the basis modes.

We confirmed this numerically by considering the case $\kappa(z) = \kappa_0\cos(\theta(z))$, where the state vector component associated with the twisted waveguide acquires a negative sign when the twisting angle spans from $\frac{\pi}{2}$ to $\frac{3\pi}{2}$. The corresponding effective coupling $\kappa'(z) = \kappa_0|\cos(\theta(z))|$ thus remains strictly positive, while yielding results identical to those obtained from $\kappa(z)$ (Supplementary Figure 1b(ii), Supplementary Figure 1c).

To clarify the respective roles of the sign and the functional form of the coupling coefficient, we compare several coupling modulations between two waveguides. In all cases, the driving period Λ is kept identical to ensure a consistent modulation scale along the propagation direction.

The corresponding waveguide geometries and simulated state evolutions are shown in Supplementary Figure 1. These results indicate that both the sign and the functional form of the coupling coefficient can influence the propagation dynamics of the optical field. When the coupling sign changes along the propagation direction, the effective phase relation between the coupled modes is modified, which leads to qualitatively different evolution behaviors. In contrast, variations in the functional form mainly affect the local magnitude of the coupling strength at each propagation position, thereby introducing quantitative differences in the detailed signal profile.

For example, in Supplementary Figure 1b(ii), d, e, and f(ii), the coupling coefficients are

$$\kappa(z) = \kappa_0 \cos\theta(z), \tag{3}$$

$$\kappa(z) = \begin{cases} \kappa_0 \dfrac{\frac{\Lambda}{4} - z}{\frac{\Lambda}{4}} & \text{if } 0 < z < \dfrac{\Lambda}{2} \\ \kappa_0 \dfrac{z - \frac{3\Lambda}{4}}{\frac{\Lambda}{4}} & \text{if } \dfrac{\Lambda}{2} < z < \Lambda \end{cases}, \tag{4}$$

$$\kappa(z) = \begin{cases} \kappa_0 \dfrac{z}{\frac{\Lambda}{4}} & \text{if } 0 < z < \dfrac{\Lambda}{4} \\ \kappa_0 \dfrac{\frac{\Lambda}{2} - z}{\frac{\Lambda}{4}} & \text{if } \dfrac{\Lambda}{4} < z < \dfrac{3\Lambda}{4}, \\ \kappa_0 \dfrac{z - \Lambda}{\frac{\Lambda}{4}} & \text{if } \dfrac{3\Lambda}{4} < z < \Lambda \end{cases} \tag{5}$$

$$\kappa(z) = \kappa_0 \sin\theta(z). \tag{6}$$

When comparing Supplementary Figure 1b(ii) with Supplementary Figure 1d, or e with f(ii), the positions where the coupling sign reverses remain unchanged, and the differences in the field evolution arise solely from the different functional forms of

$\kappa(z)$, which lead to different coupling amplitudes along the propagation direction. Consequently, the resulting field distributions are not exactly identical.

Nevertheless, for the physical phenomena investigated in the main text, the sign reversal of the coupling coefficient plays the primary role, while the functional form mainly introduces secondary quantitative modifications to the measured signal profile.

Supplementary Note 2 - Broadband performance of polarization-controlled coupling.

The relationship between coupling coefficient and polarization follows a universal physical principle and is therefore insensitive to variations in the wavelength and minor structural imperfections. Although the general behavior of wavelength-dependent coupling remains valid across different structures and wavelengths, any specific device has its own finite bandwidth. Accordingly, we investigate the broadband performance of polarization-controlled coupling. Since the length required for complete coupling between two waveguides is wavelength-dependent, a device with fixed geometry exhibits a limited operational bandwidth.

We define the operational bandwidth as the ratio between the maximum and minimum output intensity from the waveguide,

$$\Delta I(\lambda) = \frac{I_{max}(\lambda) - I_{min}(\lambda)}{I_{max}(\lambda)}. \quad (7)$$

A wavelength is considered within the operational bandwidth if this ratio exceeds 50% over an interaction length of 1 cm, while the relative angle between the waveguides is varied continuously from 0 to π. Given that terahertz time-domain spectroscopy can simultaneously generate terahertz source spanning 0.1-1 THz, we analyzed the performance across this entire frequency range. Based on the results of the two-dimensional terahertz real-time imaging, over the wavelength range of 1000um to 1493um, the ratio consistently exceeds 50%, confirming robust broadband operation of the structure. These results are presented in Supplementary Figure 13.

Supplementary Note 3 - Equal twisting rates of the waveguide during the first and

second halves of the driving period.

Here, we consider $\theta_1 = 0$ and $\theta_2 = \frac{\pi}{2}$, and the coupling coefficients between adjacent waveguides alternate as $\kappa_1(z) = \kappa_0\cos(\theta(z))$ and $\kappa_2(z) = \kappa_0\sin(\theta(z))$. The physical properties are same for $\theta_1 = 0$ and $\theta_2 = \frac{\pi}{2}$.

Although $\mathcal{H}(k, z)$ is a matrix and thus cannot be integrated into a single numerical value equal to zero, as in the previously discussed case, the system nevertheless supports flat bands at specific driving periods. This phenomenon can be understood within the framework of the Magnus expansion. The time evolution operator is expressed as $U(z) = e^{-i\Omega(z)}$, where $\Omega(z)$ is expanded as $\Omega(z) = \Omega_1(z) + \Omega_2(z) + \cdots$, with

$$\Omega_1(z) = \int_0^z H(z_1)dz_1 \quad (8)$$

$$\Omega_2(z) = \frac{i}{2}\int_0^z \int_0^{z_1} [H(z_1), H(z_2)]\, dz_2 dz_1 \quad (9)$$

Since $[H(z), H(z')] \neq 0$, the higher-order terms in the Magnus expansion generally contribute nonzero values, resulting in a nonzero quasienergy spectrum for most driving periods. However, at a specific driving period, denoted as $\Lambda_{flat} = 8.8$ for this system, all higher-order terms beyond $\Omega_1(z)$ sum exactly to zero. Consequently, the quasi-energies are determined solely by the first-order term $\Omega_1(z)$, and take on a value of zero across the entire Brillouin zone. This indicates that the system exhibits a completely flat quasi-energy band in momentum space.

Supplementary Note 4 - Robustness of phenomena under non-sinusoidal coupling (e.g., triangular-wave form).

In realistic structures, the influence of waveguide cross-sectional geometry and mode distribution on evanescent coupling implies that the relationship between the coupling coefficient and the relative polarization angle is not strictly sinusoidal or cosinusoidal, and such a functional form represents an approximation. However, regardless of the exact coupling behavior, as long as the coupling coefficient follows a variation from positive to negative values, the physical phenomena described in our work remain valid.

To demonstrate the universality of these phenomena with respect to sign-changing coupling profiles, the triangular waveform is selected as a representative example in a two-site lattice. We set

$$\kappa_1(z) = \begin{cases} \kappa_0 \dfrac{z}{\frac{\Lambda}{4}} & \text{if } 0 < z < \dfrac{\Lambda}{4} \\ \kappa_0 \dfrac{\frac{\Lambda}{2} - z}{\frac{\Lambda}{4}} & \text{if } \dfrac{\Lambda}{4} < z < \dfrac{3\Lambda}{4} \\ \kappa_0 \dfrac{z - \Lambda}{\frac{\Lambda}{4}} & \text{if } \dfrac{3\Lambda}{4} < z < \Lambda \end{cases} \tag{10}$$

$$\kappa_2(z) = \begin{cases} \kappa_0 \dfrac{\frac{\Lambda}{4} - z}{\frac{\Lambda}{4}} & \text{if } 0 < z < \dfrac{\Lambda}{2} \\ \kappa_0 \dfrac{z - \frac{3\Lambda}{4}}{\frac{\Lambda}{4}} & \text{if } \dfrac{\Lambda}{2} < z < \Lambda \end{cases} \tag{11}$$

and take the Floquet flat band case as an example. The driving period required for the emergence of flat bands still exists; however, due to the fact that the absolute value of the coupling coefficient at each propagation distance z in the triangular wave is smaller than that in the trigonometric form, the driving period Λ_{flat} supporting flat bands is shifted toward longer propagation distances (Supplementary Figure 2a).

Supplementary Note 5 - Effective Floquet fermions under extended Floquet Hamiltonian.

We develop an extended Floquet Hamiltonian framework that reveals the formation of effective Floquet fermions in both one- and two-dimension lattice, along with their associated spectral degeneracies. This framework enables the construction of two equivalent and independent atomic chains in momentum space. In real space, two corresponding atomic lattices can also be formed, which remain congruent under arbitrary conditions.

In both the one-dimension and two-dimension systems discussed above, we observe a global twofold degeneracy across the entire quasi-energy spectrum. This

spectral degeneracy arises in the one-dimensional two-site unit cell model with coupling coefficients defined as $\kappa_1(z) = \kappa_0\cos(\theta(z))$ and $\kappa_2(z) = \kappa_0\sin(\theta(z))$, as well as in the two-dimensional two-site unit cell model where the coupling coefficients are given by $\kappa_1(z) = \kappa_0\cos(\theta(z))$, $\kappa_2(z) = \kappa_0\cos(\theta(z) + \frac{2\pi}{3})$ and $\kappa_3(z) = \kappa_0\sin(\theta(z) + \frac{4\pi}{3})$.

In our Floquet systems, the extended Floquet Hamiltonian can be used to construct independent virtual fermions. The system possesses discrete time-translation symmetry, and according to the requirements of Bloch's theorem[1], the eigenmodes of the system can be written as:

$$|\psi(z)\rangle = |u(z)\rangle e^{-i\varepsilon z}, \tag{12}$$

where $u(z)$ has the same periodicity as the Hamiltonian, and ε is the quasi-energy of the system. Phase factor $\varepsilon\Lambda$ represents the phase accumulation of the mode after one period. We can perform a discrete Fourier transform on the periodic functions $\hat{H}(z)$ and $|u(z)\rangle$:

$$H(z) = \sum_n H_n e^{in\omega z}, \tag{13}$$

$$|u(z)\rangle = \sum_n u_n e^{in\omega z}, \tag{14}$$

where H_n and u_n are the Fourier components of $H(z)$ and $u(z)$, respectively, with the sum running from $-\infty$ to ∞. Substituting equations (7) and (8) into Schrödinger equation and considering only specific frequency components, we obtain

$$\sum_m (H_{n-m} + \delta_{nm} n\omega) u_m = \varepsilon u_n. \tag{15}$$

Using all u_n as basis vectors to form the extended Floquet Hilbert space, we obtain the infinitely large static extended Floquet Hamiltonian, which is a block matrix

$$(H_F)_{nm} = H_{n-m} + \delta_{nm} n\omega. \tag{16}$$

In numerical calculations, the infinite matrix needs to be truncated, typically symmetrically around H_0. This truncation is equivalent to the procedure in the effective Hamiltonian approach[2], where the infinitesimal distance differential dz is replaced by a finite time step Δt in numerical calculations.

We can perform second quantization on the orthogonal basis u_n to construct virtual Floquet fermions. Each Floquet fermion represents the different harmonic components of the original atoms in periodic time evolution, while each real atom is extended into an infinite number of Floquet fermions. The diagonal terms in the extended Floquet Hamiltonian H_F contain $\delta_{nm}n\omega$ terms, indicating that Floquet fermions corresponding to different harmonic orders are in different potentials. There is a specific direction of an effective electric field that breaks the symmetry between positive and negative frequencies. The off-diagonal terms in H_F represent the coupling strengths and topological connections between Floquet fermions.

In momentum space, analyzing a system with a single driving frequency and real-valued coupling coefficients, the extended Floquet Hamiltonian can form two independent atomic chains that are completely equivalent at high-symmetry points, leading to inevitable degeneracy. A general case can be considered: the intracell and intercell coupling strengths have the same driving frequency and no zero-frequency component, but their relative magnitudes and phases can take arbitrary values. Since the system has only a single frequency, its expansion in the frequency domain yields non-zero contributions only for H_1 and $H_{-1} = H_1^\dagger$. H_1 is not the complete Hamiltonian and is not required to be Hermitian. It contains two distinct coupling strengths, which are represented by green and red in the schematic diagram of H_F shown in Fig S3c(i). These two coupling strengths are equal when $e^{ik} = e^{-ik}$. We can construct the Floquet fermions shown on the right side of Fig S2c(i) and determine their coupling connections using the red and green indicators, resulting in the atomic lattice shown in Fig S3c(ii). Since the coupling coefficients are complex, the direction of the coupling is indicated by arrows in the figure, similar to the notation in the Haldane model[3]. Due to the absence of a zero-frequency component, there is no coupling between Floquet fermions of the same order ($A_{n\omega}$ and $B_{n\omega}$), as indicated by the red cross in Fig S3c(ii). This results in the atomic lattice effectively consisting of two independent atomic chains. Because the Floquet fermions are in an effective potential gradient, the two atomic chains are not equivalent and yield different eigenvalues.

However, when $k = 0$ or π, the two atomic chains are congruent and have the identical quasi-energies in momentum space. This means that inevitable degeneracy occurs at the boundaries and the origin of the Brillouin zone, explaining the band structure shown in Fig S3a(ii) and Fig S3b(ii).

For finite systems, each atom can similarly be extended into Floquet fermions, and two independent atomic lattices can be constructed using the extended Floquet Hamiltonian, thereby revealing their degeneracies. The same method of constructing the extended Floquet Hamiltonian determines how Floquet fermions form the atomic lattice. In the actual atomic chain, the coupling strengths between adjacent atoms have no zero-frequency component and possess a single frequency, with different magnitudes and phases allowed within and between cells. Thus, Floquet fermions of adjacent atoms at neighboring levels are coupled, forming the situation depicted in Fig S3c(iii). As long as the coupling strengths for $A_{n\omega} \rightarrow B_{(n+1)\omega}$ and $B_{n\omega} \rightarrow A_{(n+1)\omega}$ are the same within a cell or between cells. As before, the absence of a zero-frequency component ensures that the atomic lattice can be divided into two independent sublattices (solid and dashed lines). When the finite system has a complete lattice (removing the yellow part in Fig S3c(iii), with an even number of atoms), the topological structures of the two independent atomic lattices are identical: mirroring the dashed lattice results in the same connectivity and coupling strengths as the solid lattice. When the atomic potential of the lattice is the same for both A and B, the two congruent, independent lattices yield identical distributions of eigen quasi-energies, indicating that each eigenenergy in the system is doubly degenerate. Since these two degenerate states reside in two distinct virtual networks, no non-Abelian characteristics appear between each pair of degenerate states, although the degeneracy is not broken when the coupling parameters are changed. When the atomic potential of A and B is different, the two atomic lattices have the same connectivity and strength but different atomic potentials, and the degeneracy of the eigenmodes is lifted. Another way to break the degeneracy is to construct incomplete unit cells: the presence of isolated atoms similar to the yellow part in Fig S3c(iii). In this case, the two atomic lattices are not equal, and there is no

quasi-energy degeneracy.

Supplementary Note 6 - Chiral symmetry condition of Floquet system and topological properties under other coupling parameters preserving Chiral symmetry.

For a static Hamiltonian $H(k)$, chiral symmetry is defined by $\Gamma H(k)\Gamma^{-1} = -H(k)$. In contrast, for a Floquet system described by the time-evolution operator $U(\Lambda) = \hat{T}e^{-i\int_0^{\Lambda} H(z)\,dz}$, the situation is more complex. Although a time-dependent Hamiltonian may satisfy the instantaneous relation $\Gamma H(z)\Gamma^{-1} = -H(z)$, this condition alone does not guarantee chiral symmetry of the full Floquet operator $U(\Lambda)$. To ensure chiral symmetry over an entire driving period, the Floquet operator must instead satisfy

$$\Gamma U(\Lambda)\Gamma^{-1} = U^{-1}(\Lambda), \tag{17}$$

which implies that quasienergies appear in symmetric pairs $\epsilon \leftrightarrow -\epsilon$ (mod $2\pi/\Lambda$). This requirement can be fulfilled if the instantaneous Hamiltonian satisfies the generalized condition

$$\Gamma H(z)\Gamma^{-1} = -H(\Lambda - z) = -H(-z), \tag{18}$$

meaning that the driving protocol possesses a "mirror" anti-symmetry of the driving profile with respect to a reference point (e.g., z=0), corresponding to a inversion symmetry of the modulation profile. This relation can be explicitly verified through the following derivation.

Applying the chiral transformation to the time-ordered exponential gives

$$\Gamma U(\Lambda)\Gamma^{-1} = \Gamma\hat{T}e^{-i\int_0^{\Lambda} H(z)\,dz}\Gamma^{-1} = \hat{T}e^{i\int_0^{\Lambda} H(\Lambda-z)\,dz}. \tag{19}$$

Introducing a change of variable $z' = \Lambda - z$, the integration limits are inverted, leading to

$$\int_0^{\Lambda} H(\Lambda - z)\,dz = \int_{\Lambda}^{0} H(z')\,(-dz') = \int_0^{\Lambda} H(z')\,dz'. \tag{20}$$

Meanwhile, the time-ordering operator $\hat{T}$ reverses its order under the transformation $z' = \Lambda - z$, which converts $\hat{T}$ into its inverse $\hat{T}^{-1}$. Consequently,

$$\Gamma U(\Lambda)\Gamma^{-1} = \hat{T}^{-1}e^{i\int_0^{\Lambda} H(z')\,dz'} = \left[\hat{T}e^{-i\int_0^{\Lambda} H(z')\,dz'}\right]^{-1} = U^{-1}(\Lambda). \qquad (21)$$

This completes the proof that the Floquet operator satisfies the chiral symmetry relation $\Gamma U(\Lambda)\Gamma^{-1} = U^{-1}(\Lambda)$.

Two-site lattice with $\boldsymbol{\kappa_1(z) = \kappa_0|\sin(\theta(z))|}$ and $\boldsymbol{\kappa_2(z) = \kappa_0\cos(\theta(z))}$:

As discussed in the main text (titled "Floquet Flat Bands and Period-nΛ Oscillations"), when the system supports chiral symmetry, topologically protected edge states emerge within a specific range of driving periods. For the system considered in "Floquet Flat bands and Period-nΛ Oscillations" in the main text, the coupling coefficient $\kappa_1(z) = \kappa_0\cos(\theta(z))$, and $\kappa_2(z) = \kappa_0\sin(\theta(z))$do not satisfy the chiral symmetry condition $\Gamma H(k,z)\Gamma^{-1} = -H(k,-z)$. To construct topological edge states protected by chiral symmetry, we instead define $\kappa_1(z) = \kappa_0|\sin(\theta(z))|$, and $\kappa_2(z) = \kappa_0\cos(\theta(z))$, implemented by twisting the waveguide first clockwise by π and then counterclockwise by π. Supplementary Figure 4a(i) shows the quasi-energy spectrum under OBCs with 80 waveguides as a function of the driving period Λ, where topological edge states appear on the 0 gap.

Four-site lattice:

Moreover, we set two distinct driving period in the system, one of which is twice than the other. The system is a four-site lattice now, resulting in coupling coefficients $\kappa_1(z) = \kappa_0|\sin(\theta(z))|$, $\kappa_2(z) = \kappa_0|\cos(\theta(z))|$, $\kappa_3(z) = \kappa_0|\sin(2\theta(z))|$ and $\kappa_4(z) = \kappa_0|\cos(2\theta(z))|$. Supplementary Figure 4c(i) shows the quasi-energy spectrum and both 0 and π gaps open based on two different driving periods within the range $\Lambda > 3.65$. A Z-valued invariant[4] can be defined for the quasi-energy gap at 0 for a 1D periodically driven system with chiral symmetry, which also applies to our model. The invariants G_0, corresponding to the zero gaps, can be calculated through

$$G_0 = \frac{i}{2\pi}\int_{-\pi}^{\pi} \mathrm{tr}((T_0^+)^{-1}\partial_k T_0^+)dk \qquad (22)$$

The numerical results of G_π and G_0 as a function of Λ for the two-site lattice and four-site lattice are separately presented in Supplementary Figure 4c(ii) and Supplementary Figure 4c(iii).

A distinct feature of this model is the simultaneous emergence of edge states in both 0 and π gaps. When light is injected from the edge of the waveguide array, it propagates along the edge with an oscillation characterized by a 2T period. We now analyze the origin of the rigid period-2T oscillation. Consider a superposition of Floquet eigenstates with distinct quasi-energies, such as $|\psi_{\pm}(z)\rangle = e^{-i\epsilon_o z}|0\rangle \pm e^{-i\epsilon_\pi z}|\pi\rangle$, where $|0\rangle$ and $|\pi\rangle$ denote localized edge modes with quasienergies ϵ_o and ϵ_π, respectively. These modes acquire different phase factors under the time-evolution operator $U(\Lambda)$ over one driving cycle. Due to the quasienergy difference $|\epsilon_\pi - \epsilon_0| = \pi/\Lambda$, the superposition state $|\psi_{\pm}(z)\rangle$ evolves with a doubled period, satisfying $|\psi_{\pm}(z+2\Lambda)\rangle = |\psi_{\pm}(z)\rangle$. For the four-site lattice, the light does not return after a single period but instead reappears at the same site after two periods 2Λ (Supplementary Figure 4e), indicating a period-doubled dynamical response.

Supplementary Note 7 - Calculation of the mean chiral displacement (MCD).

We discuss a method to verify whether the edge states in the system are topologically protected. If chiral symmetry is not satisfied, edge states may still exist, but they are not topologically protected. The robustness of localized states can be evaluated by introducing controlled disorder into the coupling coefficients and analyzing the system's response. In particular, we apply random perturbations to the coupling values. By calculating the mean chiral displacement (MCD)[5]—a quantized topological indicator—before and after introducing disorder, we distinguish topologically protected edge states from trivial edge modes. The MCD is a long-time averaged value of a bulk observable:

$$\langle m(t)\rangle = \sum_m [m\Gamma_a|a_m(t)|^2 + m\Gamma_b|b_m(t)|^2]. \tag{23}$$

We take 1 for Γ_a and -1 for Γ_b.For systems with two energy levels,

$$\langle m\rangle_{aver} = \frac{1}{T}\int_0^T dt\langle m(t)\rangle = \frac{\mathcal{W}}{2}. \tag{24}$$

The results for twisted waveguide arrays with structure in Fig. 4a and

Supplementary Figure 4a are separately shown in Supplementary Figure 5a and Supplementary Figure 5b. For the system protected by chiral symmetry, the presence of disorder does not affect the existence of edge states. In contrast, in the absence of chiral symmetry protection, disorder of the same strength leads to the disappearance of the edge states.

Supplementary Note 8 - Topological phase transition in the 1D lattice.

To satisfy the chiral symmetry condition, the evolution operator $U(k,\Lambda)$ must obey $\Gamma U(k,\Lambda)\Gamma^{-1} = U^{-1}(k,\Lambda)$[6]. This requirement implies a symmetric inversion point along propagation, $H(k,z) = H(k,-z)$ (Supplementary Note 6), together with the local chiral symmetry relation $\Gamma H(k,z)\Gamma^{-1} = H^{-1}(k,z)$. Setting $\theta_1 = \frac{\pi}{2}$ and $\theta_2 = 0$ (Supplementary Figure 23a(i)), the waveguides twist by $\frac{\pi}{2}$ and then reverse direction. This configuration (Supplementary Figure 23a(ii)) yields coupling coefficients $\kappa_1(z) = \kappa_0|\sin(\theta(z))|$ and $\kappa_2(z) = \kappa_0|\cos(\theta(z))|$ (more situations are discussed in Supplementary Note 6). Supplementary Figure 23a(iii) shows the quasi-energy spectrum under OBCs with 80 waveguides as a function of the driving period Λ, where anomalous Floquet π modes appear within the range $\Lambda > 2.5$. The topological invariant G_π[4] can be defined for the quasi-energy gap at π as:

$$G_\pi = \frac{i}{2\pi}\int_{-\pi}^{\pi} \mathrm{tr}((T_\pi^+)^{-1}\partial_k T_\pi^+)dk\,, \tag{25}$$

where T_π^+ is the diagonal matrix element of $T(\frac{\Lambda}{2},k)$, and $T(z,k) \equiv U(z,k)e^{iH_F(k)z}$. The numerical result of G_π as a function of Λ for the two-site lattice is presented in Supplementary Figure 23a(iv). As Λ increases, G_π changes from 0 to -1, and then to -2, corresponding to 0, 2, and 4 edge states. This realization of higher-winding ($|G_\pi| > 1$) phases in the topological waveguide array enabled by polarization-controlled Floquet engineering stands in contrast to previous schemes, which typically required next-nearest-neighbor interactions[7] or asymmetric couplings[8].

Theoretical evolutions of the bulk states and edge states are shown separately in Supplementary Figure 23b(i) and Supplementary Figure 23b(iii), where the Floquet

driving period is Λ=4cm for both cases. The topological edge state still propagates along the edge with periodic-driven oscillation and a negligible amount of dissipation into the bulk, whereas the bulk state rapidly delocalizes throughout the array. Similarly, in order to show the scalability of the proposed polarization-controlled strategy, additional experiments on hollow metallic waveguides are demonstrated in Supplementary Supplementary Figure 21.

Supplementary Note 9 - Non-Abelian Transformations Based on Polarization-Controlled Coupling.

Beyond the realization of Floquet-driven phenomena, our platform further enables the implementation of non-Abelian transformations. In previous schemes[9,10], only closed adiabatic trajectories on the Bloch sphere were realized, restricting the accessible operations to pure rotations belonging to the special orthogonal group SO(2), and the realization of non-Abelian transformations requires a degenerate subspace of more than two zero modes. In contrast, the polarization-controlled platform developed here supports both closed and non-closed adiabatic evolution trajectories of zero modes in the Bloch-sphere parameter space. Consequently, the accessible operation group is extended from SO(2) to O(2), thereby expanding the family of achievable matrix operations beyond those defined by closed loops. This capability enables our system to realize a broader class of non-Abelian transformations that are inaccessible in previous platforms, and implement them within a subspace spanned by as few as two zero modes.

This capability allows straightforward realization of Pauli-X, Y and Z gates. For the logical Z gate, $Z = \begin{bmatrix} 1 & 0 \\ 0 & -1 \end{bmatrix}$, the structure consists of one straight waveguide A and one twisted waveguide B by π (Supplementary Figure 24a(i)). The large spacing between the two waveguides suppresses coupling. As the polarization direction of TE mode in B rotates from +x to -x, a geometric phase of π is accumulated, effectively introduces a factor of -1 the logical state, as illustrated in Supplementary Figure 24a(ii). The experimental output fields and phases at the end facet are shown in Supplementary Figure 24a(iii). After twisting waveguide B, the electric field remains localized in

waveguides A and B, while the relative phase between the two outputs is locked at π. The phase shown here corresponds to the total accumulated phase (dynamical + geometric). Only the relative phase difference is physically meaningful. This phase shift originates from a geometric rather than a dynamical phase, as confirmed by its invariance when the input wavelength is varied from 1160 μm to 1420 μm.

We next implement the logical X gate, $X = \begin{bmatrix} 0 & 1 \\ 1 & 0 \end{bmatrix}$, using a four-waveguide structure composed of three twisted waveguides (A, B, and C) and one straight waveguide (X) (Supplementary Figure 24b(i)). The coupling coefficients are given by $\kappa_{iX} = \kappa_0 \cos(\theta_{iX}(z))$ (i=A, B, C). The logical X gate is achieved through three sequential twisting operations, each involving a pair of twisted waveguides rotated by $\frac{\pi}{2}$ in the same direction simultaneously. Light is initially injected equally into waveguides A and B. In the first step, twisting A and C yields $\theta_{CX}(z) = \theta(z)$ and $\theta_{AX}(z) = \theta(z) + \frac{\pi}{2}$, corresponding to coupling coefficients $\kappa_0 \cos\theta(z)$ and $-\kappa_0 \sin\theta(z)$, respectively. As θ evolves from 0 to $\frac{\pi}{2}$, the zero eigenmodes $\psi_1 = (\cos\theta(z), 0, \sin\theta(z), 0)^T$ and $\psi_2 = (0, 1, 0, 0)^T$ transform continuously, transferring the optical field from A into C, as illustrated in Supplementary Figure 24b(ii). The subsequent two steps proceed similarly: after all three operations, one zero mode evolves from $A \to C \to B$, and the other from $B \to A$, both acquiring a total geometric phase of π. This process realizes a unitary operation equivalent to the logical X gate, corresponding to a non-closed adiabatic trajectory on the Bloch sphere.

The logical Y gate, $Y = \begin{bmatrix} 0 & -i \\ i & 0 \end{bmatrix}$, can be equivalently realized by $\begin{bmatrix} 0 & -1 \\ 1 & 0 \end{bmatrix}$. Steps 1 and 2 are identical to those of the X gate, while in step 3, waveguide C is twisted $\frac{\pi}{2}$ in the opposite direction. After the three steps, the two zero modes acquire a relative phase difference of π. The resulting operation corresponds to the logical Y gate, corresponding to a closed adiabatic trajectory on the Bloch sphere. The detailed derivations for all three gates are provided in Supplementary Note 18.

These operations can be combined to perform more complex non-Abelian transformations within the subspace spanned by two or more zero modes. This approach

provides a practical pathway toward applying such transformations in quantum information processing and photonic quantum computing.

Supplementary Note 10 -Terahertz time-domain spectroscopy.

The experiment is performed in the terahertz regime, using terahertz time-domain spectroscopy (THz-TDS) combined with two-dimensional terahertz real-time imaging. A schematic of the system is shown in Fig. 2a. The system employs a Ti:sapphire regenerative amplifier as the light source, which generates ultrashort pulses with a central wavelength of 800 nm, a pulse duration of 100 fs, a repetition rate of 1 kHz, and a beam diameter of 8 mm. The average output power of the laser is 900 mW, which is split into two beams: a pump beam with an energy of 880 mW and a probe beam with 20 mW. The pump beam is vertically incident on a 1 mm-thick <110>-oriented ZnTe crystal. Through the crystal's second-order nonlinear optical effect—optical rectification—terahertz pulses are generated. The emitted terahertz pulses are horizontally polarized with a beam diameter of 24 mm. After passing through a terahertz wire-grid polarizer, the terahertz wave is incident on the sample. In the probe arm, a half-wave plate and a polarizer are used in combination to adjust the polarization state of the probe light. The beam is then divided by a 50:50 beam splitter. The reflected portion is directed perpendicularly onto a sensing crystal. This system utilizes free-space electro-optic sampling based on the Pockels effect. Within the sensing crystal, the polarization state of the probe beam is modulated by the terahertz field. The modulated probe light, which carries terahertz field information, is reflected from the crystal and passed through a 4f optical system. It is then separated by a polarizing beam splitter into two orthogonally polarized beams, which are focused onto a CCD camera. Using balanced electro-optic detection, the complex amplitude of the terahertz field can be extracted and amplified from the captured images. By varying the optical path difference between the terahertz generation and probe paths, a terahertz time-domain waveform is obtained. At each time-delay point, 100 images are captured by the CCD and averaged. Fourier transformation of the time-domain data yields terahertz images at specific frequencies, and also allows extraction of the terahertz signal from individual

pixels.

Supplementary Note 11 - Terahertz vector network analyzer.

Compared with the previous THz-TDS measurements, the results obtained using the vector network analyzer (VNA) with frequency extension modules are substantially clearer and less affected by noise. This improvement mainly arises from the measurement principle of the VNA, which is based on swept continuous-wave excitation and coherent detection. Because the received signal is mixed with a phase-locked reference at the same frequency, the system selectively extracts the signal component that is coherent with the excitation while efficiently suppressing incoherent noise contributions. In addition, the narrow intermediate-frequency bandwidth of the VNA receiver further improves the signal-to-noise ratio and dynamic range. Under our measurement conditions, signals down to approximately -100 dB can still be reliably resolved, which explains the lower noise floor and the more visually distinct experimental results obtained with the VNA-based system.

Supplementary Note 12 - 3D printing technology of sample fabrication.

Projection Micro Stereolithography (PμSL) is an area projection photopolymerization-based 3D printing technology particularly suitable for fabricating complex three-dimensional microstructures. The PμSL printing process involves several key steps. A three-dimensional structural model is designed using modeling software. This model is then sliced into a series of two-dimensional images with specific patterns using slicing software, with each layer having a defined thickness. The PμSL 3D printing system subsequently projects each sliced layer for full-area exposure. By repeatedly executing this exposure step and stacking the cured layers, the desired three-dimensional structure is gradually fabricated. The fundamental mechanism of PμSL relies on the photopolymerization reaction of photosensitive resin under ultraviolet (UV) light, leading to solidification. The generation of specific patterns is achieved through a digital dynamic mask created by a Digital Micromirror Device (DMD) chip within the printing system. After importing the sliced model data into the printing system, the two-

dimensional image data is transmitted to the DMD. The DMD controls the tilt of each micromirror (corresponding to individual pixels) based on the image data. As a result, UV light from the source is spatially modulated by the DMD to form a light pattern identical to the graphic data. Finally, the modulated light is projected through an objective lens onto the surface of the liquid resin, selectively exposing specific regions to solidify the desired structure. PμSL 3D printing technology achieves a minimum feature resolution of 2 μm, enabling high-resolution, large-area, and multi-scale fabrication. Our samples were fabricated using the commercial resin designated as RG. The RG resin exhibits a hardness of 77 shore D and an elastic modulus of 1765 MPa, indicating a rigid and mechanically robust material. Its viscosity at 25°C is 1100 cp and it demonstrates low water absorption (0.77% after 24 hours) and is hydrophilic, with a contact angle between 45-60°. Besides, its dielectric constant at 10 GHz is 2.94, coupled with a low dielectric loss tangent of 0.0197.

Supplementary Note 13 - Characterization of resin material parameters.

We first characterized the real and imaginary parts of the refractive index using resin slabs with a cross-sectional area of 1×1 cm² and varying thicknesses. The optical response of the samples is characterized using reflection spectroscopy. The measured time-domain signals from the sample and a reference mirror, $E_{sam}(t)$ and $E_{ref}(t)$, are Fourier transformed to yield their complex spectra $E_{sam}(\omega)$ and $E_{ref}(\omega)$. Taking into account multiple internal reflections, the complex transmission function is defined as

$$T(\omega)=\frac{E_{sam}(\omega)}{E_{ref}(\omega)}=\frac{4N(\omega)}{[N(\omega)+1]^2}\cdot\frac{\exp\left\{-i[N(\omega)-1]\frac{\omega d}{c}\right\}}{1-\frac{[N(\omega)-1]^2}{[N(\omega)+1]^2}\exp\left\{-i2N(\omega)\frac{\omega d}{c}\right\}},\qquad(26)$$

where $N(\omega)=n(\omega)+i\kappa(\omega)$ is the complex refractive index, d is the sample thickness, and c is the speed of light in vacuum. For sufficiently thick samples, the echo signals due to round-trip propagation are well separated in time and can be neglected by choosing an appropriate time window. In this case, the transmission function simplifies to

$$T(\omega)=\frac{E_{\mathrm{sam}}(\omega)}{E_{\mathrm{ref}}(\omega)}=\frac{4n(\omega)}{[n(\omega)+1]^2}\exp\left\{-i[n(\omega)-1]\frac{\omega d}{c}\right\}. \tag{27}$$

For practical analysis, $T(\omega)$ is expressed in terms of its modulus and phase as $T(\omega)=Ae^{i\omega+\varphi}$. Under the weak absorption approximation $\kappa(\omega)\ll n(\omega)$, the real and imaginary parts of the refractive index can be extracted as

$$n(\omega)=\frac{\phi(\omega)\cdot c}{\omega d}+1, \tag{28}$$

$$\kappa(\omega)=\frac{ln\left\{\rho(\omega)\cdot\frac{[n_1(\omega)+1]^2}{4n_1(\omega)}\right\}c}{\omega d}. \tag{29}$$

The absorption coefficient then follows as

$$\alpha(\omega)=\frac{2\omega\kappa(\omega)}{c}. \tag{30}$$

Through this procedure, the THz time-domain signals are Fourier transformed to yield the refractive index, absorption spectrum, and other optical constants of the sample. For non-film samples, multiple reflections inside the material lead to Fabry–Perot interference, which must be eliminated either by preparing sufficiently thick samples or by post-processing to suppress the echoes.

To account for additional losses arising from the finite waveguide cross-section and the focused THz beam, we further measure the absorption coefficient by resin waveguides. Specifically, we fabricated nine single waveguide samples of different lengths (0.2 cm, 0.3 cm, 0.5 cm, 0.8 cm, 1 cm, 2 cm, 2.5 cm, 3 cm and 4 cm), kept the incident beam conditions identical, and measured the optical field strength at the output facet. The dependence of the output field on waveguide length, as shown in Supplementary Figure 13, yields the loss coefficient of the resin material.

The refractive index measured from the bulk resin is used as the initial value in the FDTD simulations. However, the refractive index of the fabricated waveguides can be slightly different from this value. A small offset in the refractive index used in the simulation changes the inter-waveguide coupling strength. Over a long propagation distance, this small difference can lead to a noticeable change in the output intensity distribution.

To account for this effect, we determine a single refractive-index correction of $\Delta n=0.02$ from the straight-waveguide control sample. This correction is used to

correct the small difference between the refractive index used in the original simulation and that corresponding to the fabricated waveguides. After $\Delta n = 0.02$ is determined, the same corrected refractive index is used for all wavelengths and propagation distances without further adjustment. As shown in Supplementary Figure 28, the corrected FDTD results show improved agreement with the experimental results at 1300, 1250, and 1200 μm.

Supplementary Note 14 - Characterization of metal hollow material parameters.

We also fabricated experimental samples using a high-precision metal 3D printing process to produce hollow waveguides. To characterize the waveguide loss, we manufactured eight identical metal hollow waveguides with lengths of 0.5 cm, 1 cm, 1.5 cm, 2 cm, 2.5 cm, 3 cm, 3.5 cm and 4 cm. Under identical input light conditions, we measured the optical field strength at the output end of each waveguide. The relationship between the output field strength and the waveguide length are shown in Supplementary Figure 15, reflecting the loss coefficient of the metal material.

Supplementary Note 15 - Coupling analysis of hollow metallic waveguides and experimental sample design.

In metal hollow waveguide lattices, the coupling mechanism between adjacent waveguides differs significantly from that in dielectric (e.g., resin) waveguides. Since each metal hollow waveguide is surrounded by metallic cladding, the coupling coefficient cannot be accurately modeled using free-space overlap integrals. Nevertheless, by introducing narrow slit channels between pairs of waveguides, coupling between the same modes of two neighboring waveguides can still be achieved. Since metallic surfaces do not support electric fields polarized parallel to the surface, only the field component polarized orthogonal to the slit direction can couple through the slit, enabling interaction between two waveguides. Let the polarization direction of the first waveguide form an angle $\theta(z)$ with the axis orthogonal to the slit, and the polarization direction of the second waveguide form an angle $\theta(z) + \theta_0$ with the same axis. The coupling between the two waveguides then satisfies $\kappa(z) =$

$\kappa_0 \cos(\theta(z)) \cos(\theta(z) + \theta_0) = \frac{\kappa_0}{2} \cos(\theta_0) \left(1 + \frac{1}{\cos(\theta_0)} \cos(2\theta(z) + \theta_0)\right)$. Under the condition of $\frac{1}{\cos(\theta_0)} > 1$, negative coupling can be introduced. Based on this coupling mechanism, we designed a staggered cross-sectional waveguide arrangement to suppress the influence of next-nearest-neighbor coupling.

Supplementary Note 16 - Comparison between excitation via straight and twisted waveguides.

In the system studied in the main text (titled "Floquet Flat Bands and Period-nΛ Oscillations"), although the waveguides differ structurally—being either twisted or straight—theoretical analysis indicates that the Floquet flat band phenomenon emerges irrespective of whether light is injected into a twisted or a straight waveguide. To verify this, we conducted FDTD simulations under two distinct excitation conditions: injection into a straight waveguide and injection into a twisted waveguide (Supplementary Figure 6). The results confirm that the output light distribution remains unchanged regardless of whether the injection is performed through a straight or twisted waveguide, provided that the coupling coefficients between the excited waveguide and its two nearest-neighbor waveguides are maintained identically in both configurations.

This finding, that wave propagation depends not on the type of injection waveguide (straight or twisted), but rather on the spatial profile of inter-waveguide coupling, is also applicable to other photonic structures discussed in the other parts in the main text.

Supplementary Note 17 - Propagation phenomena in lattices with all waveguides twisted.

First, all waveguides are twisted uniformly using the same method (Supplementary Figure 8a), causing the coupling coefficient to oscillate between the values of κ_0 and κ_0'. The simulation results in FDTD are shown in Supplementary Figure 8b. Second, all waveguides were twisted such that the initial relative angle between adjacent waveguides differed by $\frac{\pi}{2}$ (Supplementary Figure 8c), which theoretically suppresses nearest-neighbor coupling. The FDTD simulation results for this configuration are

shown in Supplementary Figure 8d.

Supplementary Note 18 - Theoretical derivation of state evolution in other steps of the X, Y and Z gate.

The orientation angle is defined as the angle relative to the positive y-axis, with the clockwise direction taken as positive. At each step, the smaller orientation angle between the waveguide and the positive y-axis is denoted as $\theta(z)$.

X gate:

For step 1, as $\theta(z)$ varies from 0 to $\frac{\pi}{2}$, the relative polarization angle between waveguides X and C is $\theta(z)$, resulting in a coupling coefficient of $\kappa_0\cos\theta(z)$. Meanwhile, the angle between waveguides X and A is $\theta(z)+\frac{\pi}{2}$, leading to a coupling coefficient of $-\kappa_0\sin\theta(z)$. When the modulations on κ_{ix} are sufficiently slow, the Hamiltonian is

$$H(z)=\begin{bmatrix} 0 & 0 & 0 & -\kappa_0\sin\theta(z) \\ 0 & 0 & 0 & 0 \\ 0 & 0 & 0 & \kappa_0\cos\theta(z) \\ -\kappa_0\sin\theta(z) & 0 & \kappa_0\cos\theta(z) & 0 \end{bmatrix}. \tag{31}$$

$H(z)$ supports two zero eigenmodes, whose eigenvectors are $\psi_1=(0,1,0,0)^T$ and $\psi_2=(\cos\theta(z),0,\sin\theta(z),0)^T$. After step 1, ψ_2 changes from $(1,0,0,0)^T$ to $(0,0,1,0)^T$.

For step 2, as $\theta(z)$ varies from $\frac{\pi}{2}$ to π, the relative polarization angle between waveguides X and B is $\theta(z)$, resulting in a coupling coefficient of $\kappa_0\cos\theta(z)$. Meanwhile, the angle between waveguides X and A is $\theta(z)+\frac{\pi}{2}$, leading to a coupling coefficient of $-\kappa_0\sin\theta(z)$. When the modulations on κ_{ix} are sufficiently slow, the Hamiltonian is

$$H(z)=\begin{bmatrix} 0 & 0 & 0 & -\kappa_0\sin\theta(z) \\ 0 & 0 & 0 & \kappa_0\cos\theta(z) \\ 0 & 0 & 0 & 0 \\ -\kappa_0\sin\theta(z) & \kappa_0\cos\theta(z) & 0 & 0 \end{bmatrix}. \tag{32}$$

$H(z)$ supports two zero eigenmodes, whose eigenvectors are $\psi_1=(0,0,1,0)^T$ and $\psi_2=(\cos\theta(z),\sin\theta(z),0,0)^T$. After step 2, ψ_2 changes from $(0,1,0,0)^T$ to

$(-1,0,0,0)^T$. Besides the dynamic phase, an additional geometric phase of π is accumulated.

For step 3, as $\theta(z)$ varies from $\frac{\pi}{2}$ to π, the relative polarization angle between waveguides X and C is $\theta(z)$, resulting in a coupling coefficient of $\kappa_0\cos\theta(z)$. Meanwhile, the angle between waveguides X and B is $\theta(z)+\frac{\pi}{2}$, leading to a coupling coefficient of $-\kappa_0\sin\theta(z)$. When the modulations on κ_{ix} are sufficiently slow, the Hamiltonian is

$$H(z)=\begin{bmatrix} 0 & 0 & 0 & 0 \\ 0 & 0 & 0 & -\kappa_0\sin\theta(z) \\ 0 & 0 & 0 & \kappa_0\cos\theta(z) \\ 0 & -\kappa_0\sin\theta(z) & \kappa_0\cos\theta(z) & 0 \end{bmatrix}. \quad (33)$$

$H(z)$ supports two zero eigenmodes, whose eigenvectors are $\psi_1=(1,0,0,0)^T$ and $\psi_2=(0,\cos\theta(z),\sin\theta(z),0)^T$. After step 3, ψ_2 changes from $(0,0,1,0)^T$ to $(0,-1,0,0)^T$. Besides the dynamic phase, an additional geometric phase of π is accumulated. For waveguide A and B, the three-step coupling sequence effectively realizes the operation described by the matrix $\begin{bmatrix} 0 & 1 \\ 1 & 0 \end{bmatrix}$.

Y gate:

For step 1, as $\theta(z)$ varies from 0 to $\frac{\pi}{2}$, the relative polarization angle between waveguides X and C is $\theta(z)$, resulting in a coupling coefficient of $\kappa_0\cos\theta(z)$. Meanwhile, the angle between waveguides X and A is $\theta(z)+\frac{\pi}{2}$, leading to a coupling coefficient of $-\kappa_0\sin\theta(z)$. When the modulations on κ_{ix} are sufficiently slow, the Hamiltonian is

$$H(z)=\begin{bmatrix} 0 & 0 & 0 & -\kappa_0\sin\theta(z) \\ 0 & 0 & 0 & 0 \\ 0 & 0 & 0 & \kappa_0\cos\theta(z) \\ -\kappa_0\sin\theta(z) & 0 & \kappa_0\cos\theta(z) & 0 \end{bmatrix}. \quad (34)$$

$H(z)$ supports two zero eigenmodes, whose eigenvectors are $\psi_1=(0,1,0,0)^T$ and $\psi_2=(\cos\theta(z),0,\sin\theta(z),0)^T$. After step 1, ψ_2 changes from $(1,0,0,0)^T$ to $(0,0,1,0)^T$.

For step 2, as $\theta(z)$ varies from $\frac{\pi}{2}$ to π, the relative polarization angle between

waveguides X and B is $\theta(z)$, resulting in a coupling coefficient of $\kappa_0\cos\theta(z)$. Meanwhile, the angle between waveguides X and A is $\theta(z)+\frac{\pi}{2}$, leading to a coupling coefficient of $-\kappa_0\sin\theta(z)$. When the modulations on κ_{ix} are sufficiently slow, the Hamiltonian is

$$H(z)=\begin{bmatrix} 0 & 0 & 0 & -\kappa_0\sin\theta(z) \\ 0 & 0 & 0 & \kappa_0\cos\theta(z) \\ 0 & 0 & 0 & 0 \\ -\kappa_0\sin\theta(z) & \kappa_0\cos\theta(z) & 0 & 0 \end{bmatrix}. \quad (35)$$

$H(z)$ supports two zero eigenmodes, whose eigenvectors are $\psi_1=(0,0,1,0)^T$ and $\psi_2=(\cos\theta(z),\sin\theta(z),0,0)^T$. After step 2, ψ_2 changes from $(0,1,0,0)^T$ to $(-1,0,0,0)^T$. Besides the dynamic phase, an additional geometric phase of π is accumulated.

For step 3, as $\theta(z)$ varies from $\frac{\pi}{2}$ to π, waveguide C is twisting clockwise (i.e., in the reverse direction), and the relative polarization angle between waveguides X and C is $\pi-\theta(z)$,, resulting in a coupling coefficient of $-\kappa_0\cos\theta(z)$. Meanwhile, the angle between waveguides X and B is $\theta(z)+\frac{\pi}{2}$, leading to a coupling coefficient of $-\kappa_0\sin\theta(z)$. When the modulations on κ_{ix} are sufficiently slow, the Hamiltonian is

$$H(z)=\begin{bmatrix} 0 & 0 & 0 & 0 \\ 0 & 0 & 0 & -\kappa_0\sin\theta(z) \\ 0 & 0 & 0 & -\kappa_0\cos\theta(z) \\ 0 & -\kappa_0\sin\theta(z) & -\kappa_0\cos\theta(z) & 0 \end{bmatrix}. \quad (36)$$

$H(z)$ supports two zero eigenmodes, whose eigenvectors are $\psi_1=(1,0,0,0)^T$ and $\psi_2=(0,\cos\theta(z),-\sin\theta(z),0)^T$. After step 3, ψ_2 changes from $(0,0,-1,0)^T$ to $(0,-1,0,0)^T$, without any accumulated geometric phase. For waveguide A and B, the three-step coupling sequence effectively realizes the operation $\begin{bmatrix} 0 & -i \\ i & 0 \end{bmatrix}$.

Z gate:

The structure concludes two waveguide A and B and there is no coupling between them. In the twisting step, waveguide B is twisted counterclockwise by π, which causes the state ψ_2 to transform from $(0,1)^T$ to $(0,-1)^T$, while ψ_1 remains unchanged at $(1,0)^T$. This process effectively realizes the operation $\begin{bmatrix} 1 & 0 \\ 0 & -1 \end{bmatrix}$.

Supplementary Note 19 - Winding number in two-dimension lattice.

The above definition requires that $U(k,z)$ be periodic both in propagation direction z and in momentum space (k_x, k_y). However, this condition is not automatically satisfied in general. Therefore, it is necessary to construct a smooth evolution operator $U_\epsilon(k,z)$ that is periodic in the three-dimensional parameter space, defined as follows:

$$U_\epsilon(k,z) = \begin{cases} U(k,2z) & \text{if } 0 \le z \le \Lambda/2 \\ V_\epsilon(k, 2\Lambda - 2z) & \text{if } \Lambda/2 \le z \le \Lambda \end{cases}, \quad (37)$$

where

$$V_\epsilon(k,z) = e^{-iH_{\text{eff}}(k)z}. \quad (38)$$

By appropriately choosing the branch cut of the complex logarithm function, the winding number calculation can be focused on a specific quasi-energy band ϵ:

$$\begin{aligned} \log e^{-i\epsilon\Lambda + i0^-} &= -i \cdot \epsilon\Lambda \\ \log e^{-i\epsilon\Lambda + i0^+} &= -i \cdot \epsilon\Lambda - 2\pi i \end{aligned}. \quad (39)$$

In particular, we focus on the anomalous energy gap located at $\epsilon = \frac{\pi}{\Lambda}$.

Supplementary Note 20 - Extension to resonator and interferometer platforms.

The polarization-controlled coupling mechanism proposed here is not limited to twisted waveguide arrays, but can be naturally extended to other photonic platforms such as coupled resonator lattices and Mach–Zehnder interferometer (MZI) networks. In resonator systems, one may implement a Möbius-type linking ring with a $\frac{\pi}{2}$ polarization twist per round trip (Supplementary Figure 22a). Such a structure intrinsically supports left- and right-circularly polarized eigenmodes, thereby enabling selective control of topological versus trivial phases by tuning the polarization handedness. In a unit, the coupling strength is equal as k_1, and the coupling strength is k_2 between units. The dynamic phase factor of the half cycle accumulation in the link ring (Möbius) is also considered to be the same, represented by θ', i.e., $\theta' = \theta - \theta'$. For transmission matrix method (TMM), there can be four matrices to express the process

$$\begin{bmatrix} d_i \\ b_i \end{bmatrix} = \begin{bmatrix} [\alpha Y e^{i\theta'}]^{-1} & 0 \\ 0 & \alpha Y e^{i\theta'} \end{bmatrix} \begin{bmatrix} a_i \\ c_i \end{bmatrix}$$

$$\begin{bmatrix} a_i \\ c_i \end{bmatrix} = \begin{bmatrix} M11 & M12 \\ M21 & M22 \end{bmatrix} \begin{bmatrix} h_i \\ f_i \end{bmatrix} \tag{40}$$

$$\begin{bmatrix} h_i \\ f_i \end{bmatrix} = \begin{bmatrix} [\alpha Y e^{i\theta'}]^{-1} & 0 \\ 0 & \alpha Y e^{i\theta'} \end{bmatrix} \begin{bmatrix} e_i \\ g_i \end{bmatrix}$$

$$\begin{bmatrix} e_i \\ g_i \end{bmatrix} = \begin{bmatrix} M11 & M12 \\ M21 & M22 \end{bmatrix} \begin{bmatrix} d_{i+1} \\ b_{i+1} \end{bmatrix}$$

where α represents the intrinsic loss of light propagating half cycle in a link ring (Möbius ring), and the value is the same in the main ring (regular ring). The symbol Y represents the Möbius characteristics, i.e., the twist operation, and it can be written as a matrix of $\frac{\sqrt{2}}{2}\begin{bmatrix} 1 & -1 \\ 1 & 1 \end{bmatrix}$. For matrix $\begin{bmatrix} M11 & M12 \\ M21 & M22 \end{bmatrix}$, it is derived from the coupling relationship in add-drop type micro rings. Considering the coupling relationship with Möbius ring as the link ring in add-drop type micro rings, the coupling coefficient k and transmission coefficient t need to be expressed by matrices.

$$k = \begin{bmatrix} k_1 & 0 \\ 0 & k_1 \end{bmatrix}$$

$$t = \begin{bmatrix} t_1 & 0 \\ 0 & t_1 \end{bmatrix}$$

$$\begin{bmatrix} E_d \\ E_t \end{bmatrix} = \begin{bmatrix} R_x & T_x' \\ T_x & R_x' \end{bmatrix} \begin{bmatrix} E_i \\ E_a \end{bmatrix}$$

$$R_x = -\alpha k_1 Y U^{-1} k_1 e^{i\theta'} \tag{41}$$

$$T_x' = t - \alpha^2 k_1 Y U^{-1} t_1 Y k_1 e^{i\theta}$$

$$T_x = T_x'$$

$$R_x' = R_x$$

$$U = 1 - \alpha^2 t_1 Y t_1 Y e^{i\theta}$$

We can therefore obtain the expression of transmission matrix as

$$\begin{bmatrix} M11 & M12 \\ M21 & M22 \end{bmatrix} = \begin{bmatrix} {R_x}^{-1} & -{R_x}^{-1} * T_x \\ T_x * {R_x}^{-1} & -T_x * {R_x}^{-1} * T_x + {R_x}^{-1} \end{bmatrix}. \tag{42}$$

From the above derivation, we can obtain the total transmission matrix as

$$\begin{bmatrix} d_i \\ b_i \end{bmatrix} = M \begin{bmatrix} d_{i+1} \\ b_{i+1} \end{bmatrix} = e^{-ik_x} \begin{bmatrix} d_{i+1} \\ b_{i+1} \end{bmatrix}. \tag{43}$$

In MZI-based architectures, polarization offers a route to achieve reconfigurable directional coupling. When two waveguides are oriented with orthogonal TE polarizations, they remain decoupled and independently phase-tuned via phase shifters; when rotated into parallel alignment through polarization twisting, they form an

efficient directional coupler. By concatenating orthogonal and parallel segments, joined via twisted waveguide sections, the footprint of each MZI unit can be drastically reduced, offering a powerful strategy for enhancing integration density. These extensions highlight the versatility of polarization-controlled geometric phases for scalable photonic platforms.

Supplementary Note 21 - Possible influence of residual TE-TM coupling.

When the waveguide separation is reduced to 850 or 900 μm, as used in Fig. 4, the increased modal overlap can give rise to weak cross-polarization coupling between the TE and TM modes of adjacent elliptical waveguides. To examine the possible influence of this effect on the propagation dynamics, we extend the TB basis from a single TE mode to both TE and TM modes in each waveguide. This extension is intended as a physically motivated sensitivity analysis of residual TE-TM coupling rather than an exact quantitative description of the full polarization dynamics. Once a finite TM component is excited through inter-waveguide TE-TM coupling, coupling between the TM modes of neighboring waveguides can also contribute to the subsequent evolution. In the parameter range considered here, these additional channels mainly modify the detailed site-resolved intensity distribution while preserving the characteristic propagation behavior of the TE-dominated model. Their influence can be further reduced by optimizing the waveguide geometry, for example by increasing the waveguide separation or adjusting the aspect ratio of the elliptical cross section to suppress TE-TM modal overlap.

The extended model retains the different propagation constants of the two polarizations and includes both same-polarization and cross-polarization nearest-neighbour couplings. With real coupling coefficients, the resulting Hamiltonian is Hermitian and therefore conserves the total modal power during propagation. The present Hamiltonian includes inter-waveguide TE-TM coupling but no on-site TE-TM conversion within an individual twisted waveguide. Such intra-waveguide conversion is sufficiently weak to be neglected under the parameters considered here, as shown in Supplementary Supplementary Figure 36.

For two adjacent waveguides connected by a bond $b \in \{u, v\}$, the previous TE-only model can be written as

$$i\frac{d}{dz}\begin{pmatrix} a_{1,TE} \\ a_{2,TE} \end{pmatrix} = H_{TE}^{(b)}(z)\begin{pmatrix} a_{1,TE} \\ a_{2,TE} \end{pmatrix}, \qquad H_{TE}^{(b)}(z) = \begin{pmatrix} \beta_{TE} & \kappa_{TE}^{(b)}(z) \\ \kappa_{TE}^{(b)}(z) & \beta_{TE} \end{pmatrix}. \tag{44}$$

Defining $\theta(z) = 2\pi z/\Lambda$, the two alternating nearest-neighbour bonds are described by

$$\kappa_{TE}^{(u)}(z) = \kappa_{TE,0}\cos\theta(z), \qquad \kappa_{TE}^{(v)}(z) = \kappa_{TE,0}sin\theta(z). \tag{45}$$

In the extended model, we use the basis

$$\Psi = (a_{1,TE}, a_{2,TE}, a_{1,TM}, a_{2,TM})^T, \tag{46}$$

and the Hamiltonian for either bond takes the form

$$H^{(b)}(z) = \begin{pmatrix} \beta_{TE} & \kappa_{TE}^{(b)}(z) & 0 & \kappa_{TE-TM}^{(b)}(z) \\ \kappa_{TE}^{(b)}(z) & \beta_{TE} & \kappa_{TE-TM}^{(b)}(z) & 0 \\ 0 & \kappa_{TE-TM}^{(b)}(z) & \beta_{TM} & \kappa_{TM}^{(b)}(z) \\ \kappa_{TE-TM}^{(b)}(z) & 0 & \kappa_{TM}^{(b)}(z) & \beta_{TM} \end{pmatrix}. \tag{47}$$

Here, β_{TE} and β_{TM} are the propagation constants of the two guided polarizations. The longitudinally modulated coupling coefficients are chosen as

$$\kappa_{TE}^{(u)}(z) = \kappa_{TE,0}\cos(\theta(z)), \qquad \kappa_{TE}^{(v)}(z) = \kappa_{TE,0}\sin(\theta(z)).$$

$$\kappa_{TM}^{(u)}(z) = \kappa_{TM,0}\cos(\theta(z)), \qquad \kappa_{TM}^{(v)}(z) = \kappa_{TM,0}\sin(\theta(z)). \tag{48}$$

$$\kappa_{TE-TM}^{(u)}(z) = \kappa_{TE-TM,0}\sin\theta((z)), \qquad \kappa_{TE-TM}^{(v)}(z) = \kappa_{TE-TM,0}\cos(\theta(z)).$$

The waveguide-resolved detected intensity is then written as

$$I_n(z) = \left|a_{n,TE}(z)\right|^2 + \left|a_{n,TM}(z)\right|^2. \tag{49}$$

The influence of this additional polarization channel is further reduced by the propagation-constant mismatch between the TE and TM modes. After subtracting a common propagation constant, the two polarization sectors are separated by $\Delta\beta = \beta_{TM} - \beta_{TE}$, which acts as a detuning in the coupled-mode Hamiltonian. When this detuning is large compared with the cross-polarization coupling and is away from a Floquet-assisted resonance, TE-TM transfer is suppressed and the TM sector contributes primarily as a perturbative correction to the TE-dominated dynamics. Consistent with this picture, the recalculated dynamics retain the characteristic period-Λ and period-2Λ behavior while exhibiting changes in the detailed site-resolved

intensities. We therefore use the extended two-polarization model to assess how residual TE-TM coupling may affect the calculated propagation pattern, rather than to provide an exact quantitative description of the full polarization dynamics.

Supplementary Note 22 - Refractive-index correction and quantitative comparison.

The refractive index measured from the bulk resin is used as the initial value in the FDTD simulations. However, the refractive index of the fabricated waveguides can be slightly different from this value. A small offset in the refractive index used in the simulation changes the inter-waveguide coupling strength. Over a long propagation distance, this small difference can lead to a noticeable change in the output intensity distribution.

To determine the refractive-index correction independently of the array measurements in Figs. 3 and 4, we use a separate 1-cm-long straight two-waveguide coupling sample with a center-to-center separation of 900 μm. The incident wavelength is scanned from 1100 to 1300 μm. At $\lambda = 1191$ μm, the normalized power fraction in the initially unexcited waveguide reaches its maximum, corresponding to one complete coupling transfer over the 1-cm propagation length. We then perform FDTD simulations of the same two-waveguide structure while changing only the refractive index of the resin and keeping all geometrical parameters unchanged. A correction of $\Delta n = 0.02$ reproduces the experimentally observed transfer maximum at $\lambda = 1191$ μm. We therefore use $\Delta n = 0.02$ as a single global refractive-index correction in the subsequent simulations. This correction corrects the small difference between the refractive index used in the original simulation and that corresponding to the fabricated waveguides. After $\Delta n = 0.02$ is determined, the same corrected refractive index is used for all wavelengths, propagation distances, and array configurations without further adjustment.

Supplementary Figure 28 compares the corrected FDTD results with the experimental measurements for the straight-waveguide control array at wavelengths of 1300, 1250, and 1200 μm. The corrected simulations reproduce the main wavelength-

dependent redistribution of the optical intensity observed experimentally. The corresponding quantitative comparisons are summarized in Supplementary Table 2. For the straight-waveguide control sample at 1200 μm, the mean normalized squared Euclidean distance decreases from 0.177 before correction to 0.047 after correction, while the mean distribution fidelity increases from 0.862 to 0.943. With the same correction kept fixed, the mean distribution fidelities at 1250 and 1300 μm are 0.949 and 0.958, respectively. These results show that the global refractive-index correction improves the overall agreement between the FDTD simulations and the experimental measurements.

For the Floquet-modulated arrays, the influence of this correction is smaller. In the ideal nearest-neighbor model, the coupling coefficient has the form $\kappa(\lambda, z) = \kappa_0(\lambda) cos(\theta(z) + \theta_0)$, and its integral over one complete driving period is zero. Therefore, a small change in the overall coupling strength $\kappa_0(\lambda)$ does not remove the characteristic spreading-and-refocusing behavior. This explains why the corrected FDTD results for the modulated arrays change less than those of the straight-waveguide control sample.

Supplementary Note 23 - Experimental validation of local polarization-controlled coupling.

Supplementary Figure 30 compares two two-waveguide coupling units. In the first structure, the longitudinal coupling follows the sign-changing function

$$\kappa(z) = \kappa_0 \cos(\theta(z)), \tag{50}$$

whereas in the reference structure it follows

$$\kappa(z) = \kappa_0 |\cos(\theta(z))|. \tag{51}$$

The two structures therefore have the same coupling magnitude $|\kappa(z)|$ at every propagation position but differ in whether the coupling sign reverses. The TB and FDTD simulations predict clearly different propagation dynamics for the two cases, and the experimentally measured output distributions reproduce this difference. This comparison directly verifies that the longitudinal sign reversal of the coupling has an observable physical effect on the propagation dynamics.

We further extend this validation to the four-site, three-arm unit shown in Supplementary Figure 31. A central waveguide 4 is coupled to three surrounding waveguides 1, 2, and 3, oriented along three in-plane directions separated by $\frac{2\pi}{3}$. For the sign-changing configuration, the three couplings are $\kappa_1(z) = \kappa_0\cos(\theta(z) + \theta_0)$, $\kappa_2(z) = \kappa_0\cos(\theta(z) + \theta_0 - \frac{2\pi}{3})$, and $\kappa_3(z) = \kappa_0\cos(\theta(z) + \theta_0 - \frac{4\pi}{3})$, whereas the reference configuration uses the corresponding unsigned couplings $\kappa_1(z) = \kappa_0|\cos(\theta(z) + \theta_0)|$, $\kappa_2(z) = \kappa_0\left|\cos(\theta(z) + \theta_0 - \frac{2\pi}{3})\right|$, and $\kappa_3(z) = \kappa_0\left|\cos(\theta(z) + \theta_0 - \frac{4\pi}{3})\right|$. The two configurations retain the same coupling-magnitude modulation while differing in the coupling signs. Their FDTD simulations predict distinct output intensity distributions, and the experimental measurements reproduce the main contrast in the relative intensities among the four waveguides.

The quantitative comparisons for these local coupling units are summarized in Supplementary Table 2. For the two-waveguide unit, the distribution fidelities are 0.9994 and 0.9997 for the sign-changing and unsigned configurations, respectively. For the four-site unit, the corresponding fidelities are 0.9868 and 0.9928. These measurements experimentally validate the local polarization-controlled coupling mechanisms required for the proposed two-dimensional implementation.

Supplementary Note 24 - Possible lower-loss implementations.

The relatively high absorption of the RG resin limits the propagation distance accessible in the present experiments. We therefore consider two possible approaches for reducing the propagation loss while retaining the geometric flexibility required for polarization-controlled coupling.

One possible approach is to introduce subwavelength air holes into the resin waveguide cross-section. As shown in Supplementary Figure 32, four symmetrically arranged air holes reduce the modal overlap with the absorbing resin material. The FDTD simulations show that the guided field remains predominantly polarized along the major axis of the elliptical waveguide, while the propagation loss is reduced

compared with that of the corresponding resin waveguide without air holes. At 0.26 THz and after 4 cm of propagation, the estimated propagation loss is approximately 34.0 dB for the RG-resin nonporous waveguide and approximately 9.90 dB for the porous waveguide. This design therefore provides a possible route to reducing material-induced attenuation while preserving the polarization properties required by the present coupling mechanism.

A second possible approach is to replace the RG resin with a lower-loss material compatible with high-precision 3D printing. Figure S33 compares the measured optical properties of alumina ceramic and RG resin. Over the measured terahertz frequency range, the absorption coefficient of the alumina ceramic remains lower than that of the RG resin. At 0.26 THz and after 4 cm of propagation, the estimated propagation loss is approximately 12.2 dB for the alumina-ceramic waveguide, compared with approximately 34.0 dB for the RG-resin waveguide. These results indicate that 3D-printed alumina ceramic may provide another possible route toward longer-distance implementations of polarization-controlled Floquet waveguide arrays.

These structures are presented as possible future implementations and further optimization of the waveguide geometry, material processing, and fabrication parameters would be required for a full experimental realization.

Supplementary Note 25 - Experimental repeatability and possible sources of the remaining discrepancies.

To examine the repeatability of the measured output distributions, we perform five repeated measurements for two representative samples at the maximum propagation distance of $z = 4$ cm: the straight-waveguide control array and the Floquet-modulated array in Fig. 3b. As shown in Supplementary Figure 34, the repeated measurements reproduce the main site-resolved intensity distributions in both samples. The error bars represent one standard deviation over the five measurements. The standard deviations are substantially smaller than the intensity differences between the output channels, indicating that the measured spatial features are reproducible

Several experimental factors can nevertheless contribute to the remaining site-resolved differences between simulation and experiment. In the VNA measurements, the transmitting and receiving WR-4 metallic waveguides are aligned directly with the dielectric-waveguide facets. Small variations in the probe-to-sample distance and lateral alignment can therefore change the measured intensity. In addition, the input field from the WR-4 waveguide is not identical to the ideal TE eigenmode used in the FDTD simulations, so a small contribution from other mode components may also affect the detailed intensity distribution.

The effective excitation position provides another source of sensitivity. The samples were originally fabricated with horn-shaped input tapers for the THz-TDS measurements. When the measurements were changed to the VNA system, these tapers were mechanically removed to allow direct coupling from the WR-4 metallic waveguide to the dielectric-waveguide facet. This results in a small forward displacement of the effective input facet. Figure S35 compares the TB propagation dynamics before and after this representative shift. For the structures in Fig. 3, the two couplings adjacent to the excited waveguide have the same longitudinal dependence, and the propagation pattern changes only weakly. In Fig. 4, however, the two adjacent couplings follow different sine-like and cosine-like dependences. The shift therefore changes their relative values at the excitation position and produces a more noticeable change in the subsequent propagation dynamics.

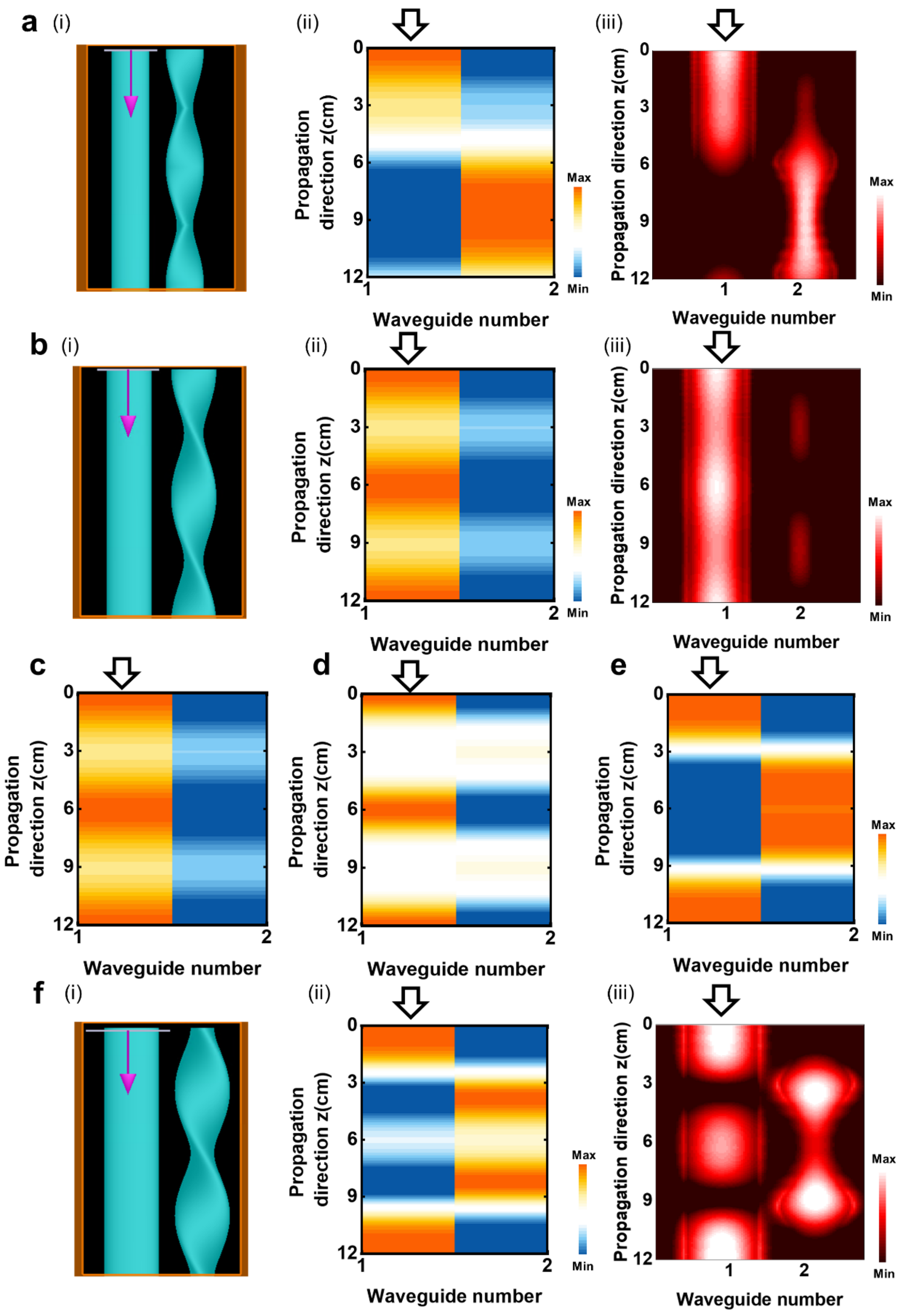

Supplementary Figure 1 | Verification of negative coupling and the roles of its sign and functional form. a, (i) Schematic of the first twisting configuration, where two initially aligned TE-polarized waveguides undergo successive clockwise and counterclockwise $\frac{\pi}{2}$ twists, resulting in an effective coupling coefficient $\kappa(z) = \kappa_0|\cos(\theta(z))|$. (ii) Calculated coupling

distribution and (iii) simulated field evolution over one driving period (Λ = 12 cm). **b**, (i) Schematic of the second configuration, in which the right waveguide is twisted continuously by 2π clockwise, yielding $\kappa(z) = \kappa_0 \cos\big(\theta(z)\big)$. (ii) Corresponding coupling distribution and (iii) simulated field evolution, showing a markedly different behavior from a despite identical structural parameters (waveguide spacing d = 1000 μm, semi-major axis a = 350 μm, semi-minor axis b = 150 μm). **c**, Numerical verification of the equivalence between $\kappa(z) = \kappa_0 \cos\big(\theta(z)\big)$ and $\kappa'(z) = \kappa_0 |\cos\big(\theta(z)\big)|$, where the negative sign is absorbed into the state vector component, effectively corresponding to the addition of a negative on-site potential. **d**, Numerical state evolution of coupling coefficient $\kappa(z) = \begin{cases} \kappa_0 \frac{\frac{\Lambda}{4} - z}{\frac{\Lambda}{4}} & if\ 0 < z < \frac{\Lambda}{2} \\ \kappa_0 \frac{z - \frac{3\Lambda}{4}}{\frac{\Lambda}{4}} & if\ \frac{\Lambda}{2} < z < \Lambda \end{cases}$. **e**, Numerical state evolution of coupling coefficient $\kappa(z) = \begin{cases} \kappa_0 \frac{z}{\frac{\Lambda}{4}} & if\ 0 < z < \frac{\Lambda}{4} \\ \kappa_0 \frac{\frac{\Lambda}{2} - z}{\frac{\Lambda}{4}} & if\ \frac{\Lambda}{4} < z < \frac{3\Lambda}{4} \\ \kappa_0 \frac{z - \Lambda}{\frac{\Lambda}{4}} & if\ \frac{3\Lambda}{4} < z < \Lambda \end{cases}$. **f**, (i) Schematic of the twisting configuration, where the major axes of the elliptical cross-sections at the initial positions of the two waveguides are perpendicular to each other, and the right waveguide is twisted continuously by 2π clockwise, resulting in an effective coupling coefficient $\kappa(z) = \kappa_0 \sin\big(\theta(z)\big)$. (ii) Calculated coupling distribution and (iii) simulated field evolution over one driving period (Λ = 12 cm).

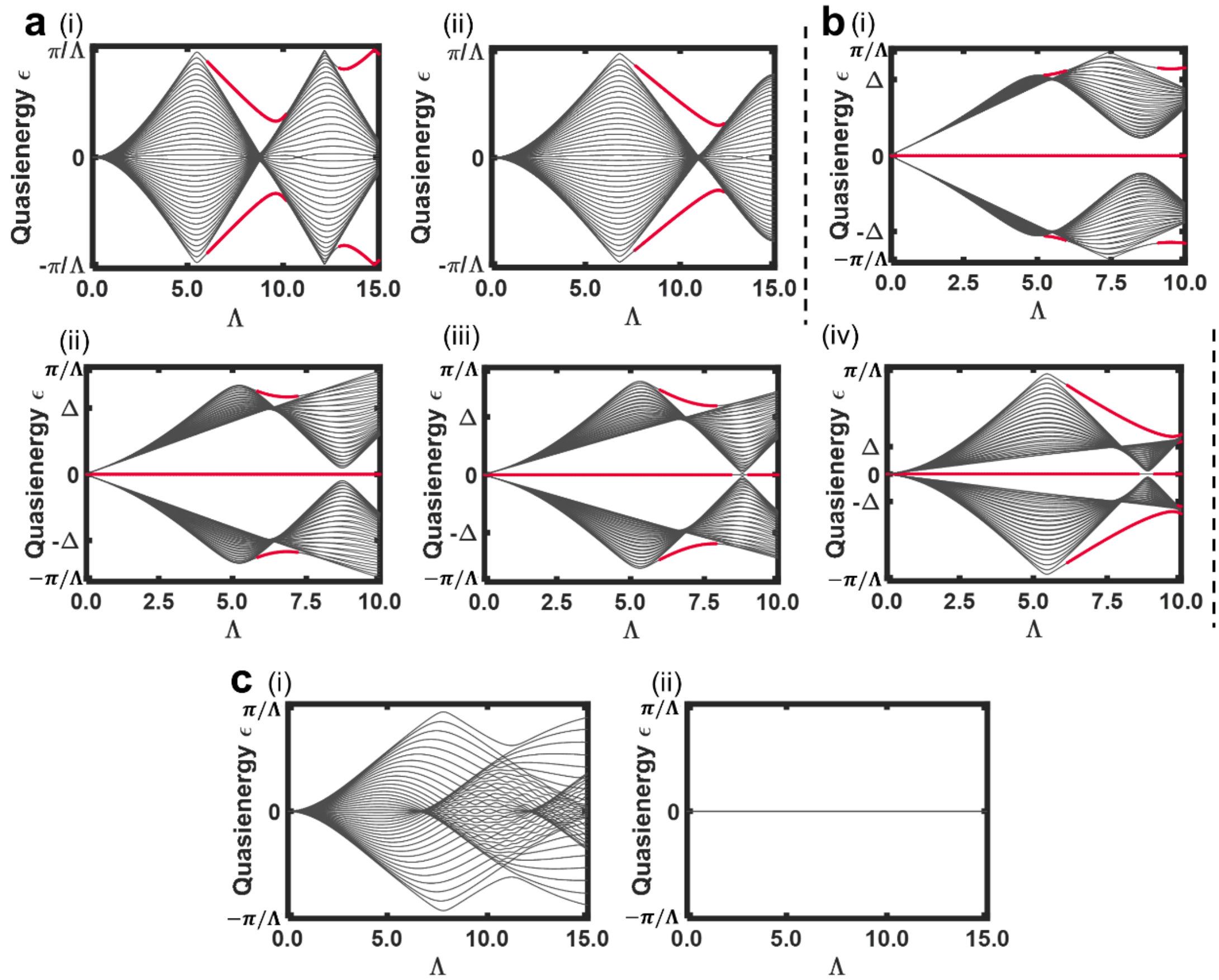

Supplementary Figure 2 | Supplementary results on flat-band quasi-energy in other structures. a, Quasi-energy bands of an array with 80 waveguides as a function of the driving period Λ, for (**i**) sinusoidal coupling modulation, and (**ii**) triangular-wave coupling modulation. **b**, Quasi-energy bands of an array with 80 waveguides as a function of the driving period Λ, for different modulation depths: (**i**) $\delta\Lambda = \frac{\Lambda}{3}$, (**ii**) $\delta\Lambda = \frac{\Lambda}{4}$, (**iii**) $\delta\Lambda = \frac{\Lambda}{5}$, and (**iv**) $\delta\Lambda = \frac{\Lambda}{12}$. **c**, Quasi-energy bands under two coupling configurations with 80 waveguides: (**i**) $\kappa_1(z) = \kappa_0 \cos(\theta(z))$ and $\kappa_2(z) = \kappa_0 \cos(\theta(z) + \frac{\pi}{4})$, (**ii**) $\kappa_1(z) = \kappa_0 \cos(\theta(z))$ and $\kappa_2(z) = \kappa_0 \cos(\theta(z))$.

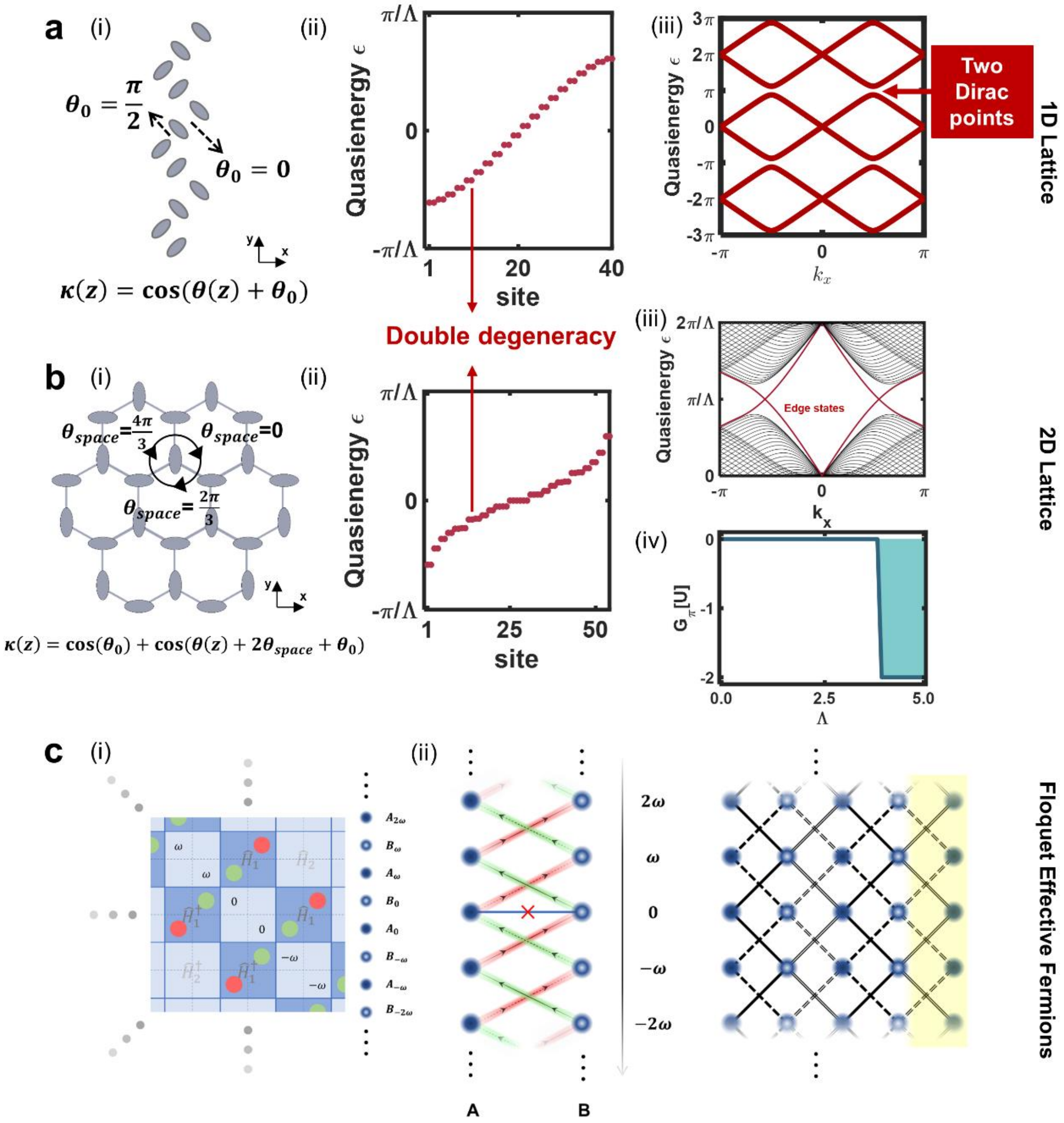

Supplementary Figure 3 | Effective Floquet fermions under extended Floquet Hamiltonian. a, One-dimensional lattice. (**i**) Schematic of the 1D two-site unit cell with couplings defined as $\kappa(z) = \kappa_0 \cos(\theta(z) + \theta_0)$. (**ii**) Quasi-energy spectrum under OBCs, showing a global twofold degeneracy. (**iii**) Quasi-energy band structure in momentum space, exhibiting two Dirac points. **b**, Two-dimensional lattice. (**i**) Schematic of the 2D two-site unit cell with couplings $\kappa(z) = \cos(\theta_0) + \cos(\theta(z) + 2\theta_{space} + \theta_0)$. (**ii**) Quasi-energy spectrum under OBCs, also displaying twofold degeneracy. (**iii**) Band structure in momentum space with edge states inside the quasi-energy gap. (**iv**) Topological phase diagram obtained from the winding number as a function of the driving period Λ. **c**, Effective Floquet fermion representation. (**i**) Extended Floquet Hamiltonian expressed in Fourier space. (**ii**) Mapping to

two equivalent and independent atomic chains in momentum space, where the absence of a zero-frequency component forbids intra-harmonic couplings (red cross). (**iii**) Construction of the corresponding Floquet fermion lattice in real space, consisting of two independent sublattices (solid and dashed).

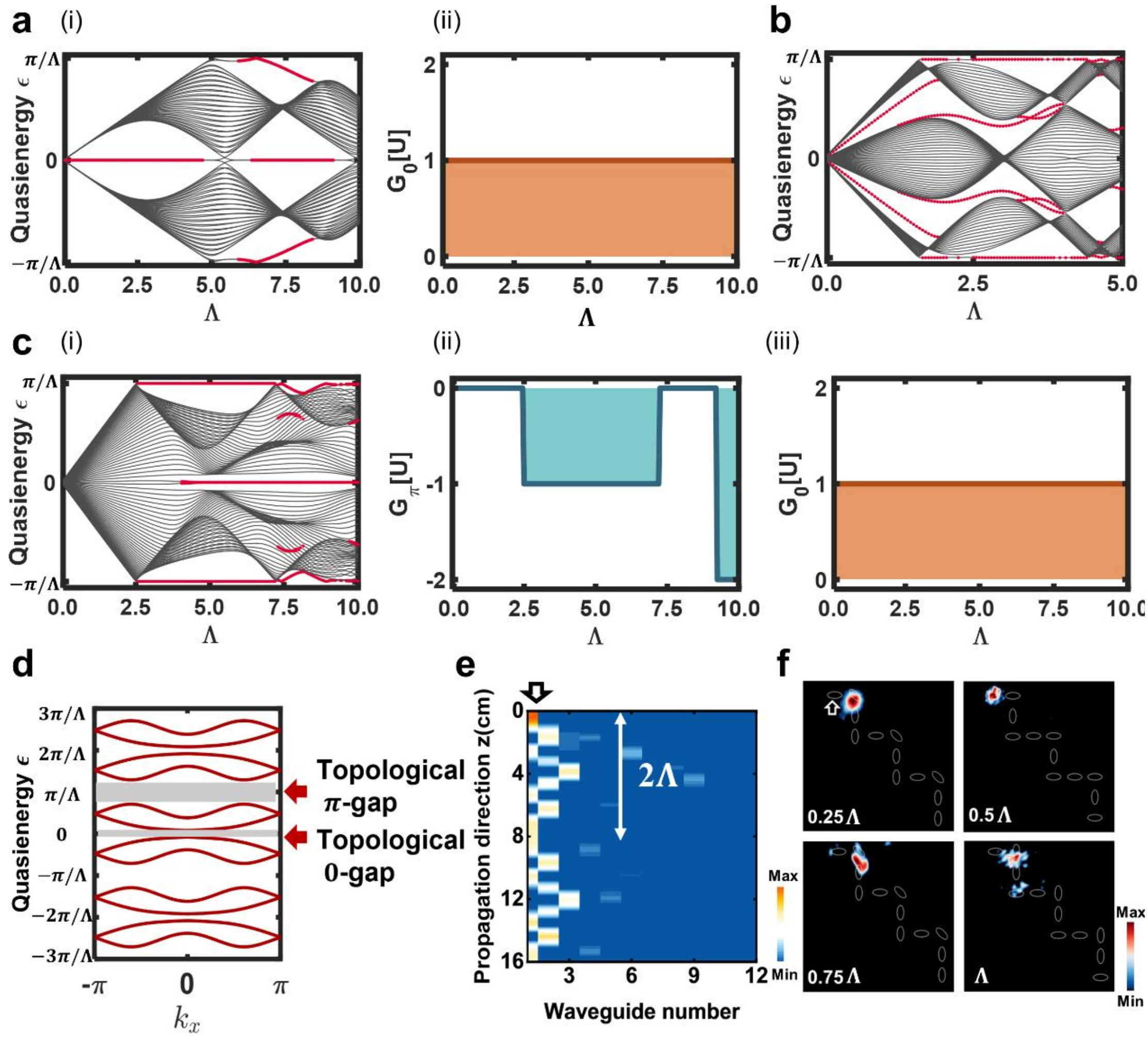

Supplementary Figure 4 | Supplementary results for the topological state protected by chiral symmetry. **a**, Quasi-energy bands of an array with 80 waveguides as a function of the driving period Λ for $\kappa_1(z) = \kappa_0\cos(\theta(z))$ and $\kappa_2(z) = \kappa_0|\sin(\theta(z))|$, together with the associated topological invariant for the zero gap. **b**, Quasi-energy bands of an array with 80 waveguides for $\kappa_1(z) = \kappa_0|\sin(\theta(z))|$, $\kappa_2(z) = \kappa_0|\cos(\theta(z))|$, $\kappa_3(z) = 2\kappa_0|\sin(\theta(z))|$, and $\kappa_4(z) = 2\kappa_0|\cos(\theta(z))|$. **c**, Quasi-energy bands of the array for $\kappa_1(z) = \kappa_0|\sin(\theta(z))|$, $\kappa_2(z) = \kappa_0|\cos(\theta(z))|$, $\kappa_3(z) = \kappa_0|\sin(2\theta(z))|$, and $\kappa_4(z) = \kappa_0|\cos(2\theta(z))|$, accompanied by the corresponding topological invariant for the π gap (**i**) and that for the zero gap (**ii**). **d**, Quasi-energy bands in momentum space for the same structure as in **c**, showing that both the π and zero gaps are topological. **e**, Numerically theoretical evolution of light intensity under central site excitation, showing recurrence at every 2Λ. **f**, Experimental results at 0.25Λ, 0.5Λ, 0.75Λ, and Λ propagation, showing that the edge state does not return to its initial position after one period, in agreement with theory.

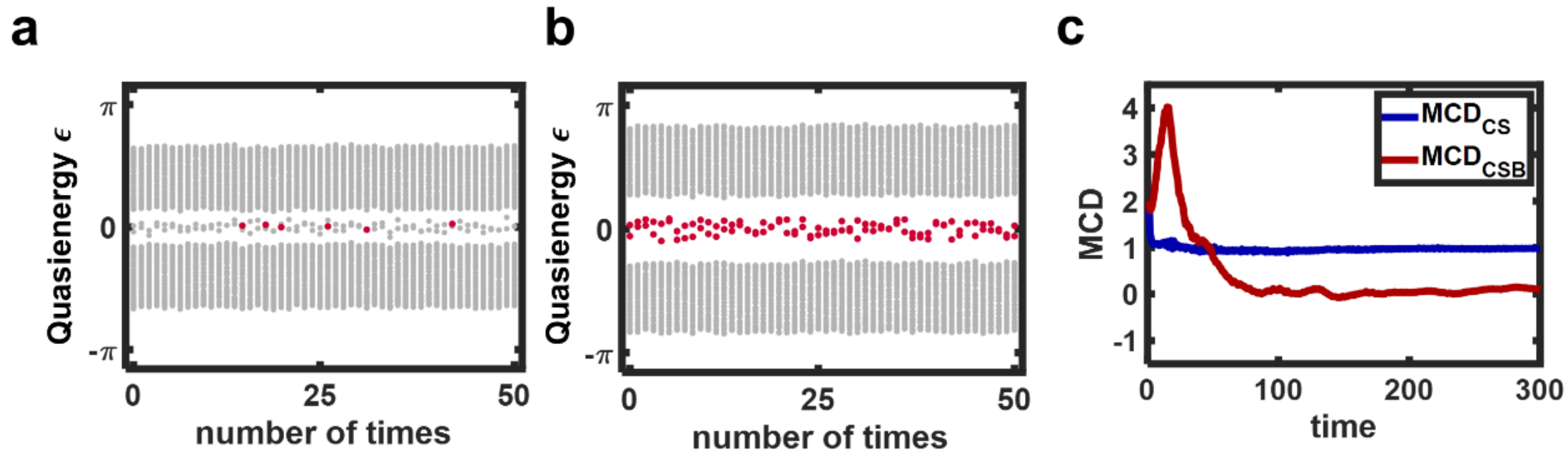

Supplementary Figure 5 | Robustness of topological states under disorder. a, In the absence of chiral symmetry protection, we introduce random perturbations to the coupling coefficients and perform 50 realizations. The spectral results reveal that edge states within the zero-energy gap are largely destroyed. **b**, When chiral symmetry is preserved, edge states persist in all 50 disorder realizations, demonstrating their topological robustness. **c**, The integrated mean chiral displacement (IMCD) is calculated for the two cases. For the chiral-symmetry-broken system, IMCD converges to 0 with increasing integration time, whereas for the system with chiral-symmetry, IMCD converges to 1, consistent with the quantized topological invariant.

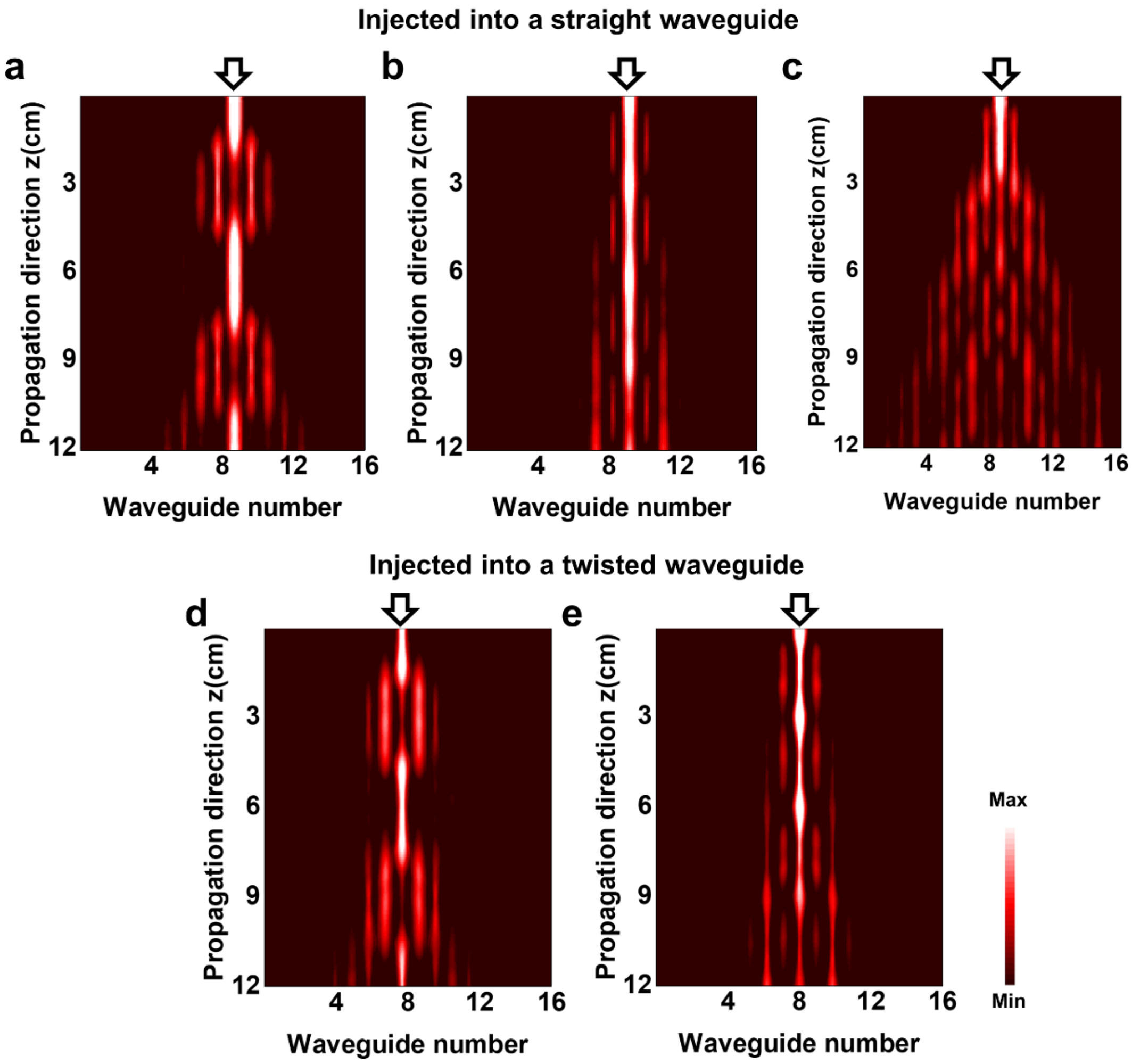

Supplementary Figure 6 | FDTD simulations of Floquet flat-band. a, Numerical propagation in an 80-waveguide array with a coupling coefficient of $\kappa(z) = \kappa_0 \sin(\theta(z))$ and a Floquet driving period of $\Lambda = 6$ cm. **b**, Numerical propagation in an 80-waveguide array with a coupling coefficient of $\kappa(z) = \kappa_0 \cos(\theta(z))$ and a Floquet driving period of $\Lambda = 6$ cm. **c**, Control case without self-imaging function. **d**, Propagation dynamics for the same structure as in **a** but with excitation from a twisted waveguide. **e**, Propagation dynamics for the same structure as in **b** but with excitation from a twisted waveguide. These results demonstrate that propagation is governed by the spatial profile of inter-waveguide coupling rather than the injected site.

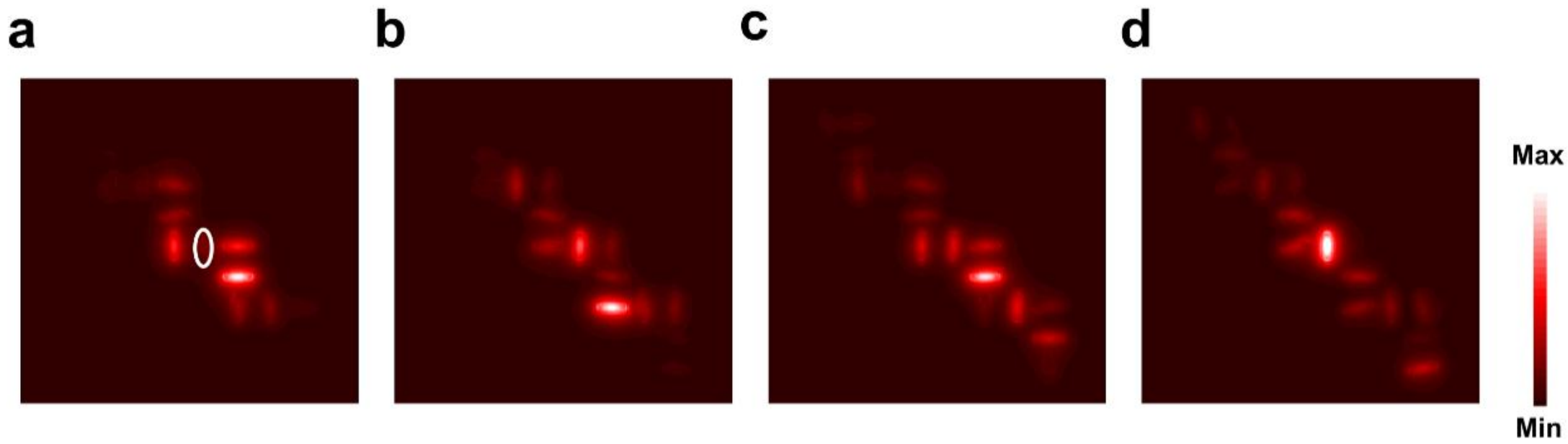

Supplementary Figure 7 | FDTD simulations of period-nΛ oscillation. Simulated results by FDTD method at 0.25Λ (**a**), 0.5Λ (**b**), 0.75Λ (**c**), and Λ (**d**) propagation, showing that the edge state return to its initial position after one period.

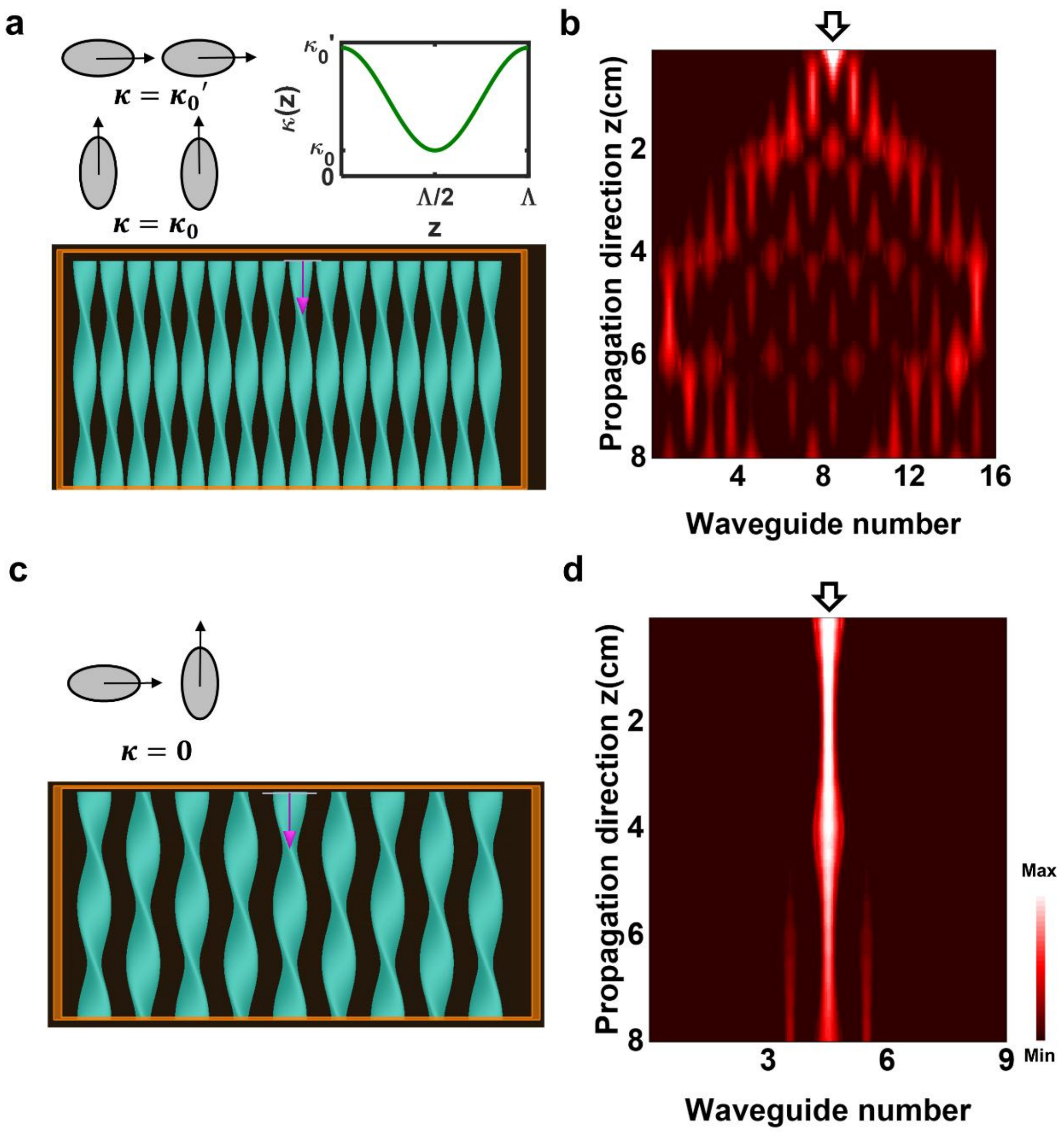

Supplementary Figure 8 | FDTD simulations of coupling modulation via uniform and relative twisting of waveguides. a, Schematic of an array where all waveguides are uniformly twisted, leading to a modification of the coupling coefficient from κ_0 to $\kappa_0{}'$. **b**, Corresponding FDTD simulation results of light propagation in the uniformly twisted array. **c**, Schematic of an array where adjacent waveguides are twisted with an initial relative angle difference of $\frac{\pi}{2}$, theoretically suppressing nearest-neighbor coupling. **d**, FDTD simulation results for the configuration in **c**, confirming the suppression of nearest-neighbor coupling.

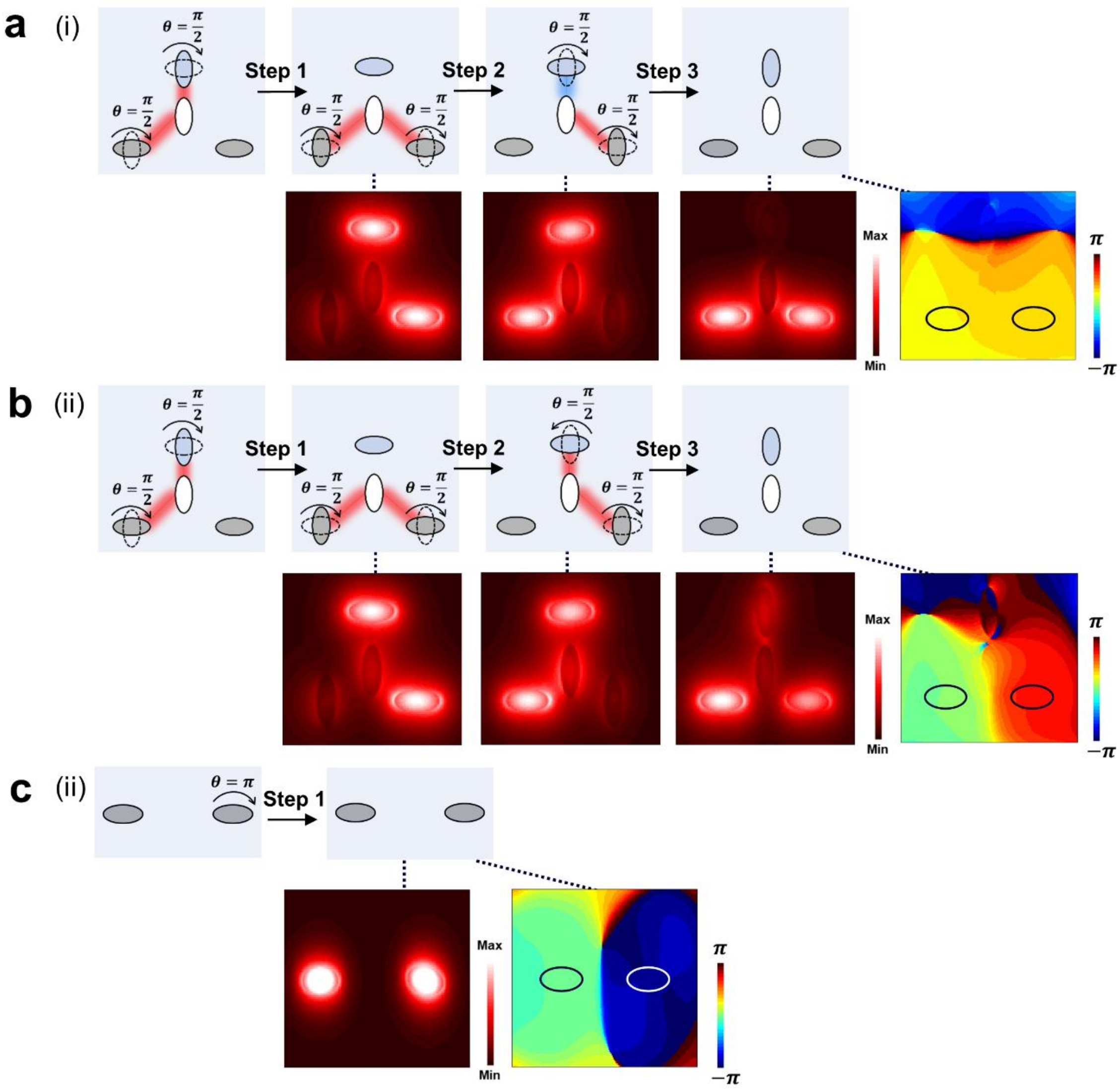

Supplementary Figure 9 | Implementation of non-Abelian operations. a, Realization of the logical X gate. The first row illustrates the structural operations at each step (Step 1–Step 3), while the second row shows the simulated field intensity distributions after Step 1, Step 2, and Step 3, together with the output phase distribution. **b**, Realization of the logical Y gate. The first row depicts the stepwise structural operations, and the second row presents the corresponding field intensity distributions after each step, followed by the output phase distribution. **c**, Realization of the logical Z gate. The first row shows the structural operation at Step 1, and the second row displays the output field intensity and phase distributions.

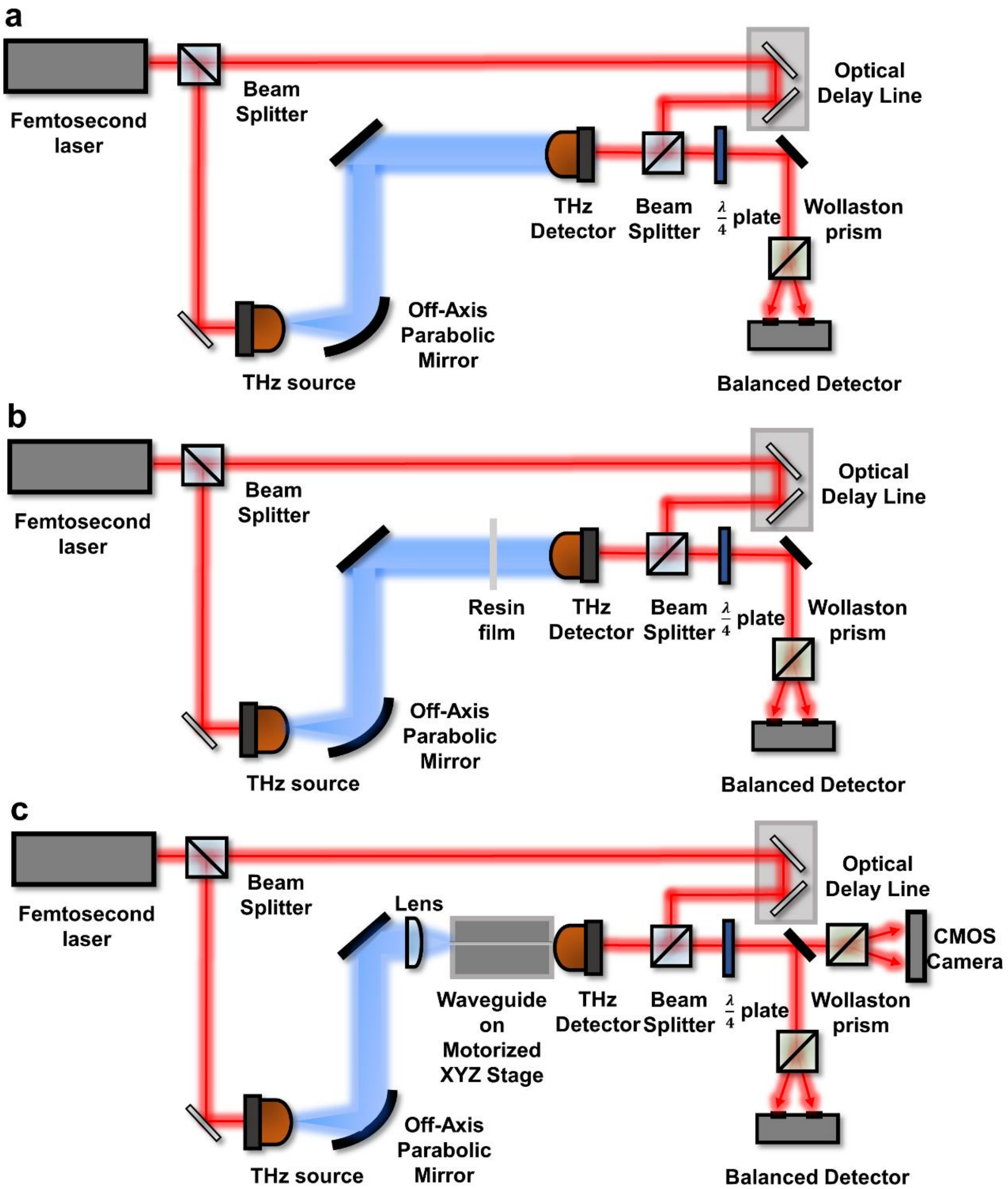

Supplementary Figure 10 | Experimental setups of the terahertz time-domain spectroscopy systems. a–c correspond to systems without samples, a resin film, and a lens coupled to a resin waveguide, respectively.

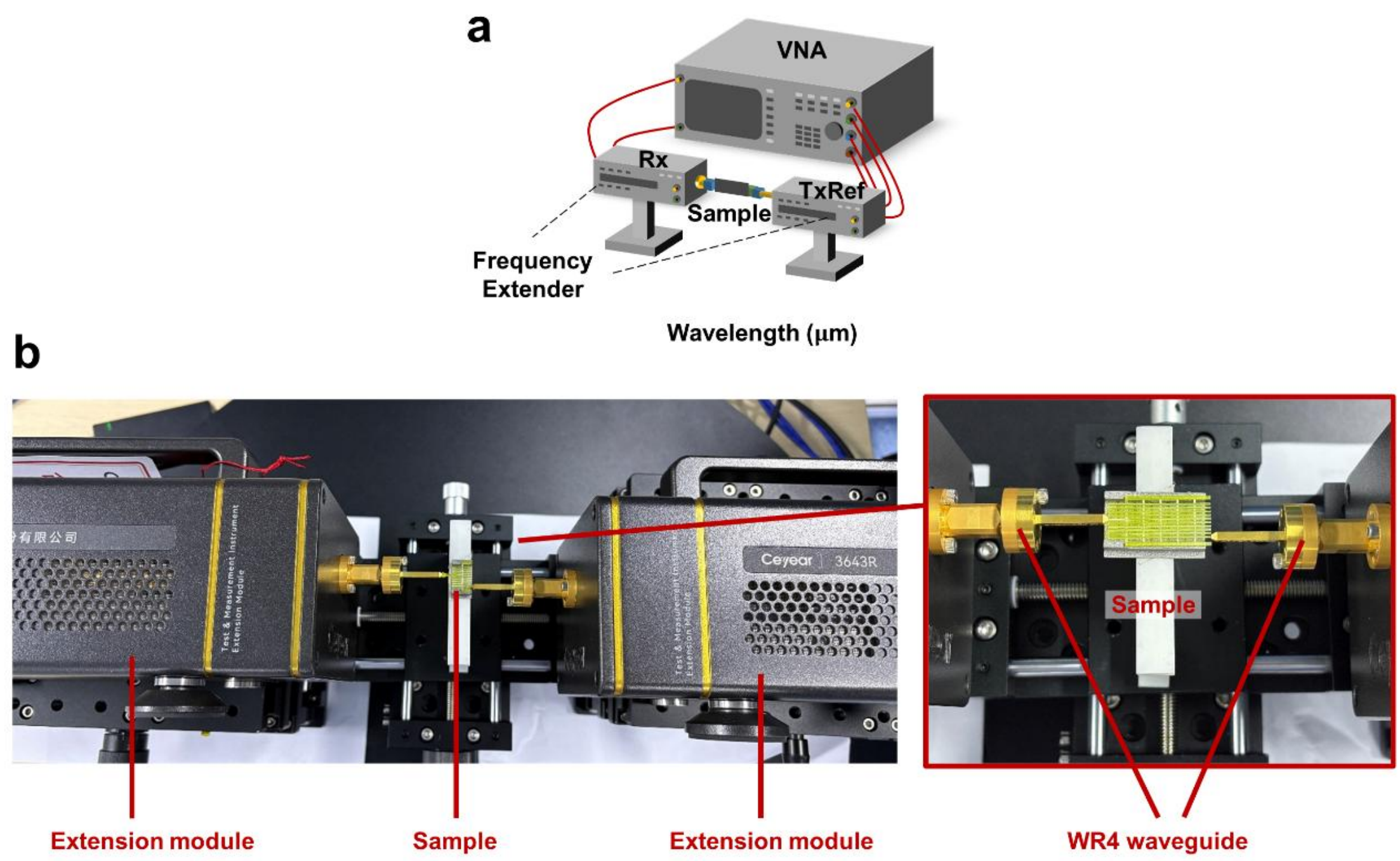

Supplementary Figure 11 | THz-VNA measurement setup for the repeated experiments. a, Schematic of the THz-VNA measurement system, consisting of a VNA, two frequency extension modules, and the sample placed between them. The TxRef module serves as the transmitting and reference port for terahertz signal generation and coherent detection, whereas the Rx module receives the transmitted terahertz signal. **b,** Photograph of the corresponding experimental setup. The sample is mounted between the transmitting and receiving extension modules, and the inset shows an enlarged view of the sample region. Two WR4 metallic waveguides are connected to the sample to couple the terahertz signal into the structure and collect the transmitted output.

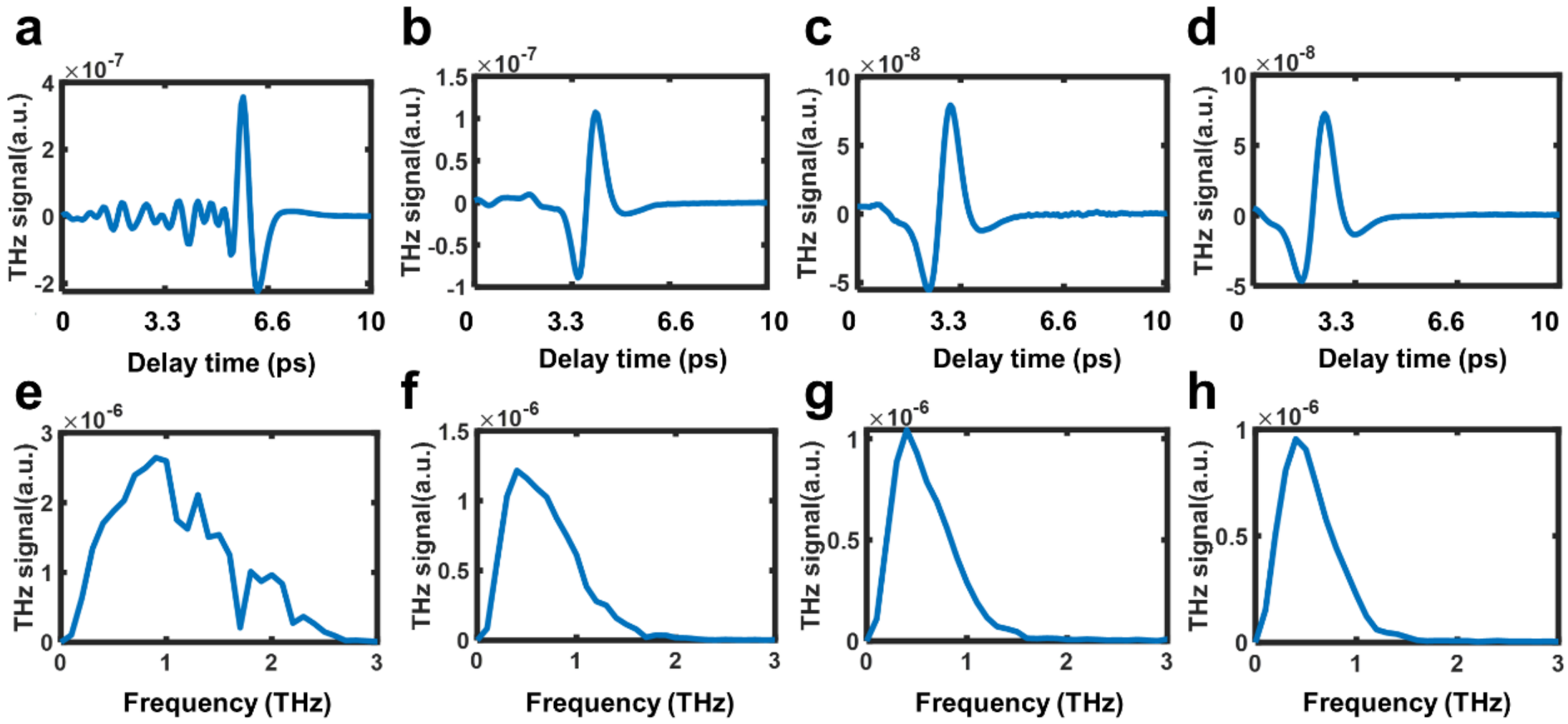

Supplementary Figure 12 | Terahertz time-domain spectroscopy of air and RG resin films with different thicknesses. a–d, Time-domain signals measured in air and through RG resin films with thicknesses of 1 mm, 1.5 mm, and 2 mm, respectively. **e–h**, Corresponding frequency-domain spectra obtained from the same measurements for air and for RG resin films of 1 mm, 1.5 mm, and 2 mm thickness.

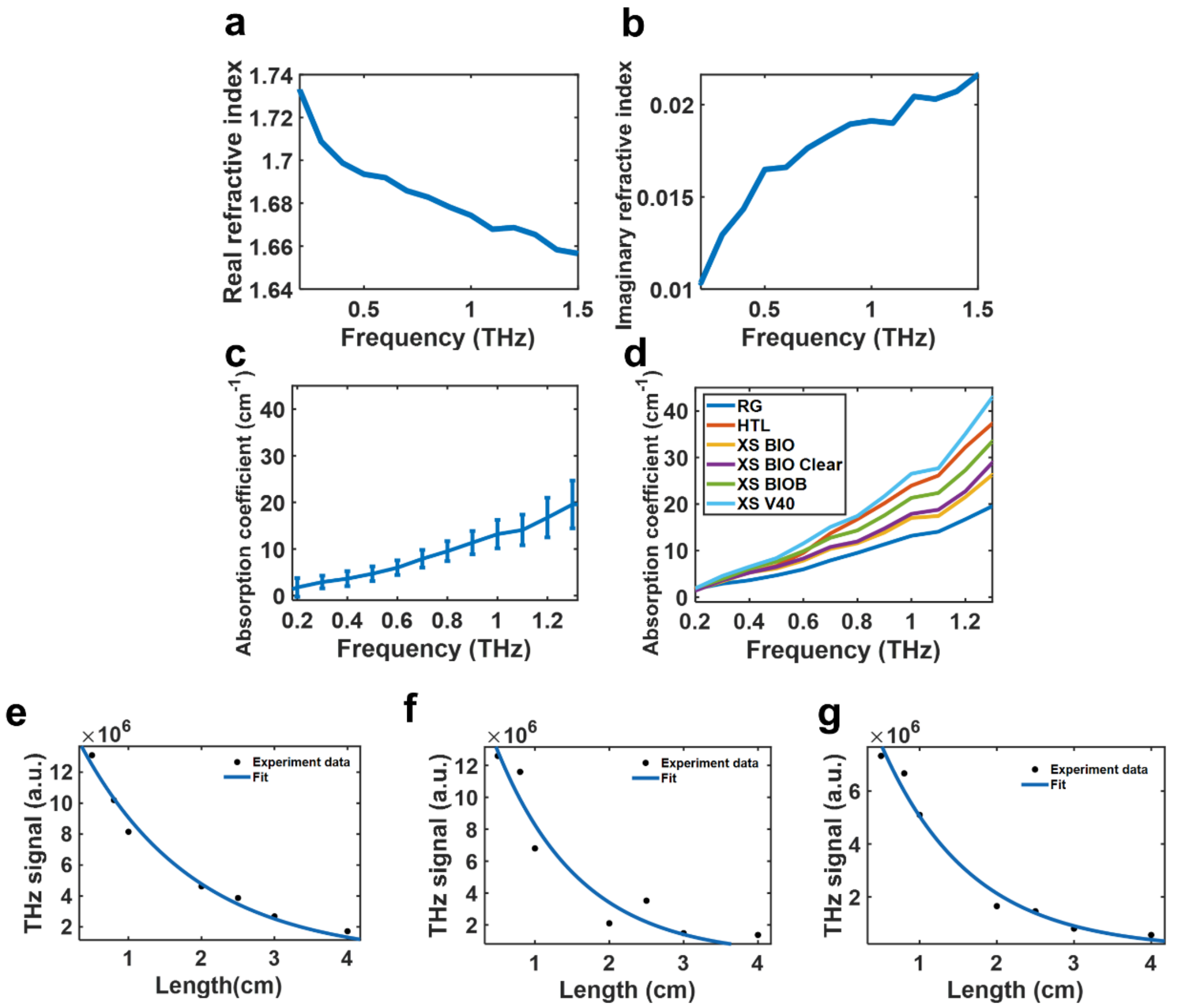

Supplementary Figure 13 | Terahertz characterization of RG resin films and elliptical straight waveguides. a, Real part of the refractive index of a 1-mm-thick RG resin film obtained from terahertz time-domain spectroscopy. **b**, Imaginary part of the refractive index of the same film. **c**, Absorption coefficient of the RG resin extracted from the THz-TDS measurements, showing values consistent with previously reported absorption coefficients of resin-based materials in the terahertz band[11]. **d**, Comparison of the absorption coefficients of several candidate resins measured by THz-TDS at different frequencies, showing that RG exhibits the lowest absorption among the tested materials and is therefore selected for sample fabrication. e–**g**, Measured output signals of elliptical waveguides with cross-sectional axes $a = 350$ μm and $b = 150$ μm at different propagation lengths. The corresponding absorption coefficients are 1.28 cm^{-1} (5.56 dB/cm), 1.598 cm^{-1} (6.94 dB/cm), and 1.718 cm^{-1} (7.46 dB/cm) for panels **e-g**, corresponding to frequencies of 0.2 THz, 0.25 THz, and 0.3 THz, respectively.

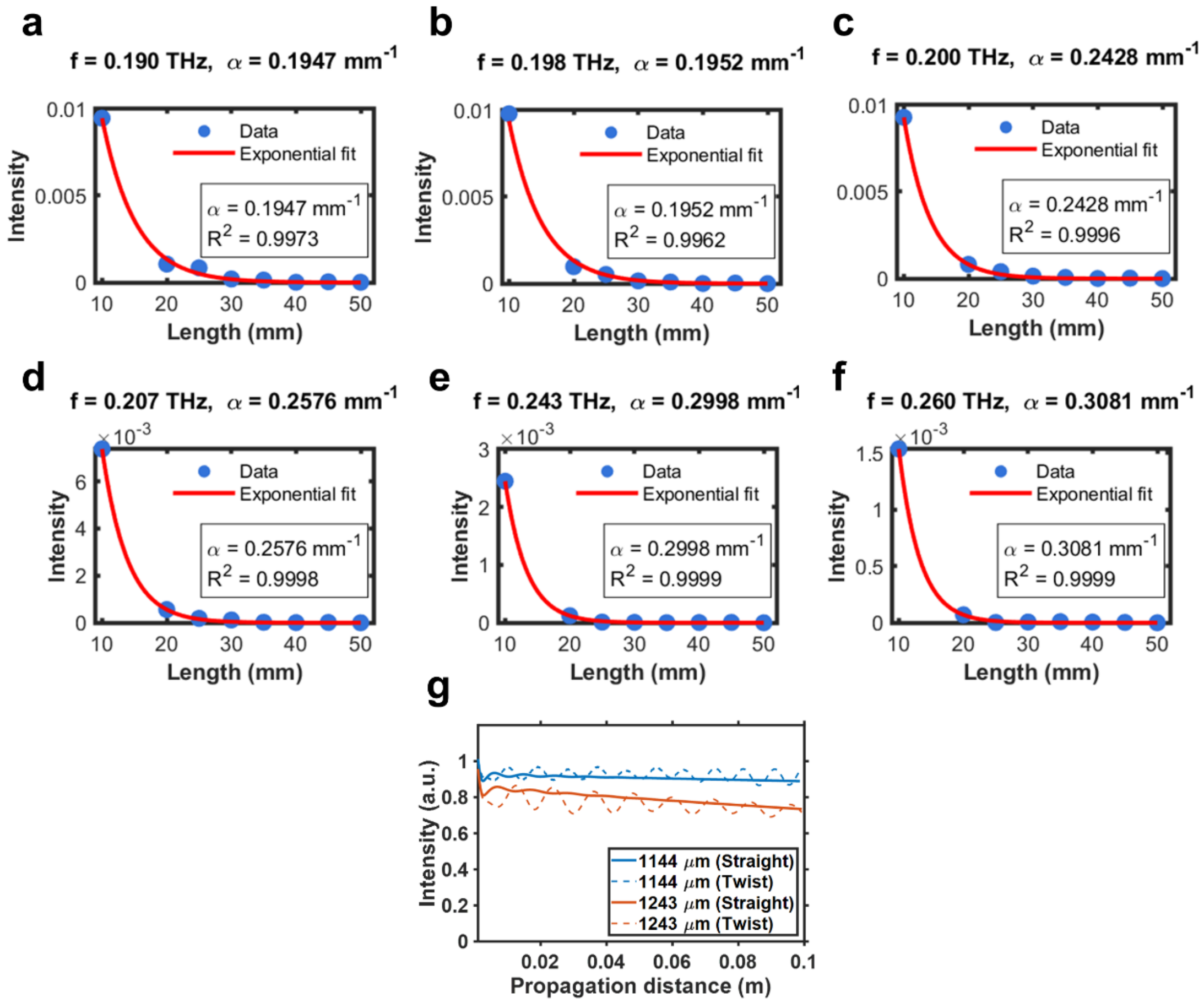

Supplementary Figure 14 | Loss characterization of twisted elliptical waveguides and comparison with straight waveguides. a–f, Measured transmitted intensities of twisted elliptical waveguides with different propagation lengths, obtained using the THz-VNA system at different frequencies. Exponential fits give the corresponding absorption coefficients. The fitted values are close to those obtained for straight waveguides, indicating that the twist does not introduce significant additional loss. The slightly higher loss in the twisted samples likely arises from increased surface roughness introduced during high-precision 3D printing of the twisted structures. **g,** FDTD-simulated intensity evolution in straight and twisted waveguides at two representative wavelengths. The simulations show that the twist introduces nearly no additional loss. The stronger absorption at longer wavelengths mainly results from weaker field confinement in the waveguide. Together with the material characterization, these results show that although the absorption coefficient of the bulk resin film increases with frequency, the effective loss in a specific elliptical waveguide also depends on the field confinement in the

waveguide, so no simple monotonic relation between loss and frequency can be directly inferred.

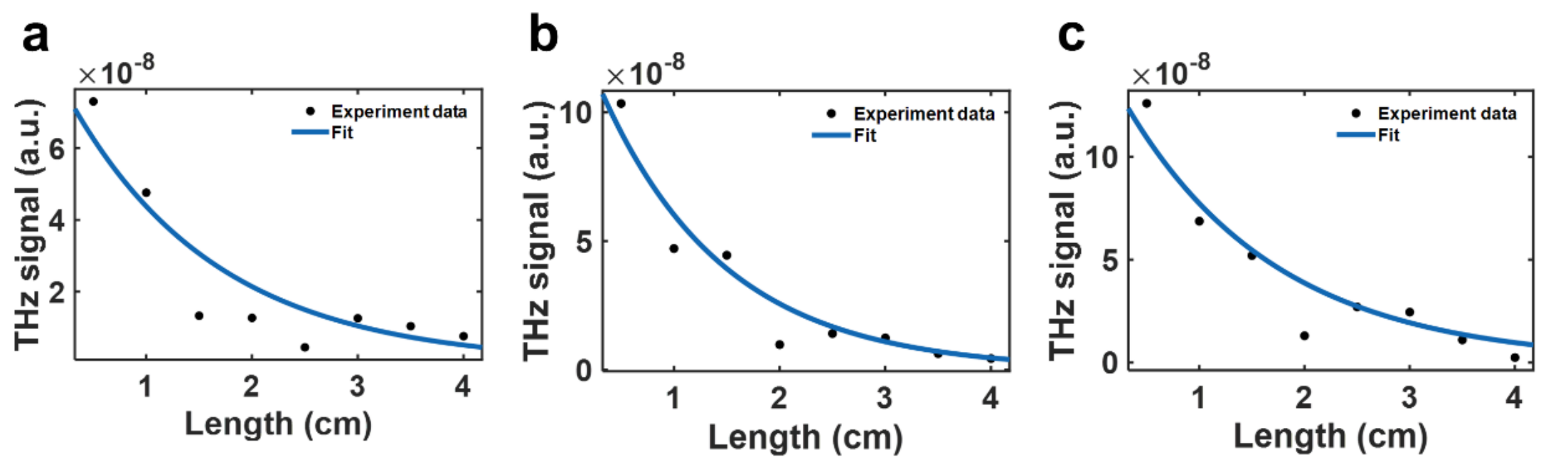

Supplementary Figure 15 | Measured transmitted intensity as a function of metallic hollow elliptical waveguide length. a–c, Measured attenuation coefficients of metallic hollow elliptical waveguides with cross-sectional axes $a = 450$ μm and $b = 250$ μm at different propagation lengths, corresponding to frequencies of 0.4 THz, 0.5 THz, and 0.6 THz, respectively.

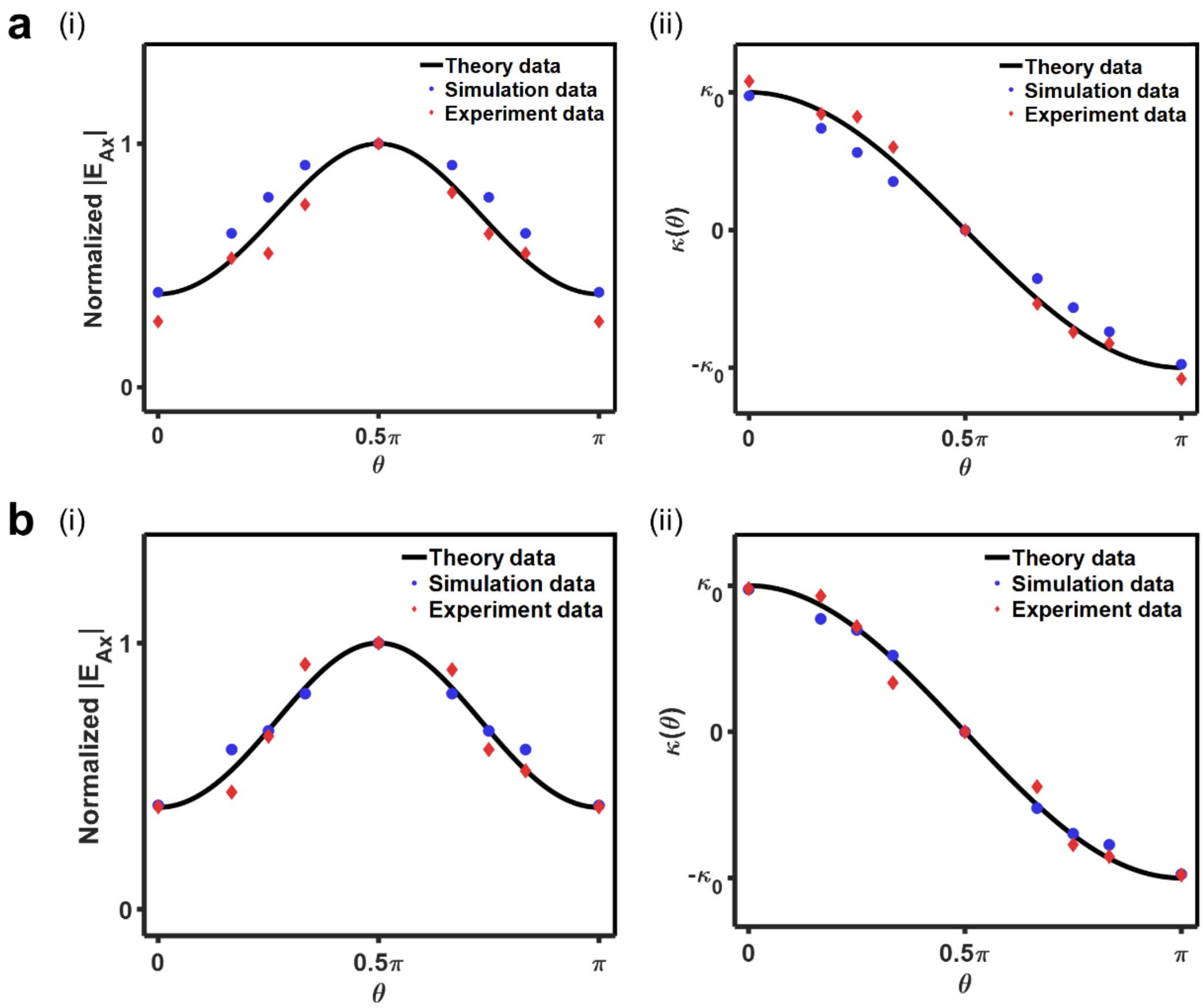

Supplementary Figure 16 | Broadband performance of polarization-controlled coupling at different wavelengths. a, Experimental, simulated, and theoretical results at $\lambda = 1000$ µm. (**i**) normalized $|E_{Ax}|$ as a function of the relative waveguide angle θ, and (**ii**) extracted coupling coefficient $\kappa(\theta)$. ***b***, Corresponding results at $\lambda = 1493$ µm. Black solid curves represent theoretical predictions, blue circles denote numerical simulations, and red diamonds mark experimental measurements.

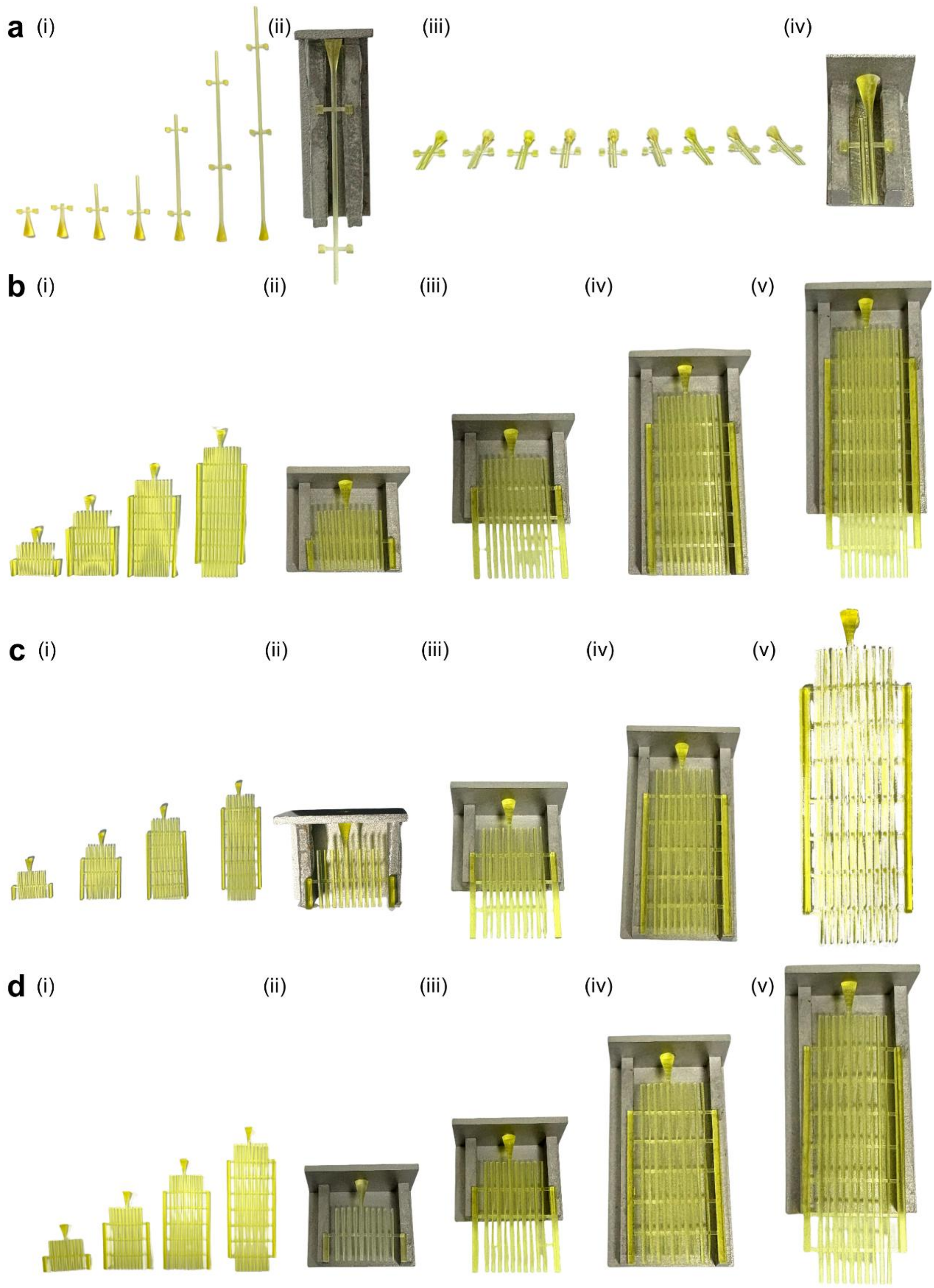

Supplementary Figure 17 | Resin waveguide samples for loss characterization, coupling coefficient measurement and Floquet flat band experiments. a, (**i–iv**) Top views of single waveguides mounted on metallic holders for loss characterization, and paired waveguide assemblies for coupling measurements. **b**, Control samples for Floquet flat-band experiments: (**i–v**) collective image of four sample lengths and individual samples with propagation distances

of 0.25Λ, 0.5Λ, 0.75Λ, and Λ. **c**, Floquet flat-band samples with $\kappa(z) = \kappa_0 \sin(\theta(z))$: (**i** – **v**) collective image of four sample lengths and the corresponding individual samples with propagation distances of 0.25Λ, 0.5Λ, 0.75Λ, and Λ. **d**, Floquet flat-band samples with $\kappa(z) = \kappa_0 \cos(\theta(z))$: (**i** – **v**) collective image of four sample lengths and the corresponding individual samples with propagation distances of 0.25Λ, 0.5Λ, 0.75Λ, and Λ.

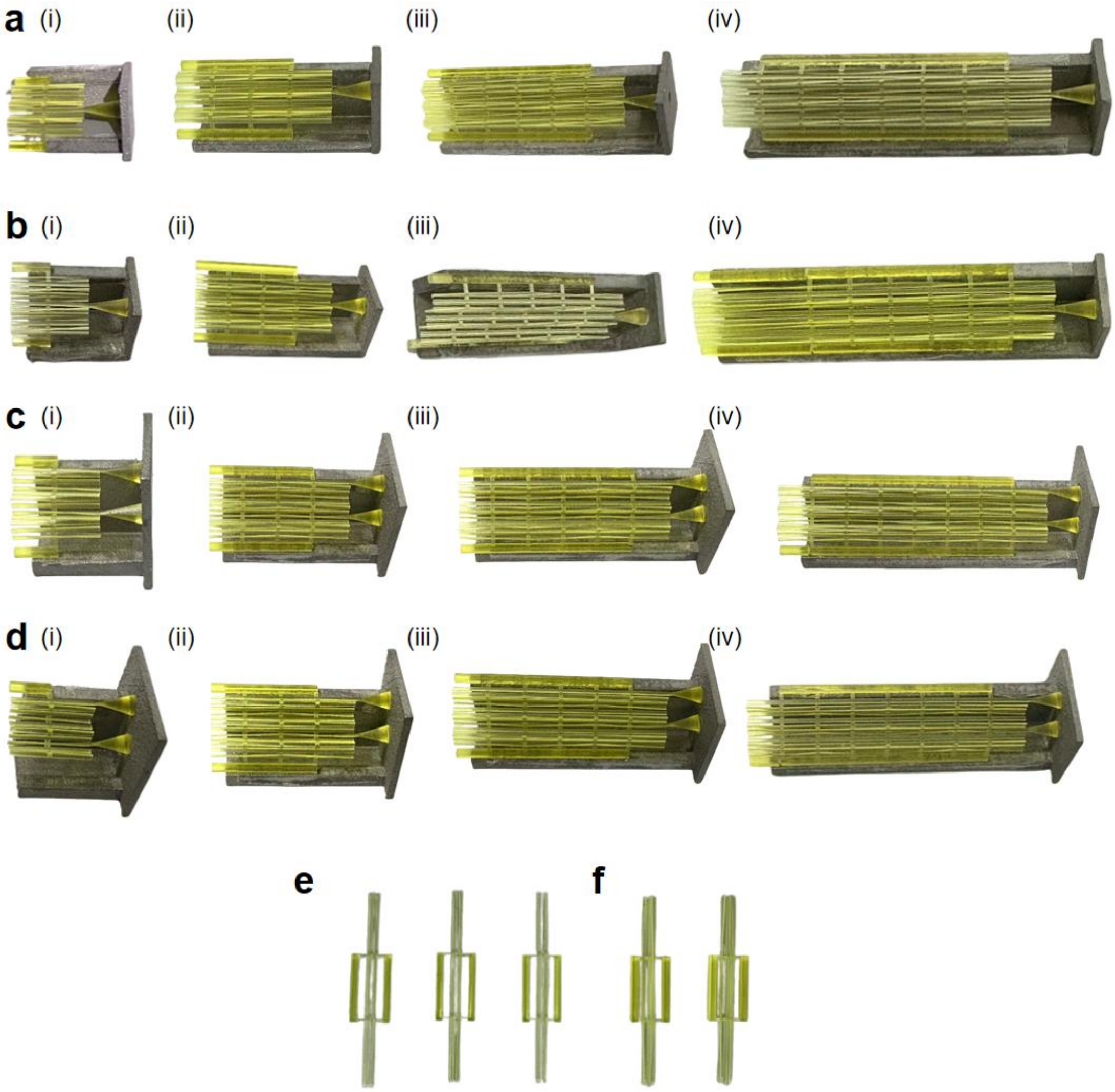

Supplementary Figure 18 | Resin waveguide samples for the period-nΛ oscillation and topological state experiments, and local coupling experiments. a, Samples for the period-Λ oscillation: (**i–v**) collective image of four sample lengths and individual samples with propagation distances of 0.25Λ, 0.5Λ, 0.75Λ, and Λ. **b**, Samples for period-2Λ oscillation: (**i–v**) collective image of four sample lengths and individual samples with propagation distances of 0.22Λ, 0.44Λ, 0.67Λ, and Λ. **c**, Samples for the edge state based on the π gap: (**i–v**) collective image of four sample lengths and individual samples with propagation distances of 0.25Λ, 0.5Λ, 0.75Λ, and Λ. **d**, Samples for the edge state with period-doubled oscillations base on the 0 and π gap: (**i–v**) collective image of four sample lengths and individual samples with propagation distances of 0.25Λ, 0.5Λ, 0.75Λ, and Λ. **e,** Three fabricated two-waveguide coupling units corresponding to the longitudinal coupling functions $\kappa(z) = \kappa_0\cos(\theta(z))$, $\kappa(z) = \kappa_0|\cos(\theta(z))|$, and $\kappa(z) = \kappa_0\sin(\theta(z))$, respectively. **f,** Fabricated four-waveguide

coupling units used for the local two-dimensional coupling experiments, corresponding to the sign-changing coupling $\kappa_{\mathrm{i}}(z) = \kappa_0 \cos(\theta(z) + \theta_0 - \theta_i)$ $(i = 1, 2, 3)$ and the unsigned coupling $\kappa_{\mathrm{i}}(z) = \kappa_0 |\cos(\theta(z) + \theta_0 - \theta_i)|$ $(i = 1, 2, 3)$, respectively.

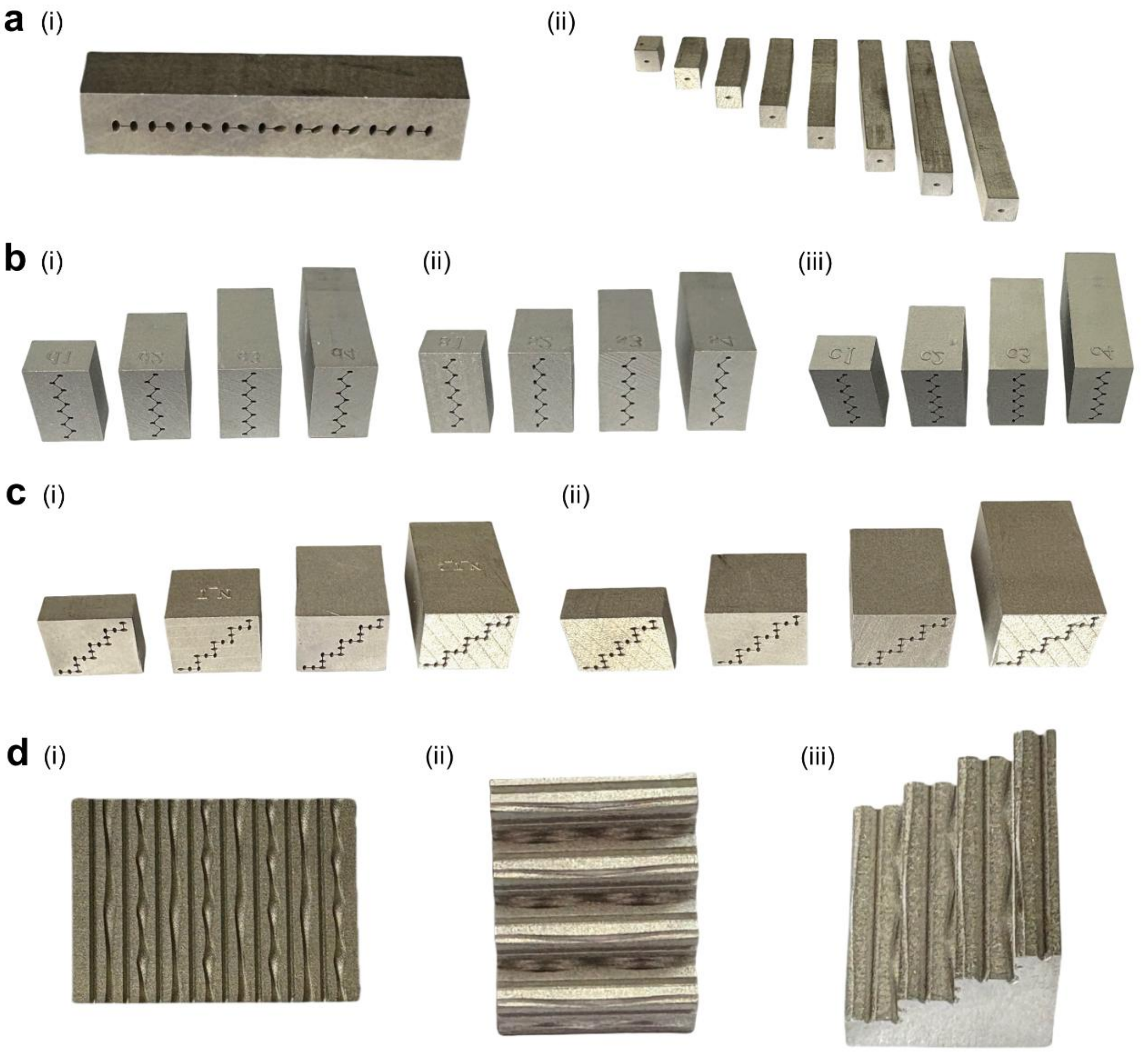

Supplementary Figure 19 | Metal hollow waveguide samples for the loss characterization, coupling coefficient measurement, Floquet flat band, and topological state experiments. **a**, (**i**) Single waveguides with different propagation distances for loss characterization, and (**ii**) paired waveguide assemblies for coupling measurements. **b**, Samples for the Floquet flat-band experiments: (**i**) collective image of control samples with propagation lengths of 0.25Λ, 0.5Λ, 0.75Λ, and Λ, (**ii**) samples with $\kappa(z) = \kappa_0 \sin(\theta(z))$ at propagation lengths of 0.25Λ, 0.5Λ, 0.75Λ, and Λ, and (**iii**) samples with $\kappa(z) = \kappa_0 \cos(\theta(z))$ at propagation lengths of 0.25Λ, 0.5Λ, 0.75Λ, and Λ. **c**, Samples for the topological state experiments: (**i**) samples for edge state based on the π gap at propagation distances of 0.5Λ, Λ, 1.5Λ, and 2Λ, and (**ii**) samples for edge state with period-doubled oscillations base on the 0 and π gap at propagation distances of 0.5Λ, Λ, 1.5Λ, and 2Λ. **d**, Internal cross-section of the metallic hollow waveguide structure: (**i**) top view, (**ii**) side view, and (**iii**) front view.

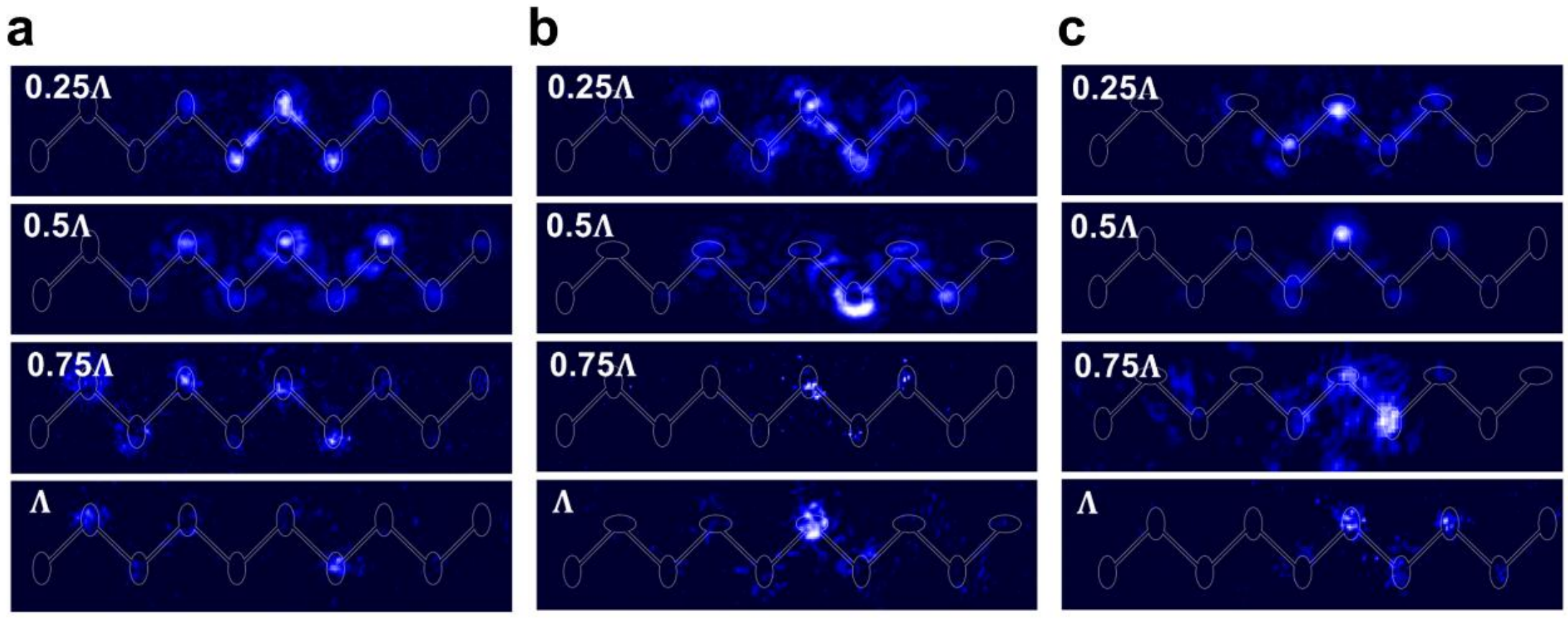

Supplementary Figure 20 | Experimental verification of Floquet flat-band dynamics using metallic hollow waveguide samples. a, Control samples with propagation distances of 0.25Λ, 0.5Λ, 0.75Λ, and Λ, showing spreading light propagation. b, Samples with $\kappa(z) = \kappa_0 \sin(\theta(z))$ and propagation distances of 0.25Λ, 0.5Λ, 0.75Λ, and Λ, demonstrating that light first spreads and then returns to the input waveguide after one driving period. **c**, Samples with $\kappa(z) = \kappa_0 \cos(\theta(z))$ and propagation distances of 0.25Λ, 0.5Λ, 0.75Λ, and Λ, showing that light spreads and refocuses back to the input waveguide after half a period, and repeats thereafter.

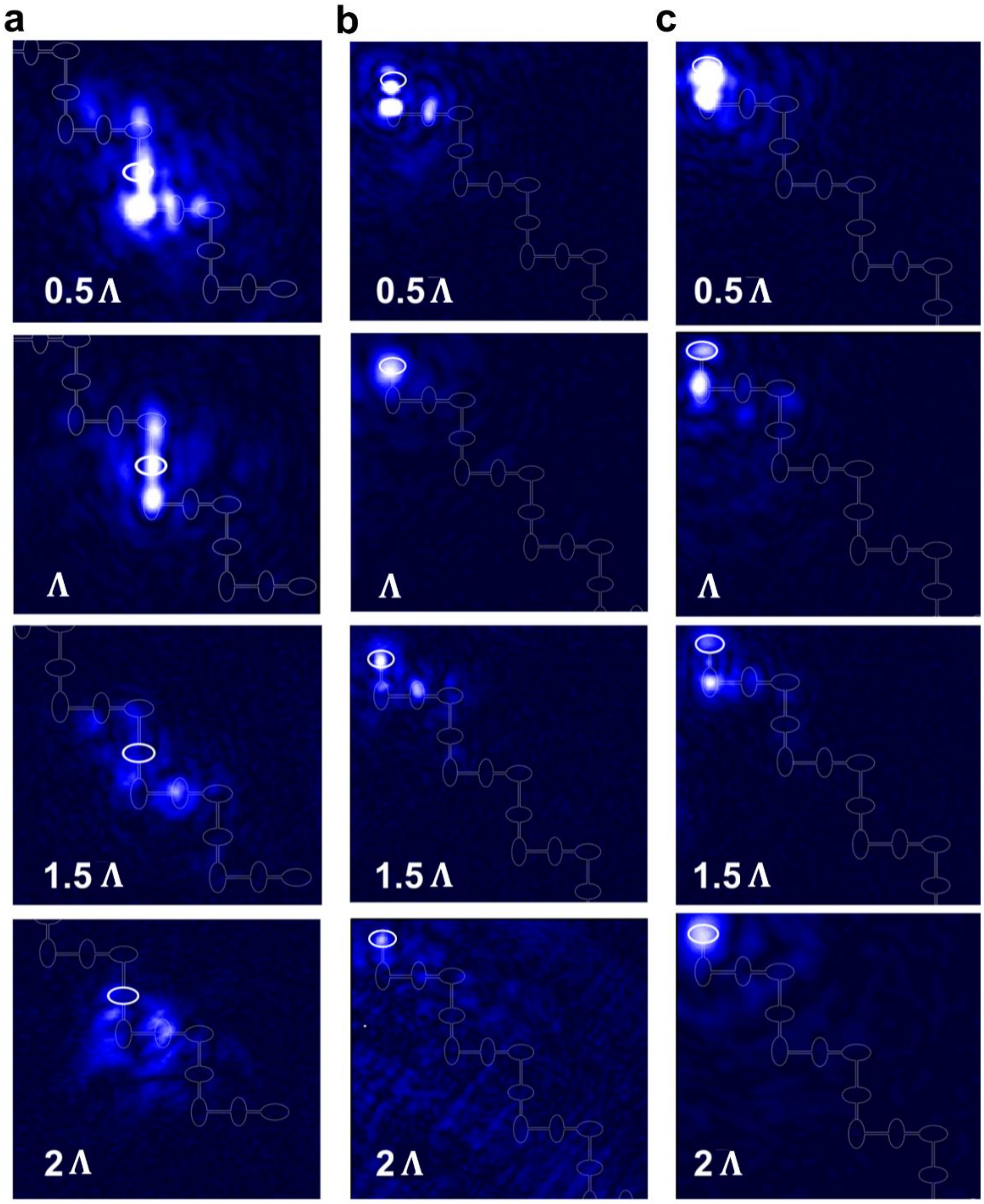

Supplementary Figure 21 | Experimental verification of the topological state using metallic hollow waveguide samples. a, Control samples at propagation distances of 0.5Λ, Λ, 1.5Λ, and 2Λ, showing spreading light propagation. **b**, Samples with $\kappa_1(z) = \kappa_0|\sin(\theta(z))|$, and $\kappa_2(z) = \kappa_0|\cos(\theta(z))|$ at propagation distances of 0.5Λ, Λ, 1.5Λ, and 2Λ, showing edge state with period oscillations base on the π gap. **c**, Samples with $\kappa_1(z) = \kappa_0|\sin(\theta(z))|$, $\kappa_2(z) = \kappa_0|\cos(\theta(z))|$, $\kappa_3(z) = \kappa_0|\sin(2\theta(z))|$, and $\kappa_4(z) = \kappa_0|\cos(2\theta(z))|$ at propagation distances of 0.5Λ, Λ, 1.5Λ, and 2Λ, showing edge state with period-double oscillations base on the 0 and π gap.

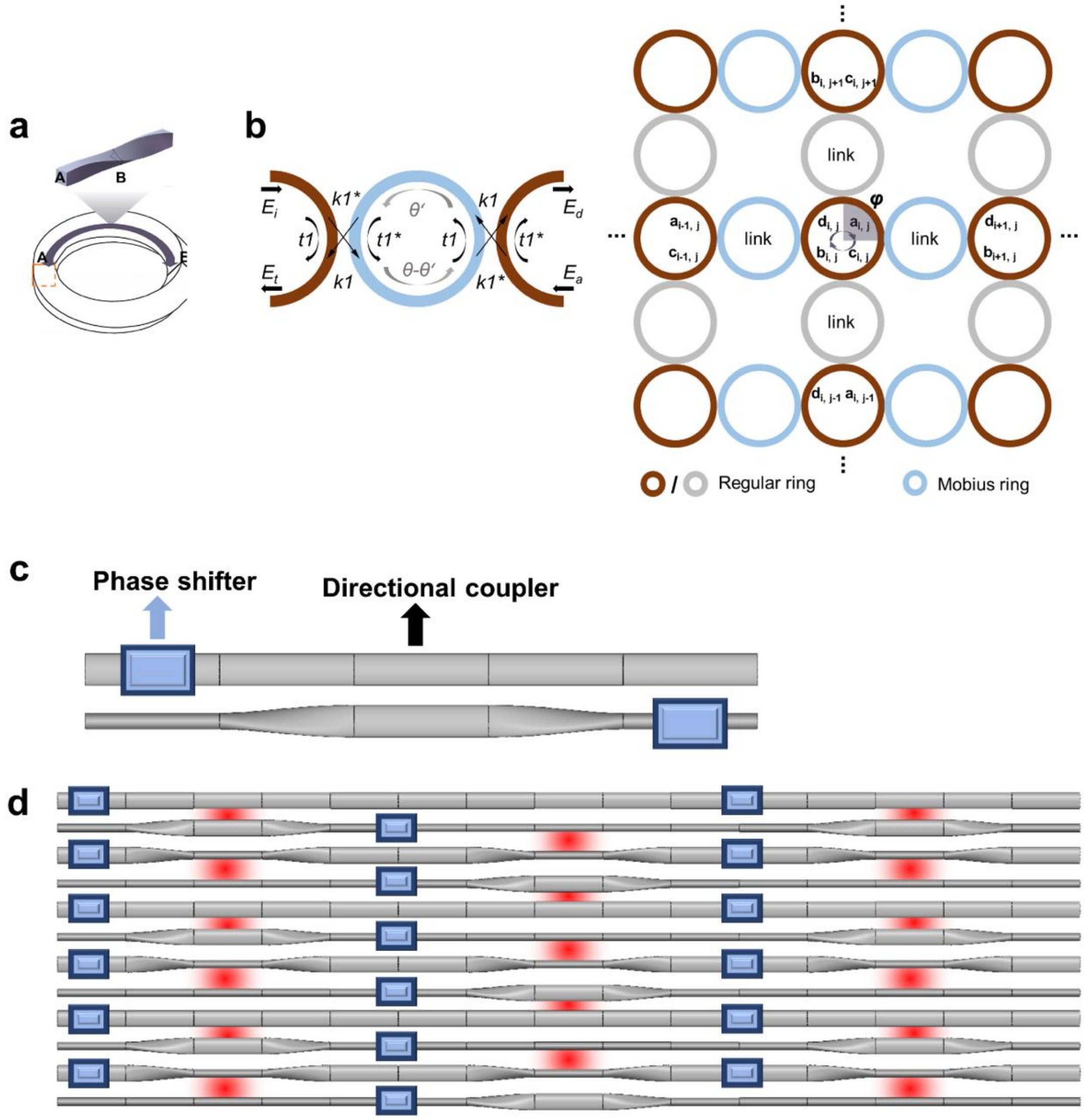

Supplementary Figure 22 | Extension of polarization-controlled coupling to resonator and interferometer systems. a, Schematic of a Möbius ring resonator with a $\frac{\pi}{2}$ twist over one round trip. **b**, Coupled microring resonators incorporating the Möbius ring as linking rings, enabling topological phase transitions controlled by left- and right- circularly polarized light. **c**, Mach–Zehnder interferometer (MZI) unit cell constructed from twisted waveguides, which reduces the device footprint. **d**, Schematic of the MZI array based on polarization-controlled coupling.

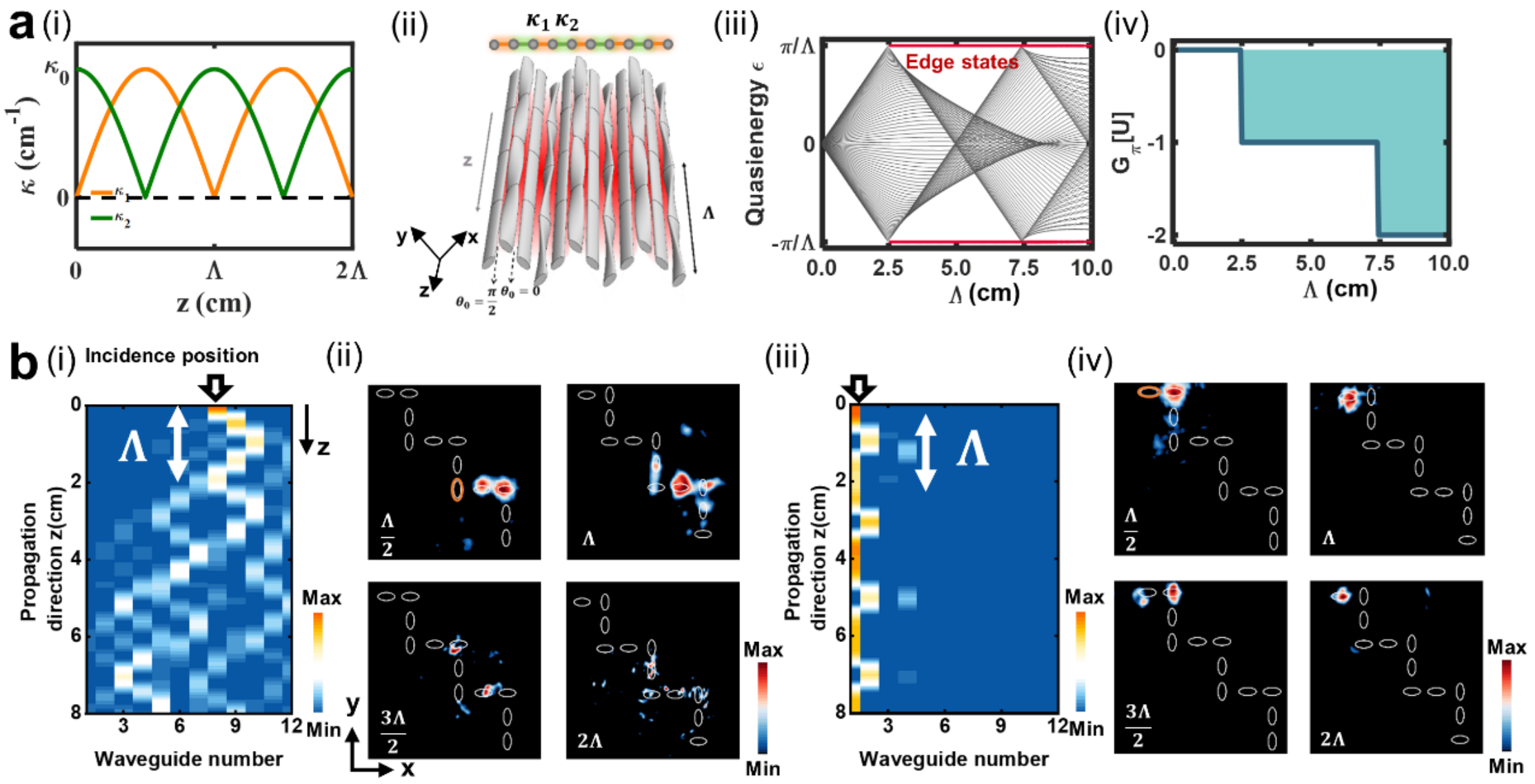

Supplementary Figure 23 | Topological phenomena in a one-demension lattice. a, Topological properties in one-dimensional lattice. **(i)** Coupling coefficient κ_1 and κ_2 as a function of propagation distance z. **(ii)** Structure of the fabricated waveguide array consisting of twelve waveguides with 1000 μm spacing and a propagation distance of $L = 4$ cm, corresponding to two Floquet driving period Λ. **(iii)** The corresponding quasi-energy spectrum under open boundary conditions, calculated for an array of eighty waveguides, exhibits edge states at $\Lambda > 2.5$. **(iv)** Topological invariant calculation confirms topological phase transition as the driving period increases. **b,** Experimental demonstration of edge states in one-dimensional systems. **(i)** Numerical theory of light evolution shows spreading into the bulk. **(ii)** Experimental results at $\frac{\Lambda}{2}$, Λ, $\frac{3\Lambda}{2}$, and 2Λ showing bulk transport and no topological localization. Orange dashed circles highlight the injected waveguides. **(iii)** Numerical theory of light evolution shows robust edge localization. **(iv)** Experimental results reveal strongly localized oscillations near the edge, verifying the presence of Floquet edge states protected by chiral symmetry.

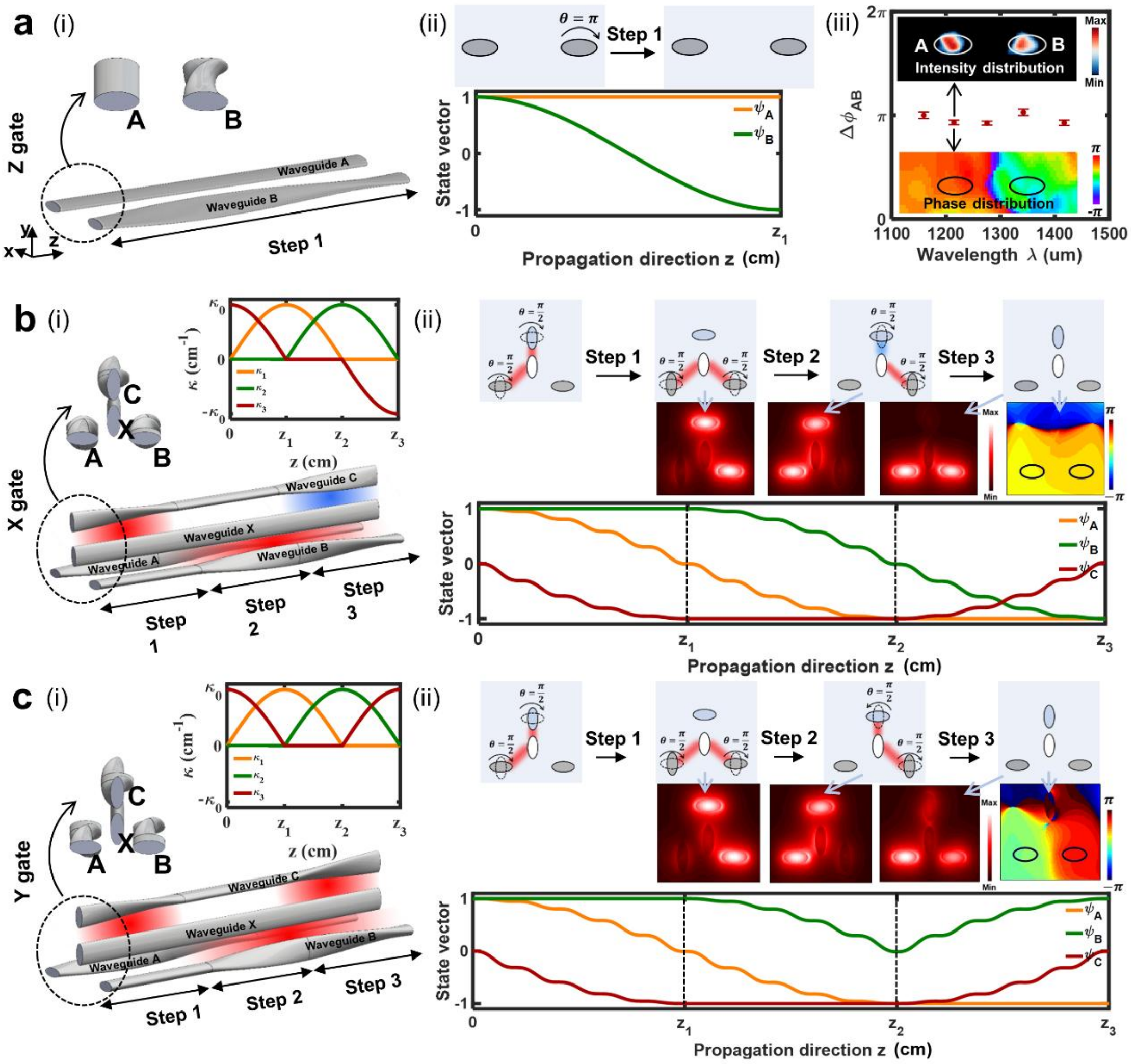

Supplementary Figure 24 | Realization of logical non-Abelian gates based on polarization-controlled coupling. a, Implementation of the logical Z gate. **(i)** Structure consisting of a straight waveguide A and a twisted waveguide B. The fabricated sample has an inter-waveguide spacing of 2000 μm and a length of 1.5 cm. **(ii)** Theoretical evolution of state vectors showing that twisting waveguide B introduces a relative phase of π between the two outputs. **(iii)** Experimental measurements of optical intensity (top) and phase (bottom) distributions confirm that the phase shift remains fixed at π across a broad wavelength range, demonstrating its geometric origin. **b**, Implementation of the logical X gate. **(i)** Four-waveguide structure composed of three twisted waveguides (A, B, C) and one straight waveguide (X). The illustration shows the coupling coefficient κ_1, κ_2 and κ_3 as a function of propagation distance z. **(ii)** Simulated and theoretical results show that the two zero modes acquire identical phase, yielding the X gate transformation. **c**, Implementation of the logical Y gate. **(i)** Similar

structure as in **b**, but in the third step waveguide C is twisted in the opposite direction. The illustration shows the coupling coefficient κ_1, κ_2 and κ_3 as a function of propagation distance z. **(ii)** Simulated and theoretical results show that the two zero modes acquire a relative π phase, yielding the Y gate transformation.

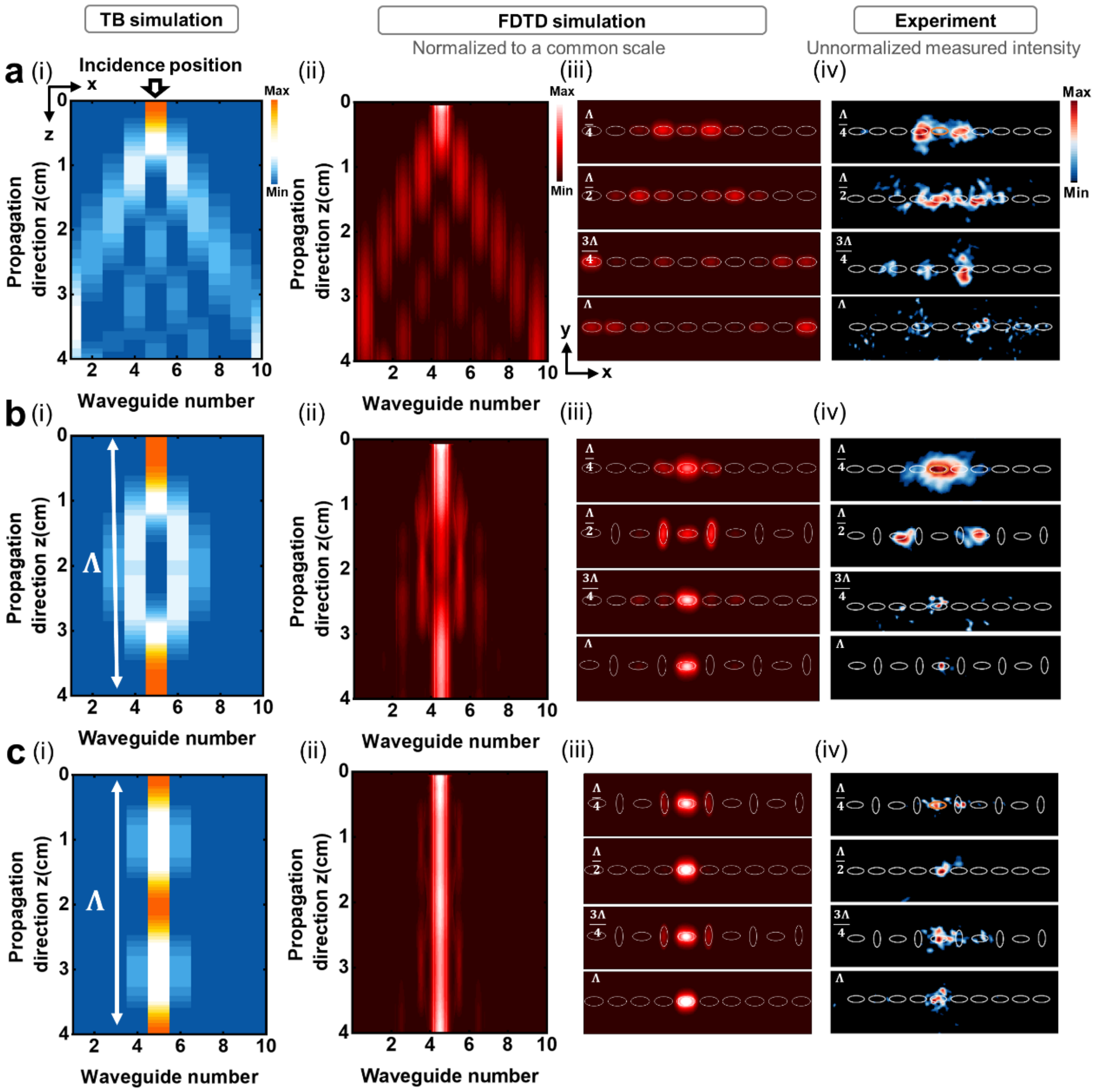

Supplementary Figure 25 | Floquet flat band: comparison between TB simulation, FDTD simulation, and THz-TDS experiment. a–c, Results for three representative cases: **a,** discrete diffraction in a straight lattice for comparison; **b,** Floquet flat band under modulation of $\kappa = \kappa_0 \sin(\theta(z))$; **c,** Floquet flat-band dynamics under modulation of $\kappa = \kappa_0 \cos(\theta(z))$. **(i)** Tight-binding (TB) simulation of the intensity evolution viewed along the propagation direction. **(ii)** Corresponding FDTD simulation of the intensity evolution in the same view. **(iii)** Transverse cross-sectional intensity distributions from FDTD simulation at selected propagation distances $z = \frac{\Lambda}{4}, \frac{\Lambda}{2}, \frac{3\Lambda}{4}$, and Λ. **(iv)** Corresponding cross-sectional intensity distributions measured in the THz-TDS system. The agreement between TB simulation, FDTD simulation, and THz-TDS experiment confirms the flat-band induced by the Floquet engineering.

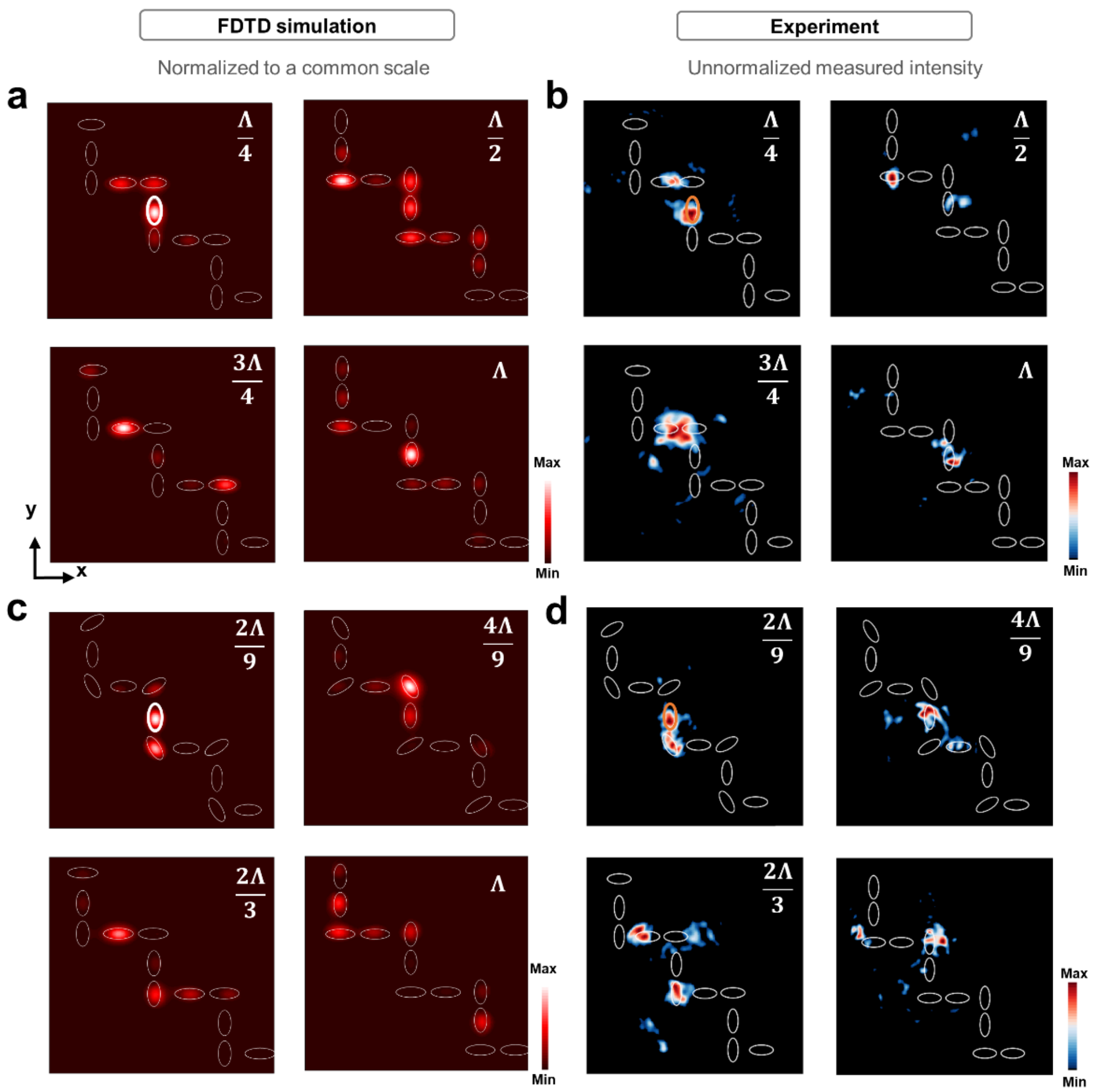

Supplementary Figure 26 | Period-Λ and Period-2Λ oscillations: comparison between FDTD simulation and experiment. a, b, Cross-sectional intensity distributions from FDTD simulation (a) and the corresponding experimental measurements (b) for the period-Λ oscillation. At $z = \Lambda$, the optical field returns to the initially excited waveguide. **c, d,** Cross-sectional intensity distributions from FDTD simulation (c) and the corresponding experimental measurements (d) for the period-2Λ oscillation. At $z = \Lambda$, the optical field does not return to the initially excited waveguide.

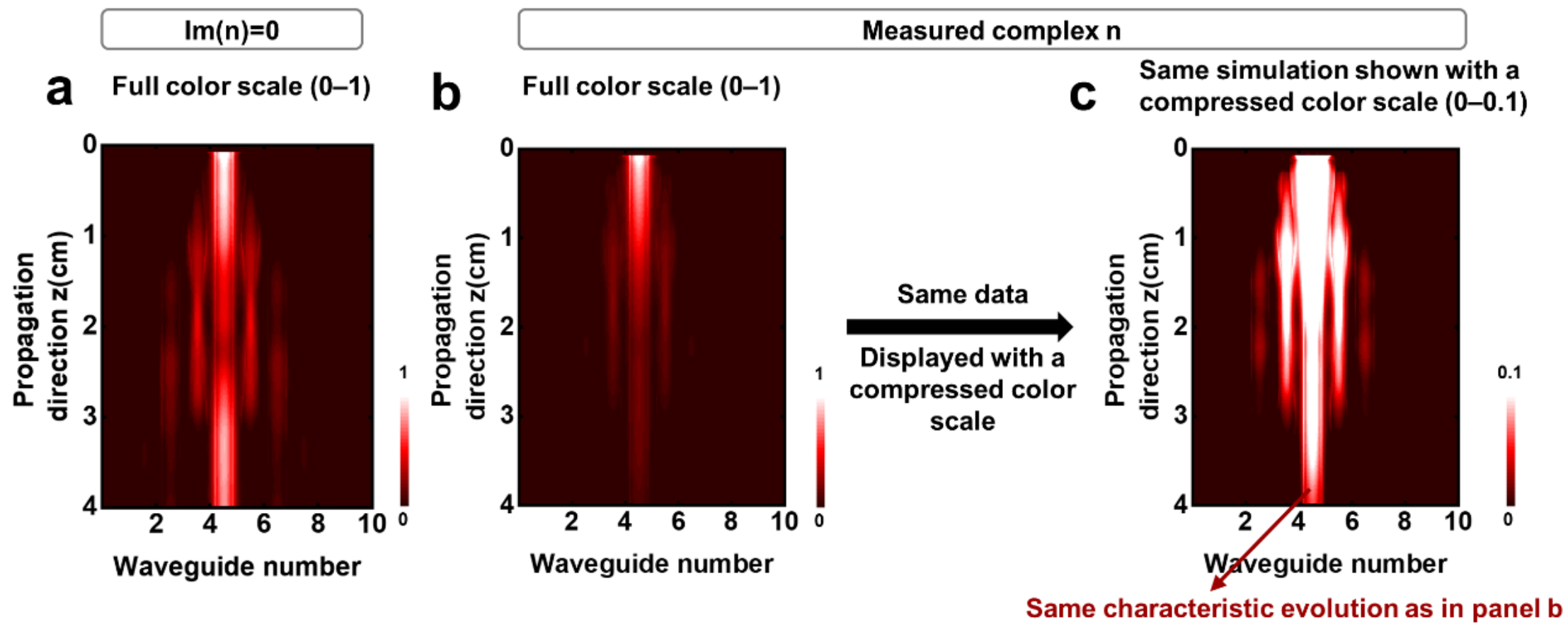

Supplementary Figure 27 | FDTD simulation with the measured material absorption. a, Intensity evolution in the flat band sample with coupling modulation $\kappa = \kappa_0 \sin(\theta(z))$, simulated using the refractive index with Im(n)=0, such that material absorption is neglected. **b, c,** Intensity evolution in the flat band sample with coupling modulation $\kappa = \kappa_0 \sin(\theta(z))$, simulated using the experimentally measured complex refractive index. **b,** Full color scale (0–1). **c,** The same result shown with a compressed color scale (0–0.1). The two panels present the same simulation data with different display ranges. The comparison shows that material absorption mainly reduces the overall signal strength, without affecting the physical phenomenon being verified.

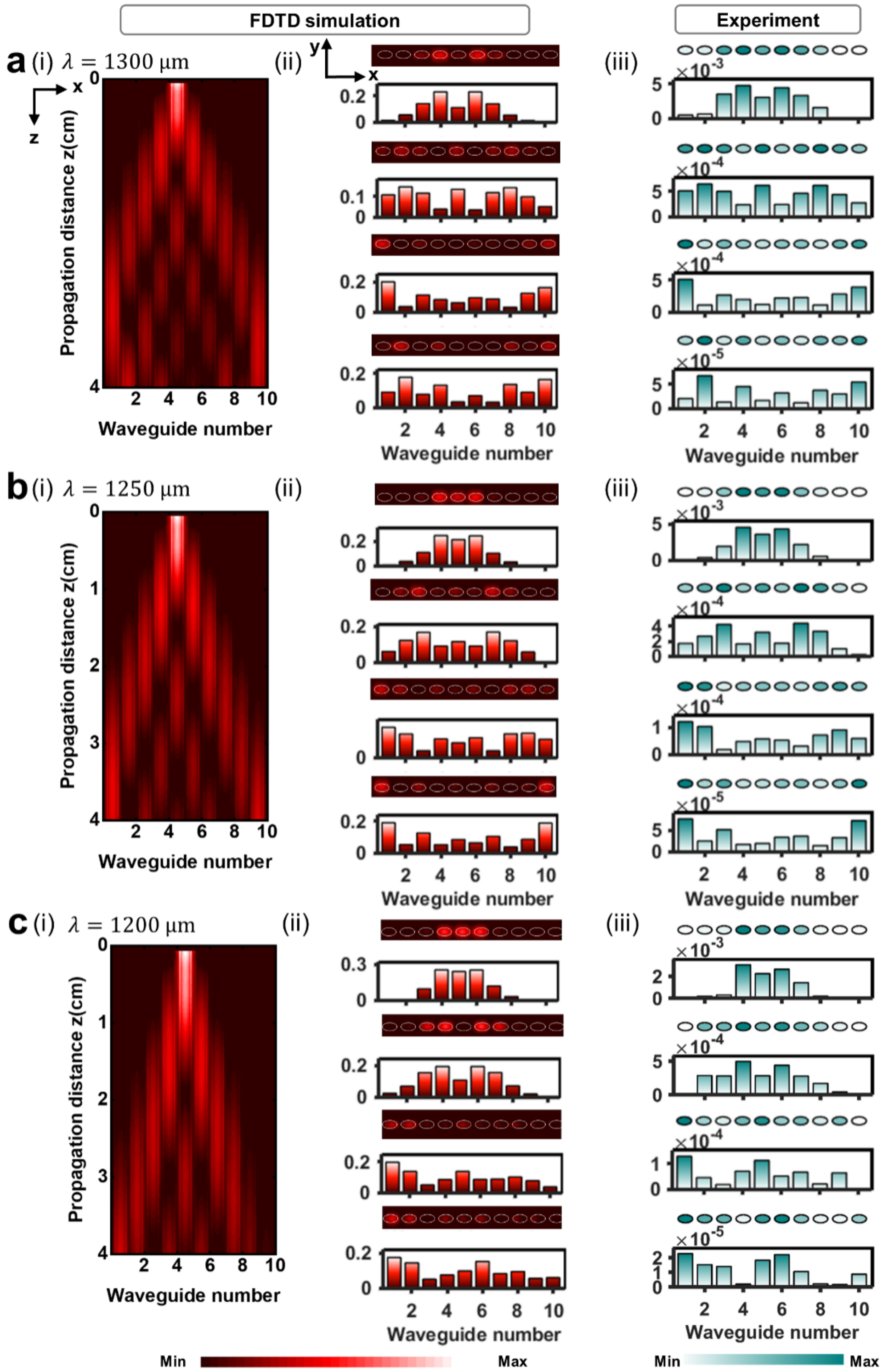

Supplementary Figure 28 | Comparison between FDTD simulations and experimental measurements at different wavelengths for the straight waveguide array after correction of the refractive index used in the simulations. a–c, Results at free-space wavelengths of $\lambda = 1300\ \mu\text{m}, 1250\ \mu\text{m},$ and $1200\ \mu\text{m}$, respectively. **(i)** FDTD simulated intensity evolution along the 4-cm-long waveguide array. **(ii, iii)** FDTD simulated and experimentally measured transverse intensity distributions, respectively, at propagation distances of $z = 1, 2, 3,$ and

4 cm, shown from top to bottom. For each cross section, the colored ellipses visualize the relative intensities in the individual waveguides, while the bar plots show the corresponding waveguide intensities. A single global correction of $\Delta n = 0.02$ is applied to the refractive index, and the corrected value is kept fixed for all wavelengths and propagation distances.

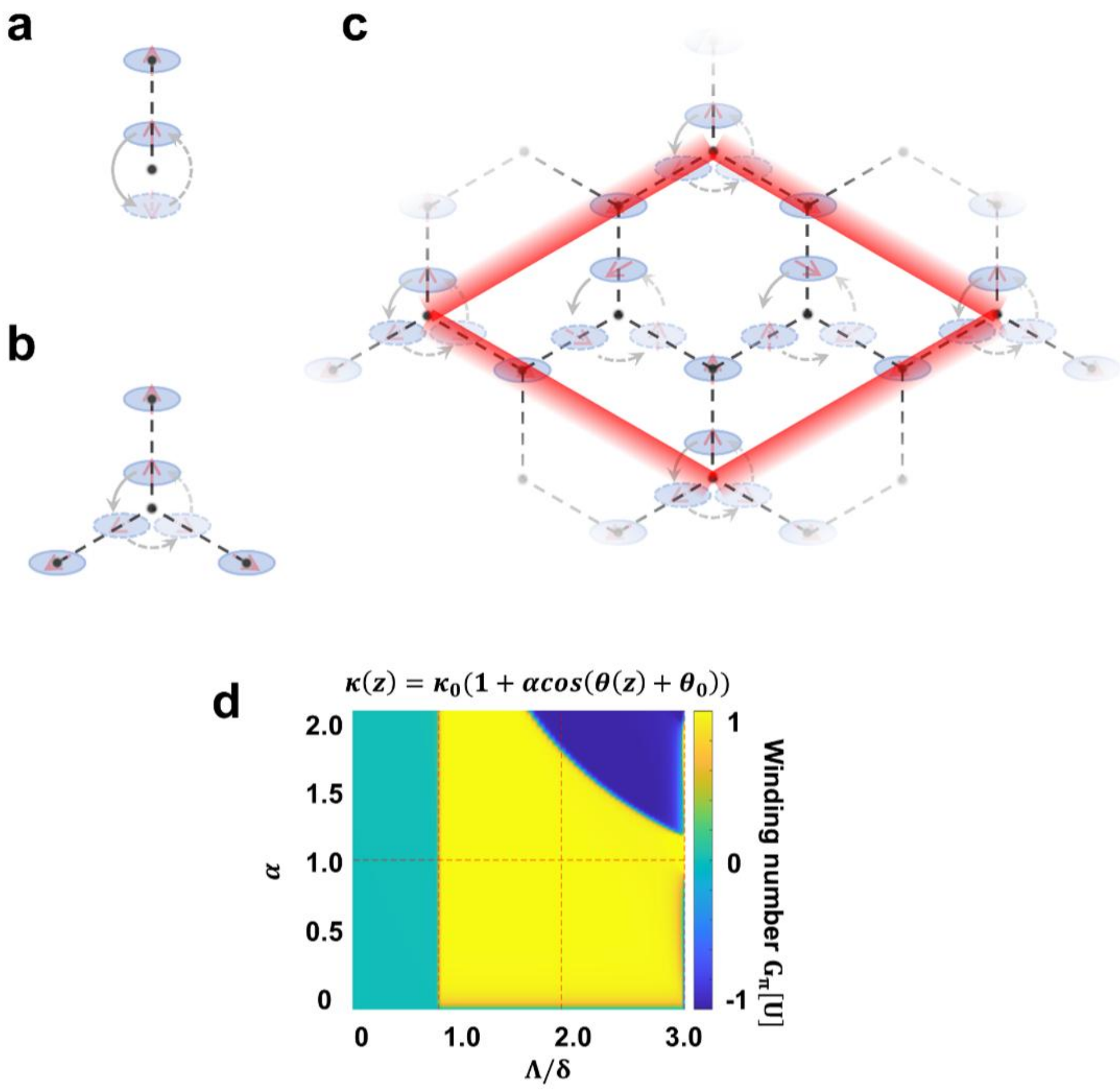

Supplementary Figure 29 | The schematic of the honeycomb lattice and the topological phase transition diagram. a, Coupling configuration of two nearest-neighbor waveguides. One elliptical waveguide remains stationary, while the other adiabatically twists about an axis offset from its own center, such that the relative polarization orientation and the center-to-center separation vary simultaneously. **b,** Arrangement of the twisting waveguide and its three nearest neighbors. The three stationary elliptical waveguides are distributed radially around the twisting one, so that the couplings along the three directions reach their maxima successively within one driving period. **c,** Schematic of the cross section of the honeycomb lattice. The region highlighted in red marks one physical unit cell, which contains six TB sites and is three times larger than the primitive cell of the TB graphene lattice. **d,** Topological phase diagram for the honeycomb lattice with polarization-controlled Floquet engineering. The winding number $G_{\pi}[U]$ is calculated as a function of the modulation depth α and the normalized driving period Λ/δ for the coupling protocol $\kappa_i(z) = \kappa_0(1 + \alpha cos(\theta(z) + \theta_i))$. For $0 < \alpha < 1$, the coupling remains positive throughout the driving cycle, whereas $\alpha > 1$ introduces coupling-sign

reversal, with $\kappa_{\max} > 0$, $\kappa_{\min} < 0$ and $|\kappa_{\max}| > |\kappa_{\min}|$. Within the parameter range shown, this unequal magnitude condition enables a topological transition from $G_\pi = 1$ to $G_\pi = -1$, corresponding to a chirality reversal of the topological edge state. Because the system undergoes two topological phase transitions in the π gap—one at the Γ point and the other at the K point—the winding number changes in two distinct steps. The transition at the Γ point increases the winding number by one and is governed solely by α, whereas the transition at the K point decreases the winding number by two. Therefore, chirality reversal requires the system to first undergo the +1 transition and subsequently the −2 transition. In the phase diagram, the boundary between the yellow and blue regions shifts toward larger α approximately following an inverse dependence on the driving period. At sufficiently large α, this boundary intersects the vertical phase boundary on the left. Beyond this intersection, chirality reversal no longer occurs; instead, only the number of edge states is reduced.

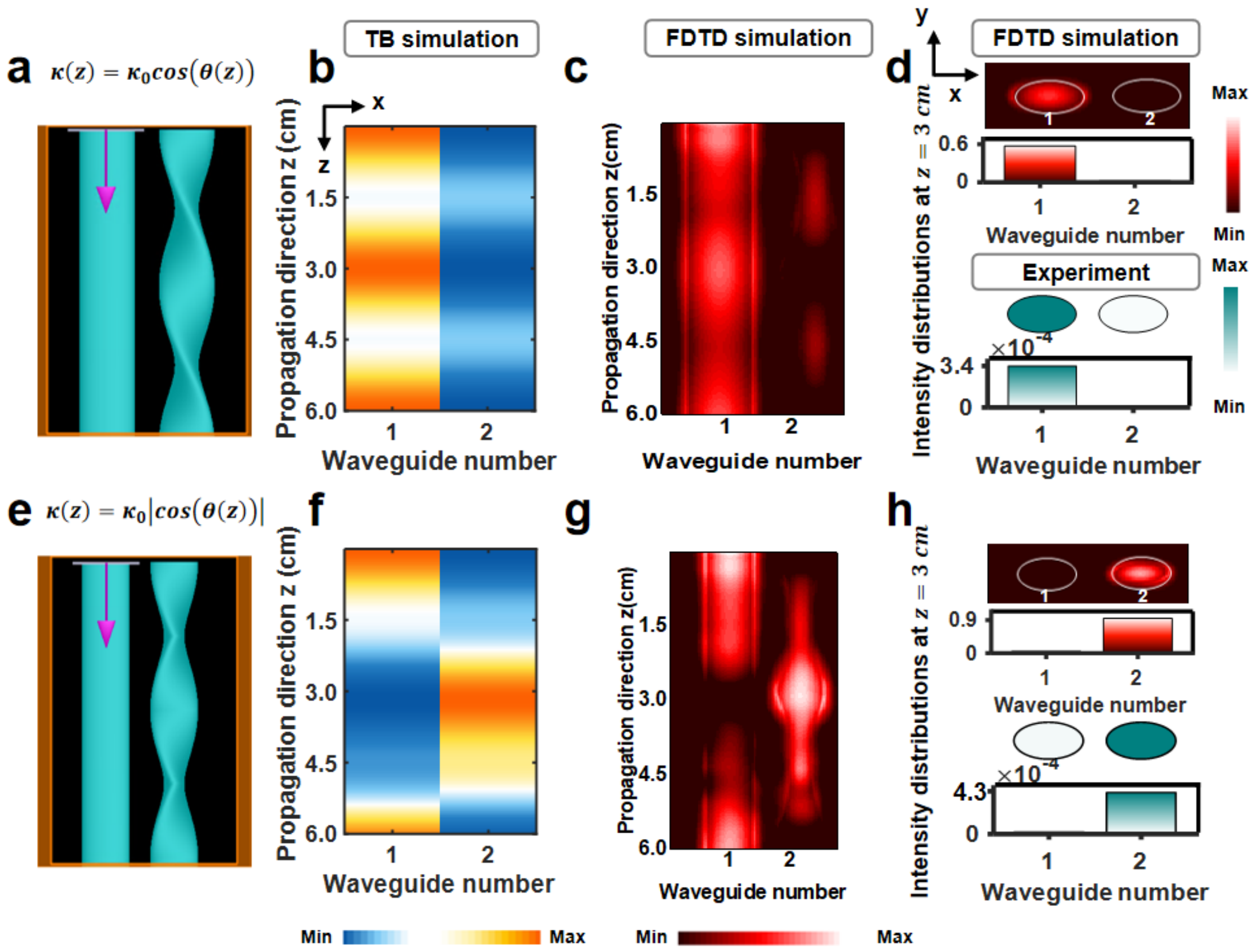

Supplementary Figure 30 | Experimental verification of the physical effect of longitudinal coupling-sign reversal in a two-waveguide unit. The two rows correspond to the longitudinal coupling functions $\kappa(z) = \kappa_0 cos(\theta(z) + \theta_0)$ (a–d), and $\kappa(z) = \kappa_0|cos(\theta(z) + \theta_0)|$ (e–h), respectively. **a, e,** Geometries of the corresponding coupling units, consisting of one straight waveguide and one twisted waveguide. **b, f,** TB calculations of the normalized intensity evolution in the two waveguides. **c, g,** Corresponding FDTD-simulated intensity evolution. **d, h,** Transverse intensity distributions at $z = 3$ cm, obtained from FDTD simulations (upper), and the corresponding experimental measurements (lower). The cosine and absolute-cosine coupling functions have the same coupling magnitude $|\kappa(z)|$ at every propagation position, but differ in whether the coupling sign reverses. Their clearly different propagation dynamics and intensity distributions therefore provide direct evidence of the physical effect of longitudinal coupling-sign reversal.

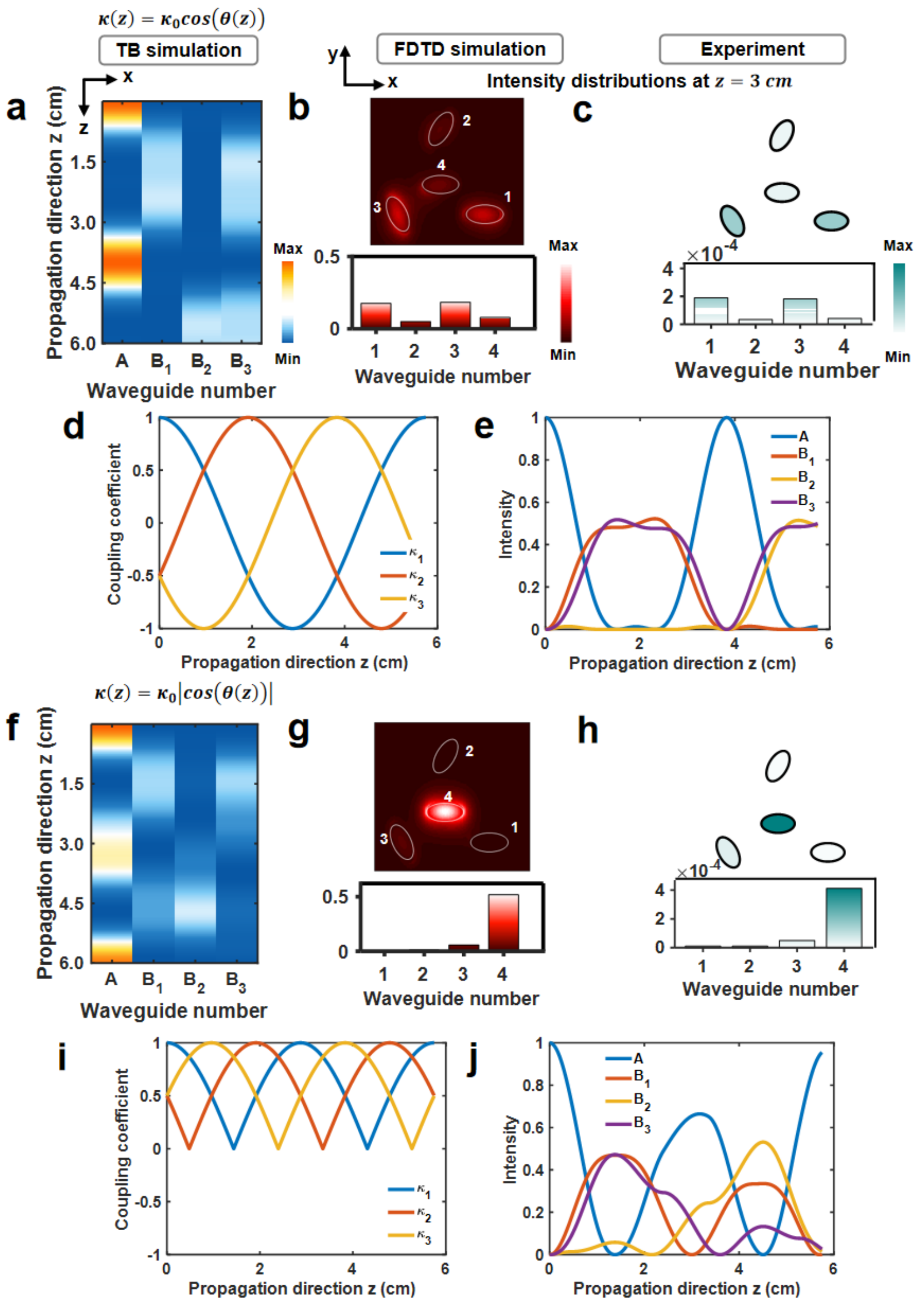

Supplementary Figure 31 | Experimental validation of multi-directional polarization-controlled coupling in a four-site unit. A central waveguide A is coupled to three surrounding waveguides B_1, B_2, and B_3, oriented along three in-plane directions separated by $\frac{2\pi}{3}$. The waveguides have elliptical cross-sections with $a = 350$ μm and $b = 150$ μm, a centre-to-centre separation of $d = 950$ μm, and an operating wavelength of $\lambda = 1200$ μm. **a–e,** Sign-changing coupling modulation. **a,** TB evolution for excitation of the central waveguide A. **b, c,** Corresponding FDTD-simulated and experimentally measured transverse intensity

distributions at $z = 3$ cm, respectively. In **c**, the filling of each ellipse represents the measured output intensity in the corresponding waveguide. **d**, Coupling coefficients between A and the three surrounding waveguides. **e**, TB intensity evolution in the four waveguides. **f-j**, Corresponding results for the reference configuration without changing the sign of coupling. **f**, TB evolution. **g, h,** FDTD-simulated and experimentally measured transverse intensity distributions at $z = 3$ cm, respectively. **i**, Coupling coefficients. **j**, Corresponding TB intensity evolution.

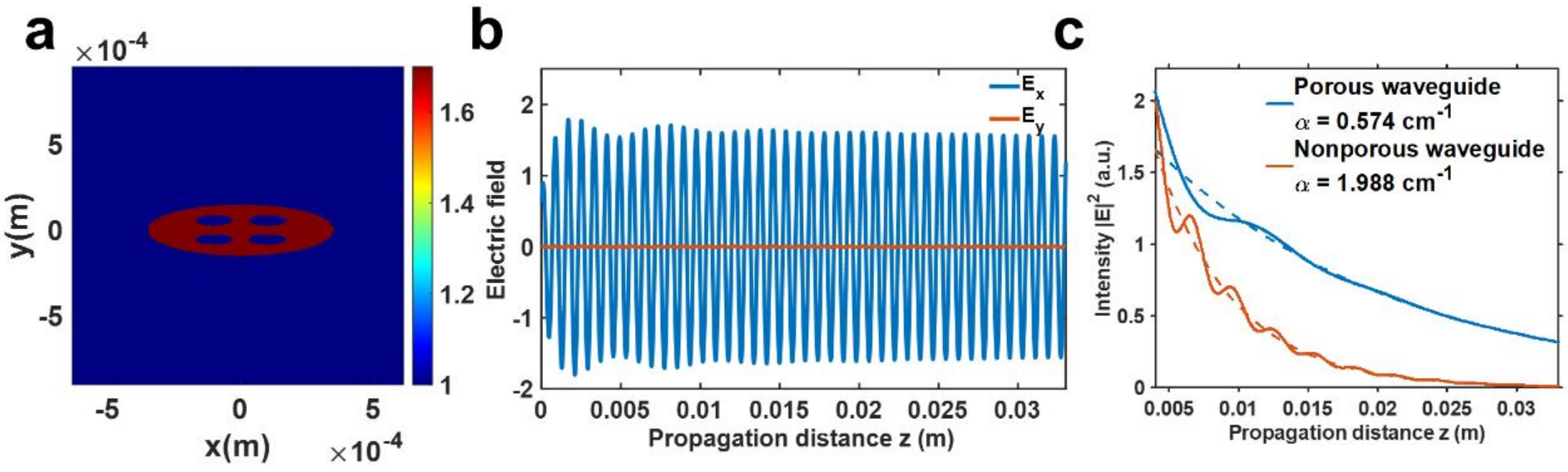

Supplementary Figure 32 | Polarization preservation and reduced propagation loss in a porous resin waveguide. a, Transverse refractive-index distribution of the proposed elliptical resin waveguide containing four symmetrically arranged subwavelength air holes. The major and minor semi-axes of the waveguide are 350 and 150 μm, respectively, while those of each air hole are 70 and 30 μm. **b,** FDTD-calculated evolution of the electric-field components E_x and E_y over a propagation distance of 30λ, for an input polarization aligned with the major axis of the elliptical waveguide. The dominant E_x component and strongly suppressed E_y component indicate that the guided mode remains predominantly linearly polarized along the intended direction. **c**, Comparison of the FDTD-calculated intensity in the porous and nonporous resin waveguides under identical excitation conditions. The porous waveguide exhibits a slower decay and retains a larger power after 30λ of propagation, indicating reduced attenuation owing to the decreased modal overlap with the lossy resin material.

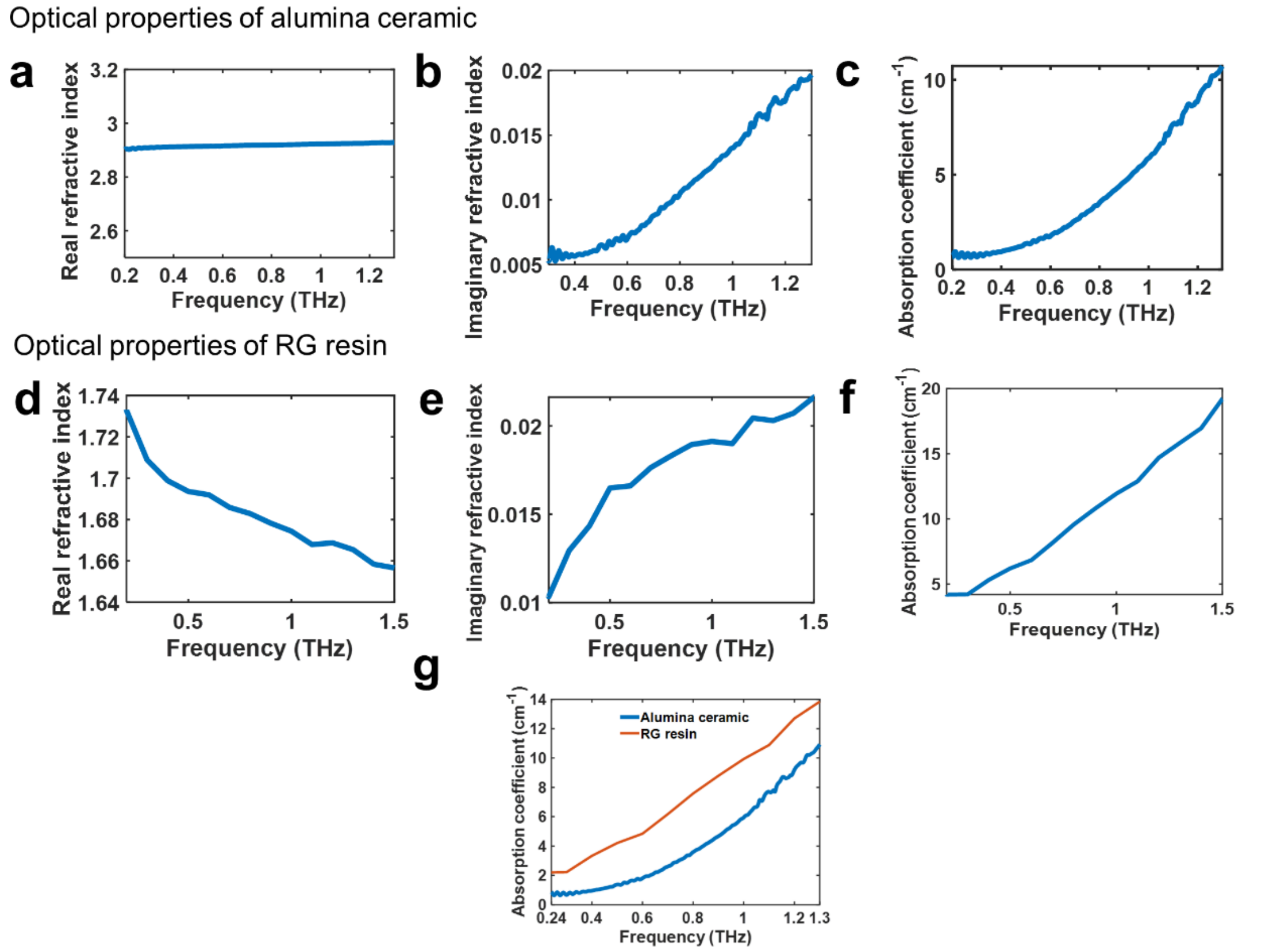

Supplementary Figure 33 | Optical properties of alumina ceramic. a–c, Real part of the refractive index, imaginary part of the refractive index, and absorption coefficient, respectively, of the alumina ceramic measured by TDS over 0.2-1.3 THz. **d–f,** Corresponding quantities of the RG resin measured by TDS over 0.24-1.3 THz. **g,** Comparison between the measured absorption coefficients of the alumina ceramic and the RG resin used in the present experiments over 0.2-1.3 THz, showing consistently lower absorption in the alumina ceramic.

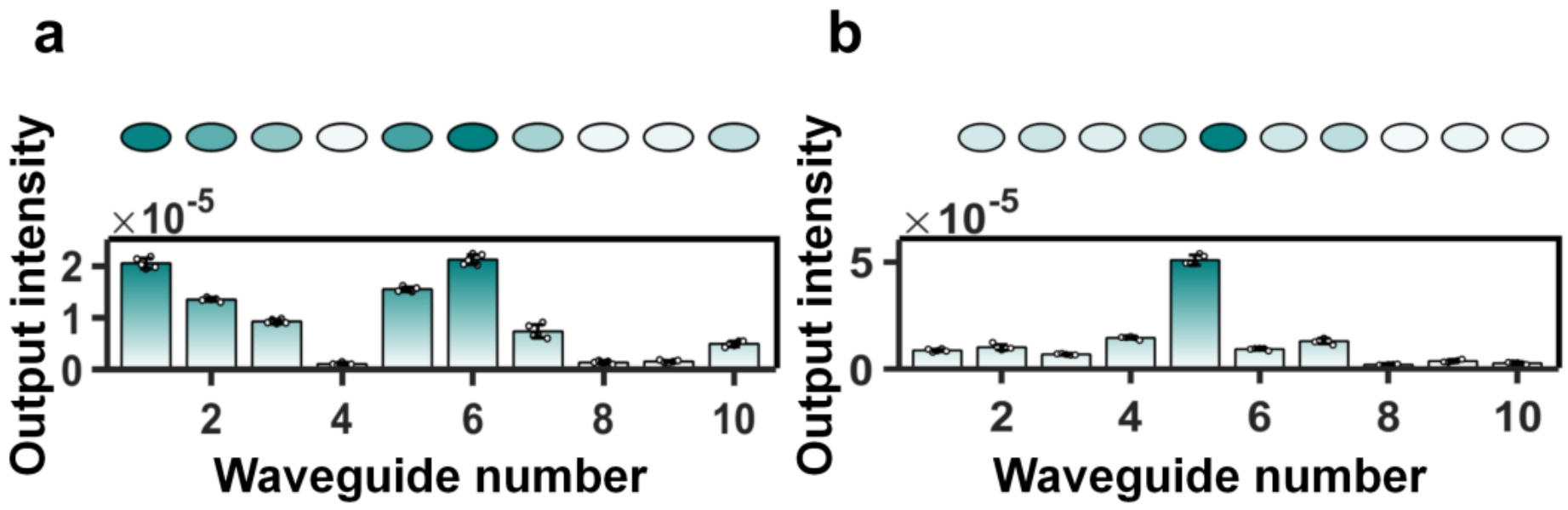

Supplementary Figure 34 | Repeatability of representative experimental output distributions at the maximum propagation distance. a, Straight waveguide array in Fig. 3a after $z = 4$ cm of propagation. **b,** modulated array in Fig. 3b after $z = 4$ cm of propagation. For each waveguide, the bar height represents the mean directly measured output intensity obtained from five repeated measurements under the same experimental conditions, the error bar indicates one standard deviation, and the individual points show the five measured values.

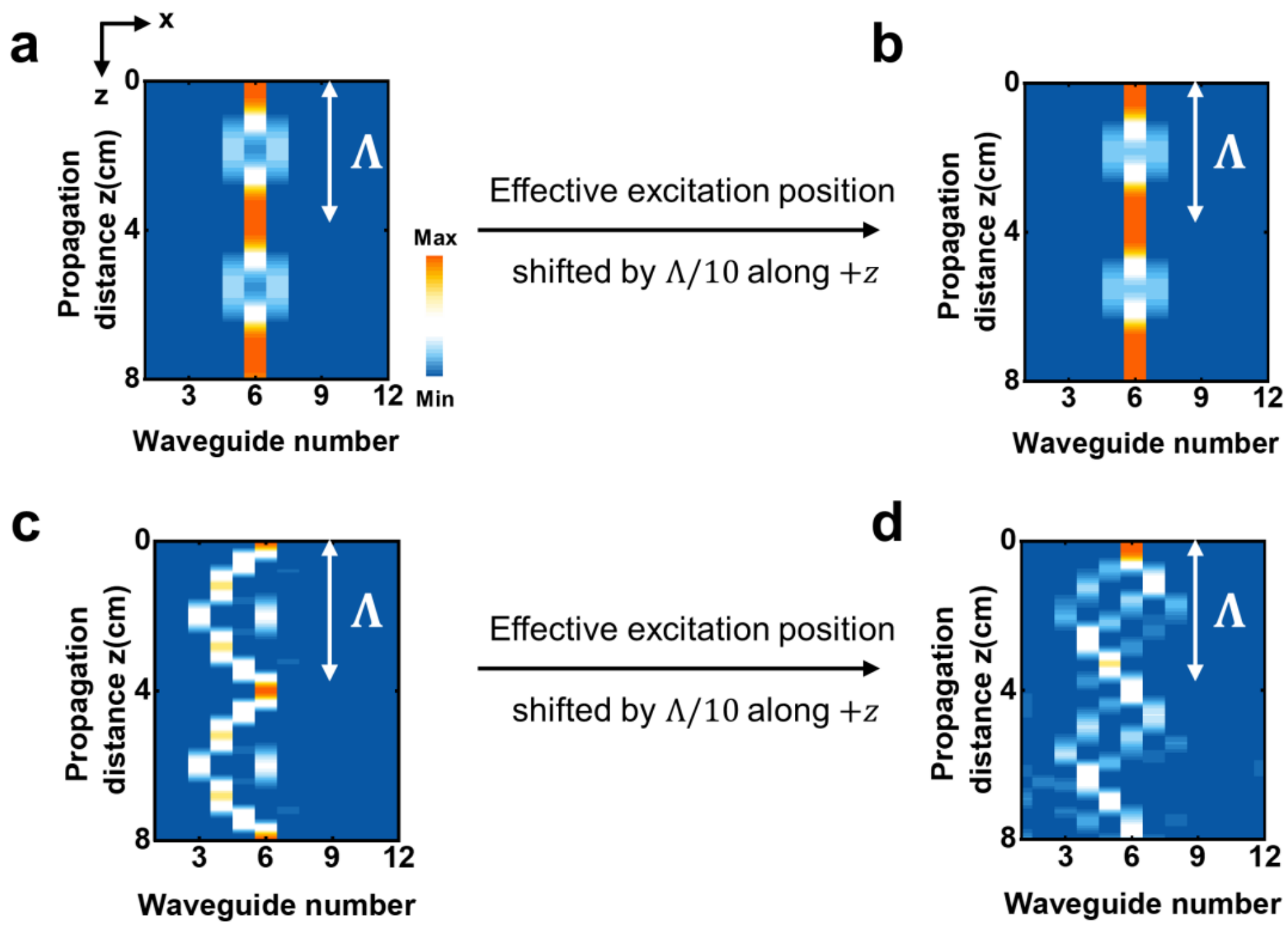

Supplementary Figure 35 | Sensitivity of the propagation dynamics to the effective excitation position. The samples were originally fabricated with horn-shaped input tapers for the THz-TDS measurements. After switching to the VNA configuration, these tapers were mechanically removed to enable direct coupling from the WR-4 metallic waveguide to the dielectric-waveguide facet, resulting in a small forward displacement (corresponding to approximately $\Lambda/10$) of the experimental input facet along the $+z$ direction. **a,b,** TB propagation dynamics for the configuration corresponding to Fig. 3, in which the two couplings adjacent to the excited waveguide have the same longitudinal dependence, being either both $sin[\theta(z)]$-like or both $cos[\theta(z)]$-like. **c,d,** Corresponding propagation dynamics for the configuration in Fig. 4, in which the two adjacent couplings follow $sin[\theta(z)]-$like and $cos[\theta(z)]$-like dependences, respectively. Panels **a** and **c** assume excitation at the original starting position, whereas panels **b** and **d** use a representative forward shift of $\Lambda/10$ along the $+z$ direction to illustrate the sensitivity to the excitation position. The propagation pattern in Fig. 3 changes only weakly under this shift, while that in Fig. 4 shows a more pronounced change because the shift modifies the relative values of the two adjacent couplings at the excitation position, thereby changing the initial power distribution toward the two sides and the subsequent interference dynamics. All coupling parameters are otherwise kept unchanged.

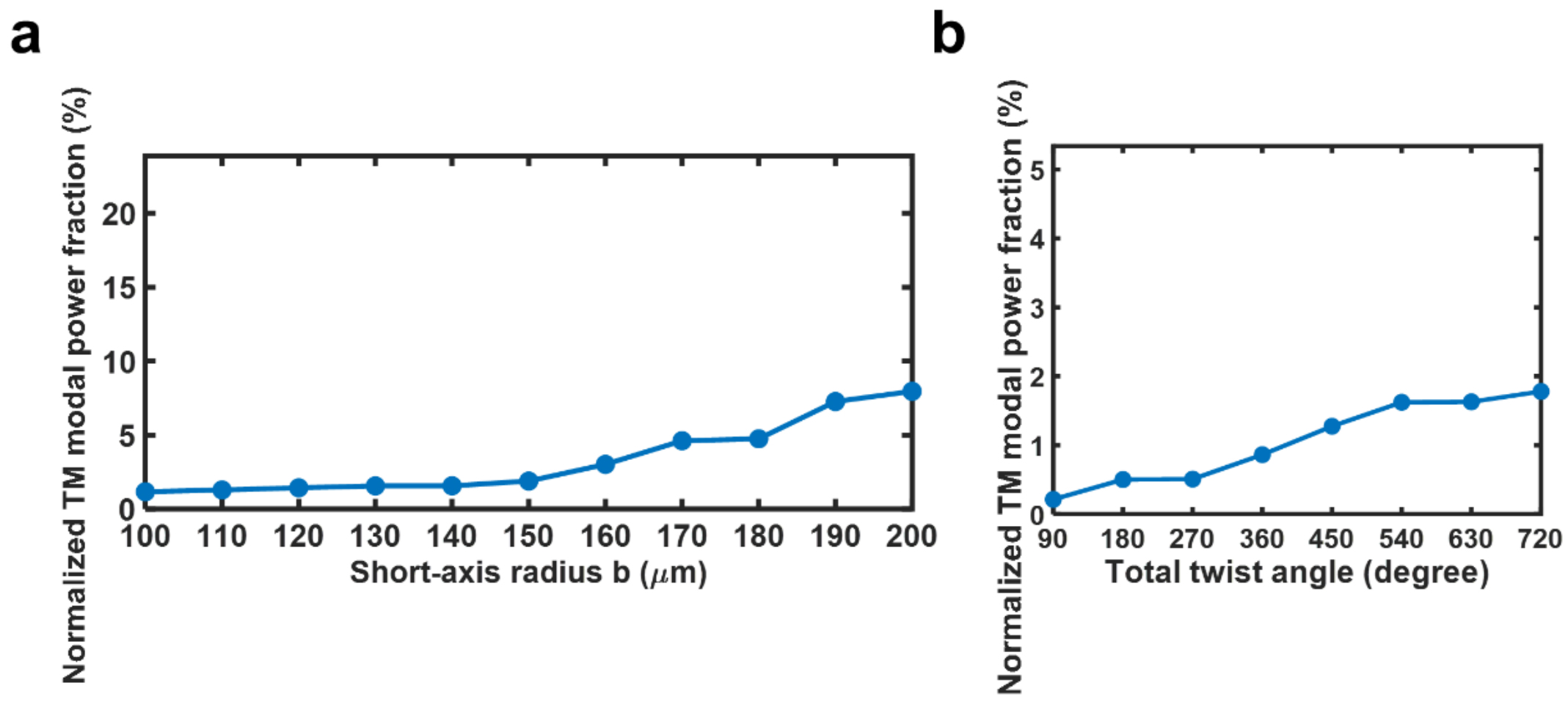

Supplementary Figure 36 | Residual TE–TM mode conversion. a, Normalized TM modal power fraction after 8 cm of propagation as a function of the short-axis radius b, with a=350 μm and a total twist angle of 720°. As b approaches a, the reduced ellipticity decreases the TE-TM propagation-constant splitting and increases mode conversion. **b**, Normalized TM modal power fraction after 8 cm as a function of the total twist angle, with a=350 μm and b=150 μm. Increasing the twist rate reduces the adiabaticity margin and leads to greater residual TE-TM conversion, although the TM fraction remains below 2%, indicating predominantly adiabatic polarization evolution.

Supplementary Table 1 | Comparison between the present work and representative polarization-, orientation-, and modal engineered experimental photonic platforms[12–18].

Platform	Reference	Controlled degree of freedom	Coupling control mechanism	Key distinction from our work				Physical phenomena
				Direct mapping from polarization angle to coupling	Floquet engineering	Physical subfield		
Twisted waveguide array	Our work	Relative polarization angle	Twist controlled modal overlap variation between adjacent waveguides	√	√	Floquet dynamics; topological Floquet physics		Floquet flat bands; period-nΛ oscillations; chirality reversal of topological edge states
Dipolar coupling systems	[15], [18]	Dipole orientation and vectorial field components	Tensorial dipole-dipole interaction; vectorial near field coupling	×	×	Topological physics; vectorial near field spectroscopy		Higher order corner and hinge modes; vectorial near field resonance shifts
Mixed S-P orbital photonic lattices	[13], [12]	Orbital parity and relative mode orientation	P orbital parity induced synthetic π flux	×	×	Topological physics; synthetic-gauge-field physics		Corner states; Aharonov–Bohm caging; Phase-controlled transport
Bianisotropic meta atom lattices	[16]	Relative orientation of split ring resonators	Magneto electric hybridization	×	×	Topological physics		Topological edge and corner states
OAM optical lattices	[17], [14]	Orbital angular momentum states	OAM induced tunnelling phases and effective flux	×	×	Topological physics		Aharonov Bohm caging; topological edge and corner states

Supplementary Table 2 | Quantitative comparison between the FDTD-simulated and experimentally measured intensity distributions. The first table lists the normalized squared Euclidean distance D_{NSE} and the distribution fidelity F at each sampled propagation distance z for Figs. 3a and for the straight-waveguide control sample at incident wavelengths of 1300 μm and 1250 μm. The results at 1200 μm correspond to Fig. 3a in the previous-round response and are included here for comparison. The second table presents the corresponding quantitative comparison for Figs. 3a, 3b and 3c. The third table presents the corresponding quantitative comparison for Figs. 4c and 4d. The last table lists D_{NSE} and F calculated between the normalized FDTD-simulated and experimentally measured output intensity distributions at $z = 3$ cm. The 1D unit consists of two coupled waveguides and compares the sign-changing coupling $\kappa(z) = \kappa_0 cos\big(\theta(z)\big)$ with the corresponding unsigned coupling $\kappa(z) = \kappa_0 \big|cos\big(\theta(z)\big)\big|$. The four-site unit consists of one central waveguide coupled to three surrounding waveguides through $\kappa(z) = \kappa_0 cos(\theta(z) - \theta_i)$, and compares this configuration with the corresponding unsigned couplings $\kappa(z) = \kappa_0 |cos(\theta(z) - \theta_i)|$, where $i = 1,2,3$. A small D_{NSE} and a value of F close to unity indicate greater agreement.

	Previous Fig. 3a: control sample at λ=1200 μm				Fig. 3a: control sample at λ=1200 μm				Supplementary Figure 28a: control sample at λ=1300 μm				Supplementary Figure 28b: control sample at λ=1250 μm			
Propagation distance z (cm)	1	2	3	4	1	2	3	4	1	2	3	4	1	2	3	4
D_{NSE}	0.050	0.134	0.257	0.266	0.019	0.035	0.071	0.064	0.014	0.019	0.006	0.043	0.002	0.021	0.049	0.019
$\overline{D_{NSE}}$	0.177				0.047				0.021				0.023			
F	0.914	0.926	0.837	0.772	0.956	0.962	0.92	0.932	0.972	0.966	0.951	0.946	0.963	0.961	0.941	0.932
$\overline{F}$	0.862				0.943				0.958				0.949			

	Fig. 3a				Fig. 3b				Fig. 3c			
Propagation distance z (cm)	1	2	3	4	1	2	3	4	1	2	3	4
D_{NSE}	0.019	0.035	0.071	0.064	0.022	0.069	0.19	0.038	0.004	0.013	0.032	0.045
$\overline{D_{NSE}}$	0.047				0.081				0.023			
F	0.956	0.962	0.92	0.932	0.970	0.936	0.913	0.933	0.969	0.901	0.947	0.922
$\overline{F}$	0.943				0.938				0.935			

	Fig4c: period-Λ oscillation				Fig4d: period-2Λ oscillation			
Propagation distance z (cm)	1	2	3	4	1	2	3	4
D_{NSE}	0.042	0.120	0.153	0.060	0.049	0.067	0.030	0.187
$\overline{D_{NSE}}$	0.094				0.083			
F	0.913144	0.884768	0.888581	0.925183	0.9138	0.875805	0.950652	0.877337
$\overline{F}$	0.903				0.905			

Structure	1D unit		2D unit	
Coupling coefficient	$\kappa(z) = \kappa_0 cos(\theta(z))$	$\kappa(z) = \kappa_0 \|cos(\theta(z))\|$	$\kappa(z) = \kappa_0 cos(\theta(z))$	$\kappa(z) = \kappa_0 \|cos(\theta(z))\|$
D_{NSE}	0.0000761	0.0000577	0.00288	0.000584
F	0.9994	0.9997	0.9868	0.9928

References

1. *Quantum Mechanics: Non-Relativistic Theory*. vol. 3 (Elsevier, 2013).

2. Kitagawa, T., Berg, E., Rudner, M. & Demler, E. Topological characterization of periodically driven quantum systems. *Phys. Rev. B* **82**, 235114 (2010).

3. Haldane, F. D. M. Model for a Quantum Hall Effect without Landau Levels: Condensed-Matter Realization of the 'Parity Anomaly'. *Phys. Rev. Lett.* **61**, 2015–2018 (1988).

4. Fruchart, M. Complex classes of periodically driven topological lattice systems. *Phys. Rev. B* **93**, 115429 (2016).

5. Wang, Y. *et al.* Large-scale full-programmable quantum walk and its applications. Preprint at https://doi.org/10.48550/arXiv.2208.13186 (2022).

6. Teo, J. C. Y. & Kane, C. L. Topological defects and gapless modes in insulators and superconductors. *Phys. Rev. B* **82**, 115120 (2010).

7. Rajabpoor Alisepahi, A., Sarkar, S., Sun, K. & Ma, J. Breakdown of conventional winding number calculation in one-dimensional lattices with interactions beyond nearest neighbors. *Commun. Phys.* **6**, 334 (2023).

8. Rafi-Ul-Islam, S. M., Siu, Z. B. & Jalil, M. B. A. Topological phases with higher winding numbers in nonreciprocal one-dimensional topolectrical circuits. *Phys. Rev. B* **103**, 035420 (2021).

9. Zhang, X.-L. *et al.* Non-Abelian braiding on photonic chips. *Nat. Photonics* **16**, 390–395 (2022).

10. Chen, Z.-G., Zhang, R.-Y., Chan, C. T. & Ma, G. Classical non-Abelian braiding of acoustic modes. *Nat. Phys.* **18**, 179–184 (2022).

11. Purlu, K. M., Dalgac, S., Korkut, I. & Elmabruk, K. Synthesis, fabrication and characterization of 3D printable photopolymer resins for terahertz applications. *J. Mater. Sci. Mater. Electron.* **36**, 1395 (2025).

12. Cáceres-Aravena, G., Guzmán-Silva, D., Salinas, I. & Vicencio, R. A. Controlled Transport Based on Multiorbital Aharonov-Bohm Photonic Caging. *Phys. Rev. Lett.* **128**, 256602 (2022).

13. Schulz, J., Noh, J., Benalcazar, W. A., Bahl, G. & Von Freymann, G. Photonic quadrupole topological insulator using orbital-induced synthetic flux. *Nat. Commun.* **13**, 6597 (2022).

14. Pelegrí, G., Marques, A. M., Ahufinger, V., Mompart, J. & Dias, R. G. Second-order topological corner states with ultracold atoms carrying orbital angular momentum in optical lattices. *Phys. Rev. B* **100**, 205109 (2019).

15. Zhang, Y., Wu, R. P. H., Shi, L. & Fung, K. H. Second-Order Topological Photonic Modes in Dipolar Arrays. *ACS Photonics* **7**, 2002–2009 (2020).

16. Bobylev, D. A. *et al.* Topological Edge and Corner States Designed via Meta-Atoms Orientation. *Laser Photonics Rev.* **17**, 2100567 (2023).

17. Pelegrí, G. *et al.* Topological edge states with ultracold atoms carrying orbital angular momentum in a diamond chain. *Phys. Rev. A* **99**, 023612 (2019).

18. Esmann, M. *et al.* Vectorial near-field coupling. *Nat. Nanotechnol.* **14**, 698–704 (2019).
