# Supplementary material for: Polarization-controlled Floquet engineering in twisted photonic lattices
Source: Nat Commun. 2026 Oct 15;17:11655. doi: 10.1038/s41467-026-78660-0 (PMC13760052; doi:10.1038/s41467-026-78660-0)
Supplement: Supplementary file 2 [file 41467_2026_78660_MOESM2_ESM.pdf]

nature portfolio

Peer Review File

Polarization-controlled Floquet engineering in twisted photonic lattices

Corresponding Author: Professor Xiaoyong Hu

This file contains all reviewer reports in order by version, followed by all author rebuttals in order by version.

Version 0:

Reviewer comments:

Reviewer #1

(Remarks to the Author)
The paper "Polarization-controlled Floquet engineering in twisted photonic lattices" by Jinze Gao et al. introduces a novel mechanism to achieve arbitrary (complex) and tunable coupling constants between waveguides. The complex coupling is achieved by rotating the elliptical cross-section of waveguides. This effect is show-cased through many examples, ranging from 1D, 2D, to topological and "non-Abelian" systems. Depending on the z-dependent coupling that should be achieved, the authors show different protocols for rotating the waveguide cross-section.
We find the idea of rotating the waveguide cross-section along z to introduce arbitrary coupling functions very interesting and novel. Although references to the influence on the polarization axis in waveguides on the coupling strength exist (see e.g. https://link.aps.org/doi/10.1103/PhysRevA.90.063823), to our knowledge, this paper is the first to use rotation protocols of the waveguide cross-sections to implement arbitrary coupling functions. If this idea could be extended to waveguide arrays in the optical frequencies, this would certainly move the field of waveguide-based quantum simulation forward. However, we have identified two substantive concerns that lead us to the paper's rejection in the current form:
1) Quality of the data and interpretation: The experimental results shown are unfortunately unconvincing. In many cases, the observed intensity maxima do not coincide with the indicated ellipse positions. This discrepancy becomes more pronounced at longer propagation distances, where the pattern increasingly resembles speckle. Without the ellipses marking the waveguide locations, it would be difficult to reliably identify the intended 'spots'. The interpretation is further complicated by the fact that each image is normalized individually, which the authors attribute to losses in the waveguides. This raises the concern that bending losses associated with the cross-section modulation may be sufficiently large that little optical power remains after relatively short propagation distances. We recommend the authors show in careful beam propagation simulations, that include bending losses, that the effect they propose is really of measurable quality and thus useful in experiments.
2) The overall organization of the manuscript requires substantial improvement. While the paper includes an extensive amount of content, key concepts and results are not always explained clearly. As a result, the central message becomes obscured by the large number of examples presented. We recommend the authors to restrict themselves to one example for 1D lattices, and one for 2D lattices. This will already prove their point that arbitrary coupling functions are possible. Further implementations can and should in our opinion be published in separate papers showing only the intended effect (e.g. non-Abelian coupling). Restricting the paper to a few examples would help to make space to explain each effect properly, move the important content for these examples from the SI to the main text and make it less fragmented.

In summary, given the major concerns discussed above, we are unable to recommend publication of the manuscript in Nature Communications. We hope, however, that our comments will help the authors to remodel the paper into a better form, and that thus their interesting idea will find the audience it deserves.

Reviewer #2

(Remarks to the Author)
In the present work the authors consider an array of coupled waveguides which can be oriented in space and their orientation modulated in a desirable matter to control coupling and its phase. As such, the idea of using polarization degree of freedom is not new (refs [1,2] to name just a few, but many early models consider polarized modes). Moreover, it has been used before for controlling coupling of phase in publication Ref. [3]. The authors then follow with several experimental results (although limited to 1D and of experiments of rather poor quality (e.g., judging from experimental figures in Fig. 3).

Conceptually, the paper offers nothing new, and the authors go all over the place to demonstrate the power of the proposed approach. Unfortunately, I do not see any of their results as groundbreaking and sufficiently novel. I would rather see one strong result instead of many mediocre ones, which are also based on the approach that is not truly original. To summarize, the paper lacks originality and does not present any solid/scientifically sound progress that would deserve dissemination in Nature Communications.

[1] Zhang, Y., Wu R. P. H., Shi, L., and Fung, K. H., Second-Order Topological Photonic Modes in Dipolar Arrays, ACS Photonics 7(8) 2002, (2020).
[2] Schulz, J., Noh, J., Benalcazar, W.A. et al. Photonic quadrupole topological insulator using orbital-induced synthetic flux. Nat Commun 13, 6597 (2022).
[3] Bobylev, D. A., Tikhonenko, D. I., Zhirihin, D. V., et al., Topological Edge and Corner States Designed via Meta-Atoms Orientation. Laser Photonics Rev 17, 2100567 (2022).

Reviewer #3

(Remarks to the Author)
There has been much efforts to develop methods for controlling the polarization of modes in various photonic platforms. The authors here present a platform with varied application across a variety of manipulation protocols. Though some of these types of manipulations have appeared in the past in various platforms, these twisted waveguide arrays provide maybe a more streamline platform for various control mechanisms.

Most claims seem to be well supported though I have some questions that need to be answered to help solidify this conclusion. Some of these questions also stand to warrant revision as to help readers understand some of the results presented, so they are not mislead or interpreted incorrectly.

In Fig 2c what is the highlighted region between frequency around .3 and .4 THz? Does this correspond to the 1000 micrometer to 1493 micrometer region described in the caption. If so maybe the units should be change to this connection is clearer.

A lot of effort is made at describing the effect of "negative coupling" as if to make a distinction between positive and negative coupling between the wave guides. The experimental result demonstrates two different signal profiles (fig. 2b(ii) and 2b(iv)) for two different coupling functions. Is it clear that the sign of the coupling and not the functional form of the coupling is the important characteristic. Its not clear to me that the role of the functional form can plays as much as a role in the measured signal profile as the sign.

Just for clarification: the results shown in Fig 2d are for waveguides of fixed coupling (i.e. no dependence on propagation coordinate 'z'). For top panel Fig 2d, is it correct that the input is in the waveguide ellipse labeled B and the output A (this is worth specifying in caption I think).

For Fig. 2c, I think I understand the red curve, what is the measured THz signal (blue dots)?

For Fig. 2c, what is the dashed circles in blue and red highlighting?

Fig 4a. Was a little confused about what A and B are. Are they the integrated areas under the curve in Fig. 4a(i) as depicted? This would make units make sense in 4a(ii). Again I think this is worth spelling out in the text/caption.

Fig 2d. Why does simulation deviate from experiment and theory when θ equals around 0 and π for red curve?

Fig 4c(iii) and 4d(iii): I think the ellipses are representing the 12 waveguide in the array. Might be useful to mention there zigzag pattern (as shown in fig. 4c(i) and 4d(i), but a little hard to see) as the axes in fig. 4c(i) and 4d(i) are rotated relative to fig 4c(iii) and 4d(iii). Or to just indicate direction along the 1D-chain in figure.

Fig 5e: Just want to confirm that these are all theoretical. If so, was wondering if the experiment has been done on this system. If it has not, I think it is important to make it clear that this topological edge state behavior in this 2D honeycomb model has not been experimentally verified in this platform.

Fig 6c: (i) shows which ellipse corresponds to A, B, C, and X. If I understand correctly, in the inset in (ii) showing the three curves as a function of z, waveguide A should acquire a π phase shift and waveguide B should be left invariant. However, in the intensity profile image it looks like A is left invariant and B gets a phase shift. Are the labels backwards here?

Fig 5b. What is Λ for these systems? Is 5b(i) and 5b(iii) both in a regime of Λ where there are edge states or is only 5b(iii)? Looking at 5a(iii) this would be determined if $\Lambda < 2.5$ or $\Lambda > 2.5$, so what is the value for these experiments in 5b(i) and 5b(iii)?

After these questions have been responded to, I can have a better sense to recommend or not to recommend the paper for publication?

For the most part methodology seems to be detailed enough to reproduce results.

Reviewer #4

(Remarks to the Author)
I co-reviewed this manuscript with one of the reviewers who provided the listed reports. This is part of the Nature Communications initiative to facilitate training in peer review and to provide appropriate recognition for Early Career Researchers who co-review manuscripts.

Version 1:

Reviewer comments:

Reviewer #1

(Remarks to the Author)

Reviewer #2

(Remarks to the Author)
While the original submission was relatively unpolished and gave the impression of having been prepared in haste, the revised manuscript presents a much more convincing case for combining polarization-controlled coupling with Floquet modulation. The authors have substantially rewritten the manuscript, addressed criticism by the reviewers, making it considerably easier to follow and clarifying both the motivation and the advantages of the proposed approach. They have also placed their work more clearly in the context of previous studies in which topological photonic phases rely on polarization-dependent coupling, while emphasizing the added role of Floquet modulation.

I believe that this substantially revised version will be of interest to the readers of Nature Communications, and I recommend it for publication in its present form.

Reviewer #3

(Remarks to the Author)
The authors have answered my questions sufficiently. I believe the representation of the data is much more streamlined and intelligible, with more speculative examples placed in the supplementary. While I think the novelty of the work is still not completely ground breaking, I still think the work may warrant publication.

Reviewer #4

(Remarks to the Author)
I co-reviewed this manuscript with one of the reviewers who provided the listed reports. This is part of the Nature Communications initiative to facilitate training in peer review and to provide appropriate recognition for Early Career Researchers who co-review manuscripts.

Version 2:

Reviewer comments:

Reviewer #1

(Remarks to the Author)
The revised manuscript presents more convincing results and we recommend to now accept the paper with minor revisions:
1. The explanation of why the refractive index requires a global shift could be clarified and adapted accordingly. The wording of the origin of the refractive index correction should be improved. It is not that the wavelength dependence of the coupling is responsible for the index shift, but that an assumed wrong refractive index lead to a slightly "wrong" intensity distribution. It would therefore be helpful if the reasoning behind this correction could be explained in a little more detail in the Supplementary Information, particularly in connection with Fig. S28.
2. There is a small typo in the Supplementary Information: in Fig. S29, “honetcomb” should be corrected to “honeycomb”.
3. The photographs of the structure in Fig. S30 are currently not helpful and rather small and therefore difficult to assess.
4. The unit “µm” appears to be missing for the values of 1300 and 1250 in the text of Table S2.
5. For improved understanding, it might be helpful to number the waveguides in figures as for example in Fig. 5(a)(iii).

6. The text from the rebuttal explaining the added figures in the SI (Fig. S28-S36) and T1 and T2 should be added to the SI. Otherwise, the figures are hard for the reader to understand without any additional explanation.

I appreciate that the impact of the results is now discussed more extensively. In particular, the manuscript provides a clearer context regarding previous work on Floquet engineering and polarization control, and it discusses how the underlying concept could potentially be implemented in other platforms. The discrepancies between theory and experiment are also addressed more thoroughly in the revised version.
Overall, I find that the revised submission is now understandable and sufficiently well contextualized. Subject to these minor corrections, I believe that the revised manuscript is suitable for publication.

Reviewer #4

(Remarks to the Author)
I co-reviewed this manuscript with one of the reviewers who provided the listed reports. This is part of the Nature Communications initiative to facilitate training in peer review and to provide appropriate recognition for Early Career Researchers who co-review manuscripts.

Open Access This Peer Review File is licensed under a Creative Commons Attribution 4.0 International License, which permits use, sharing, adaptation, distribution and reproduction in any medium or format, as long as you give appropriate credit to the original author(s) and the source, provide a link to the Creative Commons license, and indicate if changes were made.
In cases where reviewers are anonymous, credit should be given to 'Anonymous Referee' and the source.
The images or other third party material in this Peer Review File are included in the article’s Creative Commons license, unless indicated otherwise in a credit line to the material. If material is not included in the article’s Creative Commons license and your intended use is not permitted by statutory regulation or exceeds the permitted use, you will need to obtain permission directly from the copyright holder.
To view a copy of this license, visit https://creativecommons.org/licenses/by/4.0/

Dear reviewers:

Thank you for taking the time to review our manuscript entitled "Polarization-controlled Floquet engineering in twisted photonic lattices" (Manuscript ID: NCOMMS-25-101957-T).

We greatly appreciate your valuable comments and suggestions, which have been carefully considered in our revisions. Below, we have provided a point-by-point response, along with the corresponding changes made in the revised manuscript and supplementary materials for your reference. We believe that all the comments have been addressed, the revised manuscript meets the standard of *Nature Communications* for publication.

Reply to Reviewer 1:

General Comment:

The paper "Polarization-controlled Floquet engineering in twisted photonic lattices" by Jinze Gao et al. introduces a novel mechanism to achieve arbitrary (complex) and tunable coupling constants between waveguides. The complex coupling is achieved by rotating the elliptical cross-section of waveguides. This effect is show-cased through many examples, ranging from 1D, 2D, to topological and "non-Abelian" systems. Depending on the z-dependent coupling that should be achieved, the authors show different protocols for rotating the waveguide cross-section.

We find the idea of rotating the waveguide cross-section along z to introduce arbitrary coupling functions very interesting and novel. Although references to the influence on the polarization axis in waveguides on the coupling strength exist (see e.g. https://link.aps.org/doi/10.1103/PhysRevA.90.063823), to our knowledge, this paper is the first to use rotation protocols of the waveguide cross-sections to implement arbitrary coupling functions. If this idea could be extended to waveguide arrays in the optical frequencies, this would certainly move the field of waveguide-based quantum simulation forward. However, we have identified two substantive concerns that lead us to the paper's rejection in the current form.

Responses:

We sincerely thank the Reviewer for this generous assessment of the conceptual novelty and potential significance of our work. We are particularly encouraged by the Reviewer's recognition that this is the first work to use rotation protocols of waveguide cross-sections to implement arbitrary coupling functions, and if extended to optical-frequency waveguide arrays, this approach may move the field of waveguide-based quantum simulation forward.

Guided by the Reviewer's subsequent concerns, we have substantially strengthened the revised version so that this central advance is presented more clearly and supported

more convincingly through the **beam propagation simulation, improved experimental verification and a more focused presentation**.

Comments1:

Quality of the data and interpretation: The experimental results shown are unfortunately unconvincing. In many cases, the observed intensity maxima do not coincide with the indicated ellipse positions. This discrepancy becomes more pronounced at longer propagation distances, where the pattern increasingly resembles speckle. Without the ellipses marking the waveguide locations, it would be difficult to reliably identify the intended 'spots'. The interpretation is further complicated by the fact that each image is normalized individually, which the authors attribute to losses in the waveguides. This raises the concern that bending losses associated with the cross-section modulation may be sufficiently large that little optical power remains after relatively short propagation distances. We recommend the authors show in careful beam propagation simulations, that include bending losses, that the effect they propose is really of measurable quality and thus useful in experiments.

Responses:

We thank the reviewer for this important comment regarding the data quality and the possible role of propagation loss. To address this concern more directly and convincingly, we have substantially strengthened the revised manuscript in four aspects: **I. adding beam propagation simulations, II. remeasuring the key experimental results using the terahertz vector network (THz-VNA) system with higher source power and higher signal-noise-ratio, III. clarifying the physical origin of the loss both experimentally and numerically, IV. Experimentally proving the convincing of the experimental results from the previous main text.**

I. We added beam-propagation simulations using full-wave finite-difference time-domain (FDTD) method that confirm the correctness of the tight-binding (TB) analysis.

To make this validation explicit, Fig. R1 first compares the TB calculation, full-wave FDTD simulation, and the original terahertz time-domain spectroscopy (THz-TDS) measurement for the Floquet flat-band samples. The good agreement among the TB, FDTD, and experimental results confirms the validity of the theoretical model, numerical simulations and experimental measurements. The same comparison is extended to the period-Λ and period-2Λ oscillation samples in Fig. R2. The comparison further demonstrates the consistency between the theoretical model, numerical simulations and experimental measurements. The actual FDTD geometries used in these simulations are shown in Fig. R3, confirming that the simulations are performed on structures corresponding to the fabricated waveguide arrays, rather than on idealized models. The material absorption in the terahertz regime is not included in these FDTD simulations and all FDTD results are presented using a common normalization scale.

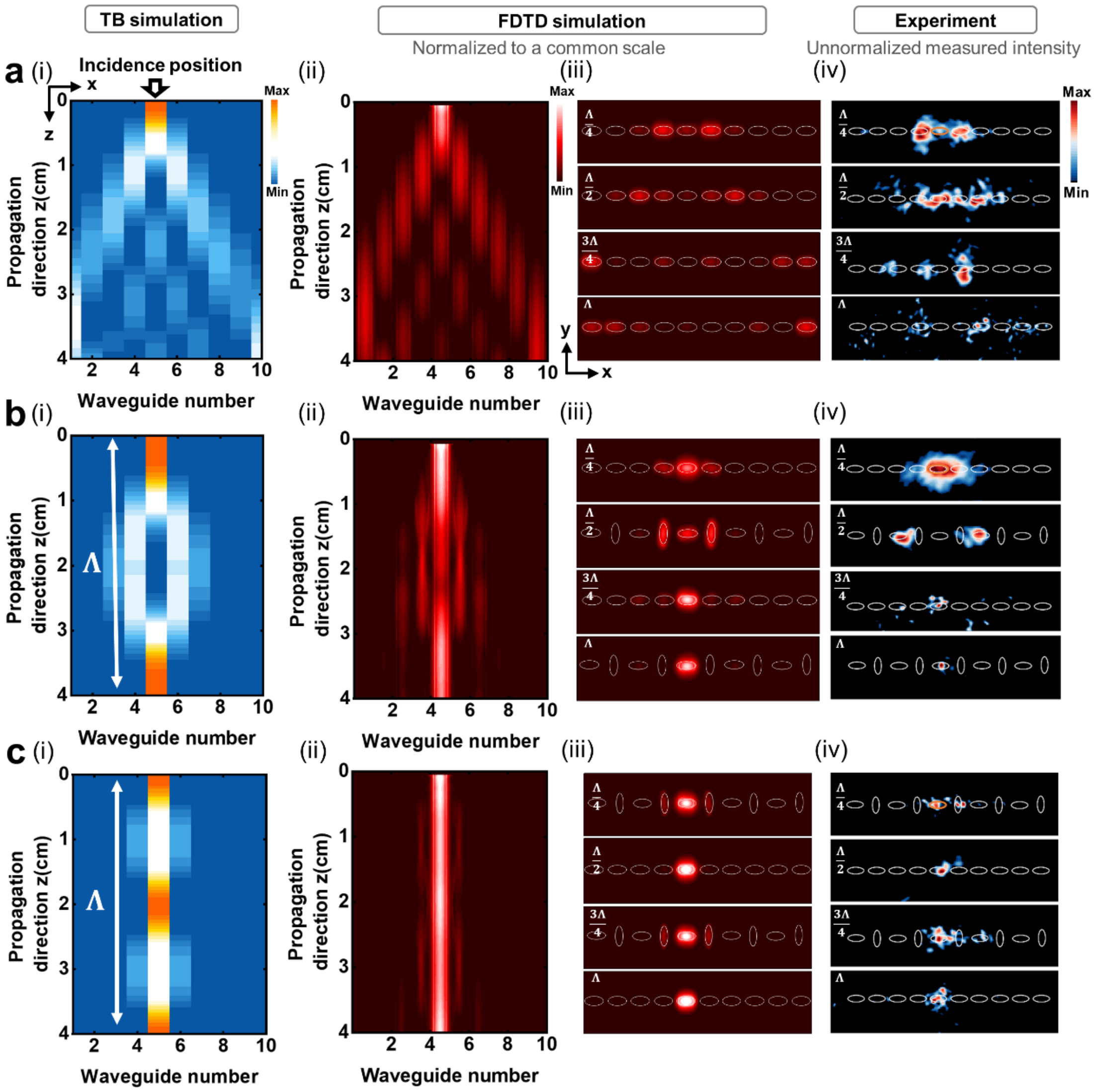

Fig. R1 | Floquet flat band: comparison between TB simulation, FDTD simulation, and THz-TDS experiment. a–c, Results for three representative cases: **a,** discrete diffraction in a straight lattice for comparison; **b,** Floquet flat band under modulation of $\kappa = \kappa_0 \sin(\theta(z))$; **c,** Floquet flat-band dynamics under modulation of $\kappa = \kappa_0 \cos(\theta(z))$. **(i)** Tight-binding (TB) simulation of the intensity evolution viewed along the propagation direction. **(ii)** Corresponding FDTD simulation of the intensity evolution in the same view. **(iii)** Transverse cross-sectional intensity distributions from FDTD simulation at selected propagation distances $z = \frac{\Lambda}{4}, \frac{\Lambda}{2}, \frac{3\Lambda}{4}$, and Λ. **(iv)** Corresponding cross-sectional intensity distributions measured in the THz-TDS system. The agreement between TB simulation, FDTD simulation, and THz-TDS experiment confirms the flat-band induced by the Floquet engineering.

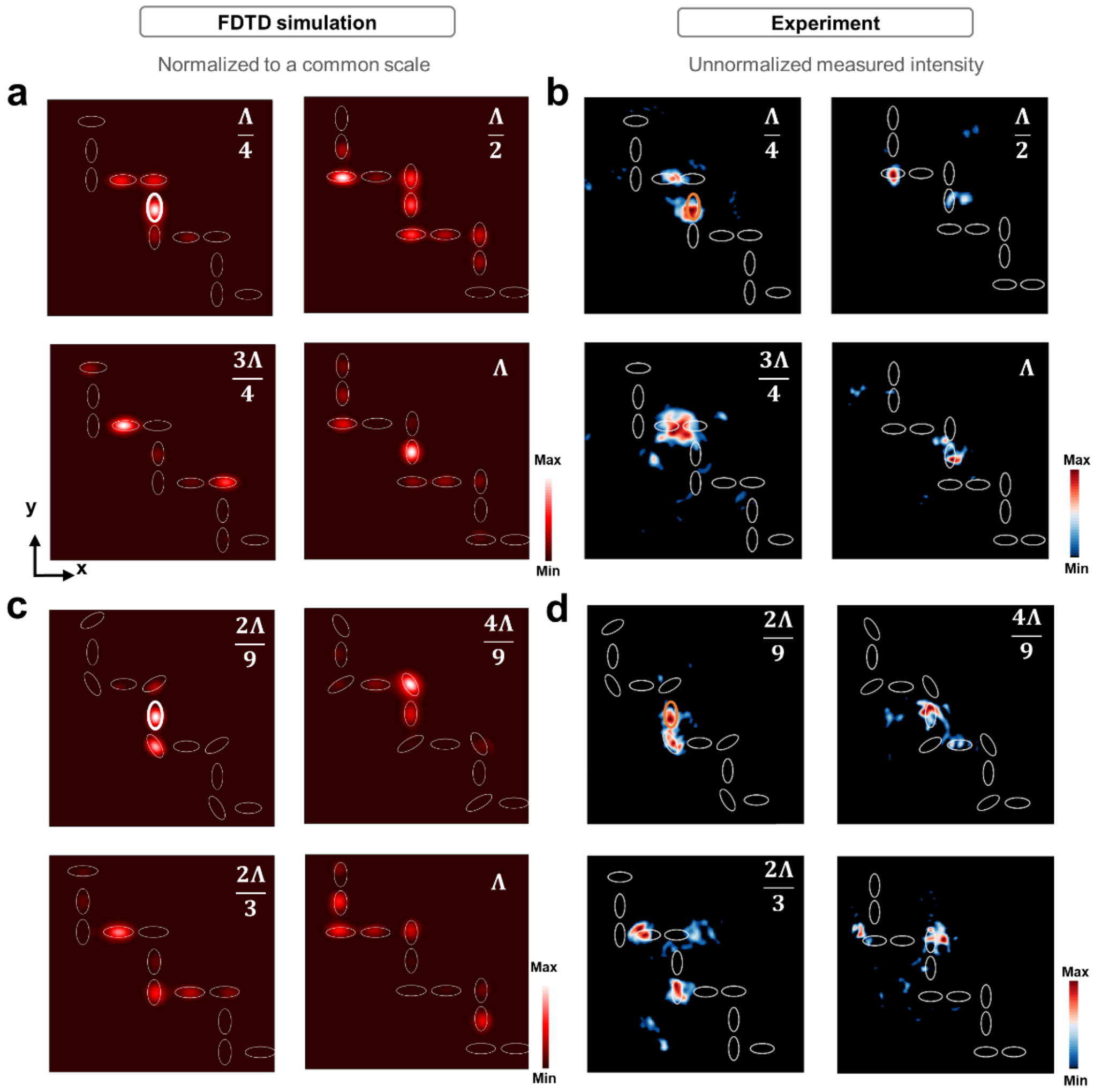

Fig. R2 | Period-Λ and Period-2Λ oscillations: comparison between FDTD simulation and experiment. a, b, Cross-sectional intensity distributions from FDTD simulation (**a**) and the corresponding experimental measurements (**b**) for the period-Λ oscillation. At $z = \Lambda$, the optical field returns to the initially excited waveguide. **c, d,** Cross-sectional intensity distributions from FDTD simulation (**c**) and the corresponding experimental measurements (**d**) for the period-2Λ oscillation. At $z = \Lambda$, the optical field does not return to the initially excited waveguide.

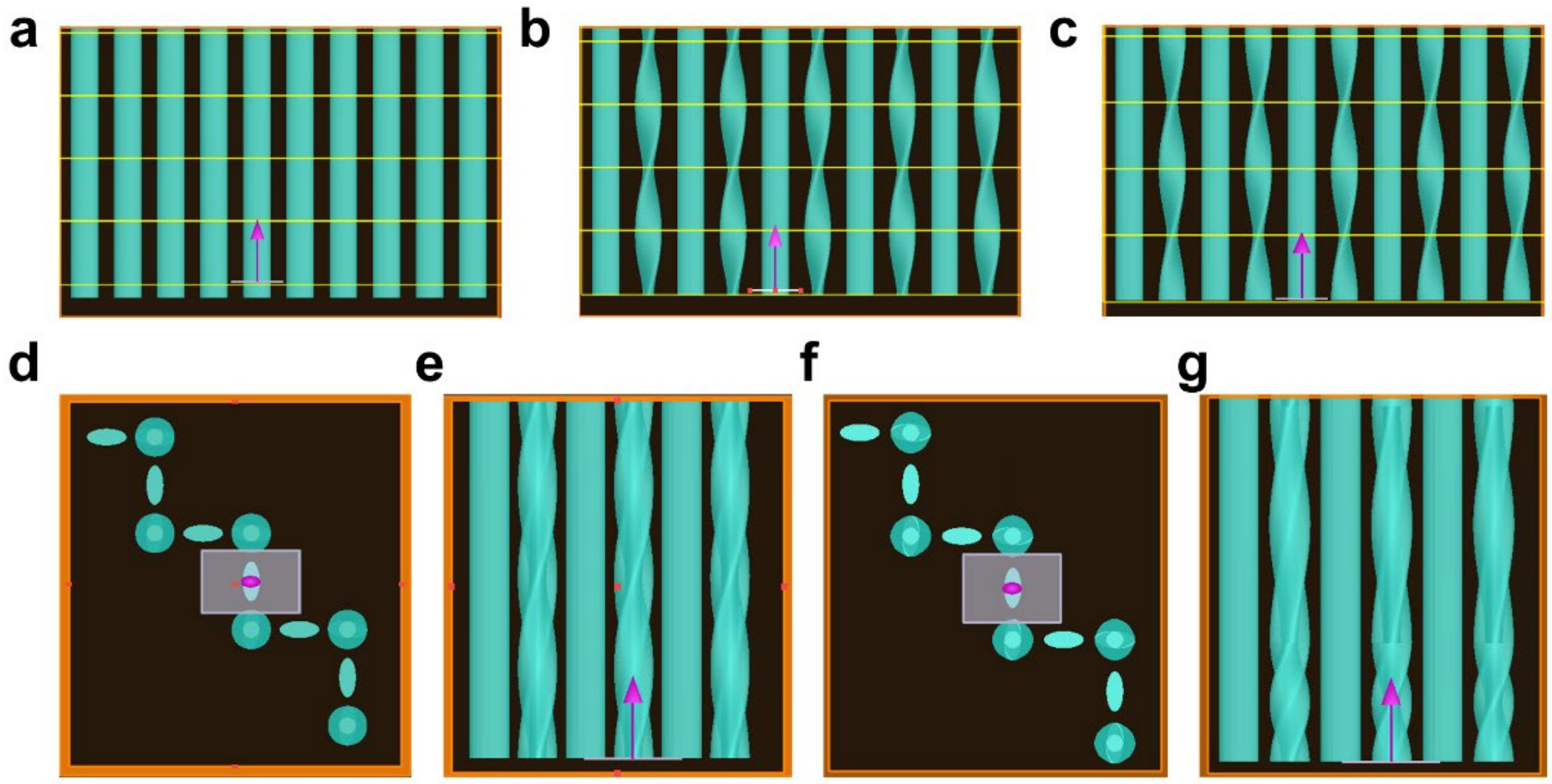

Fig. R3 | FDTD models corresponding to the structures used in the main-text simulations. a–c, Side views of the one-dimensional waveguide arrays used for the FDTD simulations in Fig. 3: **a,** straight lattice for the discrete-diffraction control case; **b,** twisted lattice realizing the Floquet flat band under modulation of $\kappa = \kappa_0 \sin(\theta(z))$; **c,** twisted lattice realizing the Floquet flat-band dynamics under modulation of $\kappa = \kappa_0 \cos(\theta(z))$. **d–g,** FDTD models for the period-Λ and period-2Λ oscillation structures in Fig. 4. **d, e,** Cross-sectional view and side view, respectively, of the waveguide array for the period-Λ oscillation. **f, g,** Cross-sectional view and side view, respectively, of the waveguide array for the period-2Λ oscillation. The magenta arrows indicate the input excitation direction.

After confirming the agreement among TB, FDTD, and experiment, we further used FDTD simulations to separate the influence of the twisted geometry from that of material absorption. Most of these simulations use the same real part of the refractive index as in the experiments while setting the imaginary part to zero, so as to isolate the effect of the structure itself. These simulations show that the twisting geometry introduces nearly no additional loss. In particular, Fig. R4 uses the experimentally measured complex refractive index, including the material absorption. This result shows that the absorption mainly reduces the overall signal amplitude, but does not change the essential light evolution or the physical phenomena demonstrated in our work. Together, these simulations confirm that the observed loss is dominated by material absorption rather than by the twisted geometry, and that the underlying physics

remains robust under realistic experimental conditions.

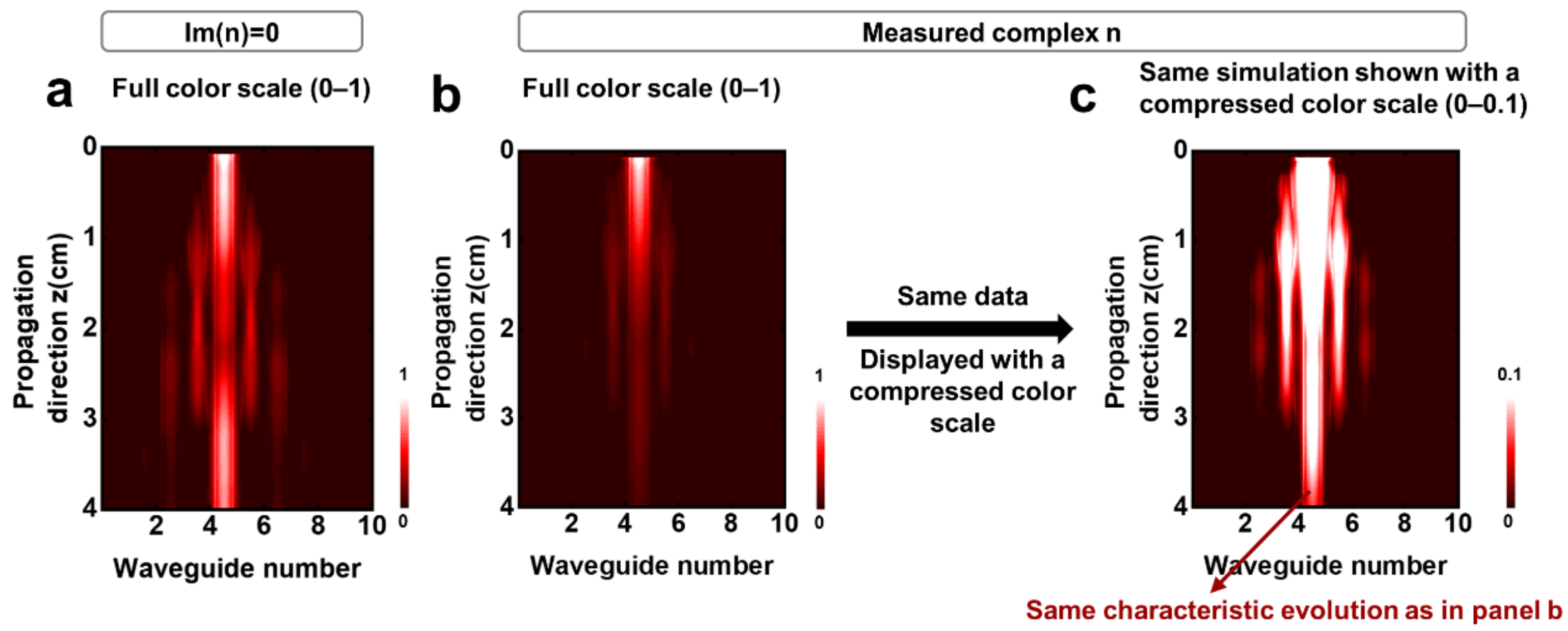

Fig. R4 | FDTD simulation with the measured material absorption. a, Intensity evolution in the flat band sample with coupling modulation $\kappa = \kappa_0 \sin(\theta(z))$, simulated using the refractive index with Im(n)=0, such that material absorption is neglected. **b, c,** Intensity evolution in the flat band sample with coupling modulation $\kappa = \kappa_0 \sin(\theta(z))$, simulated using the experimentally measured complex refractive index. **b,** Full color scale (0–1). **c,** The same result shown with a compressed color scale (0–0.1). The two panels present the same simulation data with different display ranges. The comparison shows that material absorption mainly reduces the overall signal strength, without affecting the physical phenomenon being verified.

II. We added new experiments using the THz-VNA system with higher source power and higher signal-noise-ratio. The new results are consistent with the original TDS measured results, as well as with the TB and FDTD simulated results.

To further strengthen the experimental evidence, we replaced the original experimental results in Figs. 3 and 4 of the main text with new measurements performed using the THz-VNA system. Compared with the original THz-TDS system, the THz-VNA employs electrically generated terahertz signals, which provide a stronger incident terahertz field than the optically generated signals used in THz-TDS. In addition, the THz-VNA benefits from coherent detection: the received signal is mixed with a phase-locked reference at the same frequency, which selectively extracts the coherent response and efficiently suppresses incoherent noise contributions. In addition, the narrow intermediate-frequency bandwidth and high dynamic range of the VNA

receiver enable more reliable detection of weak transmitted signals. Under our measurement conditions, signals as weak as approximately −100 dB can still be reliably resolved, so even for the longest samples, whose transmitted signals are strongly attenuated by material absorption, the output can still be detected clearly. As a result, the THz-VNA measurements exhibit a lower noise floor and more visually distinct output intensity distributions. Importantly, the newly measured results are consistent not only with the previous THz-TDS data, but also with the TB calculations and full-wave FDTD simulations. At the same time, the THz-VNA measurements exhibit much clearer and more intuitive output intensity distributions. These new results therefore provide a stronger and more complete experimental validation of the theoretical analysis and numerical simulations presented in this work. The experimental platform used for these repeated measurements is shown in Fig. R5. Based on this THz-VNA system, we remeasured the one-dimensional Floquet flat band samples and the straight lattice control sample, with the corresponding comparison between FDTD simulations and unnormalized experimental intensities shown in Fig. R6.

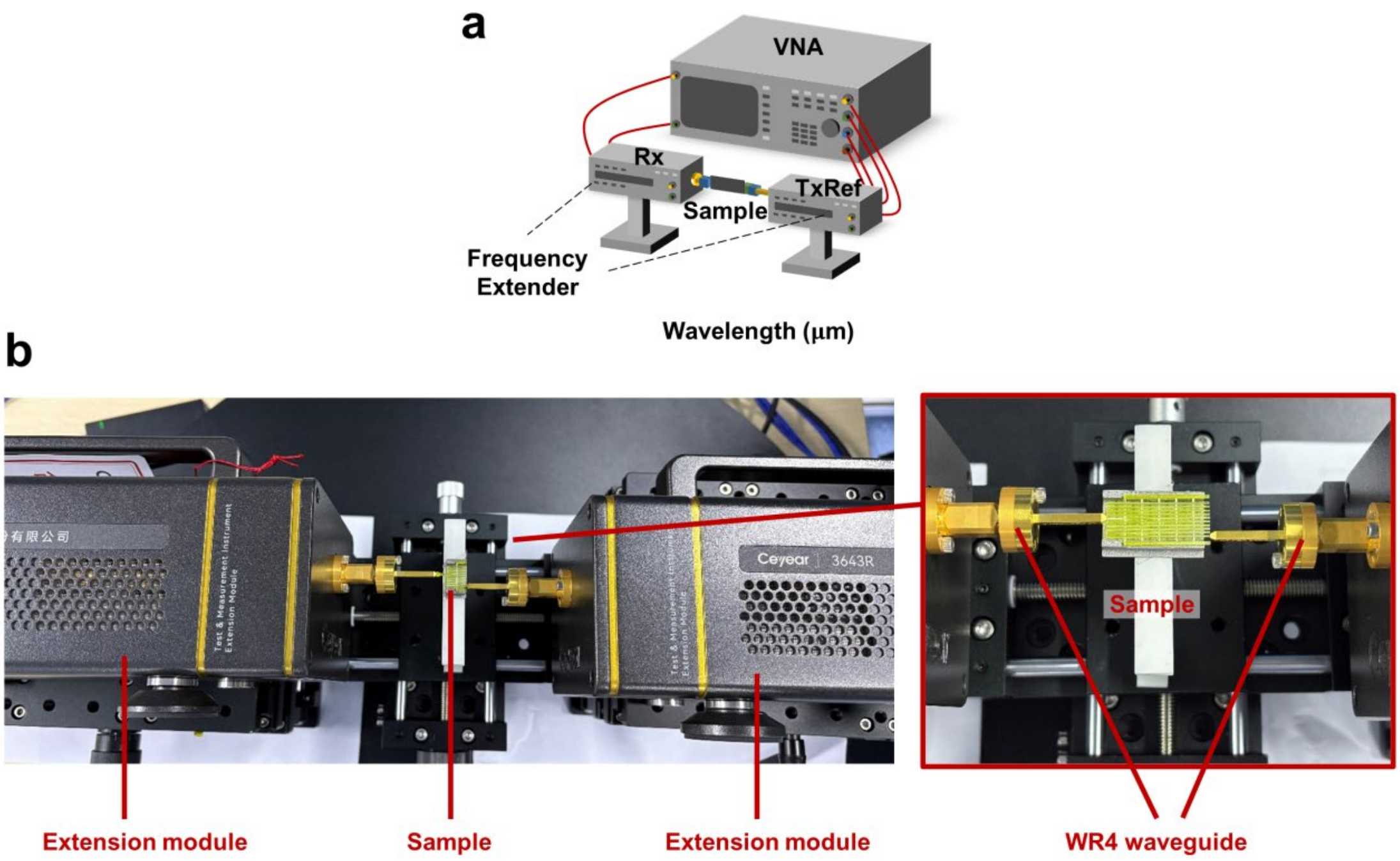

Fig. R5 | THz-VNA measurement setup for the repeated experiments. a, Schematic of the THz-VNA measurement system, consisting of a VNA, two frequency extension modules, and the sample

placed between them. The TxRef module serves as the transmitting and reference port for terahertz signal generation and coherent detection, whereas the Rx module receives the transmitted terahertz signal. **b,** Photograph of the corresponding experimental setup. The sample is mounted between the transmitting and receiving extension modules, and the inset shows an enlarged view of the sample region. Two WR4 metallic waveguides are connected to the sample to couple the terahertz signal into the structure and collect the transmitted output.

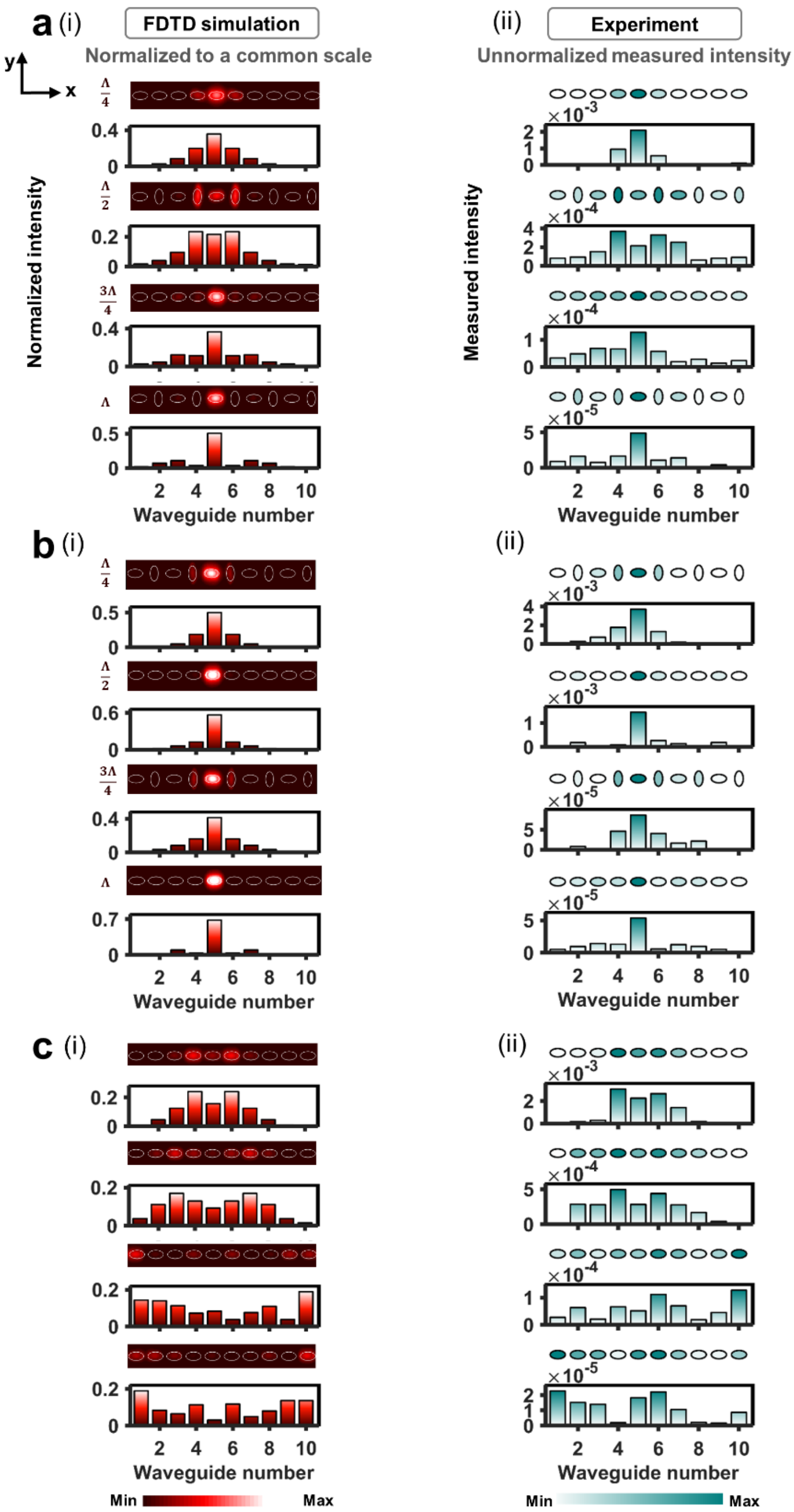

Fig. R6 | Comparison between FDTD simulations and experimental measurements for the one-dimensional lattices. a, b, Floquet flat-band dynamics under modulation of $\kappa = \kappa_0 \sin(\theta(z))$ and $\kappa = \kappa_0 \cos(\theta(z))$, respectively. **c,** Discrete diffraction in the straight-lattice control sample. **(i)** Output intensity distributions extracted from the finite-difference time-domain (FDTD) simulations at propagation distances $\frac{\Lambda}{4}$, $\frac{\Lambda}{2}$, $\frac{3\Lambda}{4}$, and Λ, normalized to a common scale. **(ii)** Corresponding experimentally measured output average intensity distributions over five measurement counts at the same propagation distances, shown as directly measured, unnormalized intensities.

We also applied the same measurement procedure to the period-nΛ oscillation samples. As summarized in Fig. R7, the measured output distributions reproduce the expected period-Λ and period-2Λ behavior and agree well with the corresponding FDTD simulations, further supporting the robustness of the observed Floquet dynamics.

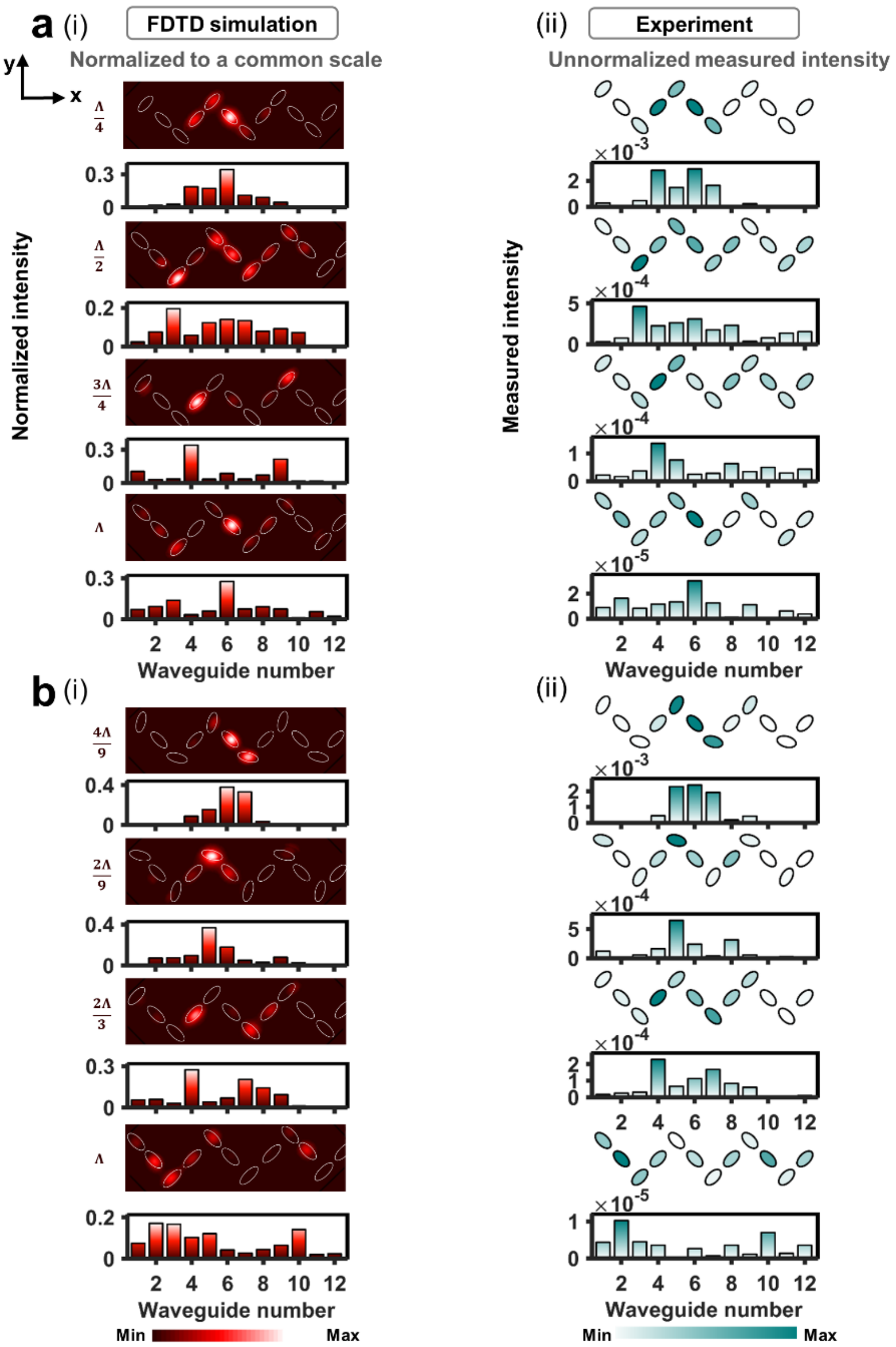

Fig. R7 | Comparison between FDTD simulations and experimental measurements for the period-nΛ oscillation structures. a, Period-Λ oscillation. **b,** Period-2Λ oscillation. **(i)** Output intensity distributions extracted from the finite-difference time-domain (FDTD) simulations at selected propagation distances, normalized to a common scale. **(ii)** Corresponding experimentally measured average output intensity distributions over five measurement counts at the same propagation distances, shown as directly measured, unnormalized intensities.

III. We clarified the physical origin of the loss. Our analysis and experiments show that any additional loss induced by the twisting is negligible, and that the dominant loss mainly arises from the material absorption of the resin in the terahertz band.

We have clarified the physical origin of the propagation loss in the revised

manuscript. In our design, the waveguides propagate along straight trajectories, while only their elliptical cross-sections twist continuously along the propagation direction. We understand the reviewer's concern as whether the twisting cross-section may induce additional radiation loss or mode mismatch. To address this point, we first characterized the material response of the RG resin and the propagation loss of straight elliptical waveguides, as shown in Fig. R8. The results show that the RG resin exhibits relatively low absorption loss in the 0.2–1.3 THz frequency range. The measured absorption coefficients of the straight elliptical RG waveguides are 5.56 dB/cm, 6.94 dB/cm, and 7.46 dB/cm at 0.2 THz, 0.25 THz, and 0.3 THz, respectively. We then characterized twisted elliptical waveguides with different propagation lengths in Fig. R9. The close fitted loss coefficients indicate that the twist itself introduces little additional loss, whereas the dominant loss originates from material absorption under the terahertz band. In particular, Fig. R9g shows that straight and twisted waveguides with the same propagation length exhibit very similar output signal intensities, further confirming that the dominant loss mainly arises from material absorption rather than the twisted geometry itself.

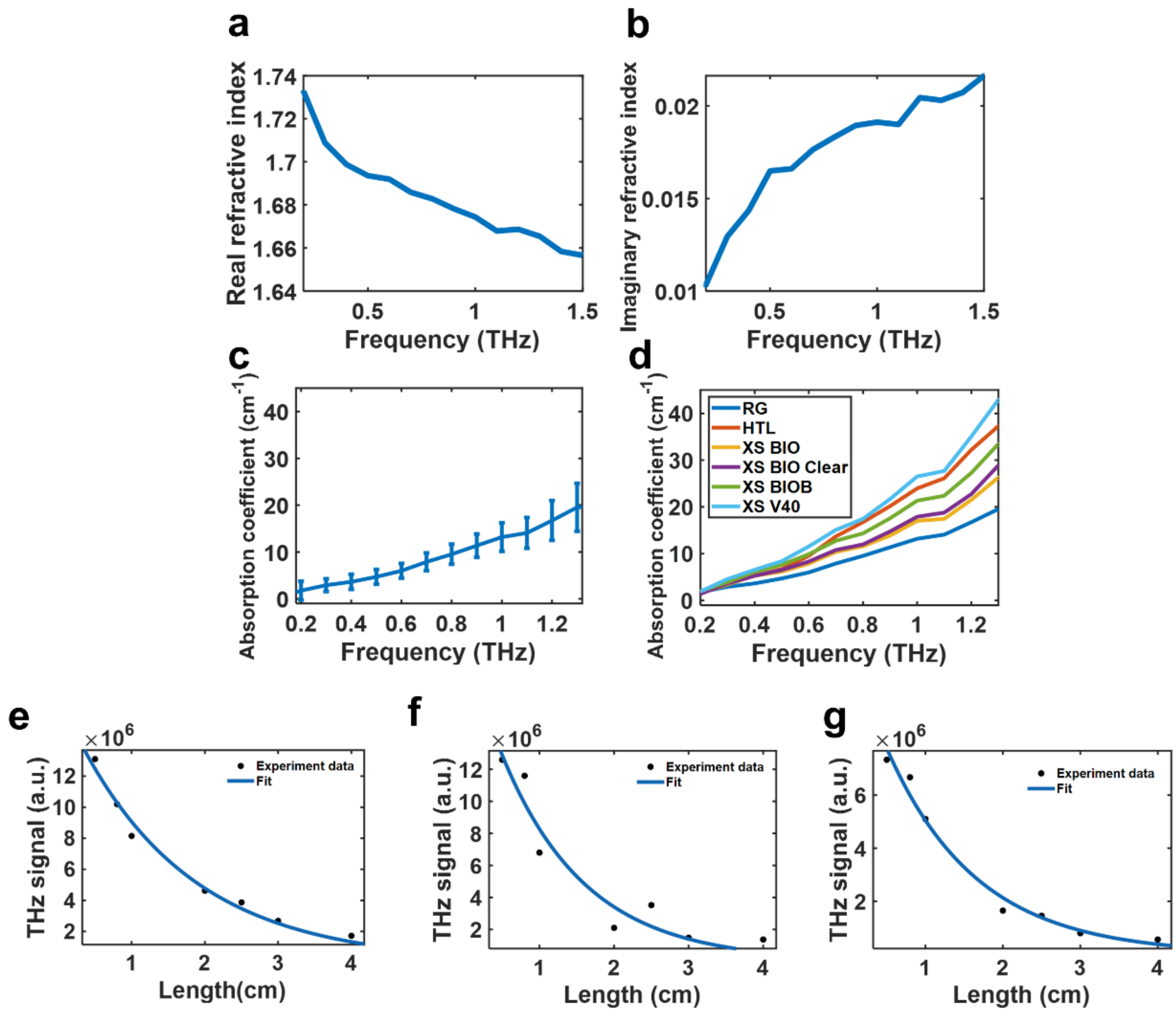

Fig. R8 | Terahertz characterization of RG resin films and elliptical straight waveguides. a, Real part of the refractive index of a 1-mm-thick RG resin film obtained from terahertz time-domain spectroscopy. **b**, Imaginary part of the refractive index of the same film. **c**, Absorption coefficient of the RG resin extracted from the THz-TDS measurements, showing values consistent with previously reported absorption coefficients of resin-based materials in the terahertz band[11]. **d**, Comparison of the absorption coefficients of several candidate resins measured by THz-TDS at different frequencies, showing that RG exhibits the lowest absorption among the tested materials and is therefore selected for sample fabrication. **e–g**, Measured output signals of elliptical waveguides with cross-sectional axes $a = 350$ μm and $b = 150$ μm at different propagation lengths. The corresponding absorption coefficients are 1.28 cm^{-1} (5.56 dB/cm), 1.598 cm^{-1} (6.94 dB/cm), and 1.718 cm^{-1} (7.46 dB/cm) for panels c-e, corresponding to frequencies of 0.2 THz, 0.25 THz, and 0.3 THz, respectively.

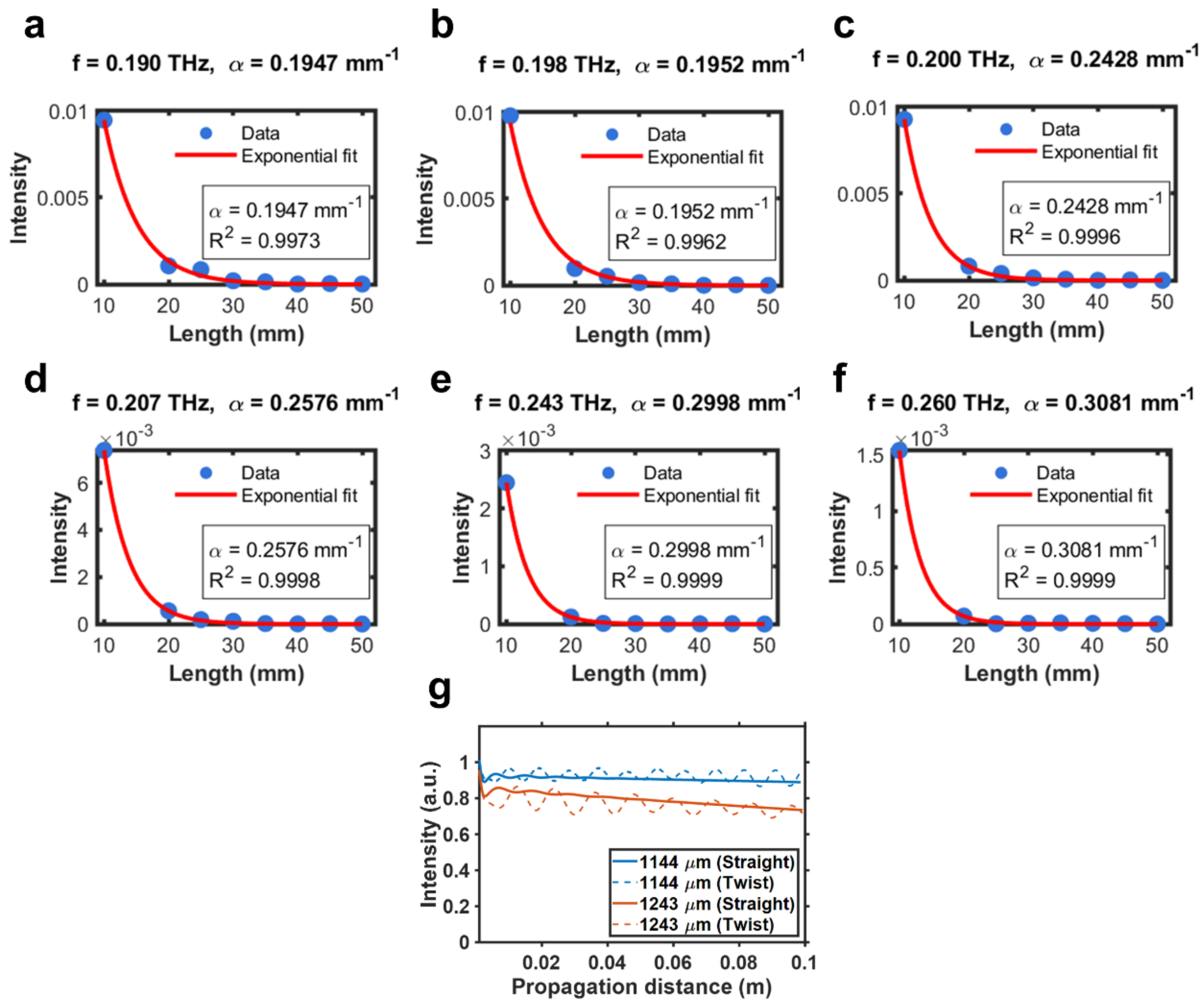

Fig. R9 | Loss characterization of twisted elliptical waveguides and comparison with straight waveguides. a–f, Measured transmitted intensities of twisted elliptical waveguides with different propagation lengths, obtained using the THz-VNA system at different frequencies. Exponential fits give the corresponding absorption coefficients. The fitted values are close to those obtained for straight waveguides, indicating that the twist does not introduce significant additional loss. The slightly higher loss in the twisted samples likely arises from increased surface roughness introduced during high-precision 3D printing of the twisted structures. **g,** FDTD-simulated intensity evolution in straight and twisted waveguides at two representative wavelengths. The simulations show that the twist introduces nearly no additional loss. The stronger absorption at longer wavelengths mainly results from weaker field confinement in the waveguide. Together with the material characterization, these results show that although the absorption coefficient of the bulk resin film increases with frequency, the effective loss in a specific elliptical waveguide also depends on the field confinement in the waveguide, so no simple monotonic relation between loss and frequency can be directly inferred.

IV. We added quantitative loss measurements for different propagation lengths, showing that even in the earlier THz-TDS system, the transmitted signal from the longest sample remains well above the noise floor.

To address whether the original THz-TDS measurements were close to the noise floor, Fig. R10 compares the transmitted signal from the longest sample with both the reference beam spot and the background noise. The comparison shows that the transmitted signal through the sample remains on the order of 10^6, whereas the pure background noise level without the sample and without the THz source is on the order of 10^5. Therefore, even for the longest sample with a length of 4 cm, the measured signal remains well above the noise floor of the THz-TDS system.

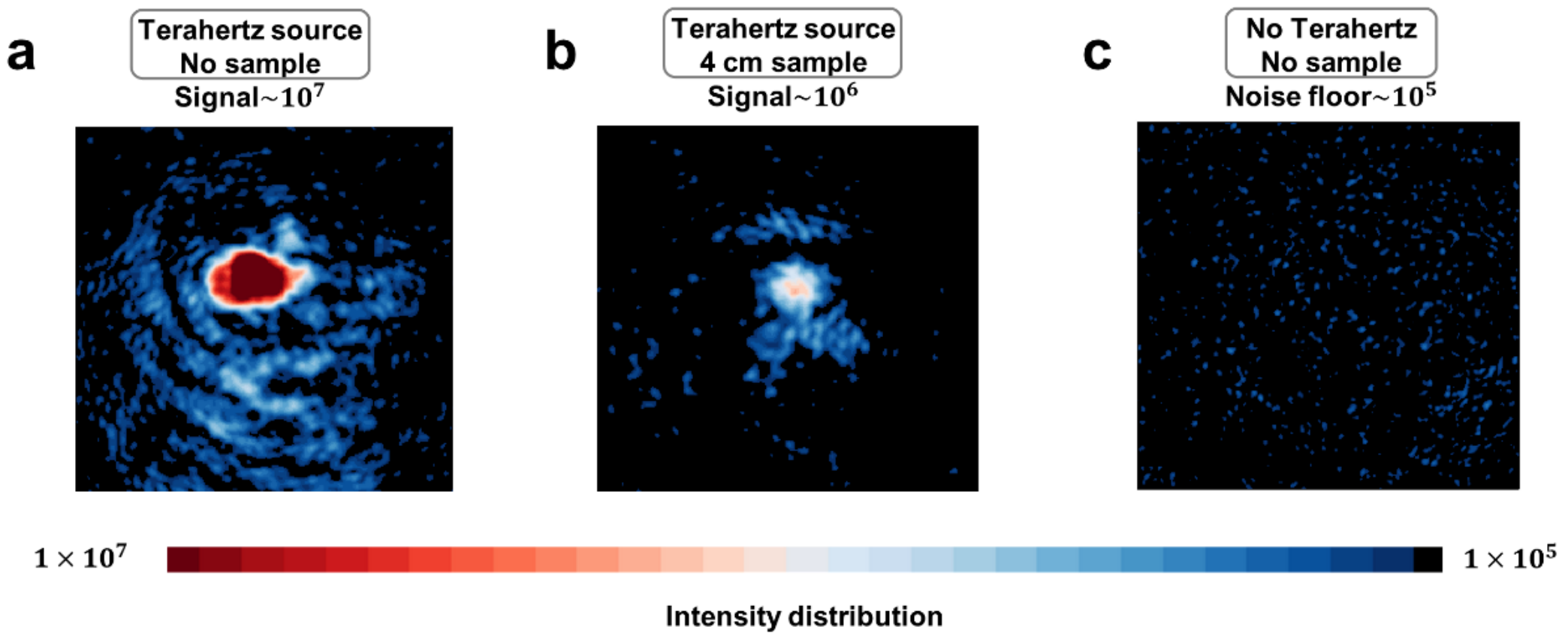

Fig. R10 | Comparison between the experimental transmitted terahertz signal from the longest sample and the noise floor in the THz-TDS system. a, Maximum terahertz beam spot recorded in the THz-TDS system without any sample, with a signal level on the order of 10^7. **b,** Output intensity distribution of the longest sample (4 cm), with a signal level on the order of 10^6. **c,** Background noise image recorded without terahertz source and without any sample, with a signal level on the order of 10^5. The comparison shows that, even for the longest sample, whose transmitted signal is strongly attenuated by material absorption, the detected output intensity remains more than one order of magnitude higher than the noise level.

Finally, we also note that the relatively large absorption in the present experiments is mainly related to the limited material choices currently compatible with high-precision 3D printing at this scale. As fabrication technologies continue to develop,

lower-loss printable materials are expected to become available, which should further improve the experimental performance of this platform.

Accordingly, in the revised manuscript and supplementary materials we have added:

I. additional beam-propagation simulations to verify the correctness of the theoretic analysis;

II. a new set of higher-quality experimental measurements based on the THz-VNA platform;

III. a clearer discussion of the physical origin of the propagation loss both experimentally and numerically;

IV. quantitative loss characterization for different propagation lengths experimentally.

Changes in the manuscript:

Main text:

In the revised manuscript, we have introduced the two measurement platforms in **Fig. 2a** and in the first paragraph of the section "**Theoretical Model of Polarization-Controlled Coupling in Twisted Waveguides**", where we now clarify that the coupling-characterization measurements are performed using THz-TDS, while the key Floquet experiments are remeasured using THz-VNA. The new THz-VNA results exhibit much clearer and less noise-affected output intensity distributions, while remaining fully consistent with the previous THz-TDS data and with the TB and FDTD simulations.

"Experiments are performed using two complementary terahertz measurement platforms (Fig. 2a). The coupling coefficient as a function of the relative polarization angle is characterized by terahertz time-domain spectroscopy (THz-TDS) combined with two-dimensional terahertz real-time imaging (Fig. 2a(i)). For the THz-TDS system, a femtosecond laser pumps a Terahertz source, and the transmitted signal through the sample is collected by a balanced detector. The sample is mounted on a motorized XYZ

stage for spatial movement. The measurements of the Floquet flat band and period-n Λ oscillations in Figs. 3 and 4 are carried out on a terahertz vector network analyzer (THz-VNA) platform with frequency extension modules (Fig. 2a(ii)), in which the sample is placed between the transmitting and receiving modules. The TxRef module serves as the transmitting and reference port for terahertz signal generation and coherent detection, whereas the Rx module receives the transmitted terahertz signal. Two WR4 metallic waveguides are connected to the sample to couple the terahertz signal into the structure and collect the transmitted output. Benefiting from swept-frequency coherent detection, the THz-VNA provides a lower noise floor and a higher dynamic range, thereby producing clearer output intensity distributions for the weak transmitted signals involved in these experiments."

Figures:

In the revised manuscript, we have introduced the two measurement platforms in **Fig. 2a.**

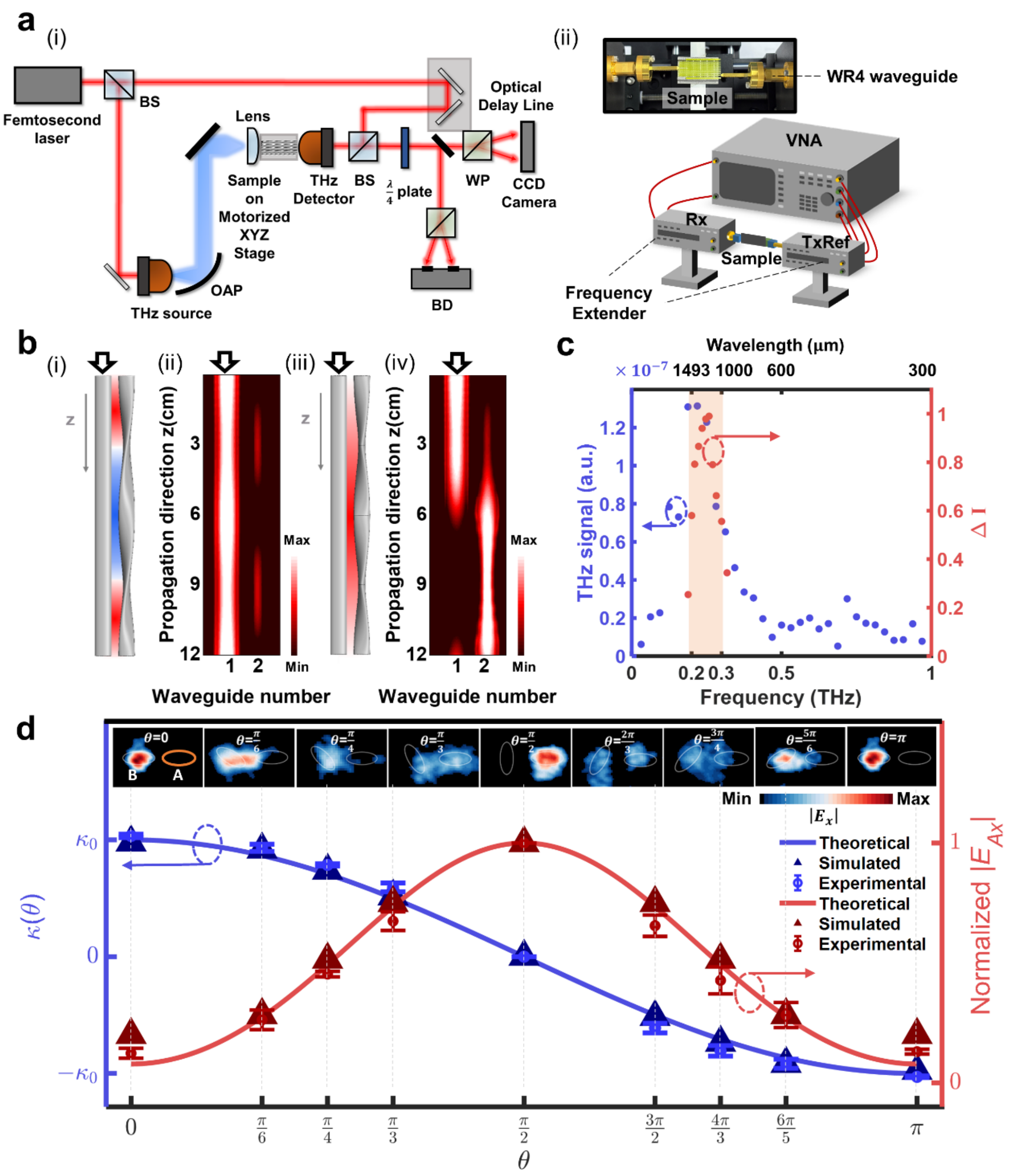

Fig. 2 | ***Experimental verification of polarization-controlled coupling in twisted waveguides. a,*** *Measurement setups.* ***(i)*** *THz-TDS setup. OAP, off-axis parabolic mirror; BS, beam splitter; BD, balance detector; WP: Wollaston prism.* ***(ii)*** *THz-VNA setup with two frequency extension modules and the sample placed between them. Inset: photograph of the setup.* ***b,*** *FDTD simulations of light propagation in two coupled elliptical waveguides.* ***(i–ii)***, *one waveguide is twisted by* 2π *along the propagation direction, yielding* $\kappa(z) = \kappa_0 cos\theta(z)$. ***(iii–iv)***, *the twisted waveguide undergoes alternating rotations of* $\pm\frac{\pi}{2}$, *yielding* $\kappa(z) = \kappa_0|cos\theta(z)|$. ***c,*** *TE mode spectrum of a single waveguide and operational bandwidth determined from the relative output intensity variation*

$\Delta I(\lambda)$. Operational bandwidth: 1000 μm to 1493 μm. ***d,*** *Experimental, simulated and theoretical results for nine pairs of waveguides with different θ. Top: measured output field distribution ($|E_{Ax}|$) after 1 cm of propagation, with normalized intensity plotted as a function of θ. Orange dashed circles highlight the injected waveguides. Bottom: Extracted coupling coefficient $\kappa(\theta)$. Solid lines denote theory; points denote simulations and experiments. Error bars indicate standard deviations.*

We repeated the key main-text experiments using a THz-VNA platform and **replaced the corresponding experimental results in the figs. 3 and 4 with the new measurements**.

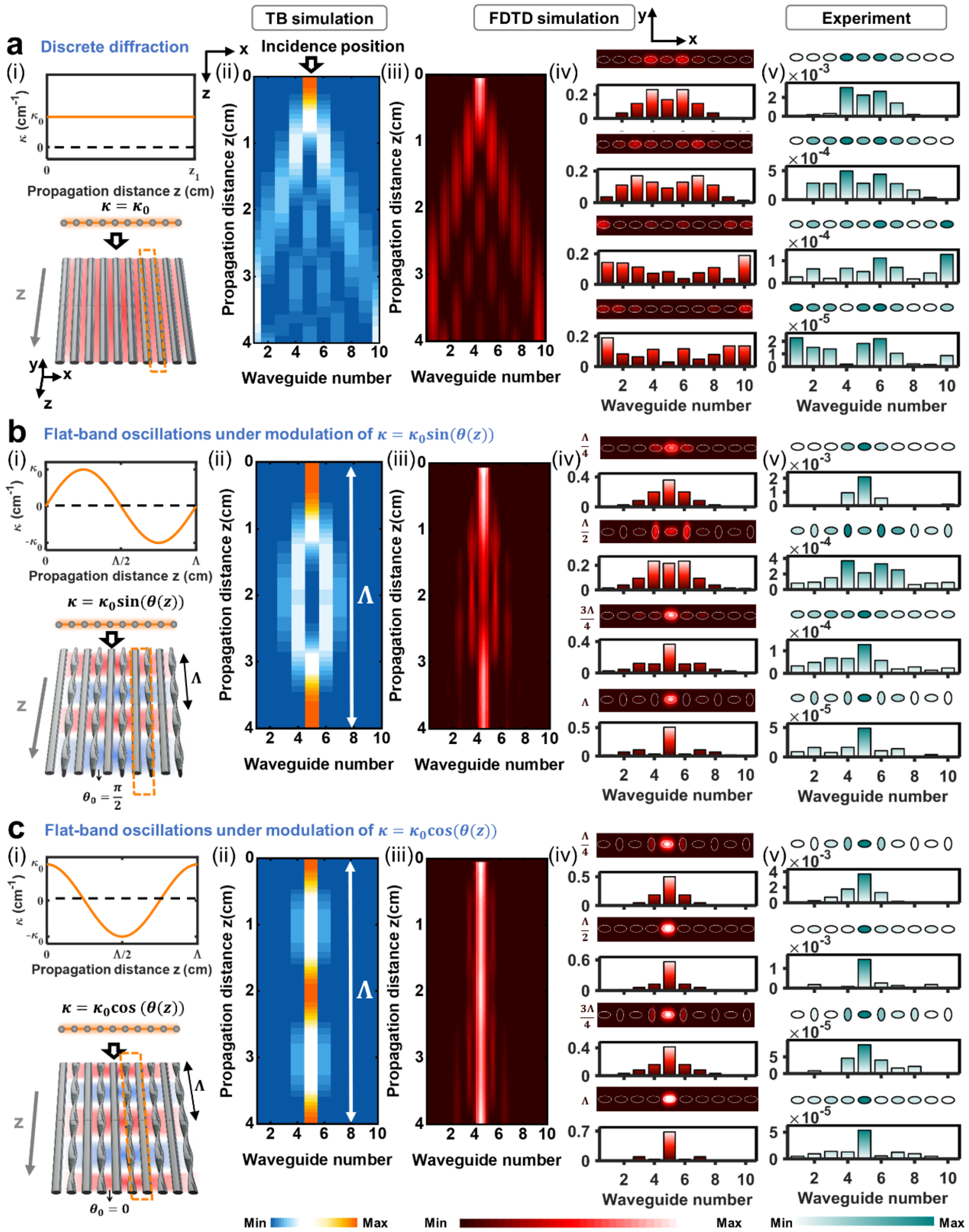

Fig. 3 | Floquet flat bands enabled by coupling modulation. Ten waveguides spaced by 1100 μm form an array with propagation distance of $\Lambda = 4$ cm ***a,*** *Discrete diffraction in a straight array with propagation-invariant coupling $\kappa = \kappa_0$* ***b,*** *Floquet flat-band dynamics under $\kappa = \kappa_0 sin(\theta(z))$.* ***c,*** *Floquet flat-band dynamics under $\kappa = \kappa_0 cos(\theta(z))$. In the left column, the orange dashed box highlights the coupling corresponds to the function plotted above in panel* ***(i)****. For each case, panel* ***(i)*** *shows the coupling modulation and waveguide geometry;* ***(ii)*** *and* ***(iii)*** *show TB and*

*FDTD simulations; and **(iv)** and **(v)** show FDTD and experimental output intensity distributions at selected propagation distances $\frac{\Lambda}{4}$, $\frac{\Lambda}{2}$, $\frac{3\Lambda}{4}$, and Λ. Light is injected into the central waveguide. The FDTD results use a common normalization scale, whereas the experimental bar plots show directly measured, unnormalized intensities.*

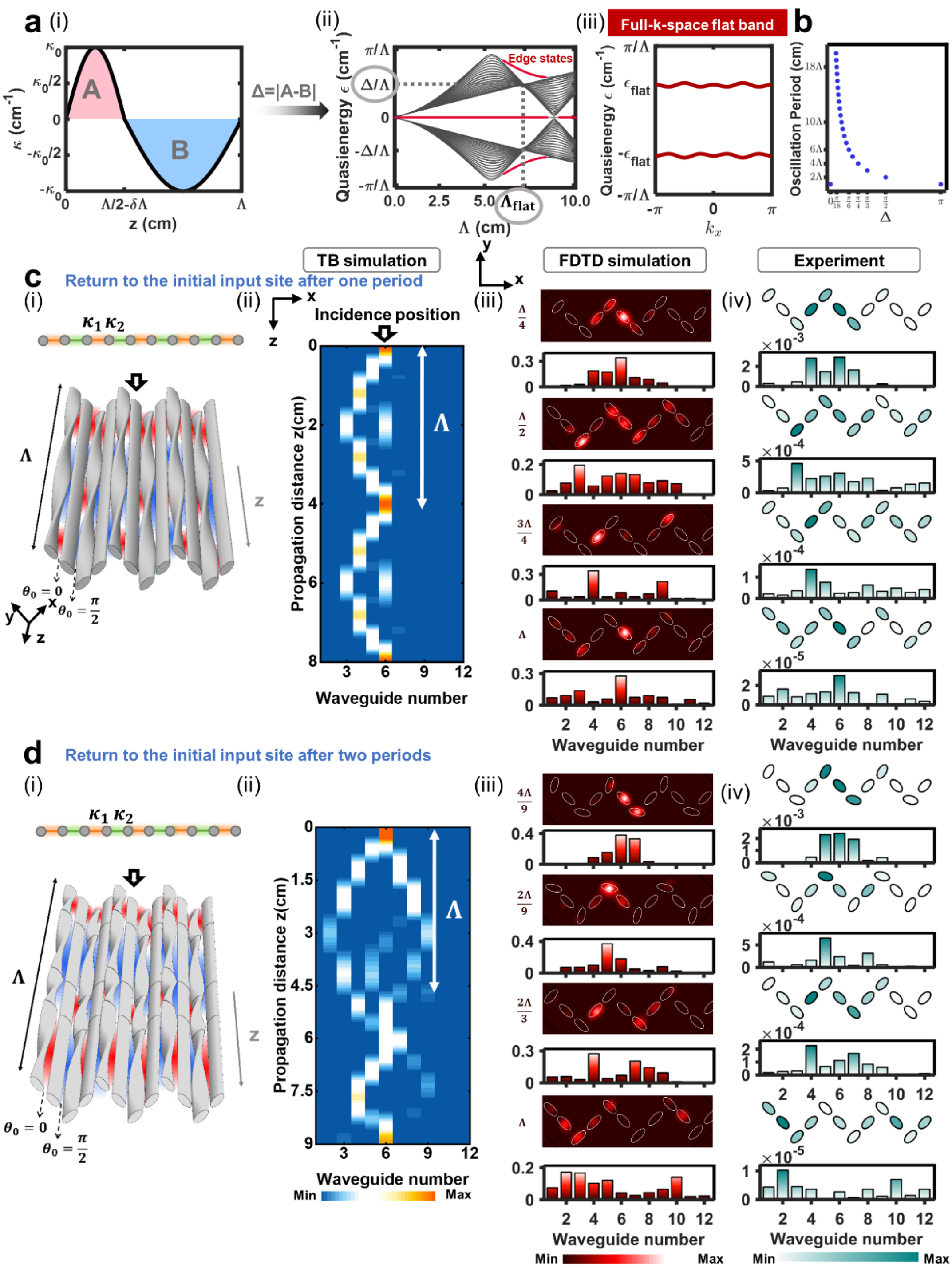

***Fig. 4 | Period-$n\Lambda$ oscillations. a**, Coupling modulation with different twisting rates. **(i)** Regions A*

and B correspond to different twisting rates within one driving period. ***(ii)*** *The corresponding quasi-energy spectrum under open boundary conditions, exhibits flat bands at* Λ_{flat}*.* ***(iii)*** *Quasi-energy spectrum under periodic boundary conditions under* Λ_{flat} *shows flat bands spanning the entire Brillouin zone with the value of* Δ/Λ*.* ***b****, Oscillation period as a function of* Δ*.* ***c, d,*** *Experimental demonstration of period-*$n\Lambda$ *oscillations for* $n = 1$ *and* $n = 2$*, respectively. The TB simulations are shown in* ***(ii)****, the FDTD simulations in* ***(iii)****, and the experimental results in* ***(iv)****. The FDTD results are normalized to a common scale across different propagation distances, whereas the experimental bar plots show directly measured, unnormalized output intensities.*

Supplementary materials:

Additional details of the THz-VNA system are provided in **Supplementary Note 11** and **Fig. S11**.

*"****Supplementary Note 11 | Terahertz vector network analyzer.***

Compared with the previous THz-TDS measurements, the results obtained using the vector network analyzer (VNA) with frequency extension modules are substantially clearer and less affected by noise. This improvement mainly arises from the measurement principle of the VNA, which is based on swept continuous-wave excitation and coherent detection. Because the received signal is mixed with a phase-locked reference at the same frequency, the system selectively extracts the signal component that is coherent with the excitation while efficiently suppressing incoherent noise contributions. In addition, the narrow intermediate-frequency bandwidth of the VNA receiver further improves the signal-to-noise ratio and dynamic range. Under our measurement conditions, signals down to approximately -100 dB can still be reliably resolved, which explains the lower noise floor and the more visually distinct experimental results obtained with the VNA-based system."

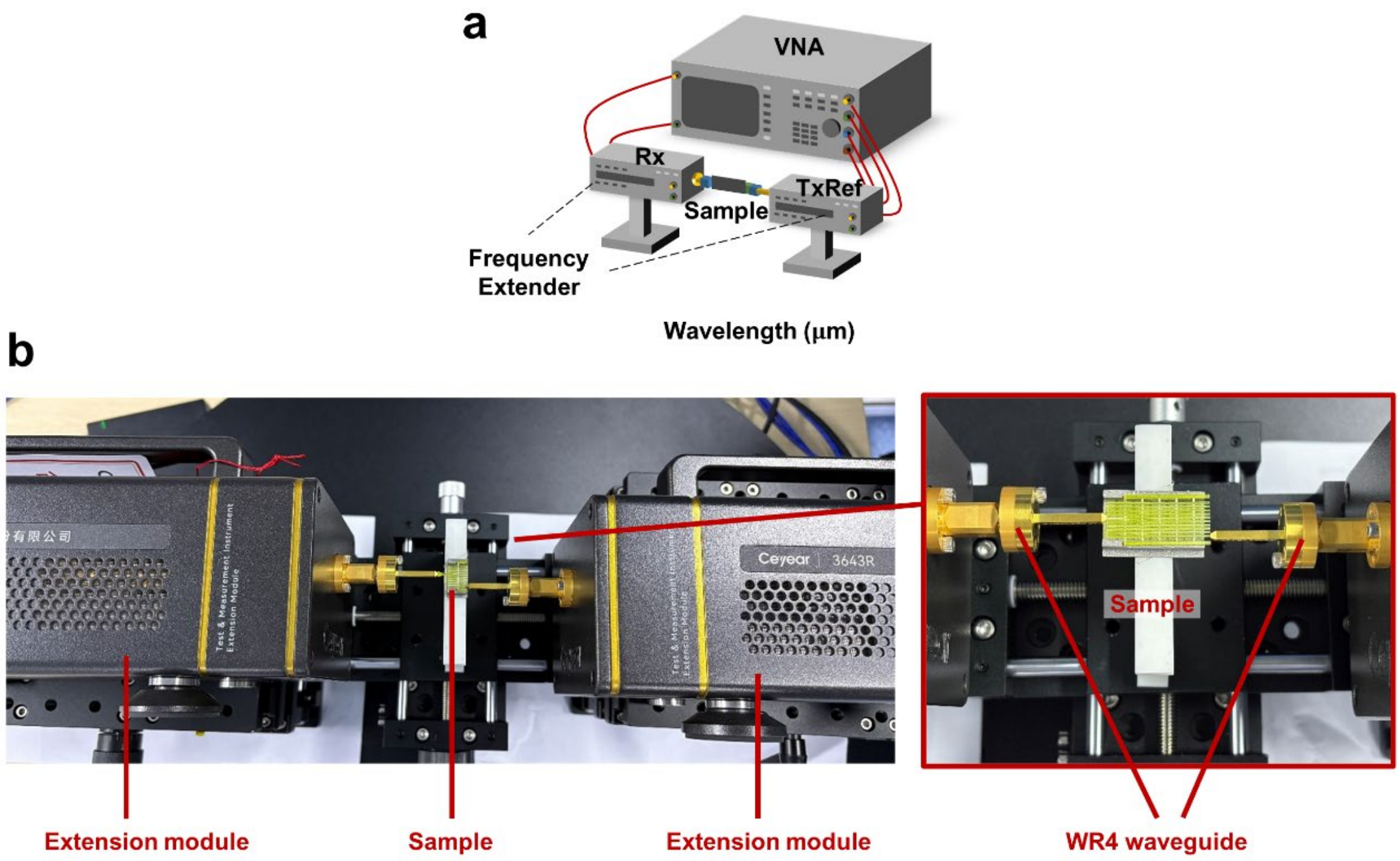

Fig. S11 | THz-VNA measurement setup for the repeated experiments. a, *Schematic of the THz-VNA measurement system, consisting of a VNA, two frequency extension modules, and the sample placed between them. The TxRef module serves as the transmitting and reference port for terahertz signal generation and coherent detection, whereas the Rx module receives the transmitted terahertz signal. b, Photograph of the corresponding experimental setup. The sample is mounted between the transmitting and receiving extension modules, and the inset shows an enlarged view of the sample region. Two WR4 metallic waveguides are connected to the sample to couple the terahertz signal into the structure and collect the transmitted output.*

In addition, to demonstrate that the earlier THz-TDS measurements are fully consistent with the theoretic analysis and FDTD simulations, we include further comparisons in the Supplementary Information: Fig. S25 shows the agreement among TB simulation, FDTD simulation, and the original THz-TDS measurements for the Floquet flat-band dynamics, while Fig. S26 presents the corresponding comparison between simulation and experiment for the period-nΛ oscillations. Together, these results show that the physical phenomena reported in the original submission are reproducible and robust, while the new THz-VNA measurements provide substantially stronger and more compelling experimental support for them.

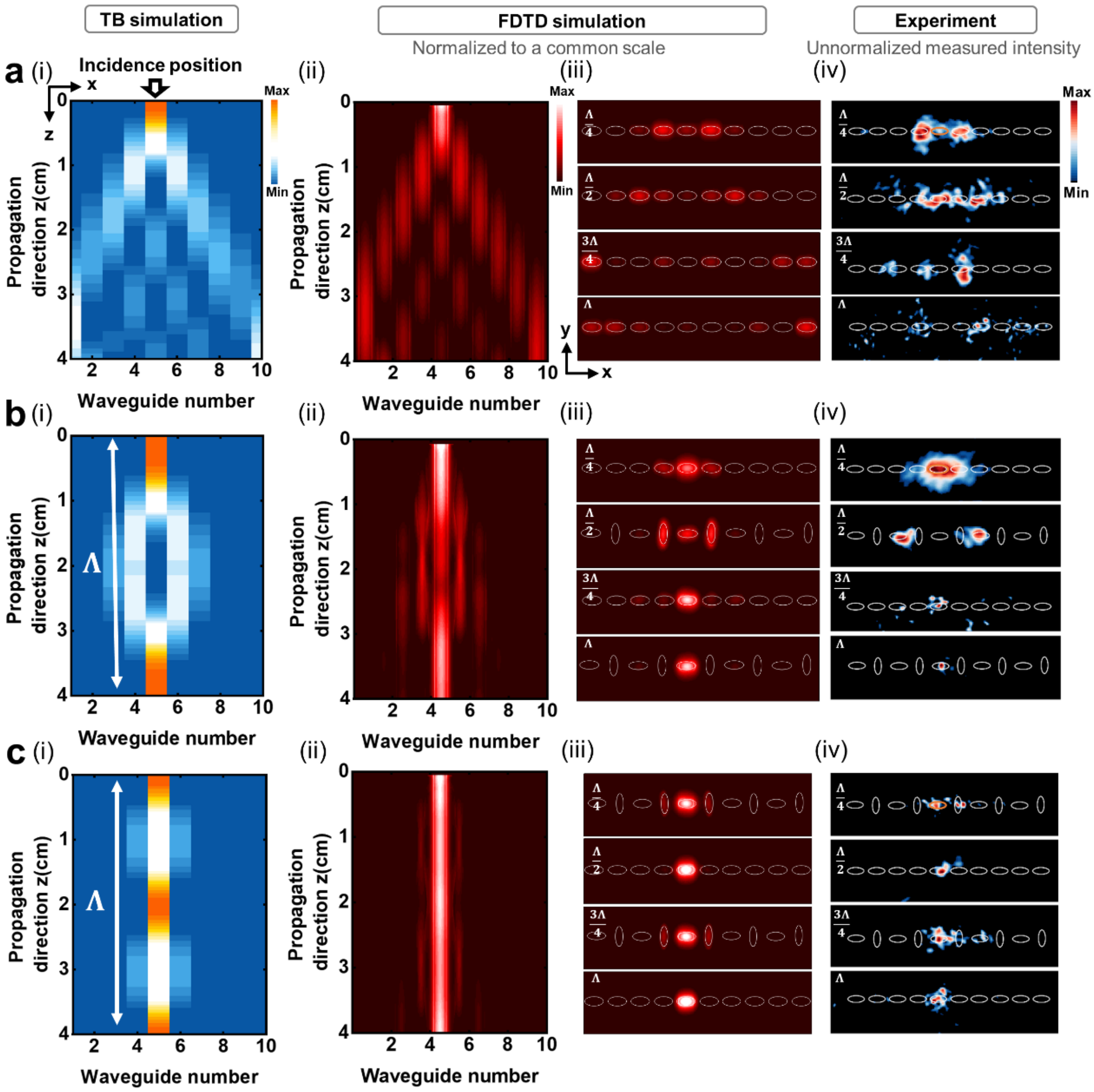

Fig. S25 | Floquet flat band: comparison between TB simulation, FDTD simulation, and THz-TDS experiment. a–c, *Results for three representative cases:* ***a,*** *discrete diffraction in a straight lattice for comparison;* ***b,*** *Floquet flat band under modulation of $\kappa = \kappa_0 sin(\theta(z))$;* ***c,*** *Floquet flat-band dynamics under modulation of $\kappa = \kappa_0 cos(\theta(z))$.* ***(i)*** *Tight-binding (TB) simulation of the intensity evolution viewed along the propagation direction.* ***(ii)*** *Corresponding FDTD simulation of the intensity evolution in the same view.* ***(iii)*** *Transverse cross-sectional intensity distributions from FDTD simulation at selected propagation distances $z = \frac{\Lambda}{4}, \frac{\Lambda}{2}, \frac{3\Lambda}{4}$, and Λ.* ***(iv)*** *Corresponding cross-sectional intensity distributions measured in the THz-TDS system. The agreement between TB simulation, FDTD simulation, and THz-TDS experiment confirms the flat-band induced by the Floquet engineering.*

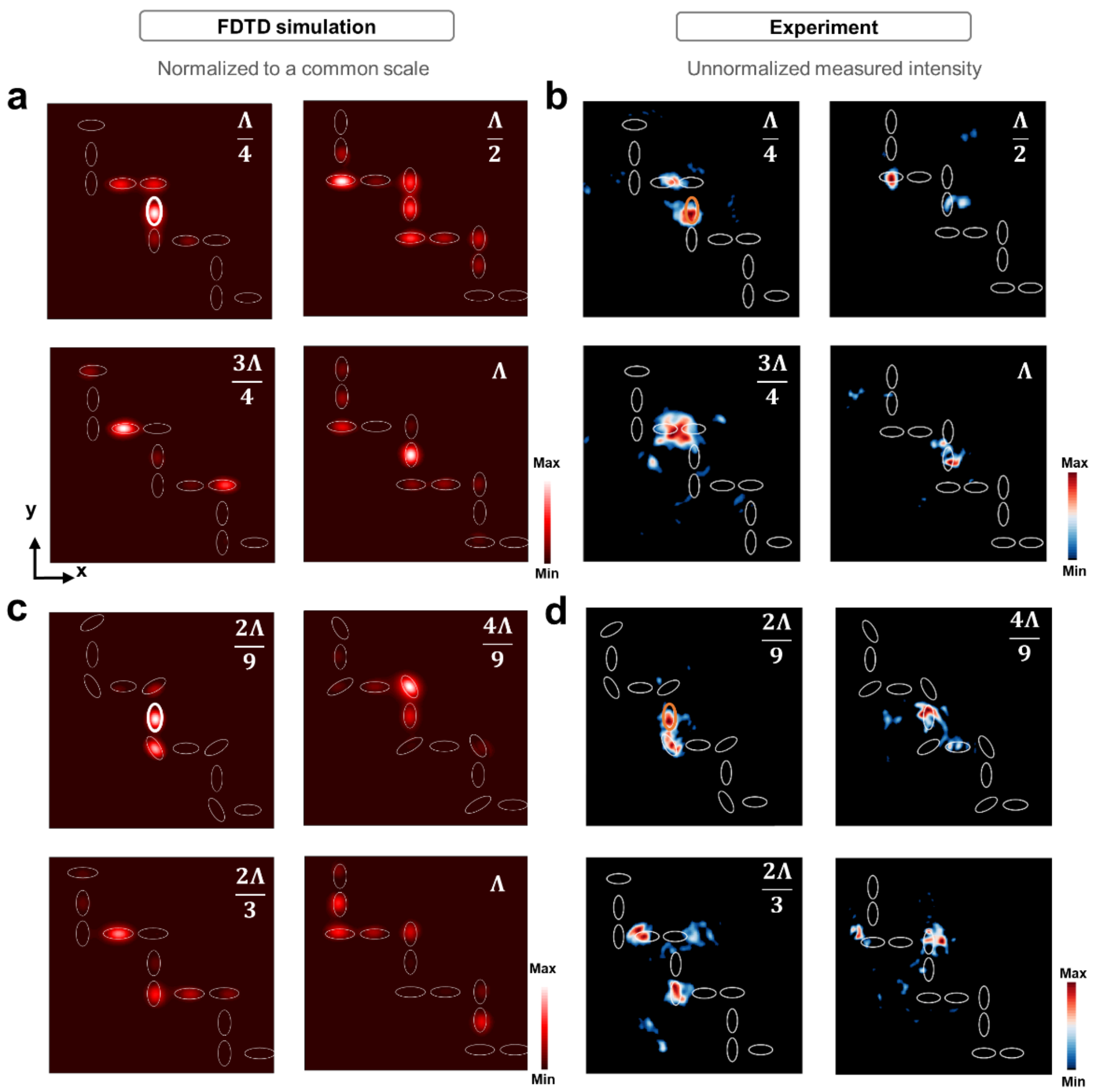

Fig. S26 | Period-Λ and Period-2Λ oscillations: comparison between FDTD simulation and experiment. a, b, *Cross-sectional intensity distributions from FDTD simulation (**a**) and the corresponding experimental measurements (**b**) for the period-Λ oscillation. At z = Λ, the optical field returns to the initially excited waveguide.* ***c, d,*** *Cross-sectional intensity distributions from FDTD simulation (**c**) and the corresponding experimental measurements (**d**) for the period-2Λ oscillation. At z = Λ, the optical field does not return to the initially excited waveguide.*

Comments2:

The overall organization of the manuscript requires substantial improvement. While the paper includes an extensive amount of content, key concepts and results are not always explained clearly. As a result, the central message becomes obscured by the large number of examples presented. We recommend the authors

to restrict themselves to one example for 1D lattices, and one for 2D lattices. This will already prove their point that arbitrary coupling functions are possible. Further implementations can and should in our opinion be published in separate papers showing only the intended effect (e.g. non-Abelian coupling). Restricting the paper to a few examples would help to make space to explain each effect properly, move the important content for these examples from the SI to the main text and make it less fragmented.

Responses:

We thank the Reviewer for this constructive suggestion. We agree that the original manuscript contained too many examples, which weakened the clarity of the central message. In the revised manuscript, we have substantially streamlined the presentation of the demonstrated phenomena in order to clarify the central message of the manuscript.

Specifically, we now focus on the **1D Floquet flat bands together with period-nΛ oscillations and the 2D chirality reversal of topological edge states**.

For the **1D case**, we retain the demonstration of **Floquet flat bands together with period-nΛ oscillations**. The period- nΛ dynamics directly originate from the quasi-energy associated with the engineered flat Floquet bands. More importantly, this 1D phenomenon relies on the central capability of our platform: direct and continuous tuning of the nearest-neighbor coupling coefficient from positive to negative values. In conventional waveguide arrays, realizing flat bands across the entire Brillouin zone solely through nearest-neighbor coupling modulation is highly challenging, especially without nonlinear effects. In our platform, the coupling coefficient is directly determined by the relative polarization angle θ, enabling the required modulation to be designed in a simple and controllable way.

For the **2D case**, we retain the **chirality reversal of chiral-symmetry-protected topological edge states**, which together illustrate how the proposed platform enables tunable topological transport in higher-dimensional lattices. This example is also chosen specifically, because the topological phase transition associated with chirality

reversal requires sign-reversible control of couplings in a 2D lattice, which is challenging to realize in reported works of waveguide arrays. Such control is enabled here by the same distinctive feature of our platform: the direct and continuous tuning of nearest-neighbor couplings from positive to negative values. This allows different nearest-neighbor couplings in the 2D lattice to be independently engineered so that they cross zero at different positions along the propagation direction, thereby fulfilling the condition for reversing the chirality of the edge states.

Following the Reviewer's suggestion, we have **moved the additional 1D topological-edge-state and non-Abelian demonstrations from the main text to the Supplementary Information**, and **transferred theoretical analysis for the retained representative examples from the Supplementary Information into the main text**. In particular, for the 1D period-n Λ oscillation case, we added a more detailed theoretical explanation of the emergence of Floquet flat bands at Λ_{flat} based on the Magnus expansion, as well as a clearer derivation of the condition for period-multiplied oscillations from the Floquet operator. For the 2D chirality-reversal example, we expanded the main-text discussion of the modulation-phase structure of the couplings, its relation to the Floquet graphene model, and the winding-number formulation used to characterize the topological phase transition. In this way, the revised manuscript is not only more focused, but also less fragmented.

We believe these revisions significantly improve the organization of the manuscript and better highlight the central message of the work: that twist-controlled polarization engineering provides a direct and versatile route for designing propagation-dependent coupling functions, including continuous sign reversal, and for realizing representative Floquet phenomena in both 1D and 2D photonic lattices.

Changes in the manuscript:

Abstract:

We have first revised the **Abstract** to reflect the refocused presentation of the revised manuscript. In particular, the revised Abstract now states more explicitly that

the manuscript is centered on the proposed polarization-controlled Floquet engineering for direct and continuous control of both the sign and magnitude of the coupling coefficient, with representative 1D and 2D Floquet phenomena presented as illustrative examples of this capability.

"Floquet engineering has emerged as a powerful approach for both classical and quantum systems, enabling versatile exploration of synthetic dimensions, topological phases, and quantum information processing. Achieving these advances, however, critically relies on the precise and continuous control over the coupling coefficient, including both its sign and magnitude. Yet in existing Floquet photonic platforms, including waveguide arrays, micro-ring resonators, and fiber-loop lattices, such control is commonly achieved through external means such as geometric spacing or phase modulation, rendering continuous and robust sign reversal challenging. Here, we present a compact Floquet platform based on twisted photonic waveguide arrays, in which twist-induced variation of the relative polarization angle between adjacent waveguides enables direct and continuous tuning of the coupling coefficient from positive to negative values. To highlight the core capability of the platform, we focus on representative driven phenomena in both one- and two-dimensional lattices: Floquet flat bands and the associated period-$n\Lambda$ *oscillations in one-dimensional lattices, as well as chiral-symmetry-protected topological edge states and their chirality reversal in two-dimensional lattices. This work paves the way for dynamic photonic engineering and provides a powerful tool potentially applicable to photonic quantum information and photonic computing."*

Introduction:

We have correspondingly revised the **Introduction** to reorganize the main storyline and to present the retained 1D and 2D examples more clearly as representative consequences of the same Floquet engineering capability.

"Here, we propose a twisted photonic waveguide platform for Floquet engineering, in which the relative polarization angle between adjacent waveguides is controlled geometrically by the twist, as illustrated in Fig. 1a. We demonstrate that twist-

controlled modulation of the relative polarization angle between neighboring waveguides enables continuous tuning of the coupling coefficient in both magnitude and sign within a compact propagation platform. Fabricated through high-precision 3D printing, these twisted waveguide arrays with polarization-controlled coupling, provide a versatile platform for exploring diverse physical phenomena. We experimentally showcase the broad utility of this platform through representative driven photonic effects in one- and two-dimensional lattices. Specifically, we first realized Floquet flat bands that extend across the entire Brillouin zone, where the quasi-energy levels can be adjusted by varying the twisting rates of positive and negative coupling segments, giving rise to period-nΛ oscillations and self-imaging in one dimensional (1D) lattices (Fig. 1b). Second, we observed the chirality reversal of the topological edge states in two-dimensional (2D) graphene-like lattices, demonstrating the platform's capabilities for complex high-dimensional Floquet engineering (Fig. 1c). Owing to its geometric nature, the platform is in principle extendable across dimensionalities, materials, and spectral ranges."

Following the Reviewer's suggestion, the 1D topological-edge-state and non-Abelian demonstrations have been moved to the Supplementary Information (**Supplementary Notes 8 and 9; Figs. S23 and S24**), while transferring key explanatory material for the retained representative examples from the Supplementary Information into the main text.

Specifically, in the section "**Floquet Flat Bands and Period-nΛ Oscillations**", we have added a more detailed theoretical explanation of the emergence of flat bands at Λ_{flat} based on the Magnus expansion, together with a clearer derivation of the condition for period-nΛ oscillations from the Floquet operator.

"We next consider the two-site lattice with $\theta_1 = 0$ and $\theta_2 = \frac{\pi}{2}$ (Fig. 4). Flat bands persist for a specific driving period Λ_{flat}, where the twisting rate in the first half of the driving period is faster than that in the second half. In Fig. 4a(i), the coupling coefficient $\kappa(z)$ over one driving period Λ is shown. The integrated areas under the

curve for the first and second halves of the period are denoted as A and B, respectively, and their difference $\Delta = |A - B|$ (dimensionless) determines the quasi-energy $\varepsilon_{flat} = \Delta/\Lambda$ of the flat band, as plotted in Fig. 4a(ii) and (iii). Therefore, the corresponding quasi-energy values at Λ_{flat} are no longer zero, which depend on the rate difference. Fig. 4a(ii), (iii) presents the quasi-energy band under open and periodic boundary conditions (OBCs and PBCs), respectively. The emergence of flat bands at Λ_{flat} can be understood within the framework of the Magnus expansion[41]. The time evolution operator is expressed as $U(z) = e^{-i\Omega(z)}$, where $\Omega(z)$ is expanded as $\Omega(z) = \Omega_1(z) + \Omega_2(z) + \cdots$, with $\Omega_1(z) = \int_0^z H(z_1)dz_1$, $\Omega_2(z) = \frac{i}{2}\int_0^z \int_0^{z_1} [H(z_1), H(z_2)]\, dz_2 dz_1$, and so on. Since $[H(z), H(z')] \neq 0$, the higher-order terms in the Magnus expansion generally contribute nonzero values, resulting in a nonzero quasi-energy spectrum for most driving periods. However, at the specific driving period, denoted as Λ_{flat}, all higher-order terms beyond $\Omega_1(z)$ sum exactly to zero. Consequently, the quasi-energies are determined solely by the first-order term $\Omega_1(z)$, and take on a value of zero across the entire Brillouin zone."

"The intra-cell coupling integrates to zero over one driving period, while the inter-cell coupling yields a finite integrated value, whose absolute value is Δ, and $\epsilon_{flat} = \Delta/\Lambda_{flat}$ gives the flat band quasi-energy across the entire Brillouin zone. By controlling ϵ_{flat} through the twisting rates, period-$n\Lambda$ oscillations ($n \in N^+$) can be realized. Specifically, when the integrated value satisfies $\Delta = \frac{\pi}{n}$, the flat-band quasi-energy becomes $\varepsilon_{flat} = \pm\frac{\pi}{n\Lambda_{flat}}$. In this case, the Floquet operator takes the form $U(\Lambda_{flat}) = P_+ e^{-i\frac{\pi}{n}} + P_- e^{i\frac{\pi}{n}}$, where $P_\pm$ are the projectors onto the $\pm\frac{\pi}{n}$ subspaces. After n driving periods, the state evolves as:

$$|\psi(n\Lambda_{flat})\rangle = (e^{-i\frac{\pi}{n}})^n|\psi_+\rangle + (e^{i\frac{\pi}{n}})^n|\psi_-\rangle = -|\psi_+\rangle - |\psi_-\rangle = -|\psi_0\rangle, \qquad (3)$$

indicating that the optical field distribution reappears, though with a global phase shift. Thus, the recurrence of field intensities requires $\Lambda_o = n\Lambda_{flat}$, while the complete revival of both amplitude and phase requires $\Lambda_o = 2n\Lambda_{flat}$. The relationship between Λ_o and Δ is shown in Fig. 4b."

In the section "**Topological Phase Transitions and Chirality Reversal of Edge States**", we have expanded the theoretical discussion of the phase structure of the coupling modulation in the 2D lattice, its connection to the Floquet graphene model, and the winding-number formulation used to characterize the topological phase transition and chirality reversal. These revisions make the presentation of the retained 1D and 2D examples more complete in the main text and reduce the need for the reader to move back and forth between the main text and the Supplementary Information.

"This phase difference determines the relative magnitude between the static component $cos(\varphi_0)$ *and the amplitude of the fundamental frequency component in the coupling strength. When the waveguides form a graphene lattice, the spatial angle* φ_{space} *of the coupling slits corresponds to the phase of the coupling modulation between neighboring waveguides. Since each waveguide couples to three neighbors in different directions, the modulation phases of the coupling coefficients are* $0, -\frac{2\pi}{3}$ *and* $-\frac{4\pi}{3}$*, respectively. This introduces chiral phase differences in the distribution of coupling strength, effectively breaking time-reversal symmetry. This structure is physically equivalent to a special case of the Floquet graphene model proposed by Takuya Kitagawa*[1]*, in which only the static and fundamental frequency components of the coupling strength are retained. However, in the present scheme, the coupling strength is extended over the entire real domain, offering greater flexibility for tuning and realizing topological phases.*

To verify the topological phase of the system, we calculate its topological invariant—the winding number. For a two-dimensional Class A lattice system without chiral symmetry, the topological classification falls into the integer group $\mathbb{Z}$*, in contrast to one-dimensional Class A systems, which cannot host nontrivial topological phases*[2]*. The winding number can be used to classify mappings from the three-dimensional parameter space* $S^1 \times S^1 \times S^1$ *to the unitary group* $U(N)$ *(i.e., the mapping from the Floquet-Brillouin zone to the space of evolution operators), and is formally defined as follows*[3]*:*

$$G_\pi[U] = \frac{1}{8\pi^2}\int dt dk_x dk_y \times Tr\left(U^{-1}\partial_t U\left[U^{-1}\partial_{k_x}U, U^{-1}\partial_{k_y}U\right]\right), \quad (5)$$

where $U(k,z)$ *is the evolution operator of the periodically driven system over one period, and* N *denotes the number of mode degrees of freedom within the unit cell, which corresponds to the dimension of the unitary group* $U(N)$ *to which the evolution operator* $U(k,z)$ *belongs. The above definition requires that* $U(k,t)$ *be periodic both in time* t *and in momentum space* (k_x, k_y)*. However, this condition is not automatically satisfied in general. Therefore, it is necessary to construct a smooth evolution operator* $U_\varepsilon(k,z)$ *that is periodic in the three-dimensional parameter space, defined as follows:*

$$U_\varepsilon(k,z) = \begin{cases} \mathrm{U}(k,2z) & if\ 0 \le z \le \Lambda/2 \\ V_\varepsilon(k,2\Lambda - 2z) & if\ \Lambda/2 \le z \le \Lambda' \end{cases} \quad (6)$$

where $V_\varepsilon(k,t) = e^{-iH_F(k)z}$*. By appropriately choosing the branch cut of the complex logarithm function, the winding number calculation can be focused on a specific quasi-energy band* ε*:*

$$\begin{aligned} loge^{-i\varepsilon\Lambda + i0^-} &= -i\cdot\varepsilon\Lambda, \\ loge^{-i\varepsilon\Lambda + i0^+} &= -i\cdot\varepsilon\Lambda - 2\pi i. \end{aligned} \quad (7)$$

In particular, we focus on the anomalous energy gap located at $\varepsilon = \frac{\pi}{\Lambda}$*.The corresponding winding number is shown in Fig. 5a(iii), where* δ*, the reciprocal of the static component* $\frac{\kappa_0}{2}\cos\theta_0$*, is taken as the energy scale. As the driving period increases, the topological invariant undergoes a sequence of transitions—from 0 to 1, and subsequently from 1 to –1. Correspondingly, the edge-state propagation reverses its direction from counterclockwise (CCW) to clockwise (CW) before and after the second phase transition.*"

Figures:

We have revised **Fig. 1** in the main text to reflect the refocused presentation of the revised manuscript. Specifically, the original panels b–d have been replaced by two panels focused on the two representative examples retained in the main text.

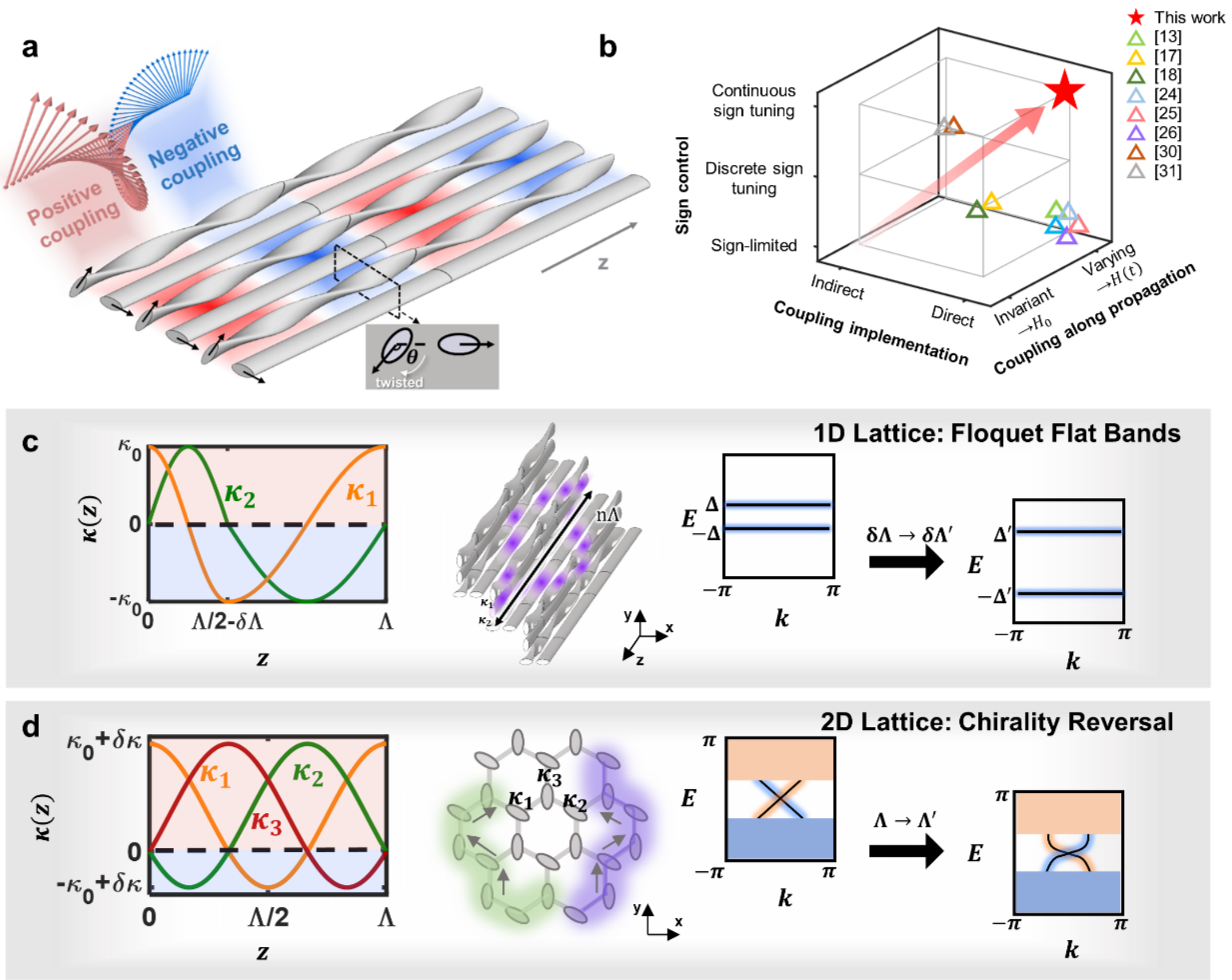

Fig. 1 | Polarization-controlled Floquet engineering enabled by twisted photonic lattices. a, *Concept of polarization-controlled coupling in twisted waveguide arrays. As the waveguides twist along* z*, the relative polarization angle* θ *between adjacent waveguides evolves continuously, allowing the nearest-neighbour coupling coefficient* $\kappa(z)$ *to be tuned from positive to negative values. Red and blue denote positive and negative coupling, respectively.* ***b,*** *Compared with previous Floquet photonics platforms, our platform is the first to enable the nearest-neighbour coupling coefficient to vary directly and continuously in both magnitude and sign along the propagation direction.* ***c,*** *1D lattice for Floquet flat-band engineering. Left: alternating couplings* κ_1 *and* κ_2*. Middle: twisted waveguide array. Right: quasi-energy spectrum exhibits Floquet flat bands at quasi-energies* $\pm\Delta$*,* $\pm\Delta'$*, ..., where different flat-band quasi-energies correspond to period-*$n\Lambda$ *oscillations with different integer* n*.* ***d,*** *2D lattice for chirality reversal of topological edge states. Left: three couplings,* κ_1*,* κ_2*, and* κ_3*. Middle: twisted waveguide array. Right: the quasi-energy spectrum supports edge states, whose chirality is reversed when the driving period is changed from* Λ *to* Λ'*.*

References:

44. Kitagawa, T., Berg, E., Rudner, M. & Demler, E. Topological characterization of periodically driven quantum systems. Phys. Rev. B 82, 235114 (2010).

45. Fruchart, M. Complex classes of periodically driven topological lattice systems. Phys. Rev. B 93, 115429 (2016).

46. Rudner, M. S., Lindner, N. H., Berg, E. & Levin, M. Anomalous Edge States and the Bulk-Edge Correspondence for Periodically Driven Two-Dimensional Systems. Phys. Rev. X 3, 031005 (2013).

Supplementary materials:

*"**Supplementary Note 8 | Topological phase transition in the 1D lattice.***

To satisfy the chiral symmetry condition, the evolution operator $U(k,\Lambda)$ must obey $\Gamma U(k,\Lambda)\Gamma^{-1}=U^{-1}(k,\Lambda)$[6]. This requirement implies a symmetric inversion point along propagation, $H(k,z)=H(k,-z)$ (Supplementary Note 6), together with the local chiral symmetry relation $\Gamma H(k,z)\Gamma^{-1}=H^{-1}(k,z)$. Setting $\theta_1=\frac{\pi}{2}$ and $\theta_2=0$ (Fig. S23a(i)), the waveguides twist by $\frac{\pi}{2}$ and then reverse direction. This configuration (Fig. S23a(ii)) yields coupling coefficients $\kappa_1(z)=\kappa_0|sin\,(\theta(z))|$ and $\kappa_2(z)=\kappa_0|cos\,(\theta(z))|$ (more situations are discussed in Supplementary Note 6). Fig. S23a(iii) shows the quasi-energy spectrum under OBCs with 80 waveguides as a function of the driving period Λ, where anomalous Floquet π modes appear within the range $\Lambda>2.5$. The topological invariant G_π[4] can be defined for the quasi-energy gap at π as:

$$G_\pi=\frac{i}{2\pi}\int_{-\pi}^{\pi}tr((T_\pi^+)^{-1}\partial_k T_\pi^+)dk\,, \tag{21}$$

where T_π^+ is the diagonal matrix element of $T(\frac{\Lambda}{2},k)$, and $T(z,k)\equiv U(z,k)e^{iH_F(k)z}$. The numerical result of G_π as a function of Λ for the two-site lattice is presented in Fig. S23a(iv). As Λ increases, G_π changes from 0 to -1, and then to -2, corresponding to 0, 2, and 4 edge states. This realization of higher-winding ($|G_\pi|>1$) phases in the topological waveguide array enabled by polarization-controlled Floquet engineering stands in contrast to previous schemes, which typically required next-nearest-neighbor interactions[7] or asymmetric couplings[8].

Theoretical evolutions of the bulk states and edge states are shown separately in Fig. S23b(i) and Fig. S23b(iii), where the Floquet driving period is Λ=4cm for both cases. The topological edge state still propagates along the edge with periodic-driven oscillation and a negligible amount of dissipation into the bulk, whereas the bulk state rapidly delocalizes throughout the array. Similarly, in order to show the scalability of the proposed polarization-controlled strategy, additional experiments on hollow metallic waveguides are demonstrated in Supplementary Fig. S21."

*"**Supplementary Note 9 |** Non-Abelian Transformations Based on Polarization-Controlled Coupling.*

Beyond the realization of Floquet-driven phenomena, our platform further enables the implementation of non-Abelian transformations. In previous schemes[4,5], only closed adiabatic trajectories on the Bloch sphere were realized, restricting the accessible operations to pure rotations belonging to the special orthogonal group SO(2), and the realization of non-Abelian transformations requires a degenerate subspace of more than two zero modes. In contrast, the polarization-controlled platform developed here supports both closed and non-closed adiabatic evolution trajectories of zero modes in the Bloch-sphere parameter space. Consequently, the accessible operation group is extended from SO(2) to O(2), thereby expanding the family of achievable matrix operations beyond those defined by closed loops. This capability enables our system to realize a broader class of non-Abelian transformations that are inaccessible in previous platforms, and implement them within a subspace spanned as few as two zero modes.

This capability allows straightforward realization of Pauli-X, Y and Z gates. For the logical Z gate, $Z = \begin{bmatrix} 1 & 0 \\ 0 & -1 \end{bmatrix}$*, the structure consists of one straight waveguide A and one twisted waveguide B by π (Fig. S24a(i)). The large spacing between the two waveguides suppresses coupling. As the polarization direction of TE mode in B rotates from +x to -x, a geometric phase of π is accumulated, effectively introduces a -1 factor the logical state, as illustrated in Fig. S24a(ii). The experimental output fields and phases at the end facet are shown in Fig. S24a(iii). After twisting waveguide B, the electric field remains localized in waveguides A and B, while the relative phase between*

the two outputs is locked at π. The phase shown here corresponds to the total accumulated phase (dynamical + geometric). Only the relative phase difference is physically meaningful. This phase shift originates from a geometric rather than a dynamical phase, as confirmed by its invariance when the input wavelength is tuned varied from 1160 μm to 1420 μm.

We next implement the logical X gate, $X = \begin{bmatrix} 0 & 1 \\ 1 & 0 \end{bmatrix}$, using a four-waveguide structure composed of three twisted waveguides (A, B, and C) and one straight waveguide (X) (Fig. S24b(i)). The coupling coefficients are given by $\kappa_{iX} = \kappa_0 cos\,(\theta_{iX}(z))$ (i=A, B, C). The logical X gate is achieved through three sequential twisting operations, each involving a pair of twisted waveguides rotated by $\frac{\pi}{2}$ in the same direction simultaneously. Light is initially injected equally into waveguides A and B. In the first step, twisting A and C yields $\theta_{CX}(z) = \theta(z)$ and $\theta_{AX}(z) = \theta(z) + \frac{\pi}{2}$, corresponding to coupling coefficients $\kappa_0 cos\theta(z)$ and $-\kappa_0 sin\theta(z)$, respectively. As θ evolves from 0 to $\frac{\pi}{2}$, the zero eigenmodes $\psi_1 = (cos\theta(z), 0, sin\theta(z), 0)^T$ and $\psi_2 = (0, 1, 0, 0)^T$ transform continuously, transferring the optical field from A into C, as illustrated in Fig. S24b(ii). The subsequent two steps proceed similarly: after all three operations, one zero mode evolves from $A \to C \to B$, and the other from $B \to A$, both acquiring a total geometric phase of π. This process realizes a unitary operation equivalent to the logical X gate, corresponding to a non-closed adiabatic trajectory on the Bloch sphere.

The logical Y gate, $Y = \begin{bmatrix} 0 & -i \\ i & 0 \end{bmatrix}$, can be equivalently realized by $\begin{bmatrix} 0 & -1 \\ 1 & 0 \end{bmatrix}$. Steps 1 and 2 are identical to those of the X gate, while in step 3, waveguide C is twisted $\frac{\pi}{2}$ in the opposite direction. After the three steps, the two zero modes acquire a relative phase difference of π. The resulting operation corresponds to the logical Y gate, corresponding to a closed adiabatic trajectory on the Bloch sphere. The detailed derivations for all three gates are provided in Supplementary Note 18.

These operations can be combined to perform more complex non-Abelian transformations within the subspace spanned by two or more zero modes. This

approach provides a practical pathway toward applying such transformations in quantum information processing and photonic quantum computing."

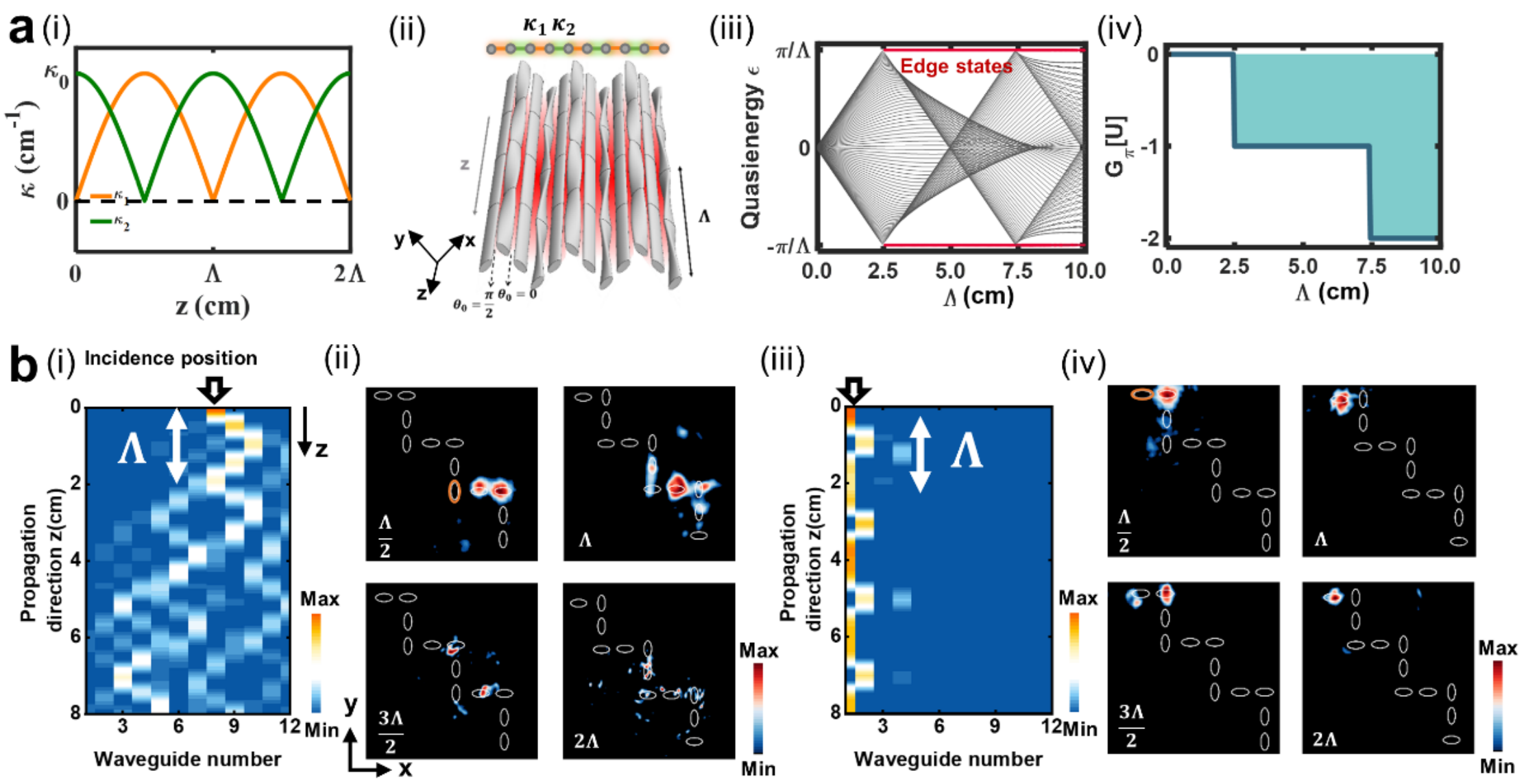

Fig. S23 | Topological phenomena in a one-demension lattice. a, *Topological properties in one-dimensional lattice.* ***(i)*** *Coupling coefficient* κ_1 *and* κ_2 *as a function of propagation distance* z*.* ***(ii)*** *Structure of the fabricated waveguide array consisting of twelve waveguides with 1000 μm spacing and a propagation distance of* $L = 4$ *cm, corresponding to two Floquet driving period* Λ*.* ***(iii)*** *The corresponding quasi-energy spectrum under open boundary conditions, calculated for an array of eighty waveguides, exhibits edge states at* $\Lambda > 2.5$*.* ***(iv)*** *Topological invariant calculation confirms topological phase transition as the driving period increases.* ***b,*** *Experimental demonstration of edge states in one-dimensional systems.* ***(i)*** *Numerical theory of light evolution shows spreading into the bulk.* ***(ii)*** *Experimental results at* $\frac{\Lambda}{2}$*,* Λ*,* $\frac{3\Lambda}{2}$*, and* 2Λ *showing bulk transport and no topological localization. Orange dashed circles highlight the injected waveguides.* ***(iii)*** *Numerical theory of light evolution shows robust edge localization.* ***(iv)*** *Experimental results reveal strongly localized oscillations near the edge, verifying the presence of Floquet edge states protected by chrial symmetry.*

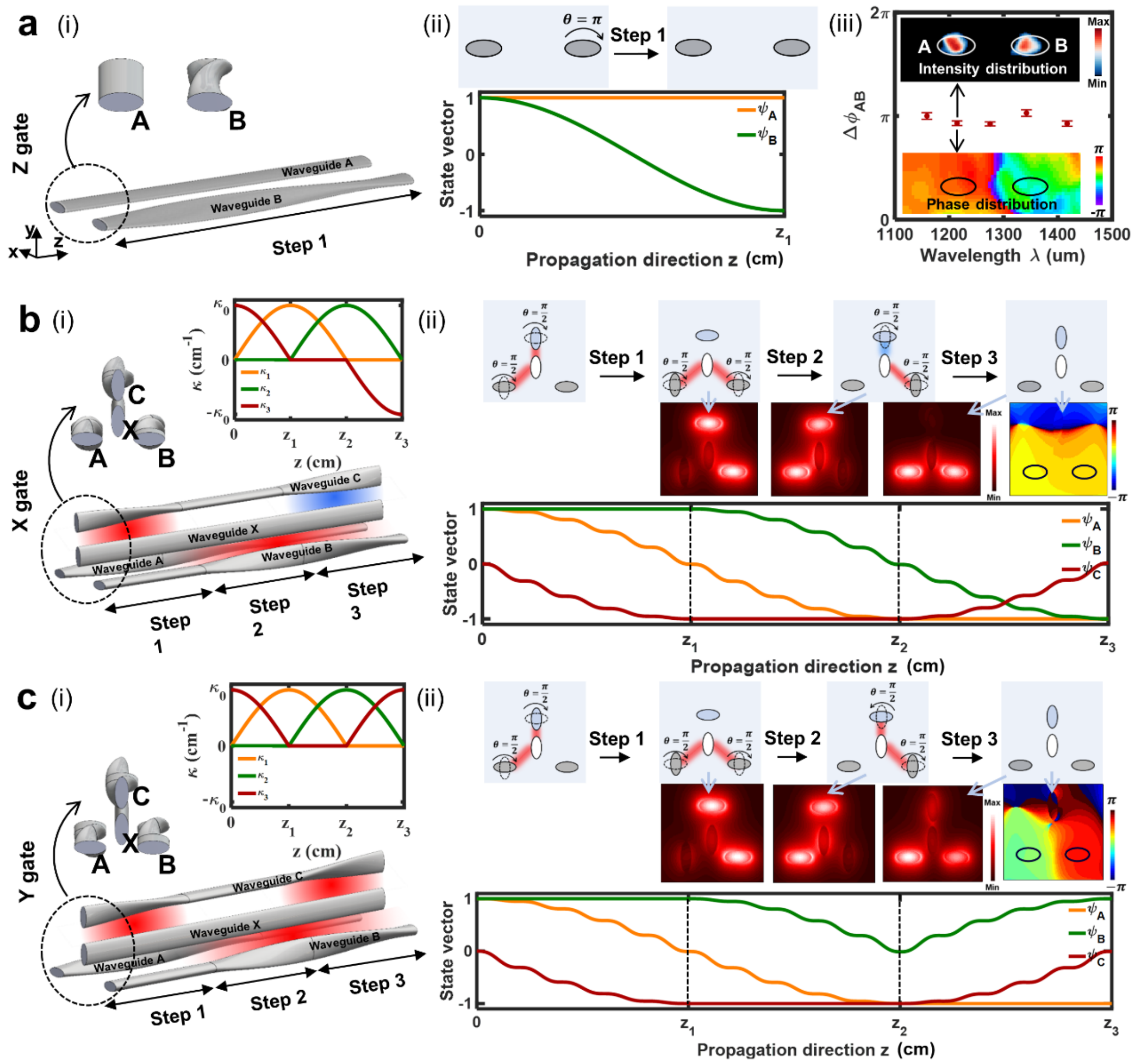

***Fig. S24 | Realization of logical non-Abelian gates based on polarization-controlled coupling. a**, Implementation of the logical Z gate. **(i)** Structure consisting of a straight waveguide A and a twisted waveguide B. The fabricated sample has an inter-waveguide spacing of 2000 μm and a length of 1.5 cm. **(ii)** Theoretical evolution of state vectors showing that twisting waveguide B introduces a relative phase of π between the two outputs. **(iii)** Experimental measurements of optical intensity (top) and phase (bottom) distributions confirm that the phase shift remains fixed at π across a broad wavelength range, demonstrating its geometric origin. **b**, Implementation of the logical X gate. **(i)** Four-waveguide structure composed of three twisted waveguides (A, B, C) and one straight waveguide (X). The illustration shows the coupling coefficient κ_1, κ_2 and κ_3 as a function of propagation distance z. **(ii)** Simulated and theoretical results show that the two zero modes acquire identical phase, yielding the X gate transformation. **c**, Implementation of the logical Y gate. **(i)** Similar structure as in **b**, but in the third step waveguide C is twisted in the opposite direction. The*

illustration shows the coupling coefficient κ_1*,* κ_2 *and* κ_3 *as a function of propagation distance z.* ***(ii)*** *Simulated and theoretical results show that the two zero modes acquire a relative* π *phase, yielding the Y gate transformation.*

Reply to Reviewer 2:

General Comment:

In the present work the authors consider an array of coupled waveguides which can be oriented in space and their orientation modulated in a desirable matter to control coupling and its phase. As such, the idea of using polarization degree of freedom is not new (refs [1,2] to name just a few, but many early models consider polarized modes). Moreover, it has been used before for controlling coupling of phase in publication Ref. [3]. The authors then follow with several experimental results (although limited to 1D and of experiments of rather poor quality (e.g., judging from experimental figures in Fig. 3). Conceptually, the paper offers nothing new, and the authors go all over the place to demonstrate the power of the proposed approach. Unfortunately, I do not see any of their results as groundbreaking and sufficiently novel. I would rather see one strong result instead of many mediocre ones, which are also based on the approach that is not truly original. To summarize, the paper lacks originality and does not present any solid/scientifically sound progress that would deserve dissemination in Nature Communications.

[1] Zhang, Y., Wu R. P. H., Shi, L., and Fung, K. H., Second-Order Topological Photonic Modes in Dipolar Arrays, ACS Photonics 7(8) 2002, (2020).

[2] Schulz, J., Noh, J., Benalcazar, W.A. et al. Photonic quadrupole topological insulator using orbital-induced synthetic flux. Nat Commun 13, 6597 (2022).

[3] Bobylev, D. A., Tikhonenko, D. I., Zhirihin, D. V., et al., Topological Edge and Corner States Designed via Meta-Atoms Orientation. Laser Photonics Rev 17, 2100567 (2022).

Responses:

We thank the Reviewer for the detailed comments regarding the originality, overall

focus, and experimental presentation of our manuscript. However, we respectfully believe that the Reviewer's assessment may not fully reflect the central novelty of our work. The references raised by Reviewer 2 are implemented in different physical systems, and their polarization- or orientation-based control relies on physical mechanisms distinct from ours. Even in the work described by Reviewer 2 as "controlling coupling of phase," the coupling is indirectly tuned through hybrid electric–magnetic responses in a static bianisotropic lattice. In contrast, our platform directly maps the linear polarization of the guided fundamental mode to the modal overlap between adjacent waveguides, thereby enabling direct control of the amplitude and sign of the nearest-neighbor coupling coefficient. More importantly, these references are not directly relevant to the core originality of our work: polarization-controlled **Floquet engineering** through direct and continuous coupling control.

Regarding the three references cited by the reviewer, namely **Zhang et al., Schulz et al., and Bobylev et al.** (Refs. **1-3** in the response), we have carefully re-examined them and clarified their distinction from our work.

Zhang et al.[1] uses tensorial dipole-dipole interactions in a dipolar breathing Kagome array and mainly demonstrates higher-order topological states, such as corner states. **It does not provide Floquet modulation or direct tunable control of the coupling coefficient itself.**

Schulz et al.[2] realizes an effective negative coupling through mixed s/p orbitals in a specially engineered four-site unit cell, leading to topological corner states. The π phase is accumulated when the wavefunction traverses the p orbital, so the sign reversal is encoded as a plaquette-scale synthetic π flux rather than obtained by direct control of the local coupling coefficient itself. **It therefore does not provide direct and continuous tuning of the coupling coefficient in sign and magnitude during propagation for Floquet engineering.**

Bobylev et al.[3] relies on orientation-dependent magnetoelectric hybridization in bianisotropic split-ring resonators and mainly demonstrates topological edge and corner

states. **It does not realize Floquet modulation or direct continuous control of the local coupling coefficient.**

To further clarify this distinction, after discussing these three references we further expanded the comparison to include several additional representative photonic works[4–6] using the polarization degree of freedom, so that the discussion is not restricted only to the three papers cited by the reviewer. This broader comparison is summarized in Table R1. As shown there, previous polarization- or orientation-related studies do not realize the central capability achieved here: direct and continuous sign-reversible coupling control in a photonic Floquet platform based on a twisted waveguide array.

Table R1 | Comparison between this work, the studies cited by Reviewer 2, and additional representative polarization- or orientation-related platforms. Comparison of the present work with representative prior studies employing polarization -related mechanisms. The table highlights the key distinctions of this work: a propagation-based twisted-waveguide Floquet platform, direct and continuous control of the nearest-neighbor coupling coefficient, and the resulting access to nontrivial Floquet phenomena in both 1D and 2D lattices.

Platform	Reference	Controlled degree of freedom	Coupling control mechanism	Key distinction from our work			Physical phenomena
				Direct polarization to coupling mapping	Floquet engineering	Physical subfield	
Twisted waveguide array	Our work	Relative polarization angle	Twist controlled modal overlap variation between adjacent waveguides	√	√	Floquet dynamics; topological Floquet physics	Floquet flat bands; period-$n\Lambda$ oscillations; chirality reversal of topological edge states
Dipolar coupling systems	[1], [4]	Dipole orientation and vectorial field components	Tensorial dipole-dipole interaction; vectorial near field coupling	×	×	Topological physics; vectorial near field spectroscopy	Higher order corner and hinge modes; vectorial near field resonance shifts
Mixed s/p orbital photonic lattices	[2]	Orbital parity of s and p modes	P orbital parity induced synthetic π flux	×	×	Topological physics	Quadrupole topological corner states
Bianisotropic meta atom lattices	[3]	Relative orientation of split ring resonators	Magneto electric hybridization	×	×	Topological physics	Topological edge and corner states
OAM optical lattices	[5], [6]	Orbital angular momentum states	OAM induced tunnelling phases and effective flux	×	×	Topological physics	Aharonov Bohm caging; topological edge and corner states

In the following, we provide a more detailed response from the perspectives of **I. Originality, II. Manuscript Organization,** and **III. Experimental Results:**

I. Originality:

Here, we would like to restate the originality of our work. The novelty does not lie in the generic use of polarization as an additional degree of freedom, but in the following specific advances:

(i) A polarization-controlled Floquet photonic platform.

We emphasize that the central contribution of this work is not the mere use of polarization, but the **introduction of a new coupling-control mechanism for Floquet photonic engineering**.

To clarify this point, we have added Fig. R11, which places our work in the context of representative Floquet photonic platforms and highlights, to the best of our knowledge, the first use of polarization-controlled coupling in a Floquet photonic system to achieve direct and continuous control over both the sign and magnitude of the coupling coefficient. This comparison shows that earlier platforms typically fall into one of several categories: fixed coupling[7,8], sign-limited coupling[9–12], or indirect real-valued coupling implemented through auxiliaries[13] or additional modulation[14]. By contrast, our platform combines direct coupling control, continuous sign tunability, and Floquet versatility in a single Floquet platform. For completeness, we also compiled a more detailed comparison across representative Floquet photonic systems, now presented as Table R2.

a

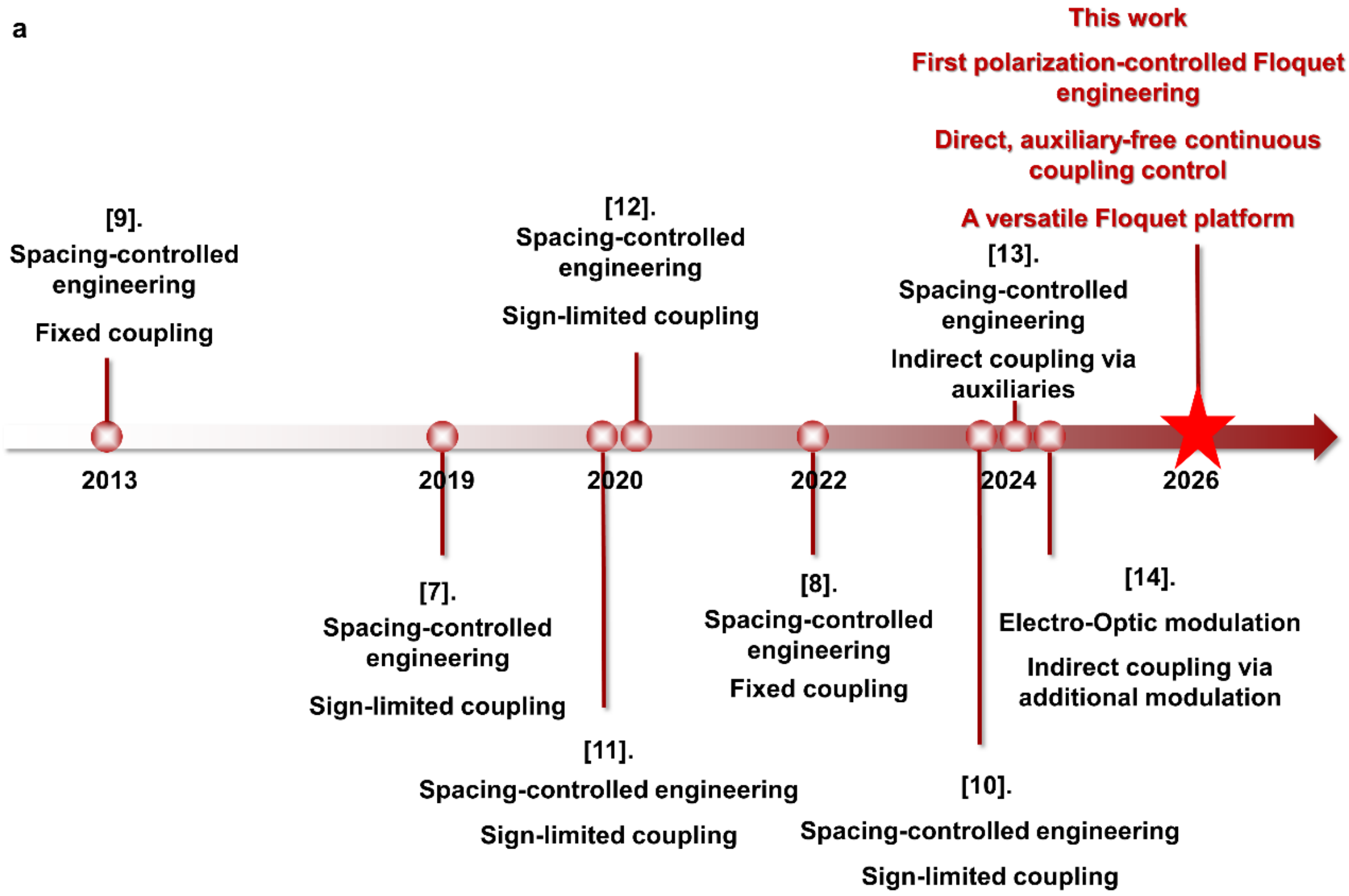

b

	Sign control	Direct coupling implementation	Coupling modulation along propagation
This work	Continuous sign tuning	√	√
[8], [9]	Discrete sign tuning	√	×
[7], [10], [11], [12]	Sign-limited	√	√
[13], [14]	Discrete sign tuning	×	√

c

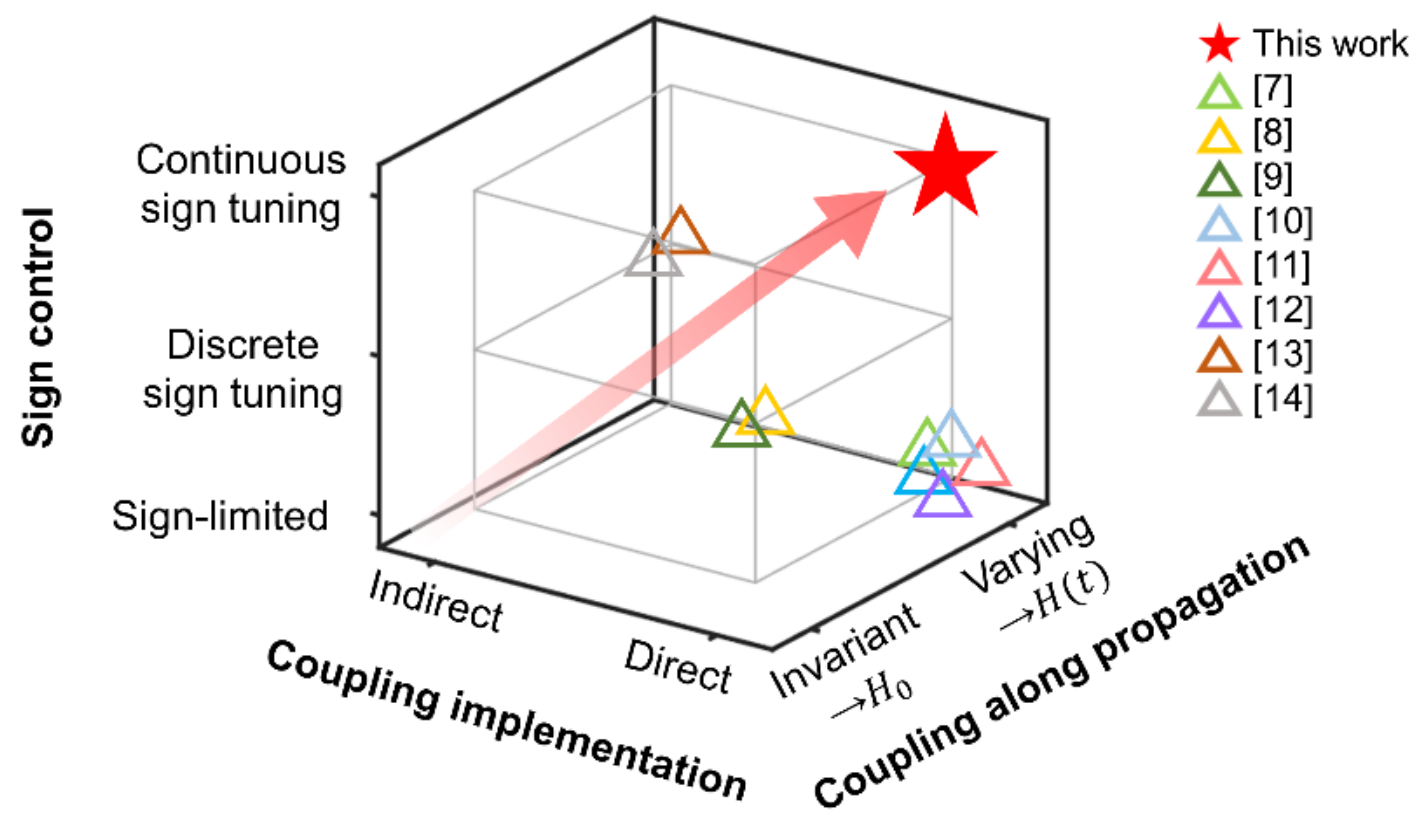

Fig. R11 | Positioning of this work among Floquet photonic platforms. a, Timeline of representative platforms in photonic Floquet engineering. **b**, Comparison of key features: continuous sign-and-magnitude control, direct coupling without auxiliaries or phase programming, and platform versatility. **c**, Conceptual comparison highlighting the distinct position of this work.

Table R2 | Comparison of the present work with representative Floquet photonic platforms in waveguide arrays, micro-ring resonators, and fiber-loop lattices.

Floquet photonic platform	Reference	Coupling control				Physical phenomena
		Direct coupling	Controlled physical degree of freedom	$\kappa(z)$ variation within one Floquet period	Continuous sign tuning of $\kappa(z)$	
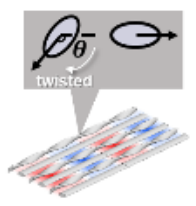 Twisted waveguide array	Our work	√	Relative polarization angle	√	√	Floquet flat bands and period-$n\Lambda$ oscillations in 1D lattices; chirality reversal of topological states in 2D lattices
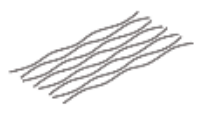 Curved waveguide array	[7]	√	Waveguide spacing	√	×	Anomalous π-edge modes
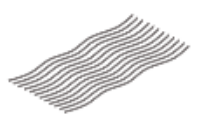 Curved waveguide array	[8]	√	Waveguide bending trajectory	×	×	Artificial gauge field-enabled dispersionless coupling
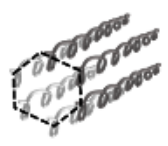 Helical waveguide array	[9]	√	Waveguide helical trajectory	×	×	Floquet topological one-way edge states
	[10]	√	Waveguide spacing	√	×	Parity-time-symmetric photonic topological insulator
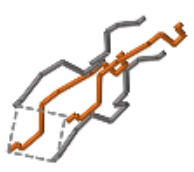 Waveguide array	[11]	√	Waveguide spacing	√	×	Bulk/edge solitons
	[12]	√	Waveguide spacing	√	×	Topological Floquet solitons
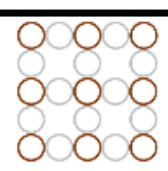 Microring resonators	[13]	×	Resonator spacing, Propagation constant detuning	√	×	Full k-space flat bands
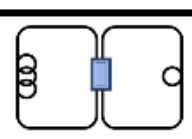 Fiber-loop lattice	[14]	×	Electro-Optic modulation	√	×	Non-Abelian lattice gauge fields

We also note that the other reviewers assess the work more positively on these points. **Reviewer 1** states that, *to their knowledge, this study is* ***the first to*** *use rotation protocols of waveguide cross-sections to implement arbitrary coupling functions, and further notes that extension of this idea to optical-frequency waveguide arrays would move waveguide-based quantum simulation forward.* **Reviewer 3** likewise *recognizes twisted waveguide arrays as* ***a more streamlined platform*** *for various control mechanisms and comments that, for the most part, the methodology is described in sufficient detail to reproduce the results.*

(ii) Direct and continuous control of the nearest-neighbor coupling coefficient in both sign and magnitude.

We realize direct control of the local nearest-neighbor coupling coefficient itself, without additional structures or modulations. During the modulation, the magnitude of the coupling coefficient oscillates between zero and the maximum value and its sign alternates between positive and negative.

(iii) Access to new physics in both 1D and 2D lattices.

This Floquet platform reveals new Floquet physics enabled by such direct coupling control. In particular, we demonstrate flat bands spanning the full Brillouin zone and period-$n\Lambda$ oscillations in 1D lattices and chirality reversal of topological edge states in 2D lattices These phenomena are difficult to realize in previous Floquet photonic platforms because direct control of both the magnitude and sign of the coupling coefficient through an experimentally accessible physical degree of freedom, such as the relative polarization angle, has remained challenging.

Taken together, Table R1, Fig. R11, and Table R2 support our central point: the present work is not a routine reuse of polarization, but the establishment of **a distinct Floquet photonic platform based on direct, continuous, and auxiliary-free coupling engineering in twisted waveguide arrays, together with representative demonstrations of nontrivial driven physics in both 1D and 2D lattices.**

We have revised the manuscript to make this distinction more precise and we state explicitly that the advance of the present work lies in the combination of: **(i)** a twisted waveguide Floquet platform, **(ii)** direct and continuous control of coupling sign and magnitude, and **(iii)** direct applicability to Floquet engineering in driven 1D and 2D lattices.

Therefore, we respectfully disagree with the assessment that the present work "offers nothing new".

II. Manuscript organization:

In response to the Reviewer’s suggestion, we have substantially reorganized the manuscript so that the central storyline is now built around the core capability of the platform and its representative 1D and 2D Floquet phenomena. Specifically, we now focus on one representative example in one dimension and one representative example in two dimensions.

For the 1D case, we retain Floquet flat bands together with period-$n\Lambda$ oscillations, because the latter arise directly from the flat-band quasi-energy and therefore serve as a dynamical consequence of the same underlying mechanism rather than as an additional independent example. More importantly, this 1D phenomenon relies precisely on the key capability of our platform, namely the direct and continuous tuning of the nearest-neighbor coupling from positive to negative values. Because the coupling is directly related to the polarization angle θ through a simple and intuitive functional relation, the modulation profile can be designed in a straightforward and controllable way, making the realization of integrated flat-band oscillations possible in a direct manner. By contrast, in previous photonic Floquet platforms, the coupling is typically either sign-limited or controlled indirectly through auxiliaries or additional modulation, and therefore does not provide an intuitive route for directly designing and implementing integrated flat-band oscillations.

For the 2D case, we retain the chirality reversal of topological edge states, because it provides a representative higher-dimensional consequence of the same key capability, namely direct and continuous sign-reversible control of the nearest-neighbour coupling coefficient. This allows different nearest-neighbor couplings in the 2D lattice to be independently engineered so that they cross zero at different positions along the propagation direction, thereby fulfilling the condition for reversing the chirality of the edge states.

In this way, the revised manuscript emphasizes more clearly that the proposed twisted-waveguide platform is the central advance, while the 1D and 2D results are presented as representative consequences of this Floquet engineering capability. This refocused presentation is also reflected in the revised Abstract, Introduction, and Fig. 1.

III. Experimental results:

Regarding the Reviewer's concern about the experimental quality, we added the new measurements using a terahertz vector network analyzer (THz-VNA) system to measure the output intensities of our samples. In this system, the terahertz signal is electrically generated by the frequency extension modules driven by the vector network analyzer, providing a stronger incident terahertz field than in the previous THz-TDS system. The transmitted signal is also detected by the VNA through coherent measurement, which gives the system a higher signal-to-noise ratio than the THz-TDS system used in the previous version. Importantly, the new THz-VNA measurements are in good agreement with the full-wave finite-difference time-domain (FDTD) simulations, and are also consistent with the previous experimental results obtained using the terahertz time-domain spectroscopy (THz-TDS) system. This consistency confirms that the original THz-TDS measurements are reliable, while the new THz-VNA measurements provide clearer and more compelling experimental evidence.

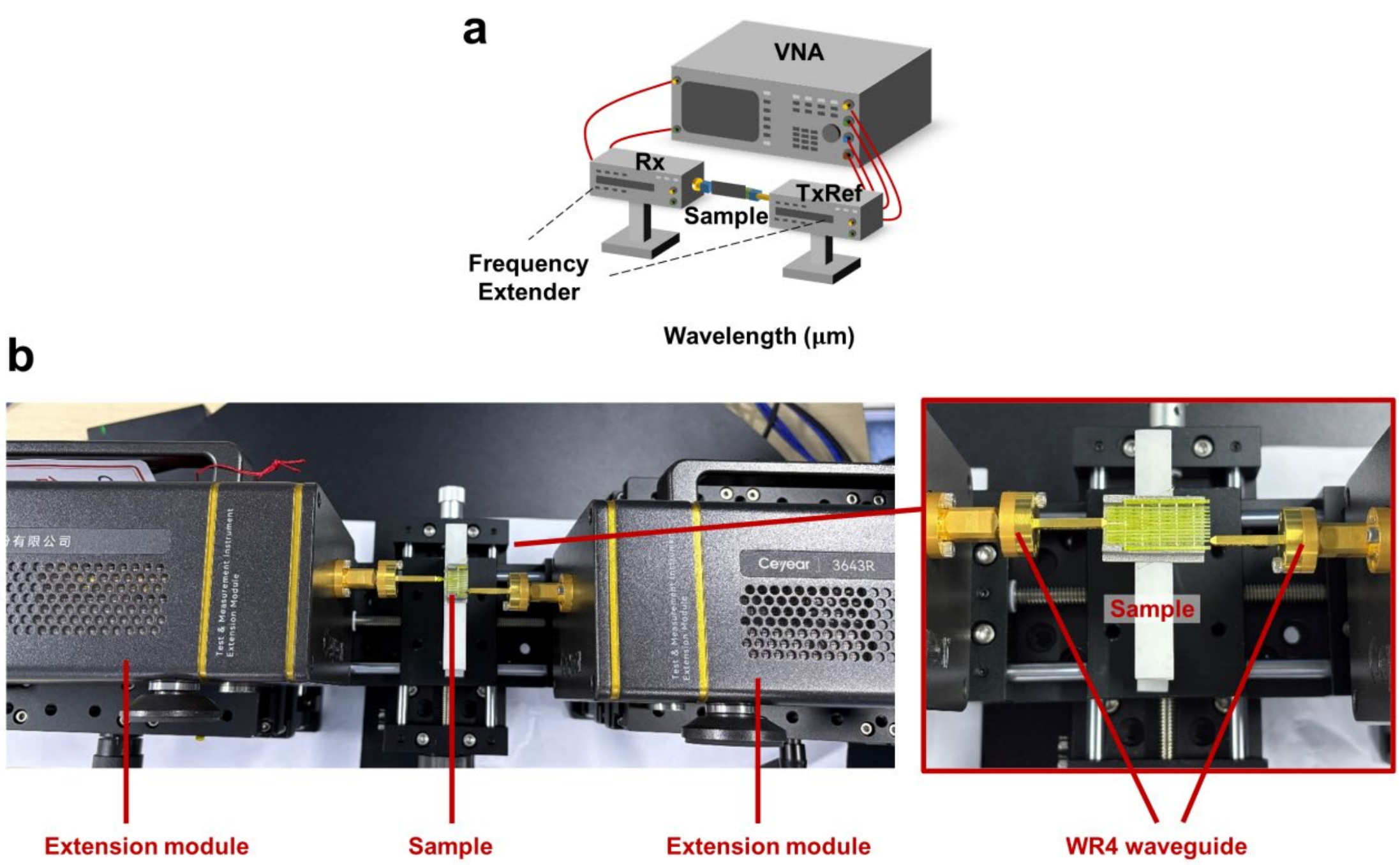

Fig. R5 | THz-VNA measurement setup for the repeated experiments. a, Schematic of the THz-VNA measurement system, consisting of a VNA, two frequency extension modules, and the sample placed between them. The TxRef module serves as the transmitting and reference port for terahertz signal generation and coherent detection, whereas the Rx module receives the transmitted terahertz

signal. **b,** Photograph of the corresponding experimental setup. The sample is mounted between the transmitting and receiving extension modules, and the inset shows an enlarged view of the sample region. Two WR4 metallic waveguides are connected to the sample to couple the terahertz signal into the structure and collect the transmitted output.

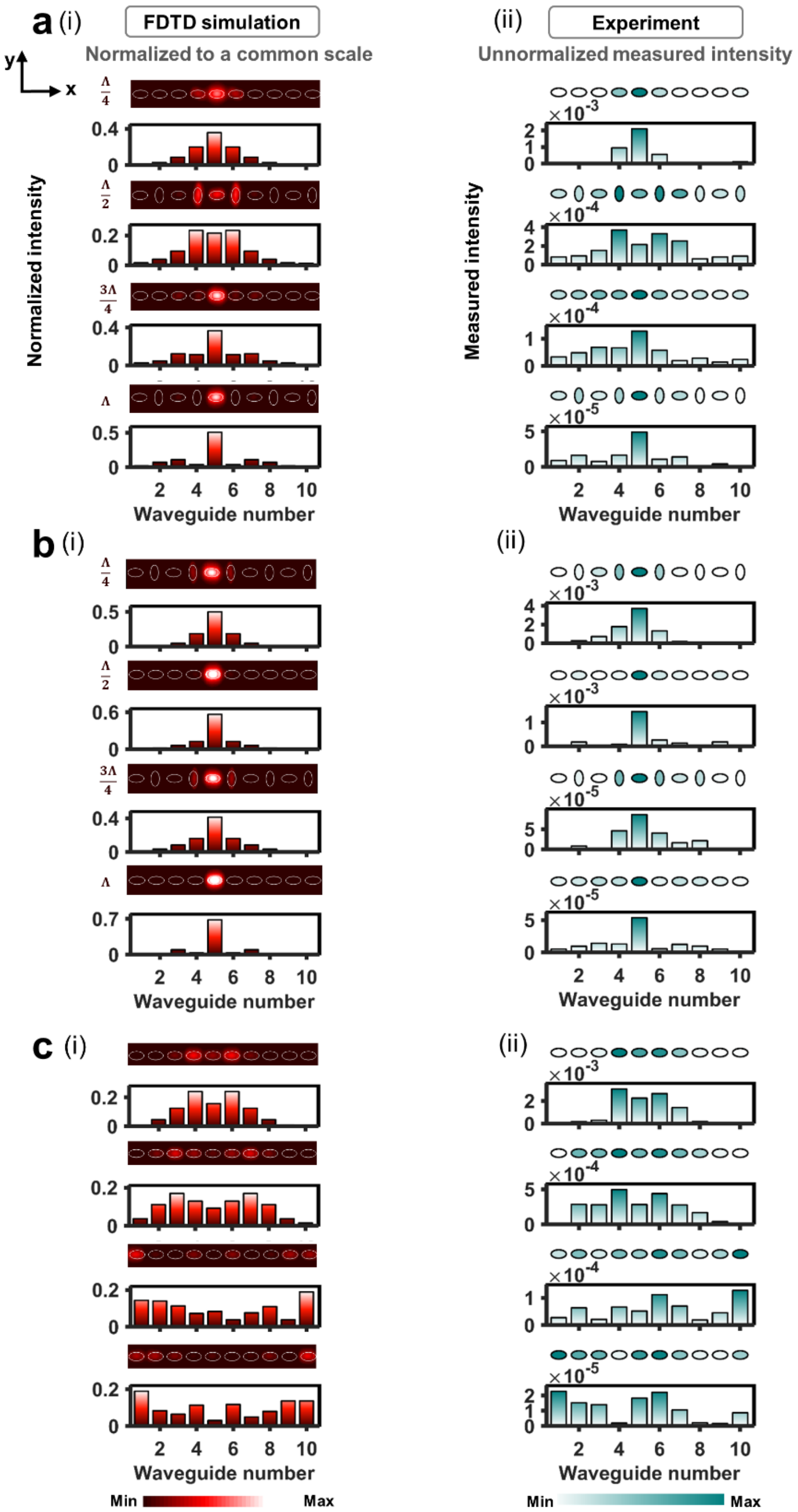

Fig. R6| Comparison between FDTD simulations and experimental measurements for the one-dimensional lattices. a, b, Floquet flat-band dynamics under modulation of $\kappa = \kappa_0 \sin(\theta(z))$ and

$\kappa = \kappa_0 \cos(\theta(z))$, respectively. **c,** Discrete diffraction in the straight-lattice control sample. **(i)** Output intensity distributions extracted from the finite-difference time-domain (FDTD) simulations at propagation distances $\frac{\Lambda}{4}$, $\frac{\Lambda}{2}$, $\frac{3\Lambda}{4}$, and Λ, normalized to a common scale. **(ii)** Corresponding experimentally measured output average intensity distributions over five measurement counts at the same propagation distances, shown as directly measured, unnormalized intensities.

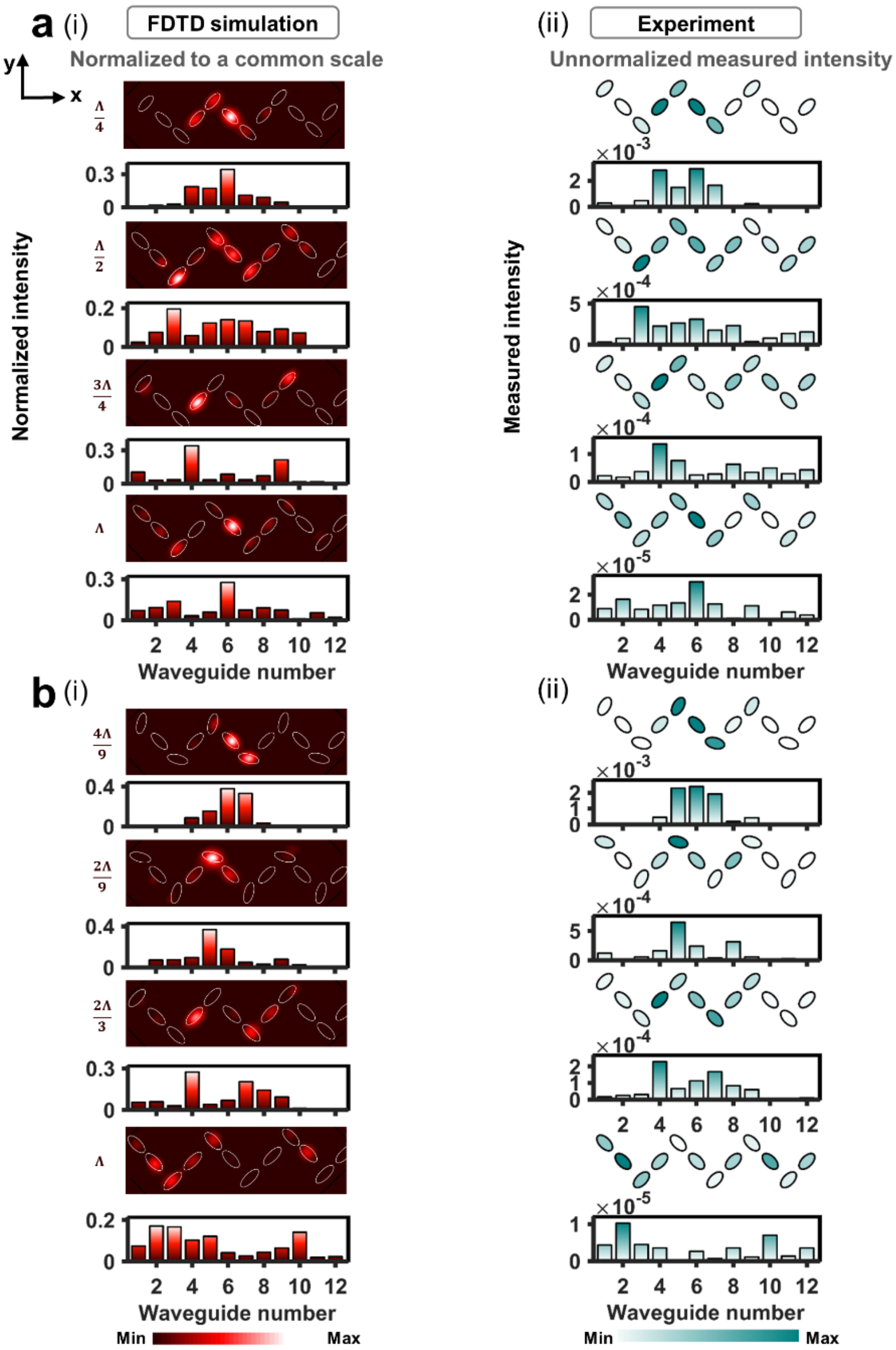

Fig. R7 | Comparison between FDTD simulations and experimental measurements for the period-nΛ oscillation structures. a, Period-Λ oscillation. **b,** Period-2Λ oscillation. **(i)** Output intensity distributions extracted from the finite-difference time-domain (FDTD) simulations at selected propagation distances, normalized to a common scale. **(ii)** Corresponding experimentally

measured average output intensity distributions over five measurement counts at the same propagation distances, shown as directly measured, unnormalized intensities.

We improve the presentation of the experimental results in the revised manuscript, including clearer figures and a more explicit comparison between experiment and theory where appropriate, so that the central physical message is conveyed more convincingly.

In summary, we respectfully believe that the present work is distinct from the prior studies cited by the Reviewer not simply because it involves polarization-related effects, but because it establishes **a new Floquet-capable twisted waveguide coupling platform with direct, continuous, and compact sign-and-magnitude control of inter-waveguide coupling**. We revise the manuscript accordingly to clarify this point more sharply, strengthen the narrative focus, and improve the presentation of the experimental results.

Changes in the manuscript:

I. Originality:

Main text:

As shown above in our response to Reviewer 1, we have revised the Abstract and Introduction to clarify more precisely the originality of the present work. In particular, we now state explicitly that the advance of this work lies not in the generic use of polarization, but in establishing a twisted waveguide Floquet platform that enables direct and continuous control of the nearest-neighbour coupling coefficient in both sign and magnitude.

Figures and tables:

We have also added **Table 1** and revised **Fig. 1,** which to position the present platform against representative previous photonic Floquet platforms in terms of coupling implementation, coupling coefficient sign control, and platform versatility. The revised **Fig. 1** is also shown above in our response to Reviewer 1.

*Table 1 | **Comparison of the present work with representative photonic Floquet platforms.** The table compares this work with representative Floquet photonic platforms in terms of direct nearest-neighbour coupling implementation, controlled physical degree of freedom, $\kappa(z)$ variation within one Floquet period, continuous sign tuning of $\kappa(z)$, and representative physical phenomena. Here, $\kappa(z)$ denotes the coupling coefficient between adjacent lattice sites.*

Floquet photonic platform	Reference	Coupling control: Direct coupling	Coupling control: Controlled physical degree of freedom	Coupling control: $\kappa(z)$ variation within one Floquet period	Coupling control: Continuous sign tuning of $\kappa(z)$	Physical phenomena
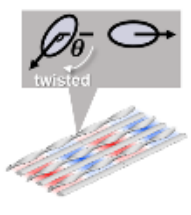 Twisted waveguide array	Our work	√	Relative polarization angle	√	√	Floquet flat bands and period-nΛ oscillations in 1D lattices; chirality reversal of topological states in 2D lattices
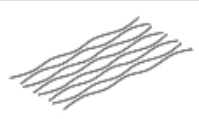 Curved waveguide array	[13]	√	Waveguide spacing	√	×	Anomalous π-edge modes
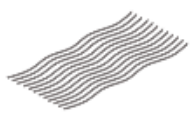 Curved waveguide array	[18]	√	Waveguide bending trajectory	×	×	Artificial gauge field-enabled dispersionless coupling
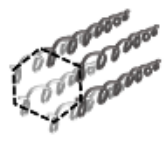 Helical waveguide array	[17]	√	Waveguide helical trajectory	×	×	Floquet topological one-way edge states
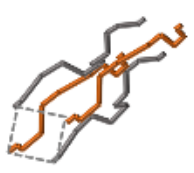 Waveguide array	[24]	√	Waveguide spacing	√	×	Parity-time-symmetric photonic topological insulator
	[25]	√	Waveguide spacing	√	×	Bulk/edge solitons
	[26]	√	Waveguide spacing	√	×	Topological Floquet solitons
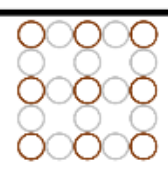 Microring resonators	[30]	×	Resonator spacing, Propagation constant detuning	√	×	Full k-space flat bands
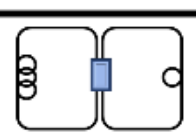 Fiber-loop lattice	[31]	×	Electro-Optic modulation	√	×	Non-Abelian lattice gauge fields

In addition, we have revised the **Introduction** to discuss the representative prior studies raised by the Reviewer 2, namely in dipolar arrays, mixed s/p lattices, and bianisotropic meta-atoms. As now stated in the revised Introduction, these approaches

rely respectively on auxiliaries, and do not provide a general route for the direct and continuous control of the inter-site coupling coefficient in waveguide arrays through the relative polarization angle between adjacent waveguides.

"Polarization is a fundamental internal degree of freedom of light and has long served as a powerful resource for optical control through geometric phases and anisotropic responses[32–34]. More broadly, internal modal degrees of freedom have enabled several nontrivial coupling mechanisms in photonic systems, including tensorial dipole-dipole coupling in dipolar arrays[35], orbital-parity-induced negative coupling in mixed-s/p lattices[36], and orientation-controlled magneto-electric coupling in bianisotropic meta-atoms[37]. These studies highlight the potential of internal degrees of freedom for engineering effective coupling, synthetic flux, and topological states. However, their mechanisms rely on dipolar tensor interactions, engineered orbital parity, or resonant electric magnetic hybridization, rather than on a direct mapping from polarization to inter waveguide coupling. A platform in which the linear polarization of a guided fundamental mode directly controls the modal overlap between adjacent waveguides would provide a compact and geometrically programmable route to continuously control both the magnitude and sign of the nearest-neighbour coupling coefficient, offering a natural driving parameter for Floquet photonic lattices."

References:

35. Zhang, Y., Wu, R. P. H., Shi, L. & Fung, K. H. Second-Order Topological Photonic Modes in Dipolar Arrays. ACS Photonics 7, 2002–2009 (2020).

36. Schulz, J., Noh, J., Benalcazar, W. A., Bahl, G. & Von Freymann, G. Photonic quadrupole topological insulator using orbital-induced synthetic flux. Nat. Commun. 13, 6597 (2022).

37. Bobylev, D. A. et al. Topological Edge and Corner States Designed via Meta-Atoms Orientation. Laser Photonics Rev. 17, 2100567 (2023).

II. Manuscript organization:

We have substantially reorganized the manuscript so that the central storyline is built around the platform itself and its representative 1D and 2D Floquet phenomena. We have revised the Abstract and Introduction accordingly, explicitly stating that the revised manuscript focuses on Floquet flat bands and period-nΛ oscillations in 1D

lattices, and on chirality reversal of topological edge states in 2D lattices. We have also revised Fig. 1 to reflect this refocused presentation, so that the retained examples are presented as representative consequences of the same coupling-engineering capability rather than as disconnected demonstrations.

III. Experimental results:

As shown above in our response to Reviewer 1, we have repeated the key main-text experiments using the THz-VNA platform and replaced the corresponding experimental results in **Figs. 3** and **4** with the new measurements. These new measurements provide much clearer output intensity distributions and are in good agreement with the theoretical analysis, numerical simulations, and our previous THz-TDS results, thereby providing substantially stronger experimental evidence for the revised manuscript. In addition, as also shown above in our response to Reviewer 1, we have revised the first paragraph of the section "**Theoretical Model of Polarization-Controlled Coupling in Twisted Waveguides**" to introduce the THz-TDS and THz-VNA platforms, and we have revised **Fig. 2a** to include both measurement setups. In addition, we have added **Supplementary Note 11** and **Fig. S11** to describe the repeated THz-VNA measurements, **Fig. S14** to quantify the propagation loss and show that the twist introduces nearly no additional loss, and **Figs. S25–S27** to provide further experiment–simulation comparisons and FDTD simulations including the experimentally measured material absorption.

Reply to Reviewer 3:

General Comment:

There has been much efforts to develop methods for controlling the polarization of modes in various photonic platforms. The authors here present a platform with varied application across a variety of manipulation protocols. Though some of these types of manipulations have appeared in the past in various platforms, these twisted waveguide arrays provide maybe a more streamline platform for various control mechanisms.

Most claims seem to be well supported though I have some questions that need to be answered to help solidify this conclusion. Some of these questions also stand to warrant revision as to help readers understand some of the results presented, so they are not mislead or interpreted incorrectly.

After these questions have been responded to, I can have a better sense to recommend or not to recommend the paper for publication?

For the most part methodology seems to be detailed enough to reproduce results.

Responses:

We greatly appreciate your positive comments and detailed summary of our work. Many of the suggestions you have given us are enlightening our research. We have carefully revised and proofread the manuscript according to your suggestions.

Comments1:

In Fig 2c what is the highlighted region between frequency around .3 and .4 THz? Does this correspond to the 1000 micrometer to 1493 micrometer region described in the caption. If so maybe the units should be change to this connection is clearer.

Responses:

We thank the Reviewer for the comment. The highlighted region corresponds to

the frequency range of 0.2 and 0.3 THz, which is consistent with the operational wavelength range of 1000 μm to 1493 μm mentioned in the caption, as obtained via the relation $\lambda = \frac{c}{f}$. Because spectral plots are conventionally presented in the frequency domain, we have retained frequency on the lower horizontal axis and, to clarify the correspondence, added the equivalent wavelength scale to the upper horizontal axis.

Changes in the manuscript:

To make this connection immediately clear to the reader, we label the upper horizontal axis of the figure with the corresponding wavelength scale in Fig. 2c.

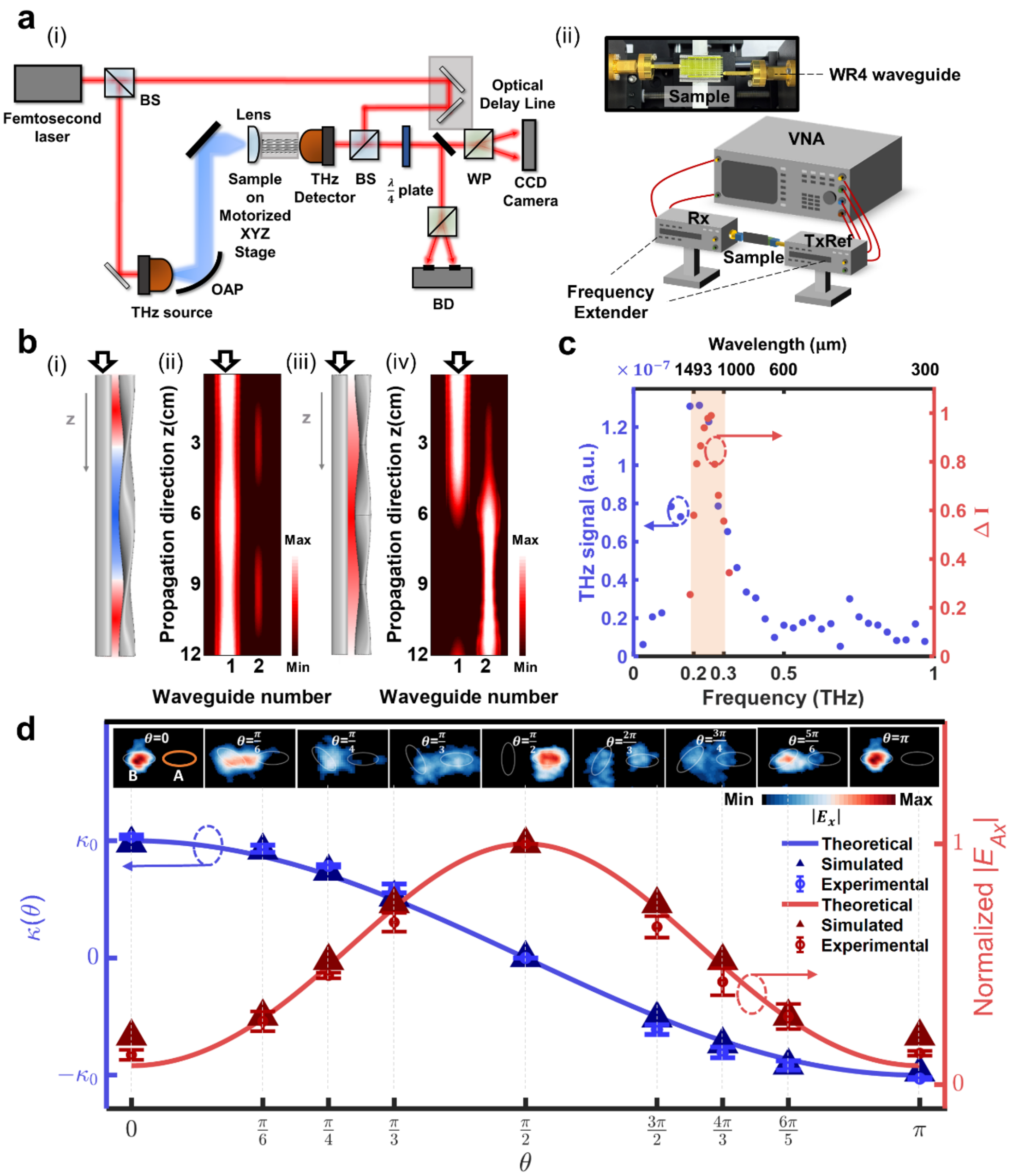

*Fig. 2 | Experimental verification of polarization-controlled coupling in twisted waveguides. a, Measurement setups. **(i)** THz-TDS measurement setup. A femtosecond laser pumps a Terahertz source, and the transmitted signal through the sample is collected by a balanced detector. The sample is mounted on a motorized XYZ stage for spatial movement. OAP, off-axis parabolic mirror; BS, beam splitter; BD, balance detector; WP: Wollaston prism.* ***(ii)*** THz-VNA measurement setup, consisting of a VNA, two frequency extension modules, and the sample placed between them. The TxRef module serves as the transmitting and reference port for terahertz signal generation and coherent detection, whereas the Rx module receives the transmitted terahertz signal. Two WR4 metallic waveguides are connected to the sample to couple the terahertz signal into the structure and

collect the transmitted output. The inset is a photograph of the corresponding experimental setup. ***b,*** *FDTD simulations of light propagation in two parallel elliptical waveguides. In* ***(i–ii)****, one waveguide is twisted by* 2π *along the propagation direction, leading to a coupling coefficient of* $\kappa(z)=\kappa_0 cos\theta(z)$. *In* ***(iii–iv)****, as a comparison, the twisted waveguide undergoes alternating rotations of* $\pm\frac{\pi}{2}$, *yielding* $\kappa(z)=\kappa_0|cos\theta(z)|$. *The contrasting propagation dynamics highlight the impact of coupling sign.* ***c****, The TE mode supported by a single waveguide defines the spectral range, while the operational bandwidth is identified from the relative output intensity variation* $\Delta I(\lambda)$. *Within the wavelength range of 1000* μm *to 1493* μm, *those satisfying* $\Delta I > 50\%$ *are defined as being within the operational bandwidth.* ***d,*** *Experimental and theoretical results for nine pairs of parallel waveguides with different relative polarization angles* θ. *Top: Electric field distribution (*$|E_{Ax}|$*) measured at the output facet after 1 cm of propagation, with normalized intensity plotted as a function of* θ. *Orange dashed circles highlight the injected waveguides. Bottom: Extracted coupling coefficient* $\kappa(\theta)$. *Solid lines represent theoretical predictions, while points indicate simulation and experimental results. The error bars are derived from the standard deviation.*

Comments2:

A lot of effort is made at describing the effect of "negative coupling" as if to make a distinction between positive and negative coupling between the wave guides. The experimental result demonstrates two different signal profiles (fig. 2b(ii) and 2b(iv)) for two different coupling functions. Is it clear that the sign of the coupling and not the functional form of the coupling is the important characteristic. Its not clear to me that the role of the functional form can plays as much as a role in the measured signal profile as the sign.

Responses:

We thank the reviewer for raising this important point, which allows us to clarify our rationale. In Figs. 2b(ii) and 2b(iv), the absolute-value function $\kappa(z)=\kappa_0|\cos\theta(z)|$ is always non-negative, while the cosine function $\kappa(z)=\kappa_0\cos\theta(z)$

alternates between positive and negative values, where $\theta(z) = \frac{2\pi z}{\Lambda}$.

The functional form of the coupling can also influence the measured signal profile. To illustrate this, we further examine three types of coupling modulation between two waveguides with different functional forms:

$$\kappa(z) = \begin{cases} \kappa_0 \dfrac{\frac{\Lambda}{4} - z}{\frac{\Lambda}{4}} & \text{if } 0 < z < \dfrac{\Lambda}{2} \\ \kappa_0 \dfrac{z - \frac{3\Lambda}{4}}{\frac{\Lambda}{4}} & \text{if } \dfrac{\Lambda}{2} < z < \Lambda \end{cases},$$

$$\kappa(z) = \begin{cases} \kappa_0 \dfrac{z}{\frac{\Lambda}{4}} & \text{if } 0 < z < \dfrac{\Lambda}{4} \\ \kappa_0 \dfrac{\frac{\Lambda}{2} - z}{\frac{\Lambda}{4}} & \text{if } \dfrac{\Lambda}{4} < z < \dfrac{3\Lambda}{4}, \\ \kappa_0 \dfrac{z - \Lambda}{\frac{\Lambda}{4}} & \text{if } \dfrac{3\Lambda}{4} < z < \Lambda \end{cases}$$

$$\kappa(z) = \kappa_0 \sin\theta(z).$$

The corresponding state evolutions are shown in Fig. R12d, e, and f(ii). And the driving period Λ is identical. In particular, for the coupling form $\kappa(z) = \kappa_0 \sin\theta(z)$, the geometric configuration of the two waveguides is presented in Supplementary Fig. R12f(i), and the corresponding simulation results are shown in Supplementary Fig. R12f(iii).

These results indicate that not only the sign of the coupling coefficient, but also its functional form, can affect the evolution of the optical field. In Fig. R12b(ii), d, e, and f(ii), the driving period Λ is identical. For the comparisons between Fig. R12b(ii) and d, as well as between e and f(ii), the positions where the coupling sign reverses remain unchanged. The differences in the field evolution therefore arise solely from the distinct functional forms of the coupling coefficient, which lead to different coupling magnitudes at each propagation position. Consequently, the resulting field propagation

is not exactly identical. Nevertheless, for the physical phenomenon investigated in this work, the sign reversal of the coupling coefficient plays the dominant role, while the functional form mainly introduces quantitative differences in the detailed field evolution.

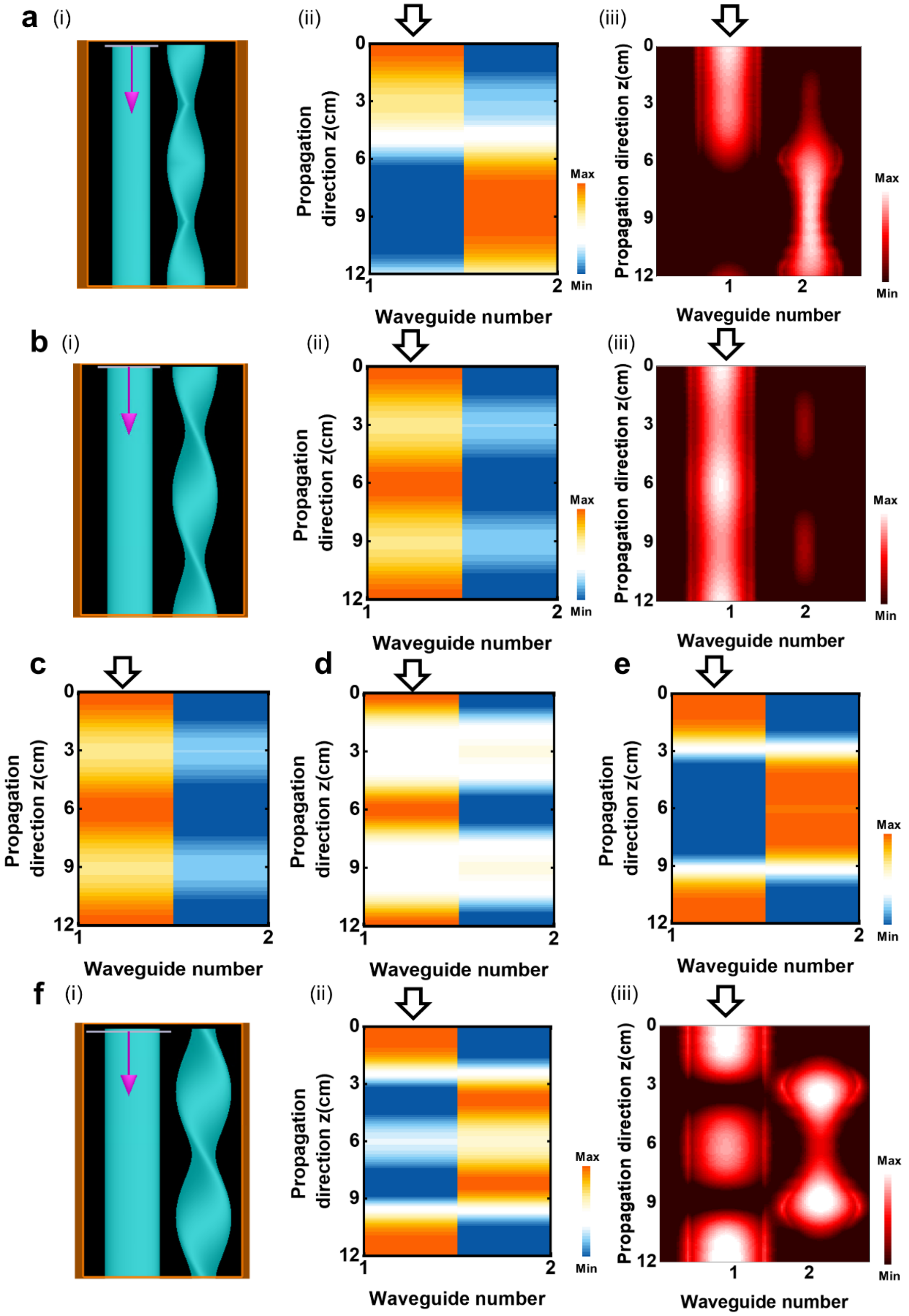

Fig. R12 | Verification of negative coupling and the roles of its sign and functional form. a, (i) Schematic of the first twisting configuration, where two initially aligned TE-polarized waveguides undergo successive clockwise and counterclockwise $\frac{\pi}{2}$ twists, resulting in an effective coupling coefficient $\kappa(z) = \kappa_0|\cos(\theta(z))|$. (ii) Calculated coupling distribution and (iii) simulated field evolution over one driving period (Λ = 12 cm). **b**, (i) Schematic of the second configuration, in which the right waveguide is twisted continuously by 2π clockwise, yielding $\kappa(z) = \kappa_0\cos(\theta(z))$. (ii) Corresponding coupling distribution and (iii) simulated field evolution, showing a markedly different behavior from a despite identical structural parameters (waveguide spacing d = 1000 μm, semi-major axis a = 350 μm, semi-minor axis b = 150 μm). **c**, Numerical verification of the equivalence between $\kappa(z) = \kappa_0\cos(\theta(z))$ and $\kappa'(z) = \kappa_0|\cos(\theta(z))|$, where the negative sign is absorbed into the state vector component, effectively corresponding to the addition of a negative on-site potential. **d**, Numerical state evolution of coupling coefficient $\kappa(z) =$

$$\begin{cases} \kappa_0 \frac{\frac{\Lambda}{4} - z}{\frac{\Lambda}{4}} & \text{if } 0 < z < \frac{\Lambda}{2} \\ \kappa_0 \frac{z - \frac{3\Lambda}{4}}{\frac{\Lambda}{4}} & \text{if } \frac{\Lambda}{2} < z < \Lambda \end{cases}$$

. **e**, Numerical state evolution of coupling coefficient

$$\kappa(z) = \begin{cases} \kappa_0 \frac{z}{\frac{\Lambda}{4}} & if\ 0 < z < \frac{\Lambda}{4} \\ \kappa_0 \frac{\frac{\Lambda}{2} - z}{\frac{\Lambda}{4}} & if\ \frac{\Lambda}{4} < z < \frac{3\Lambda}{4} \\ \kappa_0 \frac{z - \Lambda}{\frac{\Lambda}{4}} & if\ \frac{3\Lambda}{4} < z < \Lambda \end{cases}$$

. **f**, (i) Schematic of the twisting configuration, where the major axes of the elliptical cross-sections at the initial positions of the two waveguides are perpendicular to each other, and the right waveguide is twisted continuously by 2π clockwise, resulting in an effective coupling coefficient $\kappa(z) = \kappa_0\sin(\theta(z))$. (ii) Calculated coupling distribution and (iii) simulated field evolution over one driving period (Λ = 12 cm).

Changes in the manuscript:

We add one sentence to the first paragraph of the section titled "**Theoretical Model of Polarization-Controlled Coupling in Twisted Waveguides**" in the main text.

"For the physical phenomenon discussed below, the sign reversal of the coupling coefficient plays the primary role, while the functional form mainly introduces secondary quantitative modifications to the measured signal profile."

And we add the description about the roles of the sign and functional form of the coupling coefficient in the supplementary materials under the subtitle "**Supplementary Note 1 | Derivation of the negative coupling coefficient and analysis of the roles of its sign and functional form.**". And we add the corresponding figures in Fig. S1.

"*To clarify the respective roles of the sign and the functional form of the coupling coefficient, we compare several coupling modulations between two waveguides. In all cases, the driving period Λ is kept identical to ensure a consistent modulation scale along the propagation direction.*

The corresponding waveguide geometries and simulated state evolutions are shown in Fig. S1. These results indicate that both the sign and the functional form of the coupling coefficient can influence the propagation dynamics of the optical field. When the coupling sign changes along the propagation direction, the effective phase relation between the coupled modes is modified, which leads to qualitatively different evolution behaviors. In contrast, variations in the functional form mainly affect the local magnitude of the coupling strength at each propagation position, thereby introducing quantitative differences in the detailed signal profile.

For example, in Fig. S1b(ii), d, e, and f(ii), the coupling coefficients are

$$\kappa(z) = \kappa_0 cos\theta(z),$$

$$\kappa(z) = \begin{cases} \kappa_0 \dfrac{\dfrac{\Lambda}{4} - z}{\dfrac{\Lambda}{4}} & if\ 0 < z < \dfrac{\Lambda}{2} \\ \kappa_0 \dfrac{z - \dfrac{3\Lambda}{4}}{\dfrac{\Lambda}{4}} & if\ \dfrac{\Lambda}{2} < z < \Lambda \end{cases},$$

$$\kappa(z) = \begin{cases} \kappa_0 \dfrac{z}{\frac{\Lambda}{4}} & if\ 0 < z < \dfrac{\Lambda}{4} \\ \kappa_0 \dfrac{\frac{\Lambda}{2} - z}{\frac{\Lambda}{4}} & if\ \dfrac{\Lambda}{4} < z < \dfrac{3\Lambda}{4}, \\ \kappa_0 \dfrac{z - \Lambda}{\frac{\Lambda}{4}} & if\ \dfrac{3\Lambda}{4} < z < \Lambda \end{cases}$$

$$\kappa(z) = \kappa_0 sin\theta(z).$$

When comparing Fig. S1b(ii) with d, or e with f(ii), the positions where the coupling sign reverses remain unchanged, and the differences in the field evolution arise solely from the different functional forms of $\kappa(z)$*, which lead to different coupling amplitudes along the propagation direction. Consequently, the resulting field distributions are not exactly identical.*

Nevertheless, for the physical phenomenon investigated in the main text, the sign reversal of the coupling coefficient plays the primary role, while the functional form mainly introduces secondary quantitative modifications to the measured signal profile.”

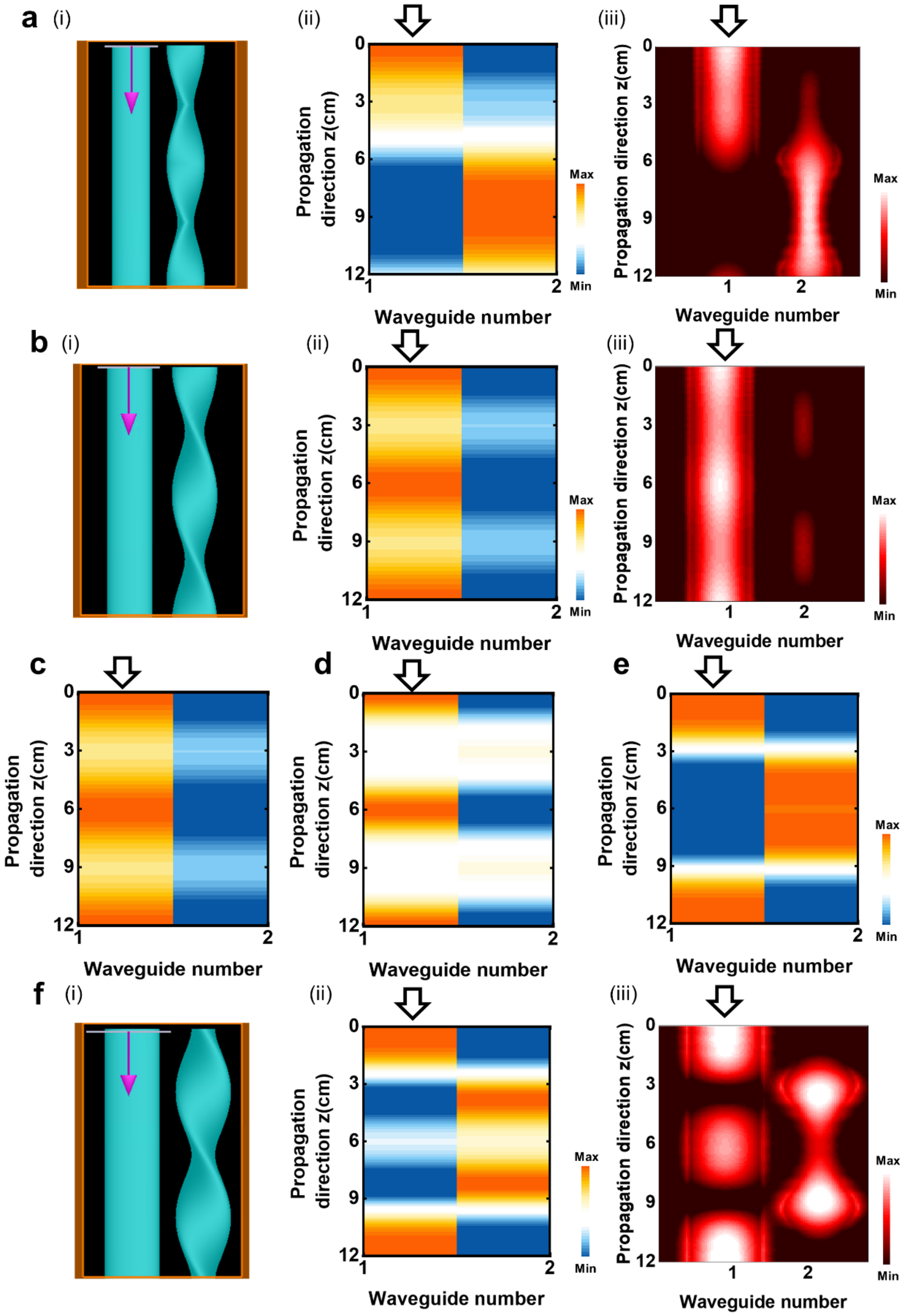

***Fig. S1* | *Verification of negative coupling and the roles of its sign and functional form. a*, *(i) Schematic of the first twisting configuration, where two initially aligned TE-polarized waveguides undergo successive clockwise and counterclockwise* $\frac{\pi}{2}$ *twists, resulting in an effective coupling coefficient* $\kappa(z) = \kappa_0|cos(\theta(z))|$. *(ii) Calculated coupling distribution and (iii) simulated field*

evolution over one driving period (Λ = 12 cm). ***b****, (i) Schematic of the second configuration, in which the right waveguide is twisted continuously by* 2π *clockwise, yielding* $\kappa(z) = \kappa_0 cos(\theta(z))$*. (ii) Corresponding coupling distribution and (iii) simulated field evolution, showing a markedly different behavior from a despite identical structural parameters (waveguide spacing* d *= 1000 μm, semi-major axis* a *= 350 μm, semi-minor axis* b *= 150 μm).* ***c****, Numerical verification of the equivalence between* $\kappa(z) = \kappa_0 cos(\theta(z))$ *and* $\kappa'(z) = \kappa_0 |cos(\theta(z))|$*, where the negative sign is absorbed into the state vector component, effectively corresponding to the addition of a negative on-site potential.* ***d****, Numerical state evolution of coupling coefficient* $\kappa(z) = \begin{cases} \kappa_0 \frac{\frac{\Lambda}{4} - z}{\frac{\Lambda}{4}} & if\ 0 < z < \frac{\Lambda}{2} \\ \kappa_0 \frac{z - \frac{3\Lambda}{4}}{\frac{\Lambda}{4}} & if\ \frac{\Lambda}{2} < z < \Lambda \end{cases}$*.* ***e****, Numerical state evolution of coupling coefficient* $\kappa(z) = \begin{cases} \kappa_0 \frac{z}{\frac{\Lambda}{4}} & if\ 0 < z < \frac{\Lambda}{4} \\ \kappa_0 \frac{\frac{\Lambda}{2} - z}{\frac{\Lambda}{4}} & if\ \frac{\Lambda}{4} < z < \frac{3\Lambda}{4} \\ \kappa_0 \frac{z - \Lambda}{\frac{\Lambda}{4}} & if\ \frac{3\Lambda}{4} < z < \Lambda \end{cases}$*.* ***f****, (i) Schematic of the twisting configuration, where the major axes of the elliptical cross-sections at the initial positions of the two waveguides are perpendicular to each other, and the right waveguide is twisted continuously by* 2π *clockwise, resulting in an effective coupling coefficient* $\kappa(z) = \kappa_0 sin(\theta(z))$*. (ii) Calculated coupling distribution and (iii) simulated field evolution over one driving period (Λ = 12 cm).*

Comments3:

Just for clarification: the results shown in Fig 2d are for waveguides of fixed coupling (i.e. no dependence on propagation coordinate 'z'). For top panel Fig 2d, is it correct that the input is in the waveguide ellipse labeled B and the output A (this is worth specifying in caption I think).

Responses:

The reviewer is correct that the experiments in Fig. 2d are indeed for static waveguides, each fabricated with a fixed relative polarization angle θ, to isolate and verify the fundamental relationship $\kappa(z) = \kappa_0 \cos\theta(z)$.

However, in the top panel, the input light is launched into waveguide A (marked by the orange circle in the schematic), and the output intensities were measured for both waveguides A and B. To clearly illustrate the influence of the twist angle θ on the coupling coefficient, we selected a configuration where, at $\theta = 0$, the optical field is precisely at the first complete coupling from waveguide A to waveguide B. Consequently, in the first and last panels, the output field is entirely localized in waveguide B, as shown in the figure. We have clarified this point in the revised figure caption to avoid any ambiguity.

Changes in the manuscript:

We add sentences to the first paragraph of the section titled “**Theoretical Model of Polarization-Controlled Coupling in Twisted Waveguides**” in the main text.

“In the top panel of Fig. 2d, the input light is injected into waveguide A (indicated by the orange circle), and the output intensities are measured for both waveguides A and B. At $\theta = 0$, the optical field is exactly at the first complete coupling from A to B, resulting in the output field being entirely localized in waveguide B in the first and last panels.”

Comments4:

For Fig. 2c, I think I understand the red curve, what is the measured THz signal (blue dots)?

Responses:

We thank the reviewer for the questions regarding Fig. 2c. The blue dots represent the measured output intensity from our waveguide structure in a terahertz time-domain spectroscopy (THz-TDS) system at different frequencies. This measured THz signal is influenced both by the frequency-dependent response of the THz-TDS system itself and by the transmission characteristics of our waveguide structure. Therefore, the blue dots effectively indicate the operational frequency range of our experimental setup.

The red dots show the variation in output intensity (ΔI) as a function of frequency under different twist angles θ. These dots demonstrate how the modulation depth induced by the twist angle changes with frequency, thereby illustrating the operational bandwidth of the proposed structure.

Changes in the manuscript:

We add sentences to the first paragraph of the section titled "**Theoretical Model of Polarization-Controlled Coupling in Twisted Waveguides**" in the main text.

"To experimentally verify the relationship between the coupling coefficient and the relative polarization angle, nine groups of resin waveguides are fabricated with, each 1cm in length. In Fig. 2c, the blue dots represent the measured THz-TDS output intensity from the waveguide structure, indicating the operational frequency range of our experiment. The red dots show the twist-angle-induced intensity variation ΔI as a function of frequency, demonstrating the bandwidth of the structure."

Comments5:

For Fig. 2c, what is the dashed circles in blue and red highlighting?

Responses:

We apologize for the lack of clarity. The dashed circles are used to indicate the corresponding vertical axes for the two datasets. The blue dashed circle points to the left vertical axis, which corresponds to the THz signal (blue dots), while the red dashed circle points to the right vertical axis, which corresponds to ΔI (red dots). This graphical convention helps to clearly associate each dataset with its respective scale.

Changes in the manuscript:

We add one sentence to the first paragraph of the section titled "**Theoretical Model of Polarization-Controlled Coupling in Twisted Waveguides**" in the main text.

"The blue and red dashed circles point to the corresponding left and right vertical axes,

respectively. Each waveguide in the resin-based array is defined relative polarization angles of 0, $\frac{\pi}{6}$, $\frac{\pi}{4}$, $\frac{\pi}{3}$, $\frac{\pi}{2}$, $\frac{2\pi}{3}$, $\frac{3\pi}{4}$, $\frac{5\pi}{6}$, *and* π, *separately."*

Comments6:

Fig 4a. Was a little confused about what A and B are. Are they the integrated areas under the curve in Fig. 4a(i) as depicted? This would make units make sense in 4a(ii). Again I think this is worth spelling out in the text/caption.

Responses:

We thank the reviewer for the thoughtful comment regarding Fig. 4a. We apologize for the lack of clarity in the original caption and have revised the text accordingly.

To clarify, in Fig. 4a(i), we illustrate the coupling coefficient $\kappa(z)$ (in units of m^{-1}) over one driving period Λ (in units of m). The driving period is divided into two segments with different twisting rates, corresponding to two distinct areas under the $\kappa(z)$ curve. Specifically, "A" and "B" denote the integrated areas under the curve for the first and second halves of the period, respectively. Since $\kappa(z)$ is plotted as a function of propagation distance z, these integrals yield dimensionless quantities.

As shown in Fig. R13a(ii) and (iii), the difference $\Delta = |\mathrm{A} - \mathrm{B}|$ determines the quasi-energy of the flat band, given by $\varepsilon_{flat} = \Delta/\Lambda$. Given that Δ is dimensionless and Λ has units of meters, ε_{flat} indeed has units of m^{-1}, which is consistent with its physical meaning as the quasi-energy per driving period. We have now explicitly stated this relationship and the corresponding units in the revised caption to avoid any confusion.

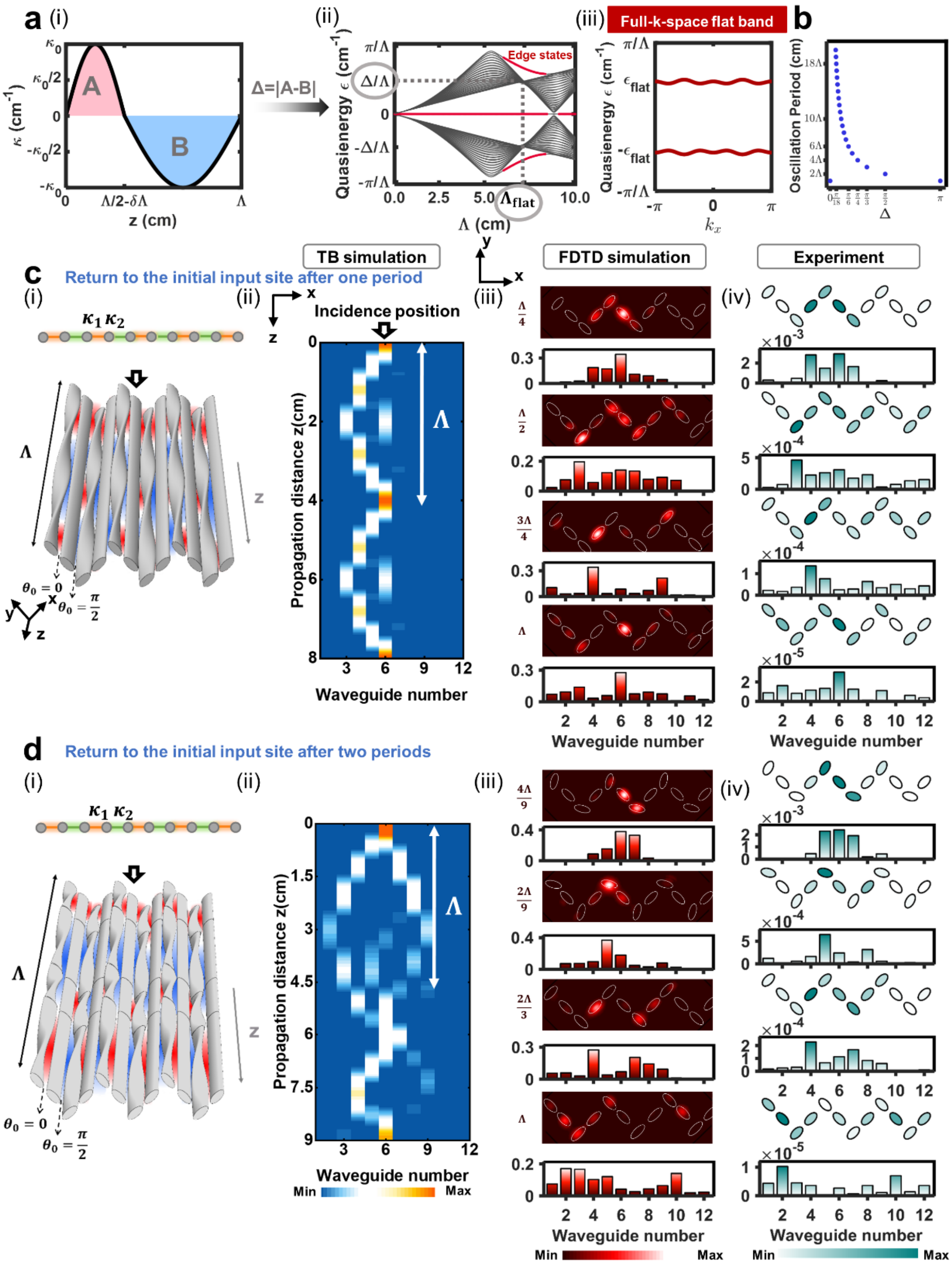

Fig. R13 | Period-nΛ oscillations enabled by polarization-controlled Floquet engineering. a, Schematic illustration of periodic polarization modulation with different twisting rates. The modulation amplitude $\Delta = |A - B|$ determines the band structure and phase. **(i)** Two regions A and B represent different twisting rates during the first and second halves of the driving period. **(ii)** The corresponding quasi-energy spectrum under open boundary conditions, calculated for an array of eighty waveguides, exhibits flat bands at Λ_{flat}. **(iii)** The corresponding quasi-energy spectrum under

periodic boundary conditions under driving period Λ_{flat} shows flat bands spanning the entire Brillouin zone with the value of Δ/Λ. **b**, Oscillation period of the Floquet states as a function of Δ. **c, d,** Twelve waveguides spaced by 850 μm form an array over a propagation distance of $\Lambda =$ 4 cm and 4.5 cm, respectively. Experimental demonstration of period-nΛ oscillations for $\text{n} = 1$ and $\text{n} = 2$, respectively. In all cases, light is injected into the central waveguide. The TB simulations are shown in **(ii)**, the FDTD simulations in **(iii)**, and the experimental results in **(iv)**. The FDTD results are normalized to a common scale across different propagation distances, whereas the experimental bar plots show directly measured, unnormalized output intensities. **c,** Period-Λ oscillation. **(i)** Schematic of the twisted waveguide array with identical twisting rates. **(ii)** TB simulation showing revival after one driving period Λ. **(iii)** FDTD output intensity distributions at propagation distances $\frac{\Lambda}{4}$, $\frac{\Lambda}{2}$, $\frac{3\Lambda}{4}$, and Λ. **(iv)** Experimentally measured output intensity distributions at the same propagation distances, confirming the period-Λ oscillation. **d,** Period-2Λ oscillation. **(i)** Schematic of the twisted waveguide array with different twisting rates. **(ii)** TB simulation showing revival after two driving periods. **(iii)** FDTD output intensity distributions at propagation distances $\frac{2\Lambda}{9}$, $\frac{4\Lambda}{9}$, $\frac{2\Lambda}{3}$, and Λ. **(iv)** Experimentally measured output intensity distributions at the same propagation distances.

Changes in the manuscript:

We add sentences to the fourth paragraph of the section titled "**Floquet Flat Bands and Period-nΛ Oscillations**" in the main text.

"In Fig. 4a(i), the coupling coefficient $\kappa(z)$ over one driving period Λ is shown. The integrated areas under the curve for the first and second halves of the period are denoted as A and B, respectively, and their difference $\Delta = |A - B|$ (dimensionless) determines the quasi-energy $\varepsilon_{flat} = \Delta/\Lambda$ of the flat band, as plotted in Fig. 4a(ii) and (iii)."

And axis units have now been included in Fig. 4 and in all other figures requiring them accordingly.

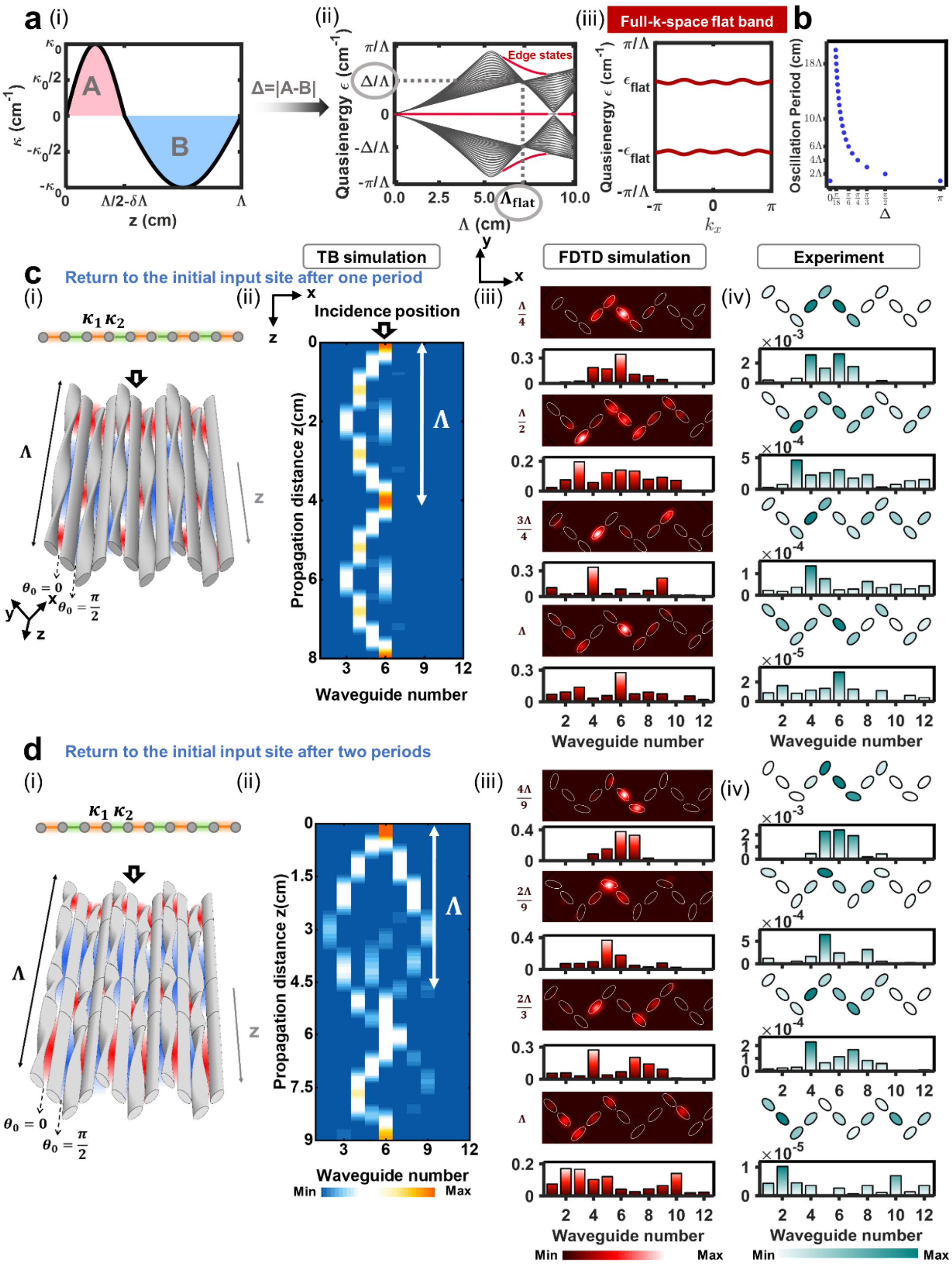

Fig. 4 | ***Period-*** $n\Lambda$ ***oscillations enabled by polarization-controlled Floquet engineering. a,*** *Schematic illustration of periodic polarization modulation with different twisting rates. The modulation amplitude* $\Delta = |A - B|$ *determines the band structure and phase.* ***(i)*** *Two regions A and B represent different twisting rates during the first and second halves of the driving period.* ***(ii)*** *The corresponding quasi-energy spectrum under open boundary conditions, calculated for an array of eighty waveguides, exhibits flat bands at* Λ_{flat}. ***(iii)*** *The corresponding quasi-energy spectrum*

under periodic boundary conditions under driving period Λ_{flat} shows flat bands spanning the entire Brillouin zone with the value of Δ/Λ. ***b****, Oscillation period of the Floquet states as a function of Δ.* ***c, d,*** *Twelve waveguides spaced by 850 μm form an array over a propagation distance of $\Lambda = 4$ cm and 4.5 cm, respectively. Experimental demonstration of period-$n\Lambda$ oscillations for $n = 1$ and $n = 2$, respectively. In all cases, light is injected into the central waveguide. The TB simulations are shown in* ***(ii)****, the FDTD simulations in* ***(iii)****, and the experimental results in* ***(iv)****. The FDTD results are normalized to a common scale across different propagation distances, whereas the experimental bar plots show directly measured, unnormalized output intensities.* ***c,*** *Period-Λ oscillation.* ***(i)*** *Schematic of the twisted waveguide array with identical twisting rates.* ***(ii)*** *TB simulation showing revival after one driving period Λ.* ***(iii)*** *FDTD output intensity distributions at propagation distances $\frac{\Lambda}{4}$, $\frac{\Lambda}{2}$, $\frac{3\Lambda}{4}$, and Λ.* ***(iv)*** *Experimentally measured output intensity distributions at the same propagation distances, confirming the period-Λ oscillation.* ***d,*** *Period-2Λ oscillation.* ***(i)*** *Schematic of the twisted waveguide array with different twisting rates.* ***(ii)*** *TB simulation showing revival after two driving periods.* ***(iii)*** *FDTD output intensity distributions at propagation distances $\frac{2\Lambda}{9}$, $\frac{4\Lambda}{9}$, $\frac{2\Lambda}{3}$, and Λ.* ***(iv)*** *Experimentally measured output intensity distributions at the same propagation distances.*

Comments7:

Fig 2d. Why does simulation deviate from experiment and theory when θ equals around 0 and π for red curve?

Responses:

We thank the reviewer for this careful observation. In the ideal theoretical model, when $\theta = 0$ or $\theta = \pi$, the coupling configuration corresponds to a condition where the transmission from waveguide A is expected to vanish, and therefore the normalized field intensity should approach zero.

However, in both the simulations and the experiments, a small but finite signal can still be observed. This deviation mainly arises from several practical factors that are not

included in the idealized theoretical model. In the theoretical model, only the TE modes polarized along the major axis of the elliptical waveguide are considered to couple with each other. In the practical simulation and experimental systems, however, although the TE mode (polarized along the major axis) and the TM mode (polarized along the minor axis) are already lifted from degeneracy, a very weak residual coupling between these modes may still exist. This weak intermodal coupling can lead to a small but non-zero signal even when the idealized theory predicts vanishing transmission.

Therefore, although the ideal theoretical prediction suggests zero transmission at $\theta = 0$ and $\theta = \pi$, the small residual signals observed in both simulation and experiment are expected and remain consistent with the overall theoretical trend. Importantly, these small deviations do not affect the overall agreement between theory, simulation, and experiment, nor the main physical conclusions of the work.

Changes in the manuscript:

We add sentences to the first paragraph of the section titled "**Theoretical Model of Polarization-Controlled Coupling in Twisted Waveguides**" in the main text.

"In the practical simulation and experimental systems, however, although the TE and TM modes are lifted from degeneracy, weak residual intermodal coupling may still occur. Together with fabrication imperfections and slight deviations in waveguide geometry, this can lead to a small but non-zero signal near $\theta = 0$ and $\theta = \pi$, where the idealized theory predicts vanishing transmission. Importantly, these small deviations do not affect the overall agreement between theory, simulation, and experiment, nor the main physical conclusions of the work."

Comments8:

Fig 4c(iii) and 4d(iii): I think the ellipses are representing the 12 waveguide in the array. Might be useful to mention there zigzag pattern (as shown in fig. 4c(i) and 4d(i), but a little hard to see) as the axes in fig. 4c(i) and 4d(i) are rotated relative

to fig 4c(iii) and 4d(iii). Or to just indicate direction along the 1D-chain in figure.

Responses:

We thank the reviewer for this helpful suggestion. In Fig. 4c(iii) and 4d(iii), the ellipses indeed represent the 12 waveguides in the array. We arranged the waveguides along the diagonal in the experiment to ensure that the polarization supported by the source and detector is aligned with the polarization defined by the waveguide cross-sectional geometry. To improve clarity, we have unified the coordinate orientations in Fig. 4c(i), 4d(i) and Fig. 4c(iii), 4d(iii), and enhanced the visibility of the waveguide positions by bolding the elliptical markers in Fig. 4c(iii) and 4d(iii).

Changes in the manuscript:

We have redrawn Fig. 4 to improve the visual clarity and consistency of the experimental geometry. Specifically, we have unified the coordinate orientations between Fig. 4c(i), 4d(i) and Fig. 4c(iii)-(iv), 4d(iii)-(iv), and enhanced the visibility of the waveguide positions by bolding the elliptical markers in the experimental panels. The revised Fig. 4 has been provided above in our response to Comments6.

Comments9:

Fig 5e: Just want to confirm that these are all theoretical. If so, was wondering if the experiment has been done on this system. If it has not, I think it is important to make it clear that this topological edge state behavior in this 2D honeycomb model has not been experimentally verified in this platform.

Responses:

We thank the reviewer for this important question. The results presented in Fig. 5e are indeed purely theoretical, as we mentioned *"Theoretical simulations of light propagation reveal the spatial distribution of the optical field at the output facet after different propagation distances, corresponding to a CW edge state, bidirectional edge transport near the phase transition, and CCW edge state, respectively, as shown in Figs.*

5c(i)-(iii)." in the third paragraph of the section titled "**Topological Phase Transitions and Chirality Reversal of Edge States**". At present, the corresponding experiment has not been carried out for this system. The main limitation arises from the physical scale of the proposed two-dimensional lattice. In our design, a single evolution period extends over several centimeters, and observing clear topological edge-state dynamics would require propagation over multiple periods, resulting in a total length on the order of tens of centimeters. However, the 3D-printed resin material used in our platform exhibits relatively strong absorption at terahertz frequencies, which leads to significant signal attenuation over such long propagation distances, making reliable experimental observation challenging. To avoid confusion, we have revised the manuscript to explicitly clarify that the results in Fig. 5e are theoretical predictions and that experimental verification in this specific platform remains an open challenge. We note that future improvements in low-loss materials or alternative fabrication platforms may enable experimental realization of this system.

Changes in the manuscript:

We add one sentence to the third paragraph of the section titled "**Topological Phase Transitions and Chirality Reversal of Edge States**" in the main text.

"Experimental realization in the present platform is challenging due to the large propagation length required (on the order of tens of centimeters) and the relatively high absorption of the 3D-printed resin at terahertz frequencies."

Meanwhile, we have also explicitly labeled the corresponding results in Fig. 5c as "*TB simulation*" to clarify that they are obtained by numerically propagating the tight-binding equations.

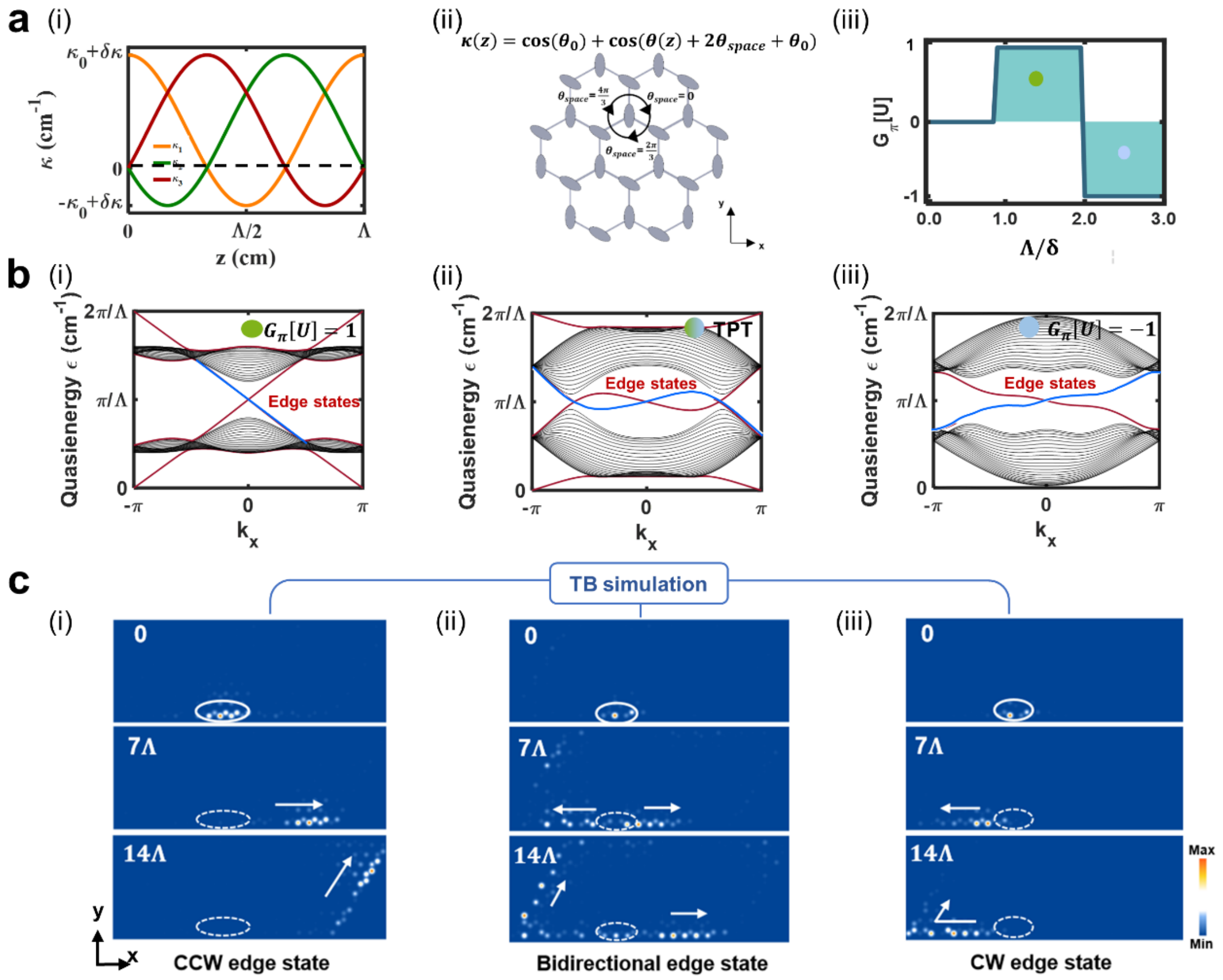

Fig. 5 | Chirality reversal of topological edge states in two-dimension Floquet photonic lattices. ***a**, Topological properties of a two-dimensional lattice. **(i)** Coupling coefficient* κ_1, κ_2 *and* κ_3 *as a function of propagation distance* z. ***(ii)** Schematic of the cross section of the dynamically modulated waveguide array with polarization modulation. **(iii)** Phase diagram of the driven system indicating the emergence of topological phases. **b**, Quasi-energy spectra for different types of edge states in two-dimensional lattices under open boundary conditions projected along the zigzag edge. **(i)** Counter-clockwise (CCW) propagating edge state under one topological phase. **(ii)** Bidirectional edge states at the topological phase transition (TPT) point. **(iii)** Clockwise (CW) propagating edge state. Red and blue curves indicate topologically protected edge states. **c**, TB simulated propagation of 2D Floquet edge states. **(i)** Excitation at the lower edge shows robust CCW edge transport. **(ii)** At the TPT point, bidirectional edge states emerge. **(iii)** Edge transport direction reverses to CW. White dashed circles highlight the injected regions.*

Comments10:

Fig 6c: (i) shows which ellipse corresponds to A, B, C, and X. If I understand correctly, in the inset in (ii) showing the three curves as a function of z, waveguide A should acquire a π phase shift and waveguide B should be left invariant. However, in the intensity profile image it looks like A is left invariant and B gets a phase shift. Are the labels backwards here?

Responses:

We thank the reviewer for this careful observation. The labels of waveguides A and B in Fig. 6c are correct and are not interchanged. The apparent discrepancy arises from the fact that the simulated phase profile reflects the total accumulated phase, including both dynamical and geometric contributions, whereas the theoretical statement that waveguide A acquires a π phase shift while waveguide B remains unchanged refers specifically to the geometric phase associated with the evolution. In this process, the dynamical phase accumulated by waveguides A and B is identical, as they share the same propagation constant and propagation length. Therefore, the observed phase difference originates solely from the geometric phase. As a result, the absolute phase of each waveguide in the simulation may differ from the idealized theoretical expectation. However, the relative phase difference between the two waveguides remains π, which is the physically relevant quantity and is fully consistent with the theoretical prediction. To avoid possible confusion, we have clarified this point in the revised manuscript.

Following Reviewer 1's suggestion to streamline the overall manuscript structure and focus the main text on the most representative examples, we retained the 1D Floquet flat bands and period-$n\Lambda$ oscillations, as well as the 2D chirality reversal of topological edge states, in the main text. Accordingly, the non-Abelian transformation results originally shown in Fig. 6 have been moved to the Supplementary Note 9, where we have now clarified the relative phase difference explicitly.

Changes in the manuscript:

Following the refocusing of the manuscript structure, the non-Abelian transformation results have been moved from the main text to Supplementary Note 9

as Fig. S24. We add one sentence to the second paragraph of the section titled "**Non-Abelian Transformations Based on Polarization-Controlled Coupling**" in the supplementary note 9.

"This capability allows straightforward realization of Pauli-X, Y and Z gates. For the logical Z gate, $Z = \begin{bmatrix} 1 & 0 \\ 0 & -1 \end{bmatrix}$, *the structure consists of one straight waveguide A and one twisted waveguide B by* π *(Fig. S24a(i)). The large spacing between the two waveguides suppresses coupling. As the polarization direction of TE mode in B rotates from +x to -x, a geometric phase of* π *is accumulated, effectively introduces a -1 factor the logical state, as illustrated in Fig. S24a(ii). The experimental output fields and phases at the end facet are shown in Fig. S24a(iii). After twisting waveguide B, the electric field remains localized in waveguides A and B, while the relative phase between the two outputs is locked at* π*. The phase shown here corresponds to the total accumulated phase (dynamical + geometric). Only the relative phase difference is physically meaningful. This phase shift originates from a geometric rather than a dynamical phase, as confirmed by its invariance when the input wavelength is tuned varied from 1160* μm *to 1420* μm*."*

Comments11:

Fig 5b. What is Λ for these systems? Is 5b(i) and 5b(iii) both in a regime of Λ where there are edge states or is only 5b(iii)? Looking at 5a(iii) this would be determined if Λ < 2.5 or Λ > 2.5, so what is the value for these experiments in 5b(i) and 5b(iii)?

Responses:

We thank the reviewer for this question. In Fig. 5b, the Floquet driving period is Λ=4cm for both panels (i) and (iii). According to the phase diagram shown in Fig. 5a(iii), edge states emerge in the regime Λ >2.5cm. Therefore, both Fig. 5b(i) and Fig. 5b(iii) are in the topological regime where edge states are expected to exist. The different behaviors observed in these two panels arise from the distinct excitation conditions

rather than a change in the topological phase.

Following Reviewer 1's suggestion to streamline the overall manuscript structure and focus the main text on the most representative examples, we retained the 1D Floquet flat bands and period-$n\Lambda$ oscillations, as well as the 2D chirality reversal of topological edge states, in the main text. Accordingly, the 1D topological edge-state results originally shown in Fig. 5b have been moved to the Supplementary Note 8, where we have now clarified the value of Λ explicitly.

Changes in the manuscript:

Following the refocusing of the manuscript structure, the 1D topological edge state results have been moved from the main text to Supplementary Note 8 as Fig. S23. We add one sentence to the third paragraph of the section titled "**Topological Phase Transitions and Chirality Reversal of Edge States**" in the Supplementary Note 8.

"Theoretical evolutions of the bulk states and edge states are shown separately in Fig. S23b(i) and Fig. S23b(iii), and the experimentally measured output fields after different distances are shown in Figs. S23b(ii) and Fig. S23b(iv), respectively. Theoretical evolutions of the bulk states and edge states are shown separately in Fig. S23b(i) and Fig. S23b(iii), where the Floquet driving period is Λ=4cm for both cases. The topological edge state still propagates along the edge with periodic-driven oscillation and a negligible amount of dissipation into the bulk, whereas the bulk state rapidly delocalizes throughout the array. Similarly, in order to show the scalability of the proposed polarization-controlled strategy, additional experiments on hollow metallic waveguides are demonstrated in Supplementary Fig. S21."

We believe these revisions will fully address the reviewer's concerns and significantly improve the clarity and rigor of our manuscript. We are grateful for the insightful feedback.

References

1. Zhang, Y., Wu, R. P. H., Shi, L. & Fung, K. H. Second-Order Topological Photonic Modes in Dipolar Arrays. *ACS Photonics* **7**, 2002–2009 (2020).
2. Schulz, J., Noh, J., Benalcazar, W. A., Bahl, G. & Von Freymann, G. Photonic quadrupole topological insulator using orbital-induced synthetic flux. *Nat. Commun.* **13**, 6597 (2022).
3. Bobylev, D. A. *et al.* Topological Edge and Corner States Designed via Meta-Atoms Orientation. *Laser Photonics Rev.* **17**, 2100567 (2023).
4. Esmann, M. *et al.* Vectorial near-field coupling. *Nat. Nanotechnol.* **14**, 698–704 (2019).
5. Pelegrí, G. *et al.* Topological edge states with ultracold atoms carrying orbital angular momentum in a diamond chain. *Phys. Rev. A* **99**, 023612 (2019).
6. Pelegrí, G., Marques, A. M., Ahufinger, V., Mompart, J. & Dias, R. G. Second-order topological corner states with ultracold atoms carrying orbital angular momentum in optical lattices. *Phys. Rev. B* **100**, 205109 (2019).
7. Song, W. *et al.* Dispersionless Coupling among Optical Waveguides by Artificial Gauge Field. *Phys. Rev. Lett.* **129**, 053901 (2022).
8. Rechtsman, M. C. *et al.* Photonic Floquet topological insulators. *Nature* **496**, 196–200 (2013).
9. Cheng, Q. *et al.* Observation of Anomalous π Modes in Photonic Floquet Engineering. *Phys. Rev. Lett.* **122**, 173901 (2019).
10. Fritzsche, A. *et al.* Parity–time-symmetric photonic topological insulator. *Nat. Mater.* **23**, 377–382 (2024).
11. Maczewsky, L. J. *et al.* Nonlinearity-induced photonic topological insulator. *Science* **370**, 701–704 (2020).
12. Mukherjee, S. & Rechtsman, M. C. Observation of Floquet solitons in a topological bandgap. *Science* **368**, 856–859 (2020).
13. Chen, S. *et al.* On-Chip Photonic Localization in Aharonov–Bohm Cages Composed of Microring Lattices. *Nano Lett.* acs.nanolett.3c05095 (2024) doi:10.1021/acs.nanolett.3c05095.
14. Cheng, D. *et al.* Non-Abelian lattice gauge fields in photonic synthetic frequency dimensions. *Nature* **637**, 52–56 (2025).

Dear reviewers:

Thank you for taking the time to review our manuscript entitled “Polarization-controlled Floquet engineering in twisted photonic lattices” (Manuscript ID: NCOMMS-25-101957B).

We greatly appreciate your valuable comments and suggestions, which have been carefully considered in our revisions. Below, we have provided a point-by-point response, along with the corresponding changes made in the revised manuscript and Supplementary Information for your reference. We believe that the reviewers' comments have been carefully addressed and that the revised manuscript has been substantially strengthened.

Reply to Reviewer 1:

Comment 1:

We thank the authors for their substantial efforts in revising the manuscript. The revised version is improved in terms of structure compared to the original submission. In particular, the manuscript is now better organized, less fragmented, and more focused on its central message. Restricting the presentation to two representative examples significantly improves readability and allows the key ideas to be communicated more clearly. I appreciate that the authors have addressed my previous concerns regarding the overall structure.

Response:

We sincerely thank the Reviewer for carefully evaluating our revised manuscript and for noting that it is now better organized and more focused. This recognition is particularly encouraging to us.

Revisions in this round

We have also made detailed revisions in response to the specific points raised in this round. We have seriously considered the Reviewer's comments, particularly those concerning (1) the positioning of the conceptual advance more carefully relative to previous modal-engineering approaches; and (2) the stength and scope of the experimental validation in a more quantitative and balanced manner. In response, we have carefully revised the manuscript throughout. The principal revisions are summarized below.

A. Revisions to the presentation of novelty and results

I. Positioning relative to previous coupling-engineering approaches. We moderated the novelty claims, incorporated the studies highlighted by the Reviewer, and clarified the distinction between static coupling selection and continuous longitudinal modulation.

II. Direct comparison among TB, FDTD, and experimental results. We have added transverse TB distributions at the same propagation distances as the corresponding FDTD and experimental results, enabling direct side-by-side comparison among all three levels.

B. Revisions to the experimental evidence and its interpretation

a. Additional experiments

I. Repeatability assessment of the experimental results.

II. Experimental validation of the coupling-sign effect with newly fabricated coupling units. We newly fabricated and measured a 1D site coupling unit and a local four-site multidirectional coupling unit demonstrating the physical role of longitudinal coupling-sign reversal.

III. Extension to lower-loss platforms with material-loss characterization of alumina ceramic. We clarified the effects of material loss, the scope of the present

experimental validation, and possible extensions to lower-loss waveguide platforms.

b. Reassessment and interpretation of the experimental results

I. Interpretation and quantitative reassessment of the agreement between experiment and simulation. We have quantified the site-resolved agreement using the normalized squared Euclidean distance and distribution fidelity, repeated the dispersion-sensitive control measurement, and adopted a more cautious interpretation of the experimental results.

We believe that these revisions provide a more accurate positioning of the conceptual contribution and a more transparent, quantitatively grounded presentation of both the strengths and the remaining limitations of the experimental evidence. Detailed responses to each concern are provided below.

Comment 2:

However, in their effort to sharpen the central message of the manuscript, the authors now overplay the uniqueness of their approach, particularly the claim that the coupling coefficients can "vary directly and continuously in both magnitude and sign." I believe this statement should be moderated. Previous works have already demonstrated continuous tuning of effective coupling coefficients in both sign and magnitude (see, for example, Fig. 2 of DOI: 10.1021/acs.nanolett.4c05951 and https://doi.org/10.1063/5.0273569). While these approaches rely on modal engineering rather than polarization-based control, they nevertheless achieve similar functionality with respect to continuous control of the coupling parameter. I therefore encourage the authors to position their contribution more carefully relative to the existing literature and to include these works in Table 1 and Fig. 1(b).

Response:

We thank the reviewer for pointing out these relevant modal-engineering studies.

We agree that in static photonic systems, continuous tuning of the magnitude and sign of an effective coupling coefficient has previously been demonstrated. We have therefore moderated the relevant statements throughout the manuscript and incorporated the two studies mentioned by the Reviewer, together with other closely related works from the same research group, into the revised discussion.

At the same time, we believe that an important distinction should be clarified. In the cited studies, different coupling values are obtained by changing the modal orientation or geometrical configuration between straight waveguide dimers. Thus, although the extracted coupling coefficient varies continuously as a function of the control parameter, the coupling remains invariant along the propagation direction within each individual structure. In other words, these works demonstrate continuous tunability across a family of static configurations, rather than continuous longitudinal modulation during propagation. In our platform, by contrast, the relative polarization angle $\theta(z)$ evolves continuously along a single twisted structure. Consequently, the coupling coefficient $\kappa(z)$ continuously changes in magnitude, passes through zero, and reverses sign along the propagation direction within each Floquet period. Therefore, the former selects different static values of κ, whereas the latter enables $\kappa(z)$ to vary continuously during propagation process.

These two works are now cited as Refs. [39] and [40], discussed in the Introduction, and included in Table 1 and Fig. 1b. Because Table 1 and Fig. 1b primarily compare Floquet photonic platforms, these related studies are explicitly identified as non-Floquet platforms in Table 1 and are represented by a gray filled triangle in Fig. 1b. The revised comparison acknowledges that these approaches provide direct and continuous tuning of the coupling magnitude and sign. At the same time, it clarifies that, in the implementations reported in the cited works, the coupling is set by the static waveguide geometry and remains invariant along the propagation direction. While reading Ref. [40], we also became aware of the theoretical work now cited as Ref. [27] and the related experimental study by Cáceres-Aravena *et al*. [28]. Ref. [28] realizes stepwise

switching between positive and negative multiorbital couplings, while our scheme continuously tunes both the coupling magnitude and sign along propagation. This continuous control allows the positive and negative couplings to have unequal magnitudes. Under the driving protocol considered here, this unequal-magnitude condition gives rise to the chirality-reversed topological phase, which is absent when the positive and negative couplings have equal magnitudes. In addition, polarization-controlled sign reversal does not require deliberate S-P mode conversion, providing a distinct implementation route based on the polarization degree of freedom.

We have incorporated Table R1 into the revised main text as Table 1 and Fig. R1 as Fig. 1b.

Table R1 | Comparison of the present work with representative photonic Floquet platforms. The table compares this work with representative Floquet photonic platforms in terms of direct nearest-neighbour coupling implementation, controlled physical degree of freedom, the longitudinal modulation of $\kappa(z)$, continuous control of both coupling magnitude and sign within one Floquet period, and representative physical phenomena. Here, $\kappa(z)$ denotes the coupling coefficient between adjacent lattice sites. For the related non-Floquet studies, continuous sign tuning denotes tuning the coupling coefficient across different static modal orientations, rather than longitudinal modulation of $\kappa(z)$ within a single structure.

Floquet photonic platform	Reference	Coupling control				Physical phenomena
		Direct coupling	Controlled physical degree of freedom	Longitudinal modulation of $\kappa(z)$	Continuous magnitude-and-sign control of within one Floquet period	
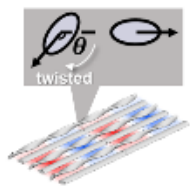 Twisted waveguide array	Our work	√	Relative polarization angle	Continuous	√	Floquet flat bands and period-$n\Lambda$ oscillations in 1D lattices; chirality reversal of topological states in 2D lattices
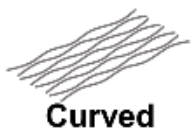 Curved waveguide array	[12]	√	Waveguide spacing	Continuous	×	Anomalous π-edge modes
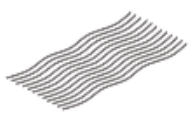 Curved waveguide array	[17]	√	Waveguide bending trajectory	×	×	Artificial gauge field-enabled dispersionless coupling
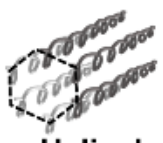 Helical waveguide array	[16]	√	Waveguide helical trajectory	×	×	Floquet topological one-way edge states
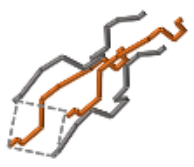 Waveguide array	[23]	√	Waveguide spacing	Stepwise	×	Parity-time-symmetric photonic topological insulator
	[24]	√	Waveguide spacing	Stepwise	×	Bulk/edge solitons
	[25]	√	Waveguide spacing	Stepwise	×	Topological Floquet solitons
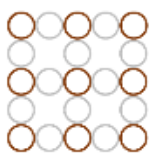 Microring resonators	[30]	×	Resonator spacing, Propagation constant detuning	Effective stepwise	×	Full k-space flat bands
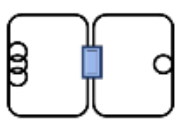 Fiber-loop lattice	[31]	×	Electro-Optic modulation	Effective stepwise	×	Non-Abelian lattice gauge fields
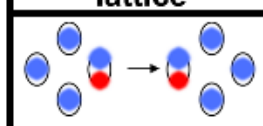 *Multi-orbital waveguide array*	*[28]*	√	*S/P orbital configuration*	*Stepwise*	*×*	*Phase-controlled chiral transport of Floquet states*
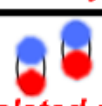 *Related non-Floquet waveguide platforms*	*[39], [40]*	√	*Relative orientation of higher-order modes*	*×*	*N.A. (static)*	*Flat bands; Aharonov–Bohm caging; topological states*

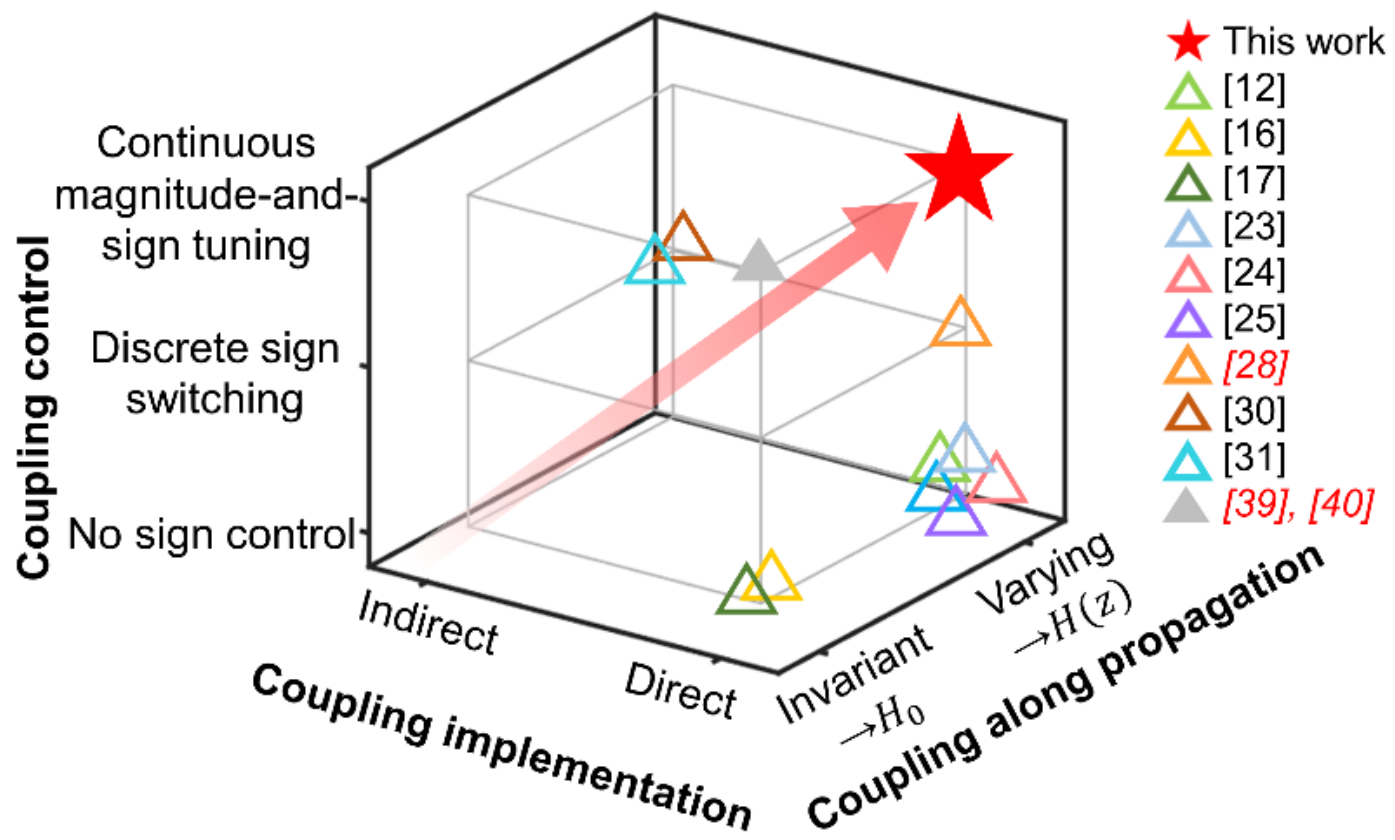

Fig. R1 | Comparison with representative Floquet photonic platforms. The solid gray triangle represents a related non-Floquet modal-engineering platform shown for comparison. The present Floquet photonic platform enables direct nearest-neighbour coupling whose magnitude and sign can be continuously tuned along the propagation direction within each Floquet period through polarization control.

To directly demonstrate the physical role of longitudinal coupling-sign reversal in our platform, we newly fabricated and measured two 1D coupling-unit samples implementing $\kappa(z) = \kappa_0 cos(\theta(z) + \theta_0)$ with $\kappa(z) = \kappa_0 |cos(\theta(z) + \theta_0)|$, respectively. These two structures have the same coupling magnitude $|\kappa(z)|$ at every propagation position but differ in whether the longitudinal sign reversal is retained. The transverse intensity distributions were experimentally measured at a propagation distance of $z = 3$ cm, as shown in Fig. R2. The clearly different output distributions provide experimental evidence that the sign variation of the coupling during propagation plays an important physical role. The experimentally measured intensities for both configurations have also been added to Fig. 2b of the revised manuscript.

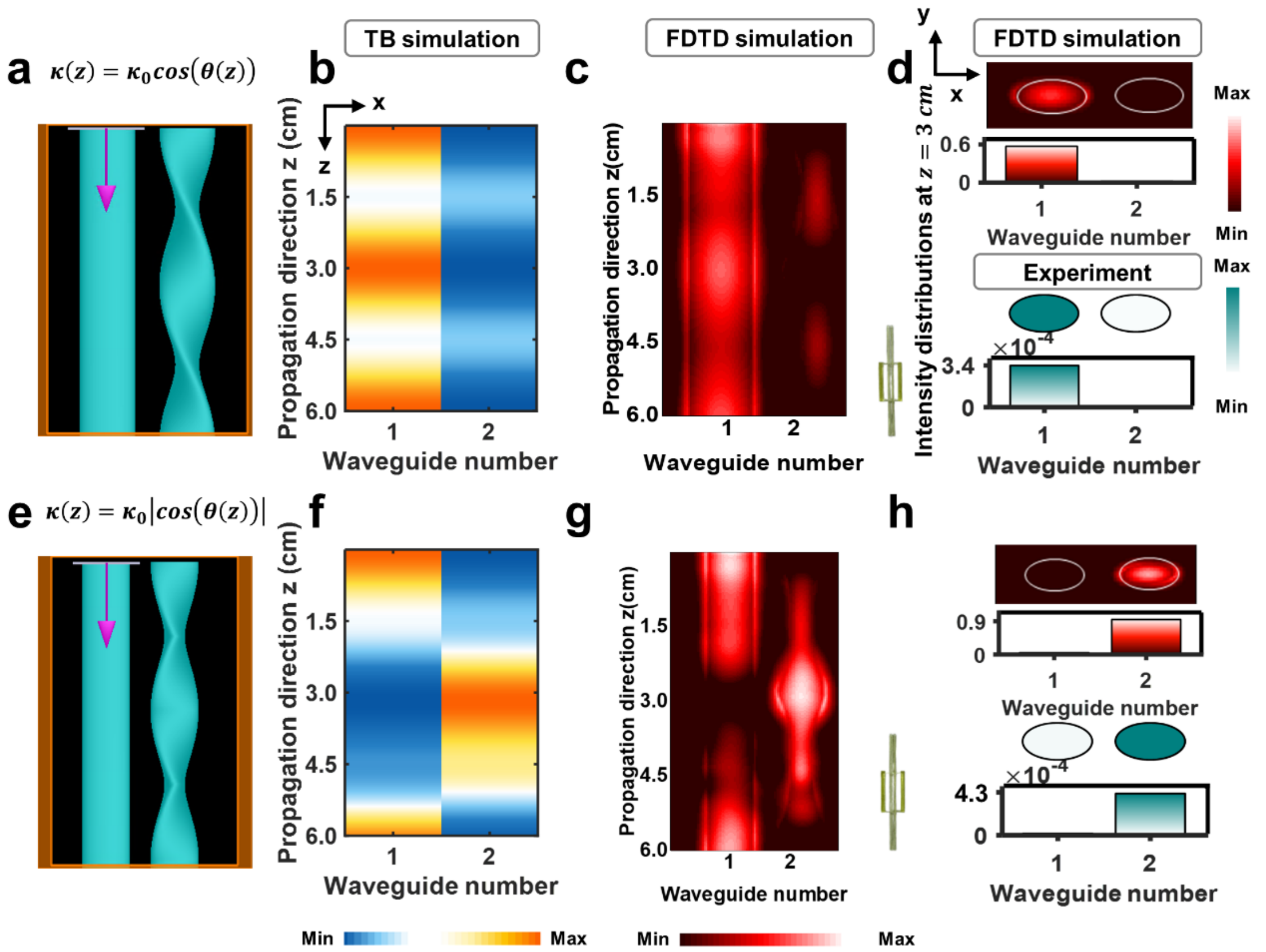

Fig. R2 | Experimental verification of the physical effect of longitudinal coupling-sign reversal in a two-waveguide unit. The two rows correspond to the longitudinal coupling functions $\kappa(z) = \kappa_0 cos(\theta(z) + \theta_0)$ (a–d), and $\kappa(z) = \kappa_0 |cos(\theta(z) + \theta_0)|$ (e–h), respectively. **a, e,** Geometries of the corresponding coupling units, consisting of one straight waveguide and one twisted waveguide. **b, f,** TB calculations of the normalized intensity evolution in the two waveguides. **c, g,** Corresponding FDTD-simulated intensity evolution. **d, h,** Transverse intensity distributions at $z = 3cm$, obtained from FDTD simulations (upper), and photographs of the fabricated samples together with the corresponding experimental measurements (lower). The cosine and absolute-cosine coupling functions have the same coupling magnitude $|\kappa(z)|$ at every propagation position, but differ in whether the coupling sign reverses. Their clearly different propagation dynamics and intensity distributions therefore provide direct evidence of the physical effect of longitudinal coupling-sign reversal.

In addition, while reviewing the reference suggested by the Reviewer (https://doi.org/10.1063/5.0273569), we noted that the approach discussed in Fig. 5 is closely related to the S-P lattice scheme included in the comparison table of representative polarization-, orientation-, and modal-engineered experimental photonic platforms provided in our previous response to Reviewer 2. We have therefore added

this reference to the comparison table.

Table R2 | Comparison between the present work and representative polarization-, orientation-, and modal engineered experimental photonic platforms.

Platform	Reference	Controlled degree of freedom	Coupling control mechanism	Key distinction from our work			Physical phenomena
				Direct mapping from polarization angle to coupling	Floquet engineering	Physical subfield	
Twisted waveguide array	Our work	Relative polarization angle	Twist controlled modal overlap variation between adjacent waveguides	√	√	Floquet dynamics; topological Floquet physics	Floquet flat bands; period-nΛ oscillations; chirality reversal of topological edge states
Dipolar coupling systems	[15], [18]	Dipole orientation and vectorial field components	Tensorial dipole-dipole interaction; vectorial near field coupling	×	×	Topological physics; vectorial near field spectroscopy	Higher order corner and hinge modes; vectorial near field resonance shifts
Mixed S-P orbital photonic lattices	[13], *[12]*	Orbital parity and relative mode orientation	P orbital parity induced synthetic π flux	×	×	Topological physics; synthetic-gauge-field physics	Corner states; *Aharonov–Bohm caging; Phase-controlled transport*
Bianisotropic meta atom lattices	[16]	Relative orientation of split ring resonators	Magneto electric hybridization	×	×	Topological physics	Topological edge and corner states
OAM optical lattices	[17], [14]	Orbital angular momentum states	OAM induced tunnelling phases and effective flux	×	×	Topological physics	Aharonov Bohm caging; topological edge and corner states

Changes in the manuscript:

Abstract:

We have revised the novelty claims and now explicitly acknowledge previous non-Floquet studies that achieved continuous control over both the magnitude and sign of the coupling coefficient.

"Further advances, however, require precise and continuous control over both the magnitude and sign of the coupling coefficient. Although static modal-engineering approaches can tune both, existing Floquet photonic platforms typically rely on geometric modulation, auxiliary structures, or phase engineering. Continuous tuning of the coupling coefficient in both magnitude and sign within a Floquet period therefore remains challenging. Here, we present a compact Floquet platform based on twisted photonic waveguide arrays, in which the twist-induced variation of the relative polarization angle between adjacent waveguides directly tunes the coupling coefficient through zero, allowing continuous access to positive and negative values with different magnitudes."

Introduction:

In the second paragraph of the Introduction, we have added the references suggested by the Reviewer and expanded the discussion of coupling-coefficient control through modal engineering.

"More broadly, internal modal degrees of freedom have enabled several nontrivial coupling mechanisms in photonic systems, including tensorial dipole-dipole coupling in dipolar arrays[36]*, orbital-parity-induced negative coupling in mixed-s/p lattices*[37]*, and orientation-controlled magneto-electric coupling in bianisotropic meta-atoms*[38]*. Related static modal-engineering studies have further demonstrated continuous tuning of the effective coupling coefficient in both magnitude and sign by varying the relative orientation of higher-order waveguide modes*[39,40]*. These studies highlight the potential of internal degrees of freedom for engineering effective coupling, synthetic flux, and topological states."*

In the first paragraph of the Introduction, we have added the following sentences:

"More recently, multi-orbital interactions have been incorporated into a Floquet protocol based on the periodic concatenation of two photonic masks, enabling phase-controlled transport of compact photonic states[27,28]*. In parallel, coupled micro-ring resonators and fiber-loop lattices have been explored to realize a variety of Floquet-*

driven photonic phenomena[29-32]. *However, coupling control in most existing photonic platforms still relies on structural modulation, auxiliary structures, phase engineering, or the concatenation of discrete photonic masks. Efficient and compact continuous tuning of the coupling coefficient in both magnitude and sign remains experimentally challenging, limiting the exploration of broader physical phenomena."*

In the final paragraph of the Introduction, we have revised the description of the novelty of our work to make it more precise.

"To place the present work in the context of existing photonic Floquet platforms, we compare it with representative previous approaches in Table 1. Earlier platforms typically realize either direct but sign-limited coupling, or control coupling through the auxiliary structures or additional modulation. By contrast, the present platform uses the relative polarization angle to realize direct and continuous longitudinal modulation of the coupling magnitude and sign within a single compact Floquet platform."

In the section "**Theoretical Model of Polarization Controlled Coupling in Twisted Waveguides**" of the **Results and Discussion**, we have added the following sentence to clarify the experimental comparison between coupling configurations with and without sign reversal along the propagation direction.

"To demonstrate the physical effect of this sign reversal, we fabricated another configuration in which the twisted waveguide undergoes successive $\frac{\pi}{2}$ *clockwise and counterclockwise twists (Fig. 2b(iii)). This configuration gives* $\kappa(z) = \kappa_0|cos\theta(z)|$, *thereby removing the sign reversal while preserving the same coupling magnitude* $|\kappa(z)|$ *at every propagation position. The upper parts of Figs. 2b(ii) and 2b(iv) show the corresponding finite-difference time-domain (FDTD) simulated intensity evolution. The intensity distributions at* $z = 3.0\ cm$ *are shown below the propagation maps, with the simulated results placed above the corresponding experimental measurements. The distinctly different light propagation dynamics observed in both the simulations and experiments provide experimental support for the polarization-controlled coupling mechanism."*

At the end of the third paragraph of the section “**Topological Phase Transitions and Chirality Reversal of Edge States**”, we have added the following sentence to clarify that access to positive and negative couplings of unequal magnitude can enable distinct physical phenomena and broaden the range of phenomena that can be explored using our platform.

“In the present driving protocol, the chirality-reversed topological phase appears only when the positive and negative coupling strengths have different magnitudes. It is absent under the equal-magnitude condition within the parameter range considered here, showing that access to positive and negative couplings of unequal magnitude is essential for realizing this transition.”

Figures:

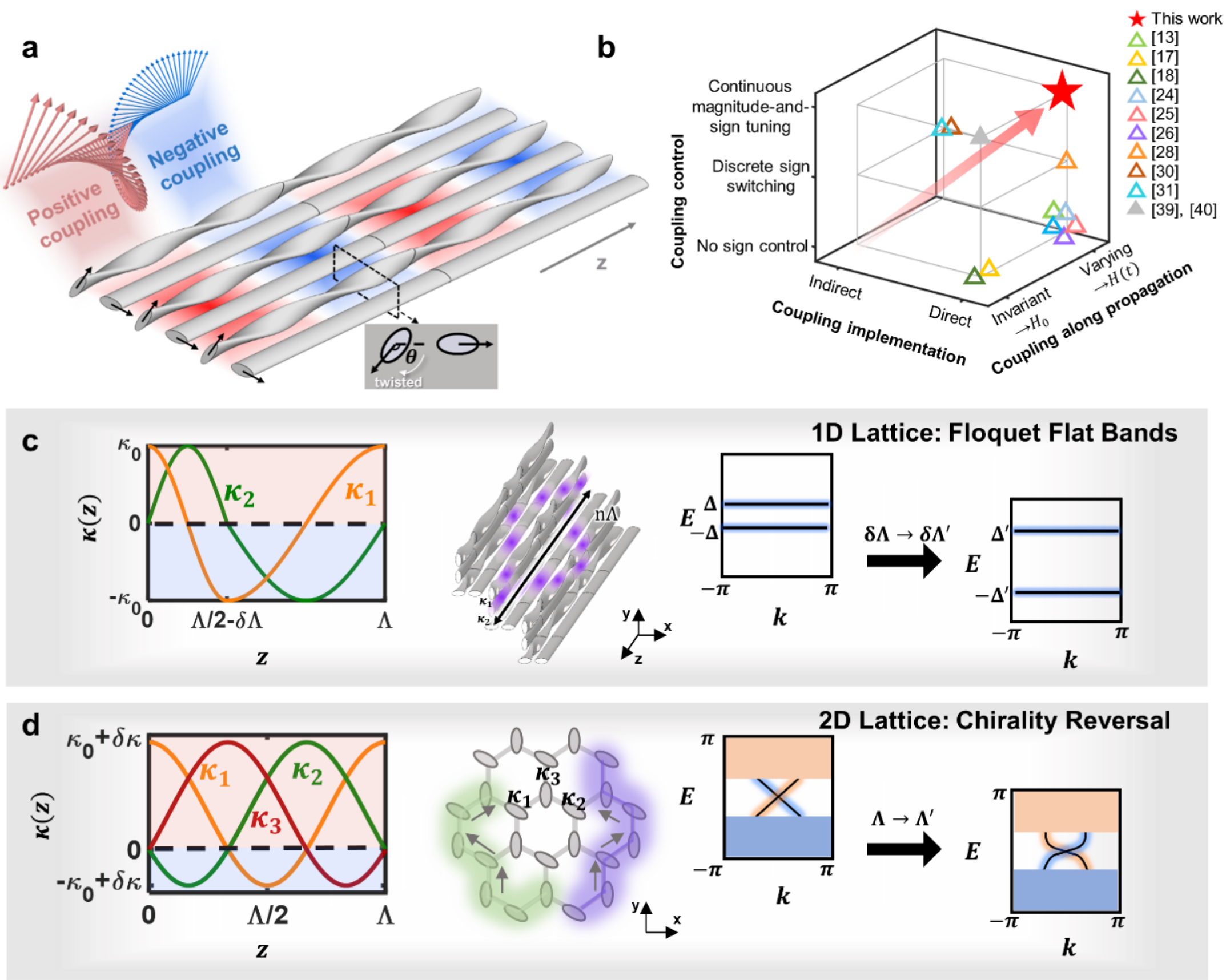

*Fig. 1 | **Polarization-controlled Floquet engineering enabled by twisted photonic lattices. a,** Concept of polarization-controlled coupling in twisted waveguide arrays. As the waveguides twist along* z*, the relative polarization angle* θ *between adjacent waveguides evolves continuously, allowing the nearest-neighbour coupling coefficient* $\kappa(z)$ *to be tuned from positive to negative*

values. Red and blue denote positive and negative coupling, respectively. ***b,*** *Comparison with representative Floquet photonic platforms. The solid gray triangle represents a related non-Floquet modal-engineering platform shown for comparison. The present Floquet photonic platform enables direct nearest-neighbour coupling whose magnitude and sign can be continuously tuned along the propagation direction within each Floquet period through polarization control.* ***c,*** *1D lattice for Floquet flat-band engineering. Left: alternating couplings* κ_1 *and* κ_2*. Middle: twisted waveguide array. Right: quasi-energy spectrum exhibits Floquet flat bands at quasi-energies* $\pm\Delta$, $\pm\Delta'$, ..., *where different flat-band quasi-energies correspond to period-*$n\Lambda$ *oscillations with different integer* n*.* ***d,*** *2D lattice for chirality reversal of topological edge states. Left: three couplings,* κ_1, κ_2, *and* κ_3*. Middle: twisted waveguide array. Right: the quasi-energy spectrum supports edge states, whose chirality is reversed when the driving period is changed from* Λ *to* Λ'*.*

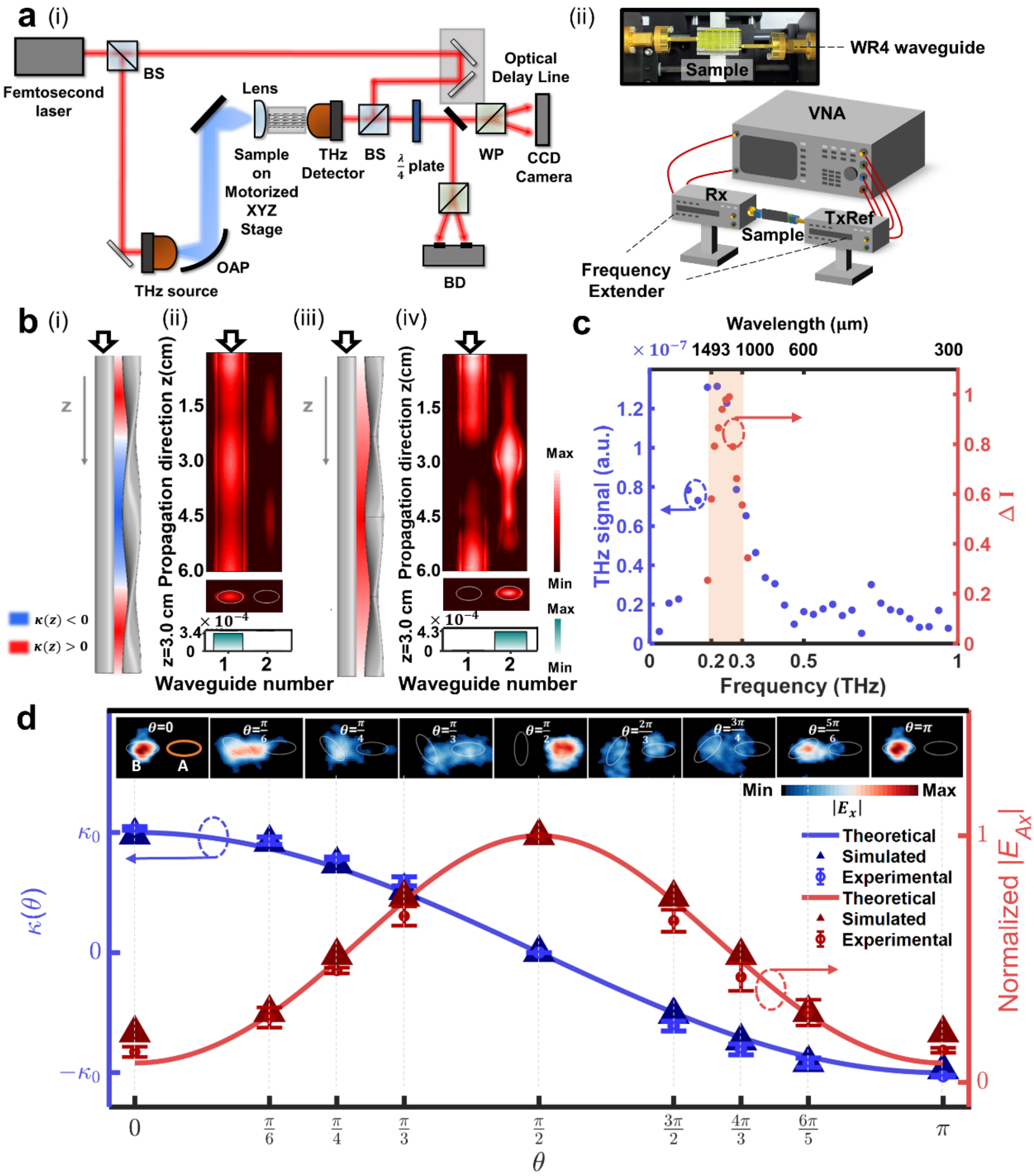

Fig. 2 | Experimental verification of polarization-controlled coupling in twisted waveguides. a, *Measurement setups.* ***(i)*** *THz-TDS setup. OAP, off-axis parabolic mirror; BS, beam splitter; BD, balance detector; WP: Wollaston prism.* ***(ii)*** *THz-VNA setup with two frequency extension modules and the sample placed between them. Inset: photograph of the setup.* ***b,*** *FDTD simulated and experimental verification of the effect of longitudinal coupling-sign reversal in two coupled waveguides.* ***(i–ii)****, one waveguide is twisted by 2π along the propagation direction, yielding $\kappa(z) = \kappa_0 cos\theta(z)$.* ***(iii–iv)****, the twisted waveguide undergoes alternating rotations of $\pm\pi/2$, yielding $\kappa(z) = \kappa_0|cos\theta(z)|$.* ***c,*** *TE mode spectrum of a single waveguide and operational bandwidth determined from the relative output intensity variation $\Delta I(\lambda)$. Operational bandwidth: 1000 μm to 1493 μm.* ***d,*** *Experimental, simulated and theoretical results for nine pairs of waveguides with different θ. Top: measured output field distribution ($|E_{Ax}|$) after 1 cm of propagation, with normalized intensity plotted as a function of θ. Orange dashed circles highlight the injected waveguides. Bottom: Extracted coupling coefficient $\kappa(\theta)$. Solid lines denote theory; points denote simulations and experiments. Error bars indicate standard deviations.*

Table:

We have incorporated Table R1 into the main text as the new **Table 1**.

References:

27. Cáceres-Aravena, G., Guzmán-Silva, D., Salinas, I. & Vicencio, R. A. Controlled Transport Based on Multiorbital Aharonov-Bohm Photonic Caging. Phys. Rev. Lett. 128, 256602 (2022).

28. Caceres-Aravena, G., Vildoso, P., Drüeke, H. & Vicencio, R. A. Phase-controlled transport of Floquet-driven compact topological photonic states. Preprint at https://doi.org/10.48550/arXiv.2607.10415 (2026).

39. Román-Cortés, D., Mazanov, M., Vicencio, R. A. & Gorlach, M. A. Observation of Invisibility Angle and Flat Band Physics in Dipolar Photonic Lattices. Nano Lett. 25, 4291–4297 (2025).

40. Vicencio, R. A. Multi-orbital photonic lattices. APL Photonics 10, 071101 (2025).

Supplementary Information:

We have incorporated Table R2 into the Supplementary Information as the new **Table S1**.

Comment 3:

I also acknowledge the authors' efforts to respond to my concerns regarding the experimental validation. The addition of FDTD simulations, independent loss measurements, and the new VNA-based characterization scheme provides useful additional information and helps clarify certain aspects of the experimental platform.

However, my main concern regarding the experimental evidence remains largely unresolved. While the revised manuscript presents a better experimental characterization through the newly introduced VNA measurement scheme, the agreement between simulations and measurements is still not sufficiently convincing. In particular, in Fig. R1, R2, and R6, the measured intensity distributions remain difficult to reconcile with the corresponding FDTD predictions. As a result, the experimental data do not provide compelling validation of the proposed coupling-engineering approach. Related to this point, I feel that the interpretation of the experimental results may be somewhat biased by an emphasis on observations that support the theoretical expectations, while the significant discrepancies receive less attention. Consequently, the revised manuscript presents the level of agreement between experiment and theory more strongly than is warranted by the data. A more cautious interpretation of the experimental results is needed here.

Response:

We sincerely appreciate the Reviewer's recognition of the additional experimental and numerical efforts, including the FDTD simulations, independent loss measurements, and VNA-based characterization. We are grateful that these additions have helped clarify the experimental platform and strengthen the presentation of the validation.

At the same time, we thank the Reviewer for raising this important concern. We agree that our previous description did not clearly explain either the degree of

agreement between experiment and simulation or the sources of the remaining differences. **To address this issue, we have therefore I. experimentally measured the representative long-distance propagation samples, II. independently calibrated a global effective refractive-index correction, III. added quantitative metrics of the agreement, IV. analyzed additional possible sources of the remaining discrepancies, and V. clarified the role and limitations of the THz-TDS system.** In addition, we have revised the relevant statements to provide a clearer and more balanced account of both the agreement and the remaining discrepancies.

For clarity, the systems shown in Figs. R6a–c of our previous-round response are now presented in the revised manuscript as Figs. 3b, 3c, and 3a, respectively. To avoid ambiguity, we use "previous Fig. 3" to refer to the figure numbering in the previous manuscript and "revised Fig. 3" to refer to the corresponding figures in the current manuscript.

I. Repeatability assessment of representative long-distance samples

To assess the repeatability of the measured output distributions, we performed five repeated measurements of two representative samples at the maximum propagation distance of $z = 4$ cm: the dispersion-sensitive straight waveguide array in previous Fig. 3a and the modulated array in previous Fig. 3b, respectively. As shown in Fig. R3, the repeated measurements reproduce the principal site-resolved distributions in both samples. The remaining variations are represented by the standard deviations across the five measurements. We retain the original experimental distributions in previous Fig. 3 and provide the repeatability analysis separately here. For the dominant output channels in both samples, the one-standard-deviation error bars are typically below approximately 10% of the corresponding mean intensities and are substantially smaller than the differences between neighboring channels. In the modulated array, for example, the intensity at waveguide 5 is approximately 5×10^{-5}, whereas most other channels remain below 1×10^{-5}, while the associated standard deviation is only on the order

of 10^{-7}. Similarly, in the straight-waveguide array, the dominant channels reach approximately 2×10^{-5}, and their variations across five repeated measurements remain much smaller than the overall channel-to-channel intensity contrast. These results support the repeatability of the measured output distributions and indicate that the main spatial features are robust against measurement fluctuations, thereby strengthening the reliability of the experimental observations.

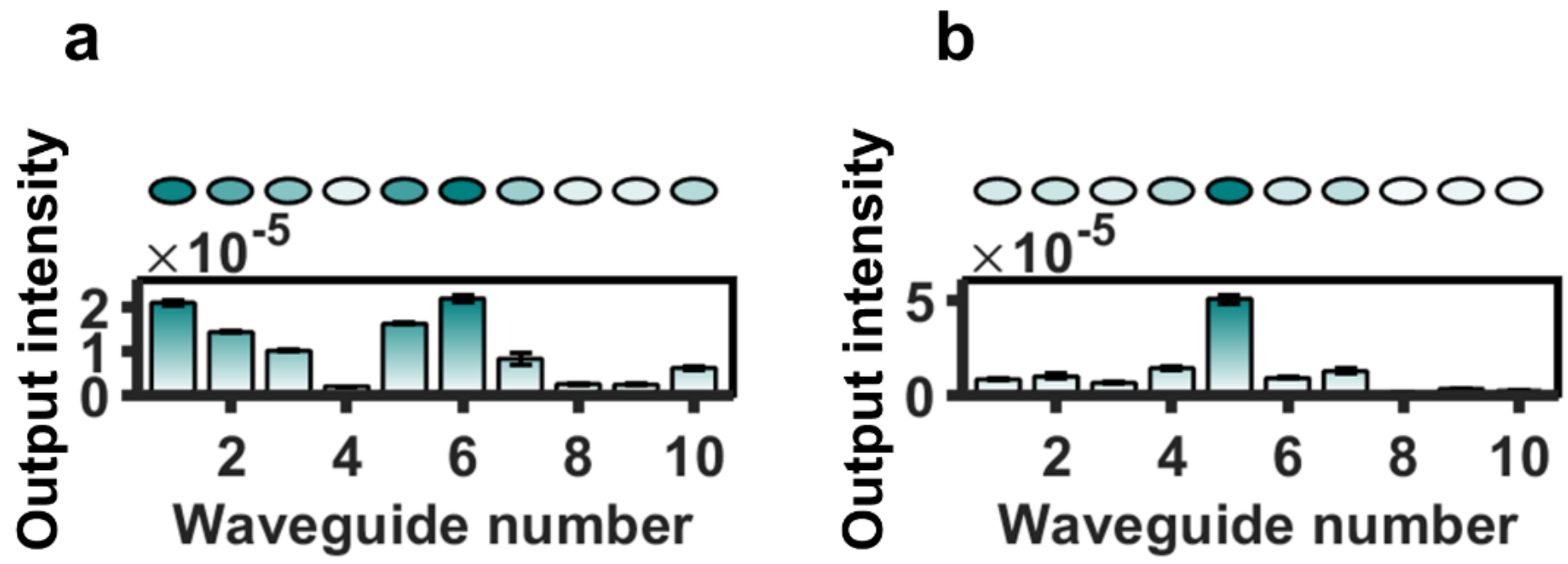

Fig. R3 | Repeatability of representative experimental output distributions at the maximum propagation distance. a, Straight waveguide array in Fig. 3a after $z = 4$ cm of propagation. **b,** modulated array in Fig. 3b after $z = 4$ cm of propagation. For each waveguide, the bar height represents the mean directly measured output intensity obtained from five repeated measurements under the same experimental conditions, and the error bar indicates one standard deviation.

II. Interpretation of the discrepancies between FDTD simulated and experimental measurements

As Figs. R1 and R2 mentioned in the previous-round comment were in the Supplementary Information primarily for qualitative comparison rather than precise quantitative analysis, the discussion here focuses mainly on the interpretation of the deviations in Fig. R6. Similar analysis also applies to all the VNA measured results retained in the revised main text.

(i) Independent calibration of the refractive index correction

Because the coupling coefficient $\kappa(\lambda)$ is wavelength dependent, even a small difference between the actual refractive index of the fabricated sample and the value

used in the simulation can accumulate over the $4\ \mathrm{cm}$ propagation distance and alter the output intensity distribution. Such a difference may arise from slight material variations introduced during the 3D-printing process. To determine the refractive-index correction independently of the site-resolved intensity distributions measured in the samples used for Figs. 3 and 4, we first used a separate $1\ \mathrm{cm}$ long straight two-waveguide coupling sample with a center-to-center separation of $d = 900\ \mu\mathrm{m}$. In the experiment, the incident wavelength was scanned from 1100 to $1300\ \mu\mathrm{m}$. At $\lambda = 1191\ \mu\mathrm{m}$, the normalized power fraction in the initially unexcited waveguide reached its maximum, corresponding to one complete coupling transfer over the $1\ \mathrm{cm}$ propagation length. We then performed FDTD simulations of the same two-waveguide structure while varying a common correction Δn to the resin refractive index and keeping all geometrical parameters fixed. A correction of $\Delta n = 0.02$ reproduced the experimentally observed transfer maximum at $\lambda = 1191\ \mu\mathrm{m}$.

We therefore adopted $\Delta n = 0.02$ as a single global effective correction to the refractive index of the printed resin. This value was subsequently kept fixed for all simulated wavelengths, propagation distances, and array configurations, while keeping the refractive index, structural geometry, and coupling parameters unchanged.

(ii) Comparison between the FDTD simulated and experimental results after the correction of refractive index

FDTD results are re-simulated for Figs. 3 and 4 using the refractive-index correction, without changing any other parameters. The resulting simulations showed improved wavelength-dependent correspondence with the measured array distributions, consistent with the conclusion obtained from the independent two-waveguide calibration. We also incorporated the effect of the global refractive-index correction into the TB simulations. As shown in Fig. R4, the recalculated FDTD results capture the main experimentally observed evolution over the wavelength range from 1200 to $1300\ \mu\mathrm{m}$ and at propagation distances from 1 to $4\ \mathrm{cm}$. The improved correspondence

is consistent with a small refractive-index mismatch being an important contributor to the original discrepancy.

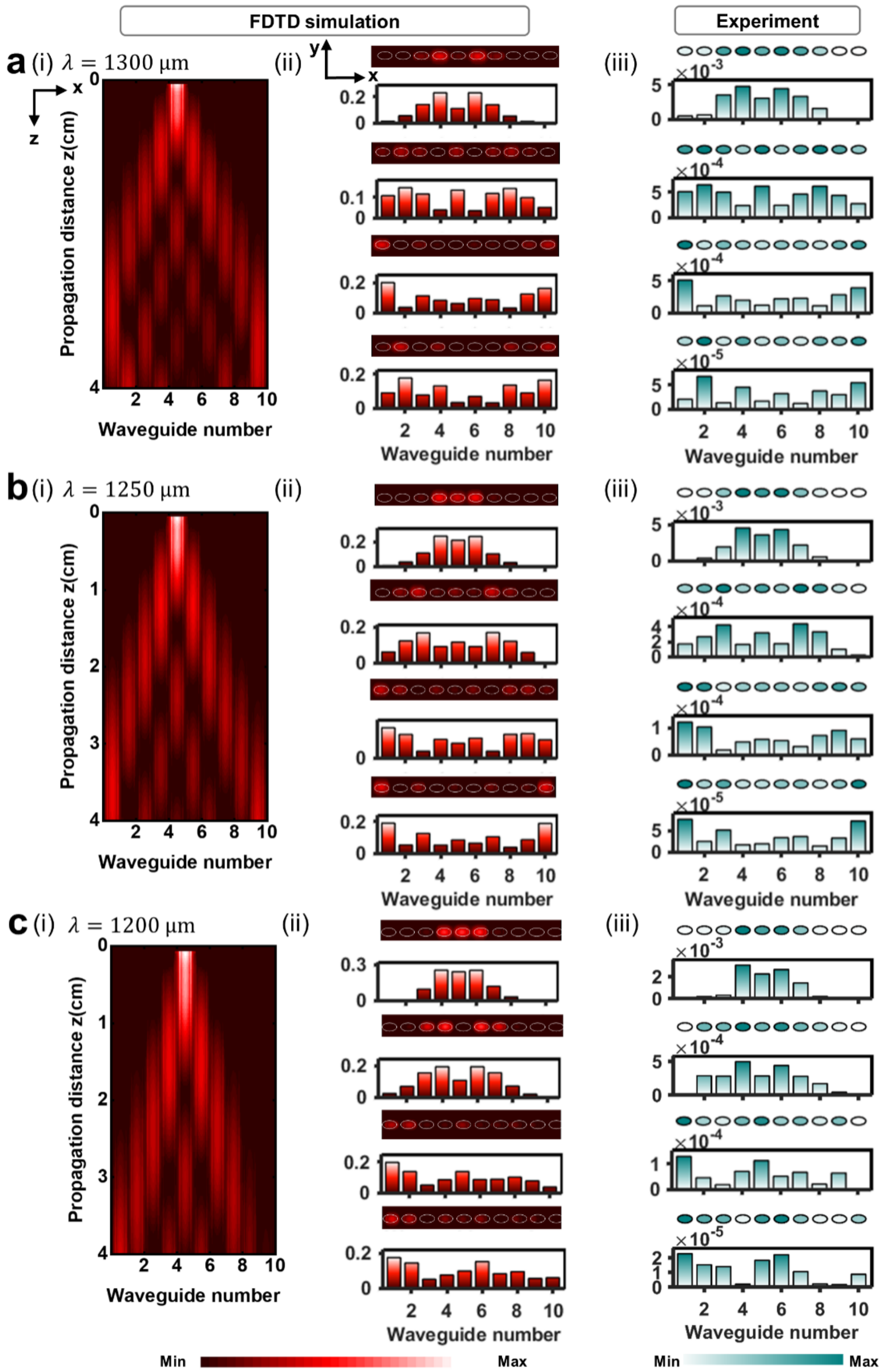

Fig. R4 | Comparison between FDTD simulations and experimental measurements at different wavelengths for the straight waveguide array after correction of the refractive index used in the simulations. a–c, Results at free-space wavelengths of $\lambda = 1300$ μm, 1250 μm, and 1200 μm, respectively. **(i)** FDTD simulated intensity evolution along the 4-cm-long waveguide

array. **(ii, iii)** FDTD simulated and experimentally measured transverse intensity distributions, respectively, at propagation distances of $z = 1, 2, 3,$ and 4 cm, shown from top to bottom. For each cross section, the colored ellipses visualize the relative intensities in the individual waveguides, while the bar plots show the corresponding waveguide intensities. A single global correction of $\Delta n = 0.02$ was first determined using an independent 1-cm-long two-waveguide coupling sample and was subsequently kept fixed for all wavelengths and propagation distances shown here.

In particular, the modulated arrays in previous Figs. 3b and 3c are less sensitive to this small refractive-index mismatch. In the ideal nearest-neighbor model, the coupling coefficient varies as $\kappa(\lambda, z) = \kappa_0(\lambda) cos(\theta(z) + \theta_0)$, and its integral over one complete driving period is zero. Therefore, a small wavelength-dependent change in the overall coupling strength $\kappa_0(\lambda)$ does not remove the spreading-and-refocusing behavior. As a result, the corrected FDTD field distributions for these modulated arrays show relatively small changes compared with the previous simulations, whereas the corresponding change is more pronounced for the straight-waveguide array control.

III. Quantitative comparison between experiment and simulation

To provide a more cautious and quantitative interpretation of the experimental results, we followed the metric employed in Ref. [Rojas-Rojas et al., Phys. Rev. A 90, 063823 (2014)], which was cited by Reviewer 1 in the first-round report, and evaluated the **normalized squared Euclidean distance** between the experimentally measured and FDTD simulated intensity distributions at each sampled propagation distance in Figs. 3 and 4. At the m-th propagation distance, let $I_{n,m}^{FDTD}$ and $I_{n,m}^{exp}$ denote the FDTD simulated and experimentally measured intensities in the n-th waveguide, respectively. The corresponding normalized intensity distributions are

$$p_{n,m}^{FDTD} = \frac{I_{n,m}^{FDTD}}{\sum_{j=1}^{N} I_{j,m}^{FDTD}}, \tag{1}$$

$$p_{n,m}^{exp} = \frac{I_{n,m}^{exp}}{\sum_{j=1}^{N} I_{j,m}^{exp}}. \tag{2}$$

At the m-th propagation distance, this distance is defined as

$$D_{NSE}(z_m) = \frac{1}{2}\frac{\sum_{n=1}^{N}\left[(p_{n,m}^{FDTD} - \bar{p}_m^{FDTD}) - (p_{n,m}^{exp} - \bar{p}_m^{exp})\right]^2}{\sum_{n=1}^{N}(p_{n,m}^{FDTD} - \bar{p}_m^{FDTD})^2 + \sum_{n=1}^{N}(p_{n,m}^{exp} - \bar{p}_m^{exp})^2}, \tag{3}$$

where N is the number of waveguides and $\bar{p}_m$ denotes the mean normalized intensity across all waveguides at z_m. Because both distributions are normalized to unit total intensity and contain the same number of waveguides,

$$\bar{p}_m^{FDTD} = \bar{p}_m^{exp} = \frac{1}{N}. \tag{4}$$

A value of $D_{NSE} = 0$ indicates a perfect match, whereas values approaching $D_{NSE} = 1$ represent the maximum possible discrepancy. Thus, smaller values indicate better agreement between the experiment and the FDTD simulation.

As a complementary measure of spatial overlap, we also calculated the **classical distribution fidelity**. The distribution fidelity is then defined as

$$F_m = \left(\sum_{n=1}^{N}\sqrt{p_{n,m}^{FDTD} p_{n,m}^{exp}}\right)^2. \tag{5}$$

This quantity satisfies

$$0 \le F_m \le 1, \tag{6}$$

with values closer to unity indicating greater agreement between the experimental and FDTD simulated spatial intensity distributions.

For M experimentally sampled propagation distances, the overall mean D_{NSE} and F is defined as

$$\overline{D_{NSE}} = \frac{1}{M}\sum_{m=1}^{M} D_{NSE}(z_m), \bar{F} = \frac{1}{M}\sum_{m=1}^{M} F_m. \tag{7}$$

We calculated both the normalized squared Euclidean distance and the classical distribution fidelity at every measured propagation distance in revised Figs. 3a–c, and Supplementary Figs. S28a, b, as well as for previous-round Fig. R6c (listed as "Previous Fig. 3a" in Table R3). All calculated values are summarized in Table R3. After incorporating a single globally corrected refractive-index value into the FDTD simulations, the revised straight waveguide results in Fig. 3a show a modest

improvement in the quantitative agreement between experiment and simulation compared with the previous results. These metrics quantify the relative site-resolved intensity distributions after normalization to unit total intensity and do not assess the absolute transmitted power or propagation loss.

Table R3 | Quantitative comparison between the FDTD-simulated and experimentally measured intensity distributions for the straight-waveguide control sample. The normalized squared Euclidean distance D_{NSE} and the distribution fidelity F are calculated at each sampled propagation distance z for Figs. 3a and for the straight-waveguide control sample at incident wavelengths of 1300, and 1250 μm. The results at 1200 μm correspond to Fig. 3a in the previous-round response and are included here for comparison.

	Previous Fig. 3a: control sample at $\lambda = 1200$ μm				Fig. 3a: control sample at $\lambda = 1200$ μm				Fig. S28a: control sample at $\lambda = 1300$ μm				Fig. S28b: control sample at $\lambda = 1250$ μm			
Propagation distance z (cm)	1	2	3	4	1	2	3	4	1	2	3	4	1	2	3	4
D_{NSE}	0.050	0.134	0.257	0.266	0.019	0.035	0.071	0.064	0.014	0.019	0.006	0.043	0.002	0.021	0.049	0.019
$\overline{D_{NSE}}$	0.177				0.047				0.021				0.023			
F	0.914	0.926	0.837	0.772	0.956	0.962	0.92	0.932	0.972	0.966	0.951	0.946	0.963	0.961	0.941	0.932
$\bar{F}$	0.862				0.943				0.958				0.949			

For the straight-waveguide control sample at 1200 μm, the mean $\overline{D_{NSE}}$ decreases from 0.177 in the previous comparison to 0.047 after applying the global refractive-index correction, while the mean fidelity $\bar{F}$ increases from 0.862 to 0.943. With the same correction kept fixed, the mean $\overline{D_{NSE}}$ values at 1250 and 1300 μm are 0.023 and 0.021, respectively, with corresponding mean fidelities of 0.949 and 0.958. These quantitative results show that the global refractive-index correction improves the overall agreement between the FDTD simulations and the experimental measurements.

We also calculated the same quantitative metrics for Figs. 3b and 3c and Figs. 4c and 4d. The corresponding results are summarized in Table R4 for Figs. 3b and 3c and in Table R5 for Figs. 4c and 4d.

Table R4 | Quantitative comparison between the FDTD-simulated and experimentally measured intensity distributions in Figs. 3a, 3b and 3c. The normalized squared Euclidean distance D_{NSE} and the distribution fidelity F are calculated at each experimentally sampled propagation distance. The row below the individual D_{NSE} and F values gives their mean values over all sampled propagation distances.

	Fig. 3a				Fig. 3b				Fig. 3c			
Propagation distance z (cm)	1	2	3	4	1	2	3	4	1	2	3	4
D_{NSE}	0.019	0.035	0.071	0.064	0.022	0.069	0.19	0.038	0.004	0.013	0.032	0.045
$\overline{D_{NSE}}$	0.047				0.081				0.023			
F	0.956	0.962	0.92	0.932	0.970	0.936	0.913	0.933	0.969	0.901	0.947	0.922
$\overline{F}$	0.943				0.938				0.935			

For Figs. 3a, 3b and 3c, the mean $\overline{F}$ across the sampled propagation distances are 0.943, 0.938 and 0.935, respectively, while the corresponding mean $\overline{D_{NSE}}$ values are 0.047, 0.081 and 0.023

Table R5 | Quantitative comparison between the FDTD-simulated and experimentally measured intensity distributions in Figs. 4c and 4d. The normalized squared Euclidean distance D_{NSE} and the distribution fidelity F are calculated at each experimentally sampled propagation distance. The row below the individual D_{NSE} and F values gives their mean values over all sampled propagation distances.

	Fig4c: period-Λ oscillation				Fig4d: : period-2Λ oscillation			
Propagation distance z (cm)	1	2	3	4	1	2	3	4
D_{NSE}	0.042	0.120	0.153	0.060	0.049	0.067	0.030	0.187
$\overline{D_{NSE}}$	0.094				0.083			
F	0.913144	0.884768	0.888581	0.925183	0.9138	0.875805	0.950652	0.877337
$\overline{F}$	0.903				0.905			

For Figs. 4c and 4d, the mean $\overline{F}$ across the sampled propagation distances are 0.903 and 0.905, respectively, while the corresponding mean $\overline{D_{NSE}}$ values are 0.094 and 0.083. These values indicate that the principal spatial features are reproduced, although the quantitative agreement is lower than that obtained for the straight-waveguide control. For the Floquet-engineered samples, we provide a further analysis of the possible origins of the remaining discrepancies in Section IV.

The same metrics were also applied to the newly fabricated 1D and four-site coupling-unit samples, as summarized in Table R6 in our response to Comment 5.

We have also replaced overly strong descriptions such as "excellent agreement" with more careful wording and explicitly discuss the remaining differences between experiment and simulation.

IV. Additional possible sources of the remaining discrepancies

(i) Measurement uncertainty associated with probe positioning

Both the excitation and output detection were implemented using WR-4 metallic hollow waveguides connected to the VNA. During the measurements, three-axis translation stages were used to scan the output probe precisely within the same plane, and repeated measurements were averaged to improve reproducibility. Nevertheless, small variations in the probe-to-sample distance and lateral alignment could not be completely eliminated. For the samples in Figs. 3b, 3c, 4c, and 4d, the electric-field polarization varies among the different output waveguides. The frequency multiplier and WR-4 metallic hollow waveguide therefore need to be rotated accordingly during the measurements, which can make the measurements more sensitive to probe-positioning errors and thereby contribute to the remaining discrepancies. Separate alignment tests showed that retracting the receiving WR-4 waveguide by 0.5 mm from the output facet reduced the detected intensity by approximately 0.8 dB, corresponding to a decrease of about 16.8%. A lateral offset of approximately 0.5 mm between the centers of the metallic and dielectric waveguides produced a reduction of about 0.5 dB, corresponding to a decrease of approximately 10.9%. These positioning uncertainties may therefore contribute to the remaining point-by-point differences between the measured and simulated intensity distributions. To reduce the instability associated with probe positioning, each measurement was repeated multiple times and the results were averaged.

(ii) Input-mode purity

In the simulations, the dielectric waveguide can be excited with an ideal TE eigenmode. In the experiment, however, the field from the WR-4 metallic waveguide is coupled through the input facet and projects onto the guided modes of the dielectric waveguide. Owing to finite mode mismatch, a small non-TE component may therefore be excited. Such a contribution is not expected to alter the overall propagation behavior, but may contribute to the remaining differences in the detailed site-resolved intensity

distributions.

V. Results validity clarification obtained from THz-TDS system

Figs. R1 and R2 in our previous response were measured using the THz-TDS imaging system. In our first-round response, Fig. R10 showed that the transmitted signal from the 4-cm-long sample remained approximately one order of magnitude above the background noise. We reproduce this comparison here as Fig. R5 for completeness. However, although this comparison confirms that the transmitted signal is clearly distinguishable from the background noise, the limited signal-to-noise ratio of the detection system reduces the reliability of a point-by-point quantitative comparison of the measured spatial intensity distribution. In addition, the samples measured using the THz-TDS setup incorporated horn-shaped input tapers to improve coupling from the incident source into the dielectric waveguides. These tapers could introduce slight distortions of the injected mode profile and thereby affect the subsequent propagation dynamics. We have therefore **retained all THz-TDS results only as qualitative evidence in the Supplementary Information**.

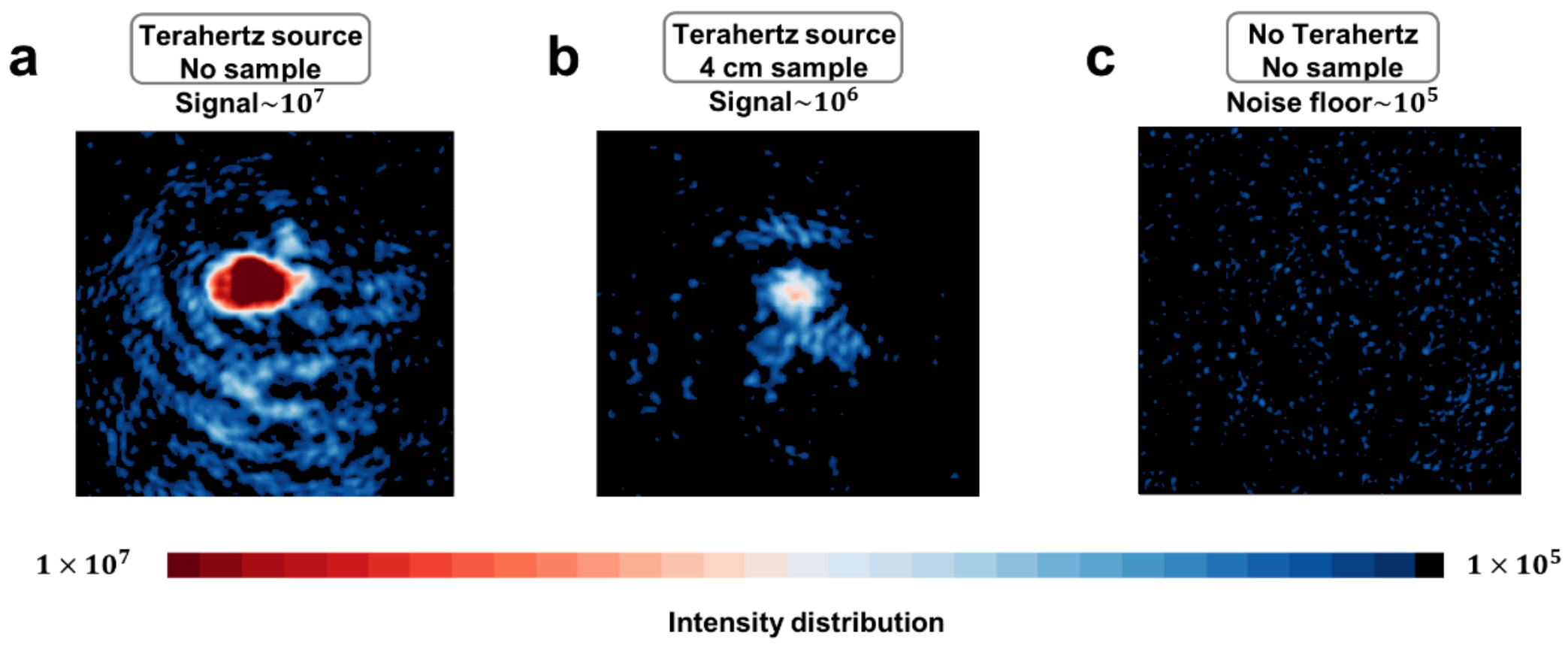

Fig. R5 | Comparison between the experimental transmitted terahertz signal from the longest sample and the noise floor in the THz-TDS system, reproduced from Fig. R10 of our first-round response.

Changes in the manuscript:

Main text:

We have replaced overly strong descriptions such as "excellent agreement" with more careful wording and explicitly discuss the remaining differences between experiment and simulation.

We have added these sentences to the first paragraph of the section entitled "**Theoretical Model of Polarization-Controlled Coupling in Twisted Waveguides**".

"The measured x-polarized output fields after 1 cm of propagation reproduce the overall dependence on the relative polarization angle predicted by theory and simulation (Fig. 2d). In the theoretical model, only the coupling between TE modes polarized along the major axis of the elliptical waveguides is considered. In the practical simulation and experimental systems, however, although the degeneracy between the TE and TM modes is lifted, weak residual intermodal coupling may still occur. Together with fabrication imperfections and slight deviations in waveguide geometry, this can lead to a small but non-zero signal near $\theta = 0$ and $\theta = \pi$, where the idealized theory predicts vanishing transmission."

We have deleted the following sentence.

"~~Importantly, these small deviations do not affect the overall agreement between theory, simulation, and experiment, nor the main physical conclusions of the work.~~"

We have added these sentences to the third paragraph of the section entitled "**Floquet Flat Bands and Period-nΛ Oscillations**".

"The quantitative comparison yields mean distribution fidelities $\bar{F}$ of above 0.935, with the corresponding mean $\overline{D_{NSE}}$ values below 0.081, as detailed in Supplementary Table 2. The measured distributions show slight deviations in the relative site intensities, particularly at intermediate and long propagation distances. The coupling coefficient is wavelength dependent, so a small deviation in the resin refractive index can accumulate over the propagation length and substantially redistribute the output intensity among neighboring sites. Additional differences may arise from output alignment and the idealized input mode used in the FDTD model. These effects can contribute to the remaining site-resolved differences."

Methods:

*"**Quantitative comparison of simulated and experimental intensity distributions:***

To quantify the agreement between the experimentally measured and FDTD-simulated output distributions in Figs. 3 and 4, the site-resolved intensities at each sampled propagation distance z are normalized to unit total intensity:

$$p_{n,m}^{FDTD} = \frac{I_{n,m}^{FDTD}}{\sum_{j=1}^{N} I_{j,m}^{FDTD}}, \qquad p_{n,m}^{exp} = \frac{I_{n,m}^{exp}}{\sum_{j=1}^{N} I_{j,m}^{exp}}, \tag{8}$$

where N is the number of waveguides. We evaluated the normalized squared Euclidean distance[49]

$$D_{NSE}(z_m) = \frac{1}{2}\frac{\sum_{n=1}^{N}\left[\left(p_{n,m}^{FDTD} - \bar{p}_m^{FDTD}\right) - \left(p_{n,m}^{exp} - \bar{p}_m^{exp}\right)\right]^2}{\sum_{n=1}^{N}\left(p_{n,m}^{FDTD} - \bar{p}_m^{FDTD}\right)^2 + \sum_{n=1}^{N}\left(p_{n,m}^{exp} - \bar{p}_m^{exp}\right)^2}. \tag{9}$$

The metric satisfies $0 \leq D_{NSE} \leq 1$, with smaller values indicating closer agreement. As a complementary measure of spatial overlap, we calculated the classical distribution fidelity

$$F_m = \left(\sum_{n=1}^{N} \sqrt{p_{n,m}^{FDTD} p_{n,m}^{exp}}\right)^2, \tag{10}$$

where $0 \leq F_m \leq 1$, and values closer to unity indicate greater overlap. For M sampled propagation distances, the mean fidelity was calculated as

$$\bar{F} = \frac{1}{M}\sum_{m=1}^{M} F_m. \tag{11}$$

These metrics quantify the relative site-resolved intensity distributions after normalization to unit total intensity and do not assess the absolute transmitted power or propagation loss."

Figures:

We recalculated the FDTD results using the single global refractive-index correction of $\Delta n = 0.02$. During this re-examination, we identified a plotting error in the experimental result at $z = 3$ cm in the previous Fig. 3a(v). The measured

intensities should have been plotted from left to right according to waveguide numbers 1-10. However, this dataset was inadvertently displayed in the reverse order, 10-1. We have corrected the plotting order in the revised Fig. 3a(v). This correction only reverses the spatial arrangement of the same measured intensity values and does not alter the raw experimental data.

The revised FDTD results show a modest improvement in the agreement with the experimental measurements compared with previous Fig. R6. Nevertheless, residual site-resolved discrepancies remain, and these are now explicitly acknowledged and discussed in the revised manuscript.

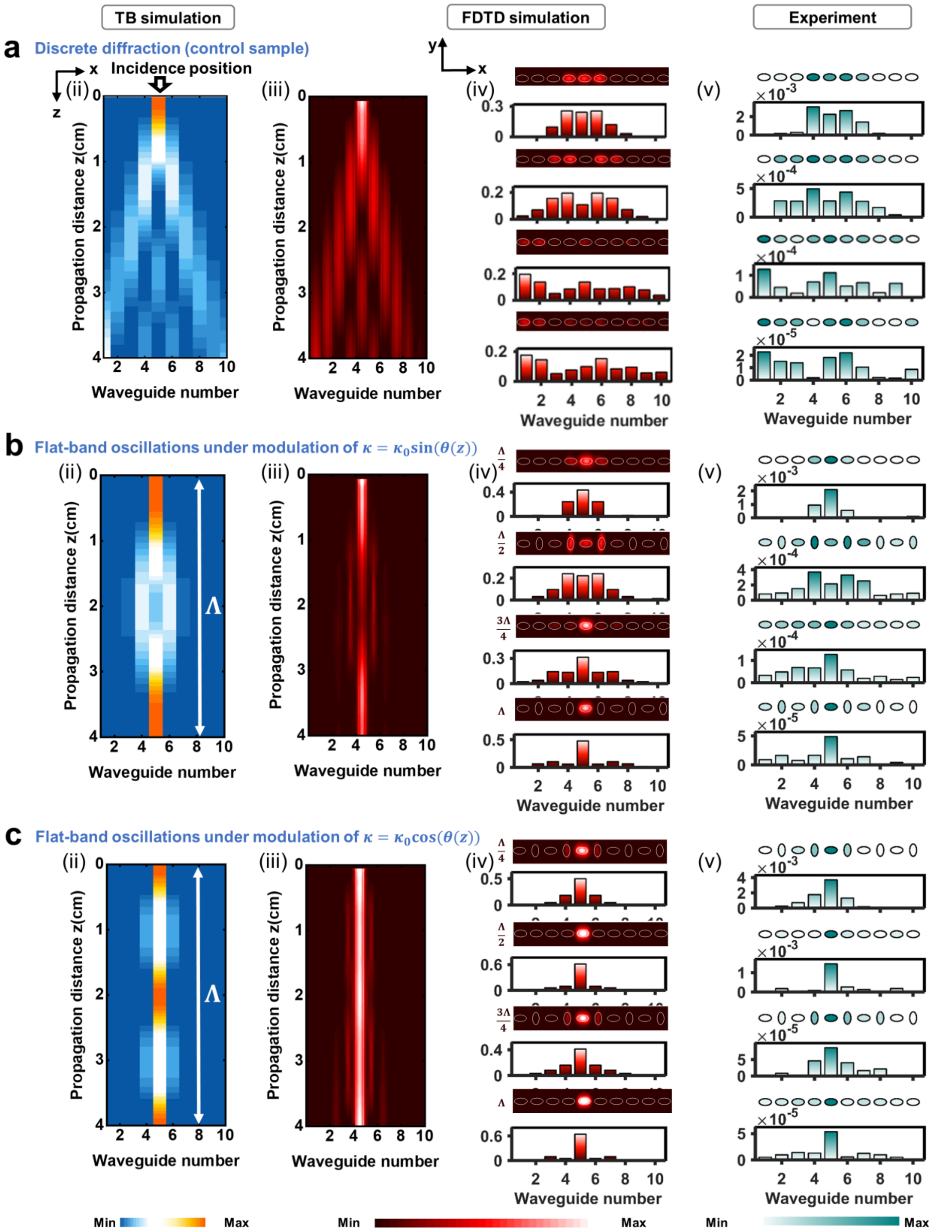

*Fig. 3 | **b,** Floquet flat-band dynamics under $\kappa = \kappa_0 sin(\theta(z))$. **c,** Floquet flat-band dynamics under $\kappa = \kappa_0 cos(\theta(z))$. For each case, panel **(ii)** and **(iii)** show TB and FDTD simulations; and **(iv)** and **(v)** show FDTD and experimental output intensity distributions at selected propagation distances $\frac{\Lambda}{4}$, $\frac{\Lambda}{2}$, $\frac{3\Lambda}{4}$, and Λ. Light is injected into the central waveguide. The FDTD results use a common normalization scale, whereas the experimental bar plots show directly measured, unnormalized intensities.*

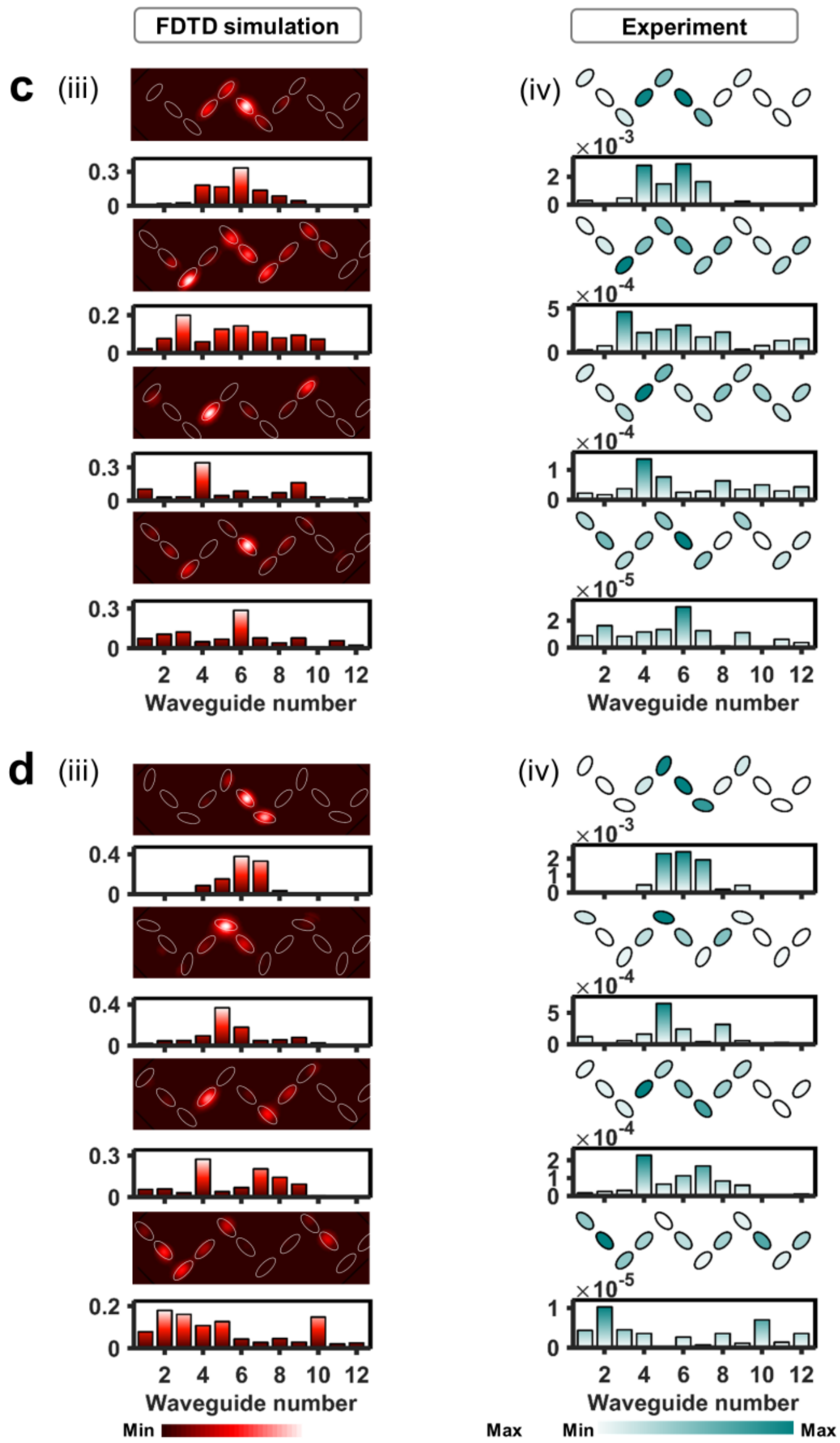

*Fig. 4 | Period-$n\Lambda$ oscillations. c, d, Experimental results of period-$n\Lambda$ oscillations for $n = 1$ and $n = 2$, respectively. Transverse intensity distributions are shown for the FDTD simulations in **(iii)** and the experiments in **(iv)**. The FDTD results are normalized to a common scale across different propagation distances, whereas the experimental bar plots show directly measured, unnormalized output intensities.*

References:

50. Rojas-Rojas, S. et al. Analytical model for polarization-dependent light propagation in waveguide arrays and applications. Phys. Rev. A ***90****, 063823 (2014).*

Supplementary Information:

We have added Fig. R4 to Supplementary Fig. S28.

We have also added Table R3, R4 and R5 to Supplementary Table S2.

Comment 4:

For completeness and ease of comparison, I would also recommend including cross-sectional plots of the tight-binding simulations analogous to those shown for the FDTD simulations and measurements in Fig. 4. Such plots would facilitate a more direct assessment of the correspondence between the different modeling levels and the experimental data.

Response:

We thank the Reviewer for this helpful suggestion. In the revised Fig. 4, we have added TB transverse intensity distributions at the same representative propagation distances as those used for the corresponding FDTD simulations and experimental measurements. The results are presented both on schematic elliptical-waveguide arrays and as normalized waveguide-resolved bar plots in the new second column, enabling direct side-by-side comparison among the TB model, FDTD simulations, and experiment.

In preparing this comparison, we also revisited the TB calculation to account for experimentally relevant effects that are absent from the idealized model. In the VNA measurements, removal of the input taper produced a small forward displacement of the dielectric input facet, thereby changing the effective excitation position. Because the two couplings adjacent to the excited waveguide in Fig. 4 have different $\sin(\theta(z))$-like and $\cos(\theta(z))$-like longitudinal dependences, the dynamics are sensitive to this effective excitation position (Supplementary Fig. S35). We therefore use the experimentally relevant starting position in the revised TB calculation, without changing the coupling parameters. The corrections modify the detailed site-resolved intensities, while the recalculated dynamics retain the characteristic period-Λ and period-2Λ behavior. Separately, we considered whether residual TE-TM coupling at the smaller adjacent-waveguide separations in Fig. 4 than in Fig. 3 could contribute to the remaining site-resolved differences. For this purpose, we introduced an extended two-polarization TB model in Supplementary Note 21. This model is used solely as a

physically motivated sensitivity analysis to assess the possible influence of residual TE–TM coupling on the propagation dynamics; it is neither used to fit the experimental intensity distributions nor intended to provide an exact quantitative description of the full polarization dynamics.

Overall, the TB, FDTD, and experimental results show qualitative consistency, although some site-resolved differences remain. We have incorporated the corresponding discussion of these discrepancies into the revised manuscript.

Changes in the manuscript:

Figures:

Fig. 4 has been revised by adding TB transverse intensity distributions and normalized waveguide-resolved bar plots at the same propagation distances as the corresponding FDTD simulations and experimental measurements.

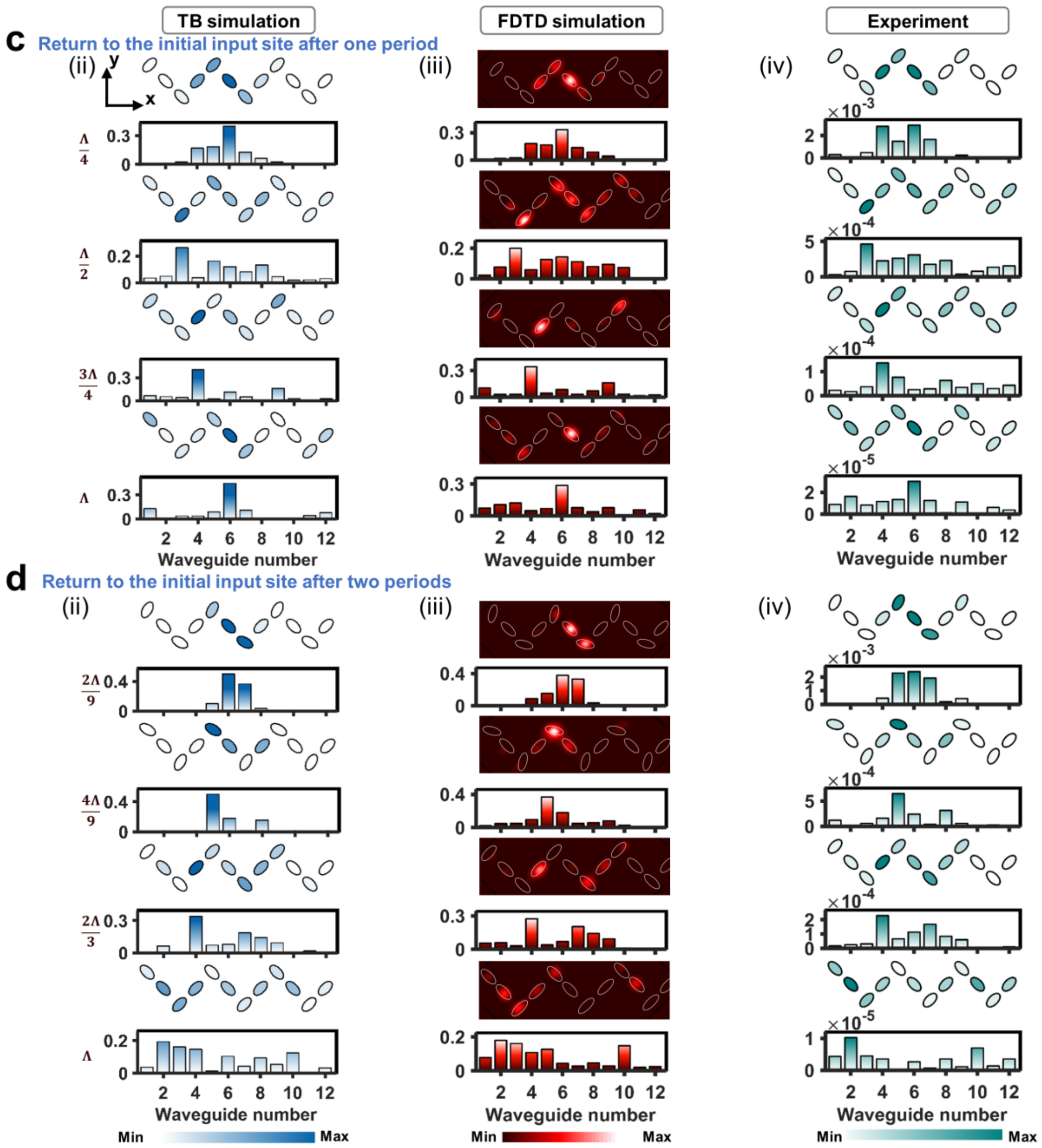

*Fig. 4 | c, d, Experimental results of period-nΛ oscillations for n = 1 and n = 2, respectively. Transverse intensity distributions are shown for the tight-binding simulations in **(ii)**, the FDTD simulations in **(iii)** and the experiments in **(iv)**. The FDTD results are normalized to a common scale across different propagation distances, whereas the experimental bar plots show directly measured, unnormalized output intensities.*

Main text:

We have added these sentences to the first paragraph of the section entitled "**Floquet Flat Bands and Period-nΛ Oscillations**".

"The TB, FDTD, and experimental results show the same overall propagation behavior, while the remaining site-resolved differences may partly arise from the

sensitivity to the effective excitation position and possible residual TE-TM coupling at the smaller waveguide separations (Supplementary Fig. S35 and Note 21).”

Supplementary Information:

*“**Supplementary Note 21 | Possible influence of residual TE–TM coupling***

When the waveguide separation is reduced to 850 or 900 μm, as used in Fig. 4, the increased modal overlap can give rise to weak cross-polarization coupling between the TE and TM modes of adjacent elliptical waveguides. To examine the possible influence of this effect on the propagation dynamics, we extend the TB basis from a single TE mode to both TE and TM modes in each waveguide. This extension is intended as a physically motivated sensitivity analysis of residual TE-TM coupling rather than an exact quantitative description of the full polarization dynamics. Once a finite TM component is excited through inter-waveguide TE-TM coupling, coupling between the TM modes of neighboring waveguides can also contribute to the subsequent evolution. In the parameter range considered here, these additional channels mainly modify the detailed site-resolved intensity distribution while preserving the characteristic propagation behavior of the TE-dominated model. Their influence can be further reduced by optimizing the waveguide geometry, for example by increasing the waveguide separation or adjusting the aspect ratio of the elliptical cross section to suppress TE-TM modal overlap.

The extended model retains the different propagation constants of the two polarizations and includes both same-polarization and cross-polarization nearest-neighbour couplings. With real coupling coefficients, the resulting Hamiltonian is Hermitian and therefore conserves the total modal power during propagation. The present Hamiltonian includes inter-waveguide TE-TM coupling but no on-site TE-TM conversion within an individual twisted waveguide. Such intra-waveguide conversion is sufficiently weak to be neglected under the parameters considered here (a semi-major axis of $a = 350\,\mu m$ and a semi-minor axis of $b = 150\,\mu m$), as shown in Supplementary Fig. S36.

For two adjacent waveguides connected by a bond $b \in \{u, v\}$, the previous TE-only model can be written as

$$i\frac{d}{dz}\begin{pmatrix} a_{1,TE} \\ a_{2,TE} \end{pmatrix} = H_{TE}^{(b)}(z)\begin{pmatrix} a_{1,TE} \\ a_{2,TE} \end{pmatrix}, \qquad H_{TE}^{(b)}(z) = \begin{pmatrix} \beta_{TE} & \kappa_{TE}^{(b)}(z) \\ \kappa_{TE}^{(b)}(z) & \beta_{TE} \end{pmatrix}. \tag{44}$$

Defining $\theta(z) = 2\pi z/\Lambda$, the two alternating nearest-neighbour bonds are described by

$$\kappa_{TE}^{(u)}(z) = \kappa_{TE,0} cos\theta(z), \qquad \kappa_{TE}^{(v)}(z) = \kappa_{TE,0} sin\theta(z). \tag{45}$$

In the extended model, we use the basis

$$\Psi = (a_{1,TE}, a_{2,TE}, a_{1,TM}, a_{2,TM})^T, \tag{46}$$

and the Hamiltonian for either bond takes the form

$$H^{(b)}(z) = \begin{pmatrix} \beta_{TE} & \kappa_{TE}^{(b)}(z) & 0 & \kappa_{TE-TM}^{(b)}(z) \\ \kappa_{TE}^{(b)}(z) & \beta_{TE} & \kappa_{TE-TM}^{(b)}(z) & 0 \\ 0 & \kappa_{TE-TM}^{(b)}(z) & \beta_{TM} & \kappa_{TM}^{(b)}(z) \\ \kappa_{TE-TM}^{(b)}(z) & 0 & \kappa_{TM}^{(b)}(z) & \beta_{TM} \end{pmatrix}. \tag{47}$$

Here, β_{TE} and β_{TM} are the propagation constants of the two guided polarizations. The longitudinally modulated coupling coefficients are chosen as

$$\kappa_{TE}^{(u)}(z) = \kappa_{TE,0} cos(\theta(z)), \qquad \kappa_{TE}^{(v)}(z) = \kappa_{TE,0} sin(\theta(z)).$$
$$\kappa_{TM}^{(u)}(z) = \kappa_{TM,0} cos(\theta(z)), \qquad \kappa_{TM}^{(v)}(z) = \kappa_{TM,0} sin(\theta(z)). \tag{48}$$
$$\kappa_{TE-TM}^{(u)}(z) = \kappa_{TE-TM,0} sin\theta((z)), \qquad \kappa_{TE-TM}^{(v)}(z) = \kappa_{TE-TM,0} cos(\theta(z)).$$

The waveguide-resolved detected intensity is then written as

$$I_n(z) = |a_{n,TE}(z)|^2 + |a_{n,TM}(z)|^2. \tag{49}$$

The influence of this additional polarization channel is further reduced by the propagation-constant mismatch between the TE and TM modes. After subtracting a common propagation constant, the two polarization sectors are separated by $\Delta\beta = \beta_{TM} - \beta_{TE}$, which acts as a detuning in the coupled-mode Hamiltonian. When this detuning is large compared with the cross-polarization coupling and is away from a Floquet-assisted resonance, TE-TM transfer is suppressed and the TM sector contributes primarily as a perturbative correction to the TE-dominated dynamics.

Consistent with this picture, the recalculated dynamics retain the characteristic period-Λ and period-2Λ behavior while exhibiting changes in the detailed site-resolved intensities. We therefore use the extended two-polarization model to assess how residual TE-TM coupling may affect the calculated propagation pattern, rather than to provide an exact quantitative description of the full polarization dynamics."

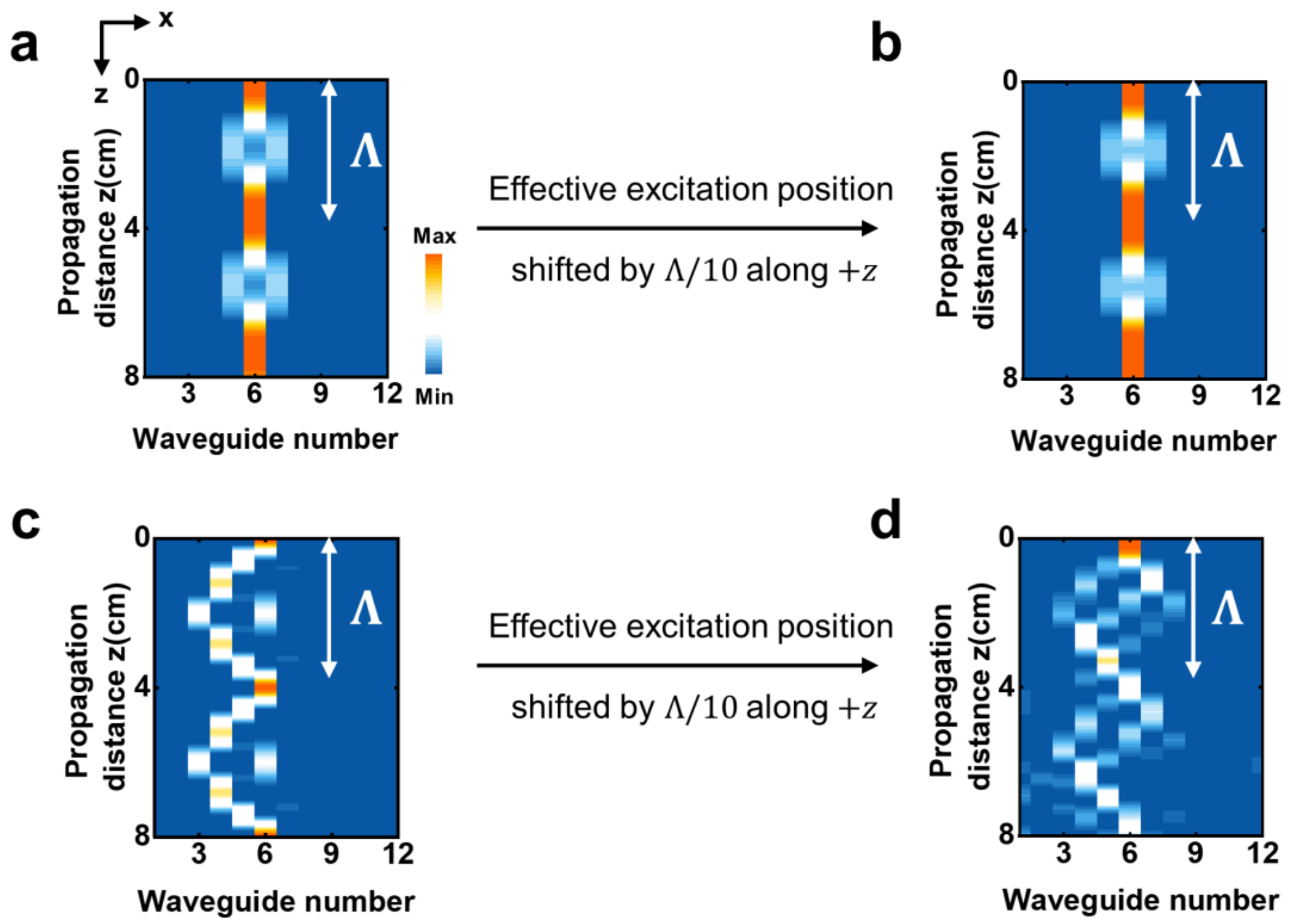

Fig. S35 | Sensitivity of the propagation dynamics to the effective excitation position. *The samples were originally fabricated with horn-shaped input tapers for the THz-TDS measurements. After switching to the VNA configuration, these tapers were mechanically removed to enable direct coupling from the WR-4 metallic waveguide to the dielectric-waveguide facet, resulting in a small forward displacement (corresponding to approximately $\Lambda/10$) of the experimental input facet along the $+z$ direction.* ***a,b,*** *TB propagation dynamics for the configuration corresponding to Fig. 3, in which the two couplings adjacent to the excited waveguide have the same longitudinal dependence, being either both $\sin[\theta(z)]$-like or both $\cos[\theta(z)]$-like.* ***c,d,*** *Corresponding propagation dynamics for the configuration in Fig. 4, in which the two adjacent couplings follow $\sin[\theta(z)]$ −like and $\cos[\theta(z)]$-like dependences, respectively. Panels* ***a*** *and* ***c*** *assume excitation at the original starting position, whereas panels* ***b*** *and* ***d*** *use a representative forward shift of $\Lambda/10$ along the $+z$ direction to illustrate the sensitivity to the excitation position. The propagation pattern in Fig. 3 changes only weakly under this shift, while that in Fig. 4 shows a more pronounced change because the shift modifies the relative values of the two adjacent couplings at the excitation position, thereby changing the initial power distribution toward the two sides and the subsequent interference dynamics. All coupling parameters are otherwise kept unchanged.*

Comment 5:

(1) The newly added loss measurements reveal an additional challenge. The reported attenuation over the experimentally relevant propagation lengths approaches two orders of magnitude. The authors demonstrate that this is predominantly caused by material absorption rather than by the modulation itself. While this clarification is valuable, it also raises questions regarding the suitability of the chosen experimental platform for demonstrating the proposed concept.

(2) A further consequence of the strong losses is that the manuscript currently lacks experimental validation of the 2D implementations (a fact that slipped by me in the first revision round), and the authors state that the attenuation will also likely prevent practical measurements on the relevant length scales for this 2D system in the future.

Response:

We sincerely thank the Reviewer for raising this important point. We agree that the measured attenuation places a substantial limitation on the propagation length accessible in the present 3D-printed resin platform and, in particular, makes an experimental observation of the long-distance chiral reversal of the 2D topological edge state difficult. We have therefore revised the manuscript to state more clearly that

(1) The present RG-resin platform was selected as a practical proof-of-principle platform for fabricating twisted waveguides and experimentally validating polarization-controlled Floquet coupling, rather than because it represents the only or intrinsically optimal material system for demonstrating these phenomena. RG resin was chosen primarily because it is readily accessible for 3D printing, economically practical, and well established in the fabrication workflow of the commercial printing provider. The corresponding printing parameters, dimensional tolerances, and processing conditions were therefore already relatively mature, substantially reducing the number of additional fabrication trials required. We agree that the material absorption limits long-

distance propagation and therefore do not regard this resin system as an optimized platform for large-scale implementations. The present experiments are intended to validate the underlying polarization-controlled coupling and representative Floquet dynamics, whereas experimental observation of long-distance 2D topological transport will require a lower-loss platform.

(2) To experimentally test the local multidirectional coupling mechanism required for a future two-dimensional geometries, we newly fabricated and experimentally measured an elementary four-site, three-arm coupling unit. The structure consists of one central waveguide coupled to three surrounding waveguides along three different in-plane directions, with $a = 350$ um, $b = 150$ um, $d = 950$ um, $\lambda = 1200$ um. For the sign-changing configuration, the three couplings are $\kappa_1(z) = \kappa_0\cos(\theta(z) + \theta_0)$, $\kappa_2(z) = \kappa_0\cos(\theta(z) + \theta_0 - \frac{2\pi}{3})$, and $\kappa_3(z) = \kappa_0\cos(\theta(z) + \theta_0 - \frac{4\pi}{3})$. A reference structure was also measured in which the corresponding couplings are $\kappa_1(z) = \kappa_0|\cos(\theta(z) + \theta_0)|$, $\kappa_2(z) = \kappa_0\left|\cos(\theta(z) + \theta_0 - \frac{2\pi}{3})\right|$, and $\kappa_3(z) = \kappa_0\left|\cos(\theta(z) + \theta_0 - \frac{4\pi}{3})\right|$. The two structures therefore retain the same coupling-magnitude modulation while differing in the coupling signs. The transverse intensity distributions were experimentally measured at a propagation distance of $z = 3$ cm, as shown in Fig. R6. The experimentally observed output distributions distinguish these two cases and agree with the corresponding numerical results. This measurement provides direct experimental support for the multidirectional polarization-controlled coupling modulation required by the proposed 2D implementation.

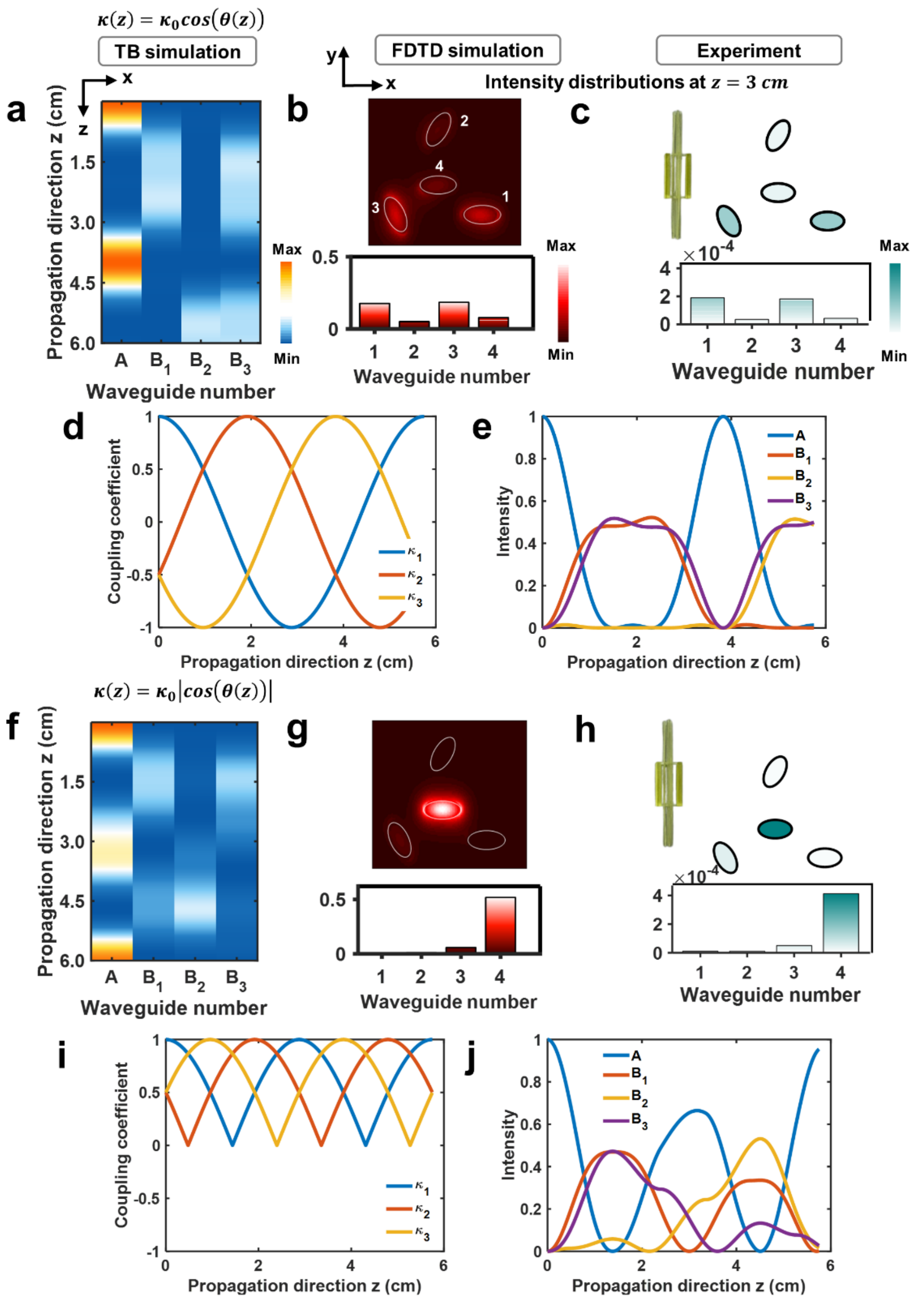

Fig. R6 | Experimental validation of multidirectional polarization-controlled coupling in a four-site unit. A central waveguide A is coupled to three surrounding waveguides B_1, B_2, and B_3, oriented along three in-plane directions separated by $\frac{2\pi}{3}$. The waveguides have elliptical cross-sections with $a = 350$ μm and $b = 150$ μm, a centre-to-centre separation of $d = 950$ μm, and an operating wavelength of $\lambda = 1200$ μm. **a–e,** Sign-changing coupling modulation. **a,** TB evolution for excitation of the central waveguide A. **b, c,** Corresponding FDTD-simulated and experimentally measured transverse intensity distributions at $z = 3$ cm, respectively. In **c**, the

filling of each ellipse represents the measured output intensity in the corresponding waveguide. **d**, Coupling coefficients between A and the three surrounding waveguides. **e**, TB intensity evolution in the four waveguides. **f-j**, Corresponding results for the reference configuration without changing the sign of coupling. **f**, TB evolution. **g, h,** FDTD-simulated and experimentally measured transverse intensity distributions at $z = 3$ cm, respectively. **i**, Coupling coefficients. **j**, Corresponding TB intensity evolution. The pale-yellow images at the lower left of c and h are photographs of the newly fabricated samples.

To provide a quantitative assessment of the newly fabricated unit-level experiments, we also calculated D_{NSE} and F between the FDTD-simulated and experimentally measured output distributions for both the 1D two-waveguide unit introduced in response to Comment 2 and the four-site multidirectional coupling unit introduced here. The results are summarized in Table R6.

Table R6 | Quantitative comparison between FDTD simulations and experimental measurements for the newly fabricated coupling-unit samples. The normalized squared Euclidean distance D_{NSE} and classical distribution fidelity F are calculated between the normalized FDTD-simulated and experimentally measured output intensity distributions at $z = 3$ cm. The 1D unit consists of two coupled waveguides and compares the sign-changing coupling $\kappa(z) = \kappa_0 cos(\theta(z))$ with the corresponding unsigned coupling $\kappa(z) = \kappa_0 |cos(\theta(z))|$. The four-site unit consists of one central waveguide coupled to three surrounding waveguides through $\kappa(z) = \kappa_0 cos(\theta(z) - \theta_i)$, and compares this configuration with the corresponding unsigned couplings $\kappa(z) = \kappa_0 |cos(\theta(z) - \theta_i)|$, where $i = 1,2,3$. A small D_{NSE} and a value of F close to unity indicate greater agreement.

Structure	1D unit		2D unit	
Coupling coefficient	$\kappa(z) = \kappa_0 cos(\theta(z))$	$\kappa(z) = \kappa_0 \|cos(\theta(z))\|$	$\kappa(z) = \kappa_0 cos(\theta(z))$	$\kappa(z) = \kappa_0 \|cos(\theta(z))\|$
D_{NSE}	0.0000761	0.0000577	0.00288	0.000584
F	0.9994	0.9997	0.9868	0.9928

We emphasize that this elementary-unit experiment is not presented as a full experimental realization of the complete two-dimensional topological lattice or of the predicted chiral reversal of its edge state. Rather, it experimentally validates the local coupling mechanism underlying the proposed 2D implementation, namely, the simultaneous polarization-controlled modulation of the coupling coefficients along multiple in-plane directions. The full chiral-reversal dynamics remain a theoretical

prediction in the present work because their required propagation length exceeds that practically accessible in the current resin platform. Nevertheless, other 2D effects requiring shorter propagation distances may be experimentally accessible. We have also characterized the optical properties of alumina ceramic and numerically evaluated the attenuation of porous resin waveguides, as discussed in our response to Comment 6. These approaches may provide possible routes toward longer-distance implementations. For the porous-waveguide design, a 20-cm-long 2D lattice considered for observing chirality reversal would correspond to an estimated propagation loss of approximately 49.5 dB. This value remains below the dynamic range of the VNA system, although additional coupling and insertion losses would also need to be considered in a future experimental implementation. We have revised the manuscript accordingly to distinguish clearly between the experimentally demonstrated local 2D coupling mechanism and the longer-distance 2D topological phenomenon.

Changes in the manuscript:

Main text:

Introduction:

We have added these sentences to the third paragraph of "**Introduction**".

"We showcase the broad utility of this platform through experimental demonstrations of representative driven phenomena in one-dimensional lattices and local multidirectional coupling in a two-dimensional configuration, together with a theoretical prediction of topological edge transport in two-dimensional lattices."

"Second, exploiting the ability to realize positive and negative couplings with unequal magnitudes, we theoretically predict the chirality reversal of the topological edge states in two-dimensional (2D) honeycomb lattices, illustrating the potential of the coupling scheme for higher-dimensional Floquet engineering (Fig. 1d)."

We have added these sentences to the first paragraph of the section entitled "**Theoretical Model of Polarization-Controlled Coupling in Twisted Waveguides**".

"The quantitative comparison yields distribution fidelities F of 0.9994 and 0.9997 for panels (ii) and (iv), respectively, while the corresponding normalized squared Euclidean distances D_{NSE} are 0.00008 and 0.00006. These values indicate that the relative intensity distribution between the two output waveguides is consistently reproduced in the FDTD simulations and experiments."

We have also added these sentences to the first paragraph of the section entitled "**Topological Phase Transitions and Chirality Reversal of Edge States**".

"Before considering the full 2D lattice, we first consider a four-site, three-arm unit in which a central waveguide is coupled to three neighboring waveguides through couplings with relative phase shifts. To demonstrate the effect of longitudinal coupling-sign reversal, we compare the signed couplings, described by $\kappa_1(z) = \kappa_0 cos(\theta(z))$, $\kappa_2(z) = \kappa_0 cos(\theta(z) - 2\pi/3)$, and $\kappa_3(z) = \kappa_0 cos(\theta(z) - 4\pi/3)$, with the corresponding unsigned couplings, described by their absolute values $\kappa_1(z) = \kappa_0 |cos(\theta(z))|$, $\kappa_2(z) = \kappa_0 |cos(\theta(z) - 2\pi/3)|$, and $\kappa_3(z) = \kappa_0 |cos(\theta(z) - 4\pi/3)|$. The FDTD simulations predict distinct output intensity distributions for the two cases, and the experiment reproduces the main contrast in the relative intensities across the output sites, as shown in Fig. 5a. The quantitative comparison yields distribution fidelities F of 0.9868 and 0.9928 for panels (ii) and (iii), respectively, while the corresponding normalized squared Euclidean distances D_{NSE} are 0.00288 and 0.00058. These values support the consistency between the FDTD-simulated and experimentally measured output distributions."

"We then theoretically investigate the chirality reversal of Floquet edge states produced by varying the driving period in a 2D honeycomb lattice. The calculated phase diagram and TB propagation dynamics illustrate how polarization-controlled couplings could be used for higher-dimensional Floquet engineering."

Figures:

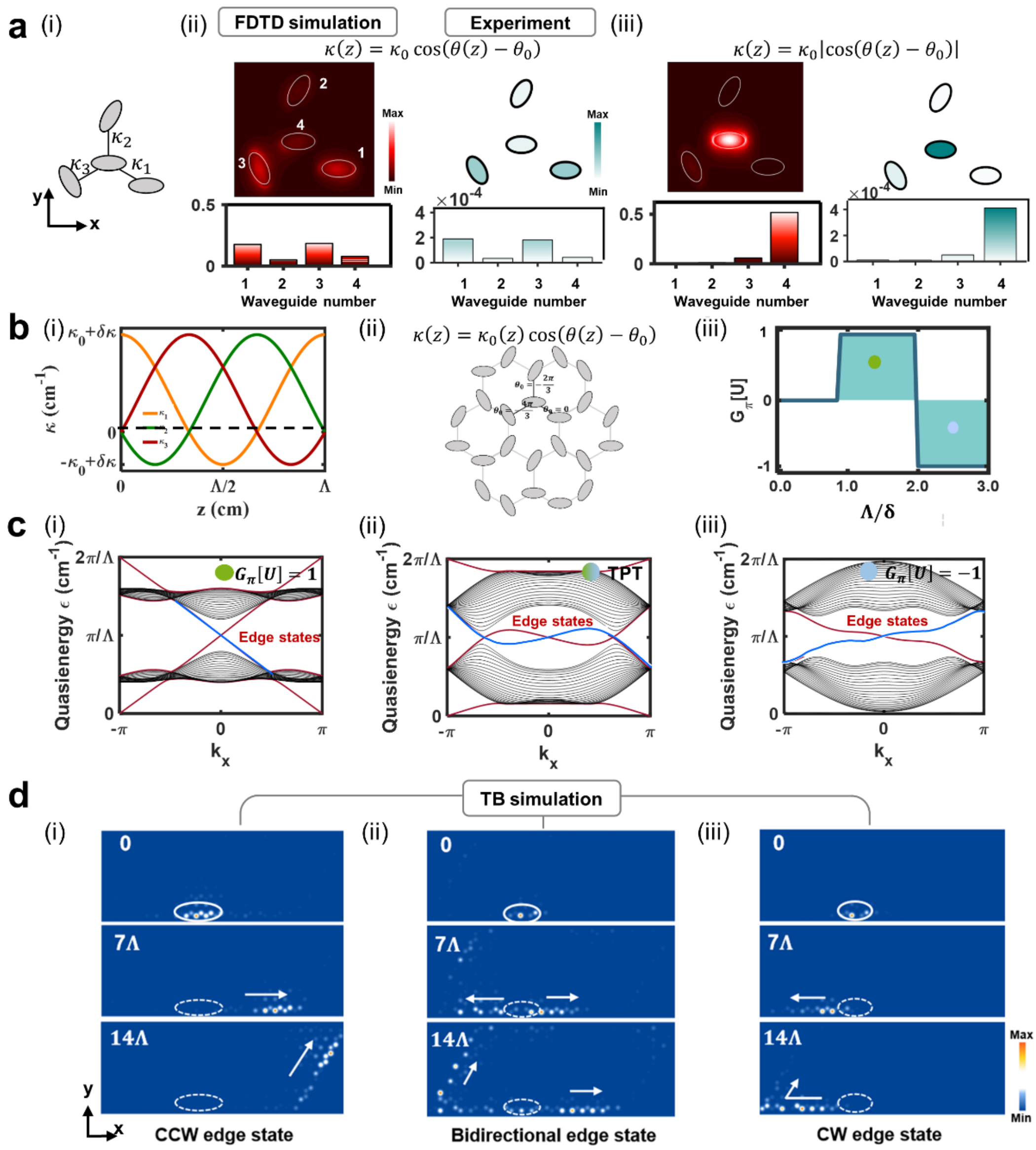

*Fig. 5 | **Multidirectional coupling and chirality reversal in two-dimensional Floquet photonic lattices. a, FDTD-simulated and experimental verification of the effect of longitudinal coupling-sign reversal in a four-site, three-arm unit.** (i) Cross-sectional schematic of the unit, in which a central waveguide is coupled to three neighboring waveguides through κ_1, κ_2, and κ_3. (ii) FDTD-simulated and experimentally measured output intensity distributions for the signed couplings $\kappa_1(z) = \kappa_0 cos(\theta(z))$, $\kappa_2(z) = \kappa_0 cos(\theta(z) - 2\pi/3)$, and $\kappa_3(z) = \kappa_0 cos(\theta(z) - 4\pi/3)$. (iii) Corresponding results for the unsigned couplings $\kappa_1(z) = \kappa_0|cos(\theta(z))|$, $\kappa_2(z) = \kappa_0|cos(\theta(z) - 2\pi/3)|$, and $\kappa_3(z) = \kappa_0|cos(\theta(z) - 4\pi/3)|$. **b,** Topological properties of the 2D lattice. **(i)** Coupling coefficient κ_1, κ_2 and κ_3 as a function of propagation distance z. **(ii)** Schematic of the cross section of the twisted waveguide array. **(iii)** Phase diagram of the driven system indicating the emergence of topological phases. **c,** Quasi-energy spectra for different types of edge states in 2D lattices under open boundary conditions projected along the zigzag edge. **(i)** CCW propagating edge state under one topological phase. **(ii)** Bidirectional edge states at the*

topological phase transition (TPT) point. ***(iii)*** *Clockwise (CW) propagating edge state. Red and blue curves indicate topologically protected edge states.* ***d,*** *TB simulated propagation of 2D Floquet edge states.* ***(i)*** *Excitation at the lower edge shows robust CCW edge transport.* ***(ii)*** *At the TPT point, bidirectional edge states emerge.* ***(iii)*** *Edge transport direction reverses to CW. White dashed circles highlight the injected regions.*

Supplementary Information:

We have added Fig. R6 to Supplementary Fig. S31.

We have also added Table R6 to Supplementary Table S2.

Comment 6:

Overall, I continue to find the central idea of the paper interesting and potentially valuable for researchers working on waveguide-based simulators and artificial gauge fields. As Reviewer 3 noted, the approach could ultimately provide a streamlined platform for coupling modulation and the implementation of arbitrary gauge-field engineering. Whether the concept can be successfully transferred to low-loss optical waveguide platforms remains an important open question.

Response:

We thank the reviewer for highlighting the important question of whether the proposed concept can be transferred to lower-loss optical waveguide platforms. To address this point, we discuss three potential complementary implementation routes. First, subwavelength air holes may be introduced into the printed resin waveguides to reduce the modal overlap with the lossy material while retaining the geometric flexibility of 3D printing. Second, the resin may be replaced with high-purity alumina ceramic, providing a lower-loss material platform that remains compatible with 3D-printing techniques. Third, the proposed concept may be transferred to femtosecond-laser-written optical waveguides. These three approaches provide possible routes for extending the present concept toward lower-loss waveguide platforms.

I. Low-loss designs compatible with 3D printing

(i) Waveguides with subwavelength air holes

A possible future route within polymer-based terahertz platforms is to introduce subwavelength porosity[1,2] into the waveguide cross-section. By reducing the modal overlap with the absorbing polymer, porous waveguides can lower material-induced attenuation, while previous studies have shown that substantial birefringence can be retained or even enhanced[3]. Realizing this route would require dedicated optimization and experimental characterization of the porous twisted geometry. We therefore present it here only as a possible structural concept for reducing the modal overlap with the absorbing material and thereby lowering the propagation loss. At 0.26 THz, after 4 cm of propagation, the estimated propagation losses are approximately 34.0 dB for the solid RG-resin waveguide, which remains within the measurable range of the VNA setup, and 9.90 dB for the porous RG-resin waveguide.

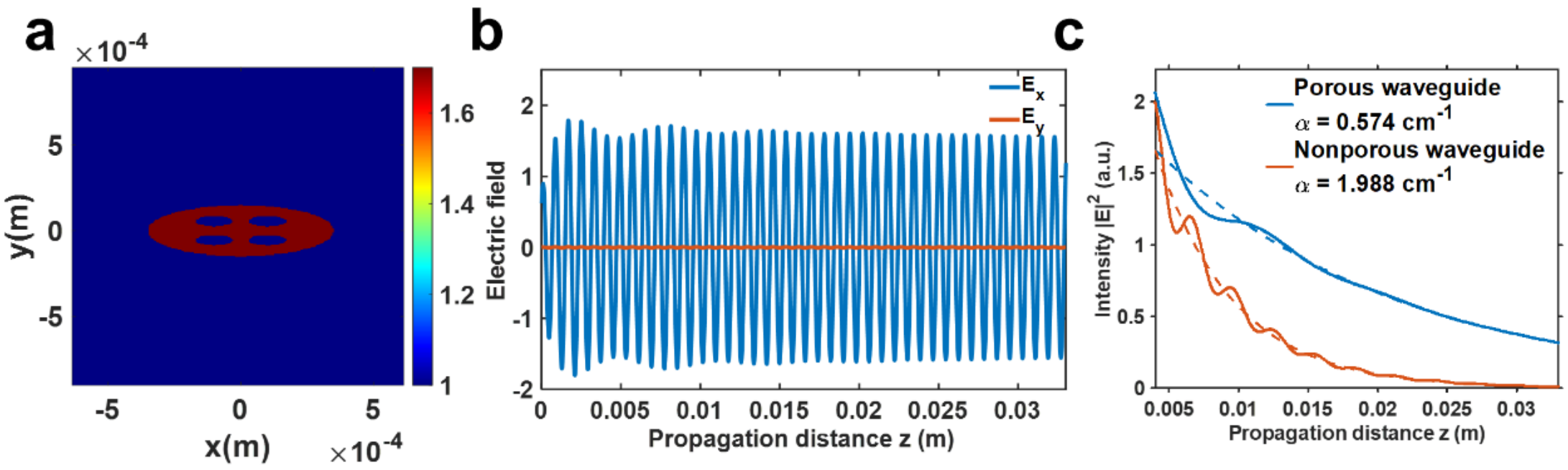

Fig. R7 | Polarization preservation and reduced propagation loss in a porous resin waveguide. **a,** Transverse refractive-index distribution of the proposed elliptical resin waveguide containing four symmetrically arranged subwavelength air holes. The major and minor semi-axes of the waveguide are 350 and 150 μm, respectively, while those of each air hole are 70 and 30 μm. **b,** FDTD-calculated evolution of the electric-field components E_x and E_y over a propagation distance of 30λ, for an input polarization aligned with the major axis of the elliptical waveguide. The dominant E_x component and strongly suppressed E_y component indicate that the guided mode remains predominantly linearly polarized along the intended direction. **c**, Comparison of the FDTD-calculated intensity in the porous and nonporous resin waveguides under identical excitation conditions. The porous waveguide exhibits a slower decay and retains a larger power after 30λ of propagation, indicating reduced attenuation owing to the decreased modal overlap with the lossy

resin material.

(ii) Optical characterization of alumina ceramic as a possible lower-loss material

As a further possible route toward a lower loss implementation, high-precision 3D printing of alumina ceramics may be considered for THz waveguides. The commercial CA-100A alumina slurry is compatible with 10-μm-class printing equipment. Our measurements further show that, over 0.24-1.3 THz, the absorption coefficient of the alumina ceramic remains consistently lower than that of the RG resin used in the present experiments, as shown in Fig. R8. At 0.26 THz, after 4 cm of propagation, the estimated propagation losses are approximately 34.0 dB for the RG-resin waveguide, which remains within the measurable range of the VNA setup, and 12.2 dB for the alumina-ceramic waveguide. These results suggest that 3D printed alumina ceramics may provide a practical route to reducing material absorption while retaining sufficient geometric flexibility for polarization-controlled THz waveguide designs.

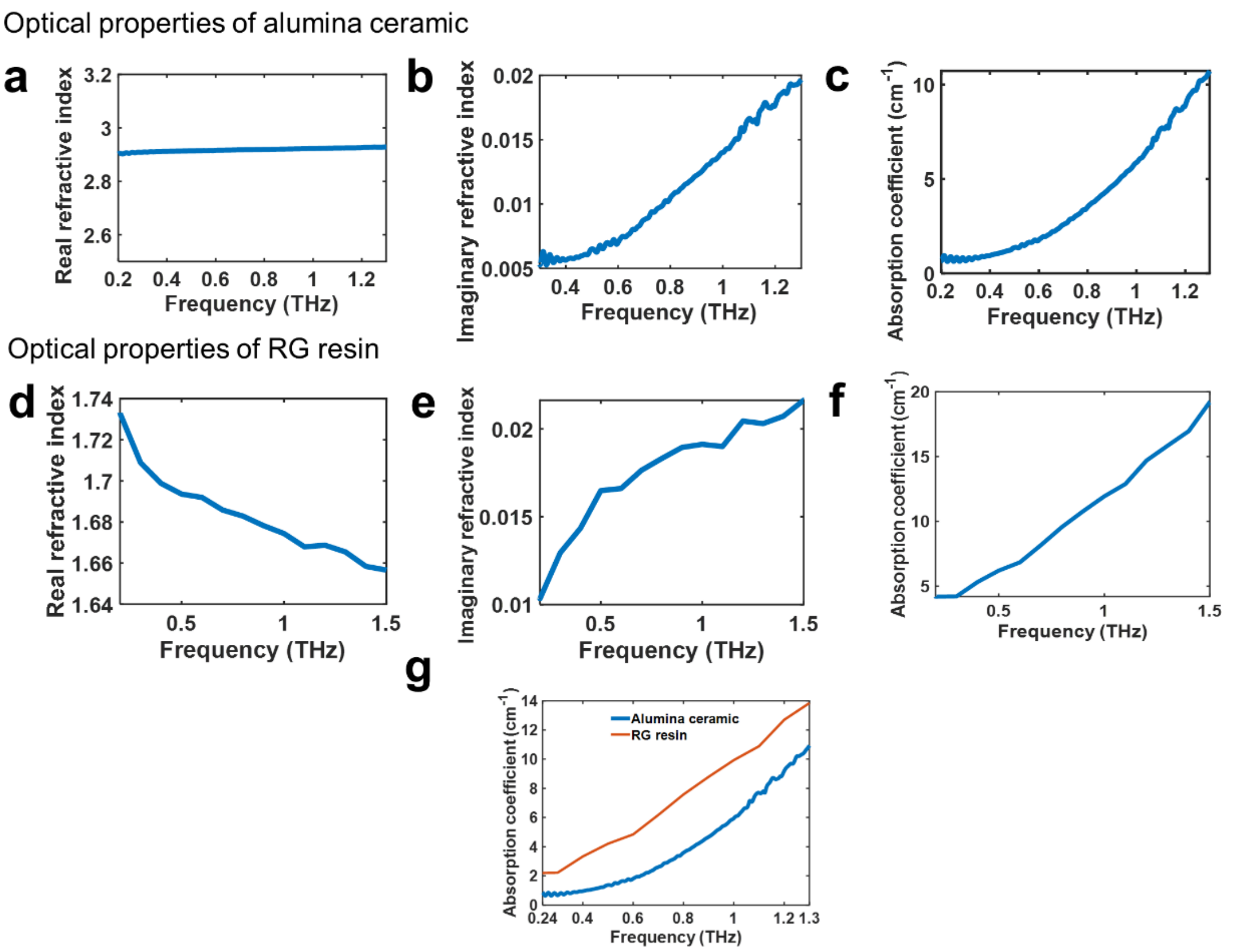

Fig. R8 | Optical properties of alumina ceramic. a–c, Real part of the refractive index, imaginary

part of the refractive index, and absorption coefficient, respectively, of the alumina ceramic measured by TDS over 0.2-1.3 THz. **d–f,** Corresponding quantities of the RG resin measured by TDS over 0.24-1.3 THz. **g,** Comparison between the measured absorption coefficients of the alumina ceramic and the RG resin used in the present experiments over 0.24-1.3 THz, showing consistently lower absorption in the alumina ceramic.

II. Discussion on possible implementation in femtosecond-laser-written optical waveguides

Inspired by the femtosecond-laser-written waveguide systems reported in the references cited by the reviewer, we considered whether the required rotating birefringent structure could be implemented using three-dimensional laser-written waveguide technique, extending the working wavelength from terahertz regime to visible and near-infrared band. In such a design, two partially overlapping tracks could follow mutually symmetric helical trajectories around a common straight centerline, so that the effective birefringent principal axis rotates continuously while the overall propagation axis remains straight. This possibility combines previously demonstrated double-track polarization control[4] with the established three-dimensional writing of helical Floquet waveguides[5]. However, the required track separation, overlap, helical radius, and pitch have not been experimentally optimized for the present coupling protocol, and we therefore present this only as a possible future implementation rather than as a demonstrated low-loss realization.

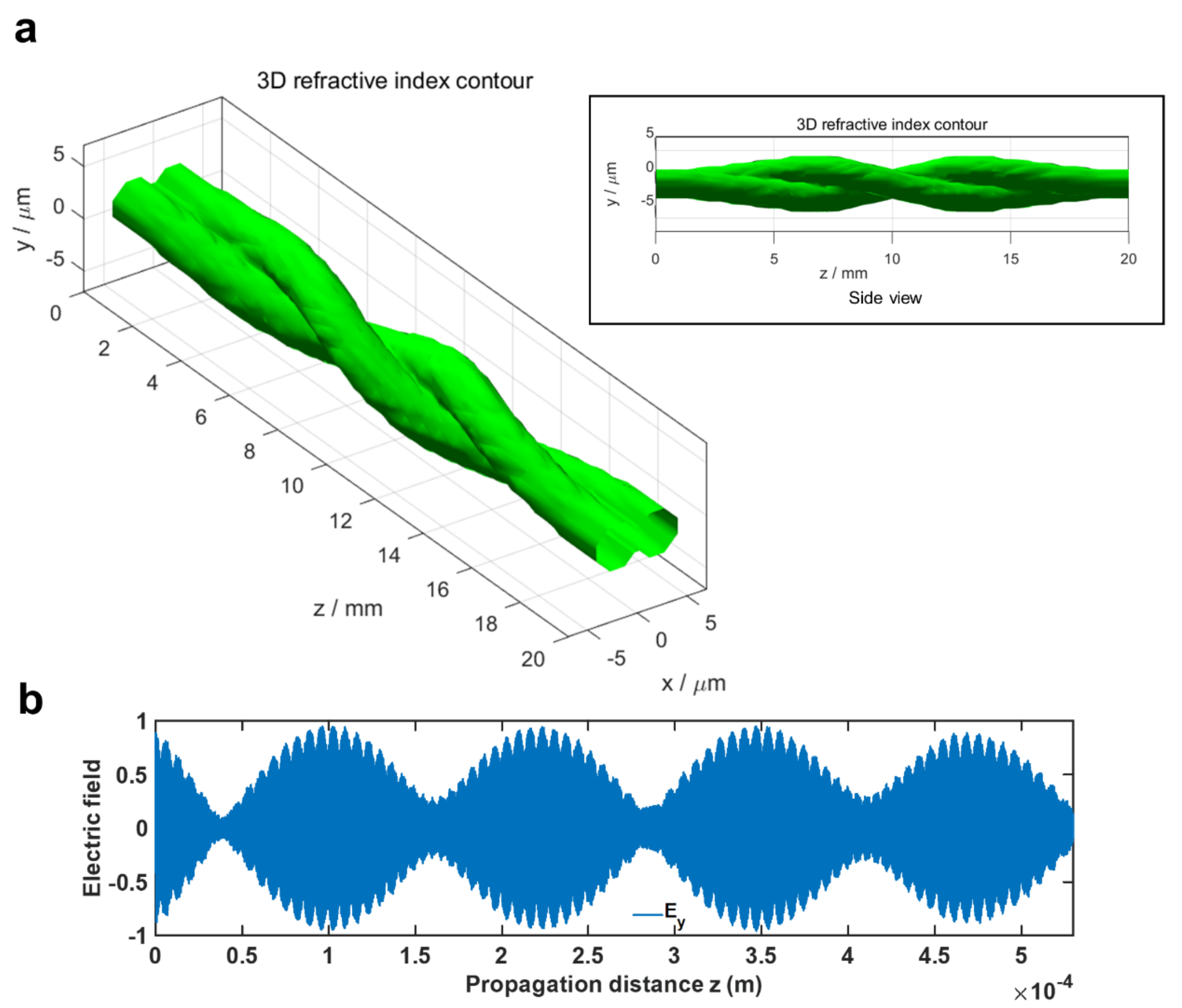

Fig. R9 | Conceptual implementation in femtosecond-laser-written waveguides. a, Three-dimensional refractive-index contour of a proposed double-track birefringent waveguide formed by two laser-written tracks following mutually symmetric helical trajectories around a common straight centerline. The relative orientation of the two tracks, and hence the effective birefringent principal axis of the combined waveguide, rotates continuously along the propagation direction while the overall waveguide centerline remains straight. The inset shows the corresponding side view of the model. The illustrated structural parameters are designed for operation at a wavelength of 785 nm. **b**, Simulated evolution of the E_y field component along the twisted double-track waveguide, showing that the optical polarization follows the continuously rotating birefringent axis during propagation. To reduce the computational domain and memory requirements, a shorter twist period than that illustrated in **a** is used in this simulation.

Changes in the manuscript:

We have added these sentences to the first paragraph of the section entitled **"Conclusion".**

"We acknowledge that the relatively high absorption loss of the resin platform used

in the present experiments limits the observation of phenomena over substantially longer propagation distances. Although the present experiments are implemented in 3D-printed resin waveguides, the concept may be extended to lower-loss platforms through several complementary approaches, such as porous waveguides with reduced modal overlap with the absorbing material and 3D-printed high-purity alumina ceramic waveguides. Further optimization of material processing and waveguide geometry may improve the performance of these implementations. With continued advances in femtosecond-laser-writing techniques, the concept may also be transferable to waveguide platforms operating in the visible and near-infrared regimes."

Supplementary Information:

We have added Fig. R7 to **Supplementary Fig. S32** and added Fig. R8 to **Supplementary Fig. S33**.

Comment 7:

Nevertheless, in its current form, I do not believe that the experimental evidence reaches the standard expected for publication in Nature Communication's broad-scope, high-impact journal. The conceptual contribution is interesting, but the experimental validation remains insufficiently convincing. I would therefore recommend transfer to a more specialized venue, such as Communication Physics or a field-specific optics/photonics journal, where the work's conceptual advances could still be of considerable interest despite the current experimental limitations.

Response:

We thank the Reviewer for providing this candid overall assessment. We understand that the principal concern is whether the experimental evidence is sufficiently convincing for the scope of the journal. In response, we have undertaken substantial additional work in this revision, including repeated measurements to assess experimental repeatability, new experiments on representative 1D and local 2D coupling units, quantitative comparisons between the measured and simulated intensity

distributions, and a more cautious discussion of the scope and limitations of the present experimental validation. We have also clarified that the full 2D chirality-reversal dynamics remain a theoretical prediction in the present work, whereas the local multidirectional coupling mechanism underlying the proposed 2D implementation and representative 1D Floquet dynamics are supported experimentally. We hope that these additions and clarifications address the Reviewer's remaining concerns and allow the suitability of the revised manuscript to be reconsidered.

Reply to Reviewer 2:

General comment:

While the original submission was relatively unpolished and gave the impression of having been prepared in haste, the revised manuscript presents a much more convincing case for combining polarization-controlled coupling with Floquet modulation. The authors have substantially rewritten the manuscript, addressed criticism by the reviewers, making it considerably easier to follow and clarifying both the motivation and the advantages of the proposed approach. They have also placed their work more clearly in the context of previous studies in which topological photonic phases rely on polarization-dependent coupling, while emphasizing the added role of Floquet modulation.

I believe that this substantially revised version will be of interest to the readers of *Nature Communications*, and I recommend it for publication in its present form.

Response:

We sincerely thank the Reviewer for the positive assessment of our revised manuscript and for recommending its publication. We greatly appreciate the Reviewer's constructive comments, which have helped us improve the clarity, motivation, and presentation of the work.

Reply to Reviewer 3:

General comment:

The authors have answered my questions sufficiently. I believe the representation of the data is much more streamlined and intelligible, with more speculative examples placed in the supplementary. While I think the novelty of the work is still not completely ground breaking, I still think the work may warrant publication.

Response:

We thank the Reviewer for acknowledging that our revisions have sufficiently addressed the questions and improved the clarity and presentation of the manuscript. We also appreciate the Reviewer's assessment that the work may warrant publication.

References

1. Hassani, A., Dupuis, A. & Skorobogatiy, M. Porous polymer fibers for low-loss Terahertz guiding. *Opt. Express* **16**, 6340 (2008).

2. Hassani, A., Dupuis, A. & Skorobogatiy, M. Low loss porous terahertz fibers containing multiple subwavelength holes. *Appl. Phys. Lett.* **92**, 071101 (2008).

3. Atakaramians, S., Afshar V., S., Fischer, B. M., Abbott, D. & Monro, T. M. Low loss, low dispersion and highly birefringent terahertz porous fibers. *Opt. Commun.* **282**, 36–38 (2009).

4. Wang, C.-Y., Gao, J. & Jin, X.-M. On-chip Rotated Polarization Directional Coupler Fabricated by Femtosecond Laser Direct Writing. *Opt. Lett.* **44**, 102 (2019).

5. Rechtsman, M. C. *et al.* Photonic Floquet topological insulators. *Nature* **496**, 196–200 (2013).

Dear reviewers:

Thank you for taking the time to review our manuscript entitled "Polarization-controlled Floquet engineering in twisted photonic lattices" (Manuscript ID: NCOMMS-25-101957).

We greatly appreciate your valuable comments and suggestions, which have been carefully considered in our revisions. Below, we have provided a point-by-point response, along with the corresponding changes made in the revised manuscript and Supplementary Information for your reference.

Comment 1:

The explanation of why the refractive index requires a global shift could be clarified and adapted accordingly. The wording of the origin of the refractive index correction should be improved. It is not that the wavelength dependence of the coupling is responsible for the index shift, but that an assumed wrong refractive index lead to a slightly "wrong" intensity distribution. It would therefore be helpful if the reasoning behind this correction could be explained in a little more detail in the Supplementary Information, particularly in connection with Fig. S28.

Response:

We thank the Reviewer for this clarification. We agree that our previous wording could incorrectly suggest that the wavelength dependence of the coupling coefficient is responsible for the refractive-index correction. This is not our intended meaning. Rather, a small offset in the resin refractive index initially assumed in the FDTD simulations slightly changes the inter-waveguide coupling strength, which can accumulate over the propagation distance and lead to differences in the site-resolved intensity distribution.

We have therefore revised the corresponding description in the main text and added a more detailed explanation in the Supplementary Note 13 in connection with Fig. S28.

Changes in the manuscript:

Main text

We have added these sentences to the third paragraph of the section entitled "**Floquet Flat Bands and Period-nΛ Oscillations**".

"The simulated intensity distribution is sensitive to the refractive index assumed for the resin, because a small offset in this parameter changes the inter-waveguide coupling strength. The resulting small coupling mismatch can accumulate over the propagation distance and redistribute the site-resolved output intensity. We therefore independently calibrated a single global refractive-index correction using the straight waveguide control array, as detailed in Supplementary Fig. S28."

Supplementary Information

We have added these sentences to the final part of Supplementary Note 13.

"The refractive index measured from the bulk resin is used as the initial value in the FDTD simulations. However, the refractive index of the fabricated waveguides can be slightly different from this value. A small offset in the refractive index used in the simulation changes the inter-waveguide coupling strength. Over a long propagation distance, this small difference can lead to a noticeable change in the output intensity distribution.

To account for this effect, we determine a single refractive-index correction of $\Delta n = 0.02$ *from the straight-waveguide control sample. This correction is used to correct the small difference between the refractive index used in the original simulation and that corresponding to the fabricated waveguides. After* $\Delta n = 0.02$ *is determined, the same corrected refractive index is used for all wavelengths and propagation distances without further adjustment. As shown in Fig. S28, the corrected FDTD results show improved agreement with the experimental results at 1300, 1250, and 1200 µm."*

Comment 2:

There is a small typo in the Supplementary Information: in Fig. S29, "honetcomb" should be corrected to "honeycomb".

Response:

We thank the Reviewer for pointing out this typo. "honetcomb" has been corrected to "honeycomb" in Fig. S29.

Changes in the manuscript:

The typo in Fig. S29 has been corrected.

Comment 3:

The photographs of the structure in Fig. S30 are currently not helpful and rather small and therefore difficult to assess.

Response:

We thank the Reviewer for this helpful comment. We agree that the photographs previously included in Figs. S30 and S31 were too small to clearly show the fabricated structures. We have therefore removed these small photographs from Figs. S30 and S31

and relocated them to Fig. S18, where the fabricated resin samples are presented together. The enlarged photographs are now shown as Figs. S18e and S18f. Fig. S18e presents the three two-waveguide coupling units corresponding to the $\kappa(z) = \kappa_0\cos(\theta(z))$, $\kappa(z) = \kappa_0|\cos(\theta(z))|$, and $\kappa(z) = \kappa_0\sin(\theta(z))$ coupling profiles, while Fig. S18f presents the four-waveguide coupling units corresponding to the sign-changing and unsigned coupling configurations used for the local two-dimensional coupling experiments. This revised presentation allows the fabricated structures to be assessed more clearly.

Changes in the manuscript:

Supplementary Information

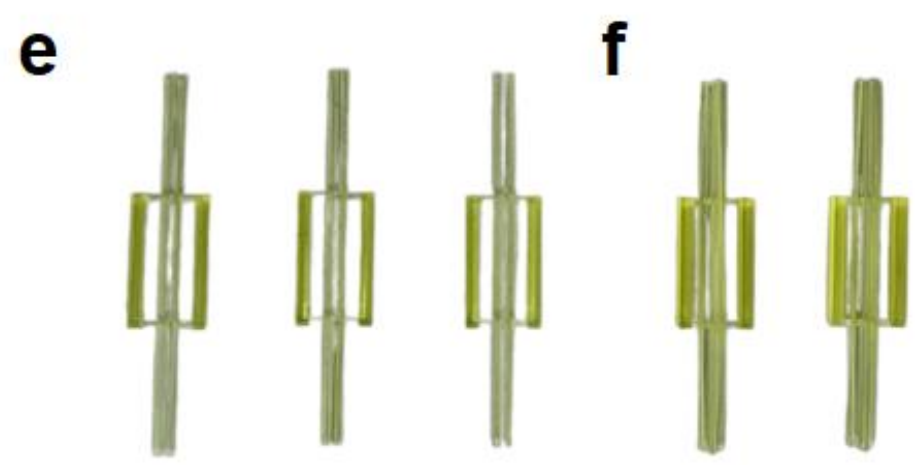

"Fig. S18 | Resin waveguide samples for the period-nΛ oscillation and topological state experiments, and local coupling experiments. e, Three fabricated two-waveguide coupling units corresponding to the longitudinal coupling functions $\kappa(z) = \kappa_0 cos(\theta(z))$, $\kappa(z) = \kappa_0|cos(\theta(z))|$, and $\kappa(z) = \kappa_0 sin(\theta(z))$, respectively. f, Fabricated four-waveguide coupling units used for the local two-dimensional coupling experiments, corresponding to the sign-changing coupling $\kappa_i(z) = \kappa_0 cos(\theta(z) + \theta_0 - \theta_i)$ $(i = 1, 2, 3)$ and the unsigned coupling $\kappa_i(z) = \kappa_0|cos(\theta(z) + \theta_0 - \theta_i)|$ $(i = 1, 2, 3)$, respectively."

Comment 4:

The unit "μm" appears to be missing for the values of 1300 and 1250 in the text of Table S2.

Response:

We thank the Reviewer for noting this omission. The missing unit "μm" has been added to the values 1300 and 1250 in the text of Table S2.

Changes in the manuscript:

Supplementary Information

We have added the unit "µm" to the relevant values in the caption of Table S2.

"The first table lists the normalized squared Euclidean distance D_{NSE} *and the distribution fidelity* F *at each sampled propagation distance* z *for Figs. 3a and for the straight-waveguide control sample at incident wavelengths of 1300* μm *and 1250* μm*."*

Comment 5:

For improved understanding, it might be helpful to number the waveguides in figures as for example in Fig. 5(a)(iii).

Response:

We thank the Reviewer for this suggestion. To improve readability, we have added explicit waveguide numbers to the relevant schematics and transverse intensity-distribution panels in Figs. 2, 5, S30, and S31, following the numbering convention used in Fig. 5a(iii). These labels make the correspondence between individual waveguides and their simulated or measured intensities clearer.

Changes in the manuscript:

We have added waveguide numbers to the relevant schematics and transverse intensity-distribution panels in Figs. 2, 5, S30, and S31.

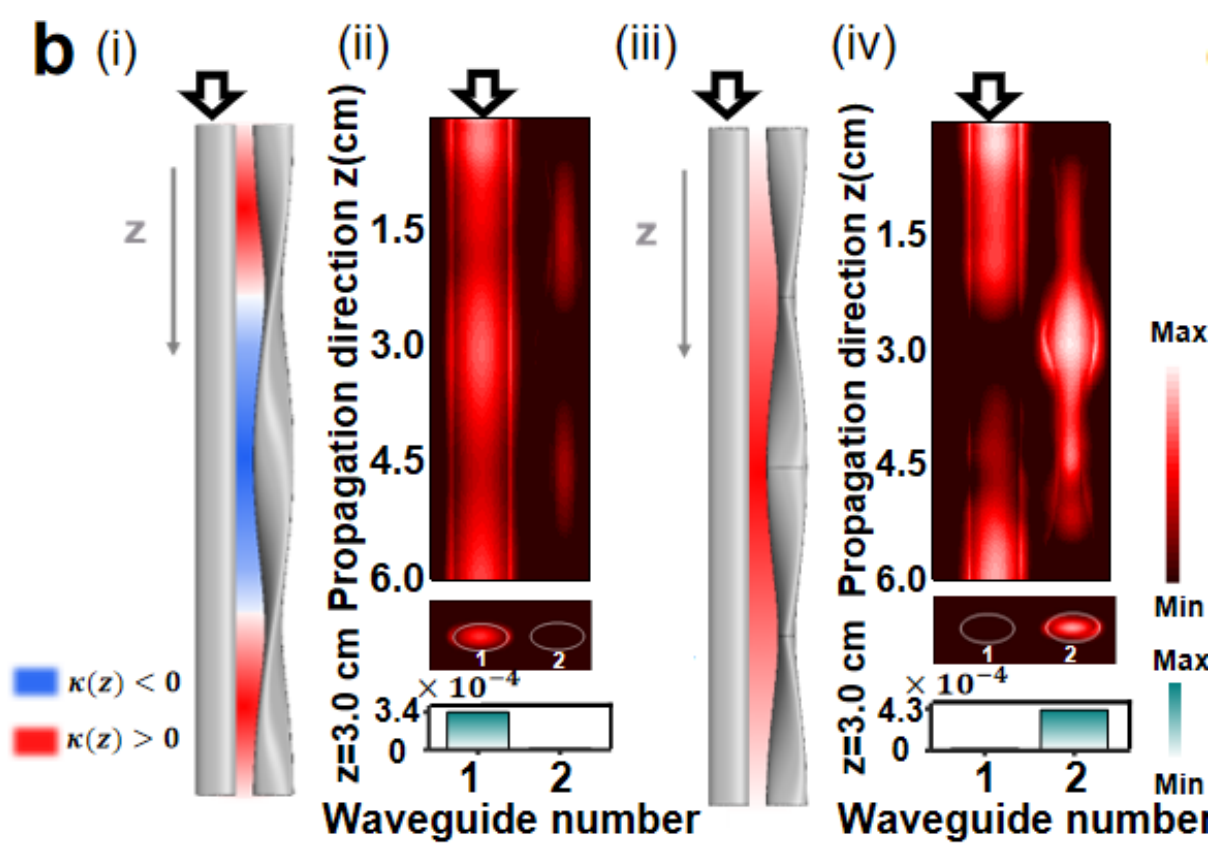

*"**Fig. 2b,** FDTD simulated and experimental verification of the effect of longitudinal coupling-sign reversal in two coupled waveguides. **(i–ii)**, one waveguide is twisted by*

*2π along the propagation direction, yielding $\kappa(z) = \kappa_0 cos\theta(z)$. **(iii–iv)**, the twisted waveguide undergoes alternating rotations of $\pm\pi/2$, yielding $\kappa(z) = \kappa_0|cos\theta(z)|$."*

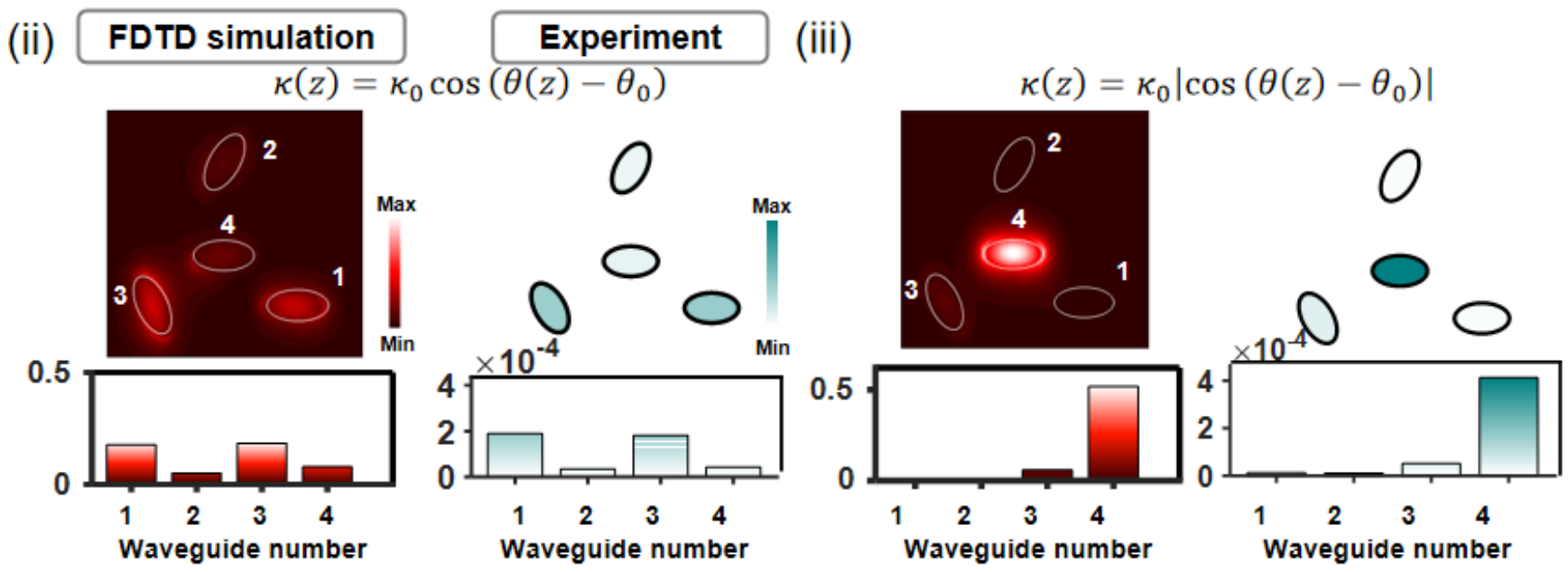

*"**Fig. 5a,** (ii) FDTD-simulated and experimentally measured intensity distributions for the couplings $\kappa_1(z) = \kappa_0 cos(\theta(z))$, $\kappa_2(z) = \kappa_0 cos(\theta(z) - 2\pi/3)$, and $\kappa_3(z) = \kappa_0 cos(\theta(z) - 4\pi/3)$. (iii) Corresponding results for the couplings $\kappa_1(z) = \kappa_0|cos(\theta(z))|$, $\kappa_2(z) = \kappa_0|cos(\theta(z) - 2\pi/3)|$, and $\kappa_3(z) = \kappa_0|cos(\theta(z) - 4\pi/3)|$."*

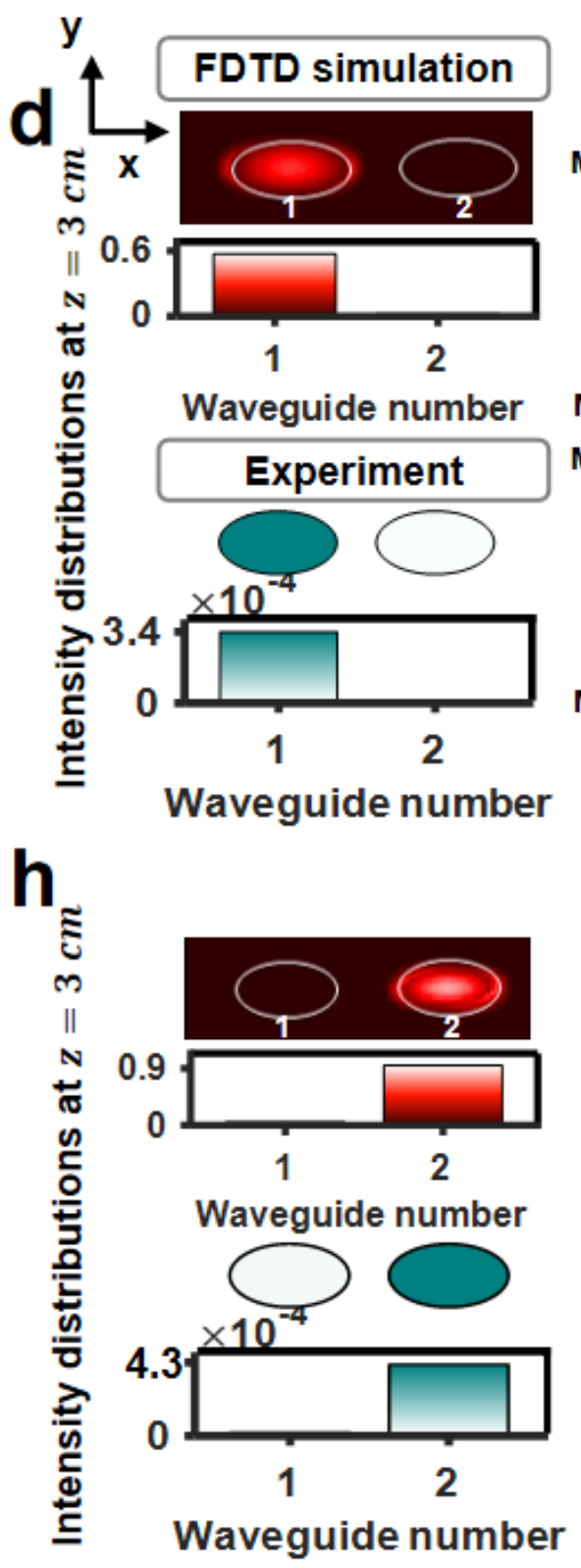

*"**Fig. S30d, h,** Transverse intensity distributions at $z = 3$ cm, obtained from FDTD simulations (upper), and the corresponding experimental measurements (lower). The cosine and absolute-cosine coupling functions have the same coupling magnitude $|\kappa(z)|$ at every propagation position, but differ in whether the coupling sign reverses."*

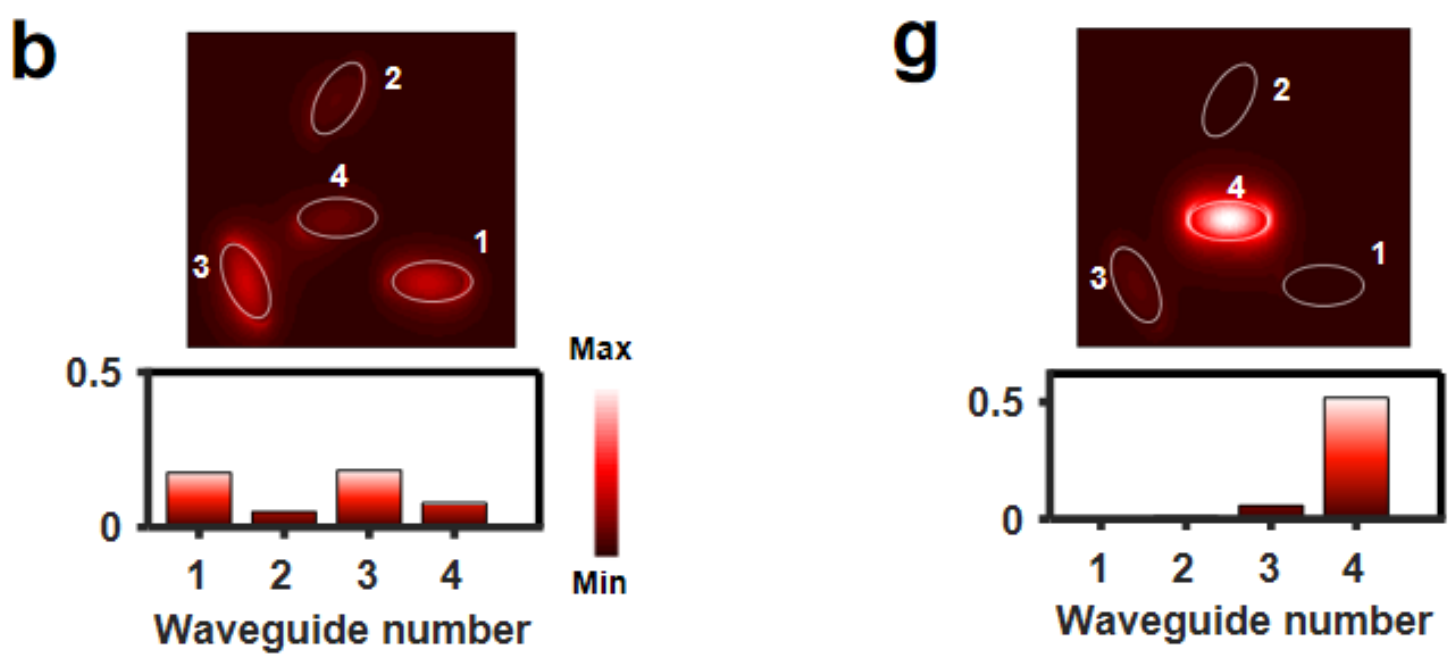

*"**Fig. S31b, g,** Corresponding FDTD-simulated transverse intensity distributions at* $z = 3\ cm$ *under sign-changing coupling modulation and sign-unchanging coupling modulation, respectively."*

Comment 6:

The text from the rebuttal explaining the added figures in the SI (Fig. S28-S36) and T1 and T2 should be added to the SI. Otherwise, the figures are hard for the reader to understand without any additional explanation.

Response:

We thank the Reviewer for this helpful suggestion. We agree that the newly added supplementary figures and tables should be understandable without referring to the rebuttal letter. In the previous revision, much of the detailed explanation associated with Figs. S28–S36 and Tables S1 and S2 was provided mainly in the rebuttal, while the Supplementary Information contained primarily the corresponding figure and table captions.

We have therefore incorporated these explanations into the Supplementary Information and reorganized them into four new Supplementary Notes. Supplementary Note 22 explains the refractive-index correction used in the FDTD simulations and the quantitative comparison summarized in Fig. S28 and Table S2. Supplementary Note 23 describes the local experimental validation of polarization-controlled coupling and provides the context for Figs. S30-S31. Supplementary Note 24 discusses possible lower-loss implementations corresponding to Figs. S32 and S33. Supplementary Note 25 presents the experimental repeatability and analyzes possible sources of the

remaining discrepancies, corresponding to Figs. S34–S36.

These additions provide the motivation, analysis, and physical interpretation of the supplementary results directly within the Supplementary Information, so that the figures and tables can now be understood independently of the rebuttal letter.

Changes in the manuscript:

Supplementary Information

We have added four new sections, Supplementary Notes 22–25, to provide the detailed explanations associated with Figs. S28, S30–S36 and Tables S1 and S2. The newly added sections are:

*"**Supplementary Note 22 -** Refractive-index correction in the FDTD simulations.*

***Supplementary Note 23 -** Experimental validation of local polarization-controlled coupling.*

***Supplementary Note 24 -** Possible lower-loss implementations.*

***Supplementary Note 25 -** Experimental repeatability and possible sources of the remaining discrepancies."*

Detailed explanations and discussions of the corresponding supplementary figures and tables are provided in the respective Notes.
